# GradientScanSurv—An exhaustive association test method for gene expression data with censored survival outcome

**DOI:** 10.1371/journal.pone.0207590

**Published:** 2018-12-05

**Authors:** Ming Yi, Ruoqing Zhu, Robert M. Stephens

**Affiliations:** 1 NCI RAS Initiative, Cancer Research Technology Program, Frederick National Laboratory for Cancer Research, Frederick, MD, United States of America; 2 Department of Statistics, University of Illinois Urbana-Champaign, Champaign, IL, United States of America; University of Oklahoma Health Sciences Center, UNITED STATES

## Abstract

Accurate assessment of the association between continuous variables such as gene expression and survival is a critical aspect of precision medicine. In this report, we provide a review of some of the available survival analysis and validation tools by referencing published studies that have utilized these tools. We have identified pitfalls associated with the assumptions inherent in those applications that have the potential to impact scientific research through their potential bias. In order to overcome these pitfalls, we have developed a novel method that enables the logrank test method to handle continuous variables that comprehensively evaluates survival association with derived aggregate statistics. This is accomplished by exhaustively considering all the cutpoints across the full expression gradient. Direct side-by-side comparisons, global ROC analysis, and evaluation of the ability to capture relevant biological themes based on current understanding of RAS biology all demonstrated that the new method shows better consistency between multiple datasets of the same disease, better reproducibility and robustness, and better detection power to uncover biological relevance within the selected datasets over the available survival analysis methods on univariate gene expression and penalized linear model-based methods.

## Introduction

### Overview of survival analysis with categorical variables

The realization of precision/personalized medicine requires quantitation of relevant prognostic biomarkers for each individual that guide their diagnosis and treatment. Of course, beyond the identification of disease subtypes, one component in the identification of the relevant panel of biomarkers is the association of those variables with patient survival outcomes [1–4]. These biomarkers can be generalized to represent variables that are categorical with a limited number of discrete values. Conveniently, categorized or discrete covariates including clinical features such as pathological cancer stage or genomic features like gene mutation status can be directly used to classify patients for survival analysis (reviewed in [5]). Such categorical variables that can be classified into two or more categories based on corresponding covariates, e.g., KRAS mutation vs. wild type, can be subjected to the Kaplan-Meier estimator method to produce survival curves and a logrank test can be performed to assess the significance of the difference in survival outcome between the groups [1]. As a result, categorical genomic alteration covariates including mutation status have drawn a great deal of attention as prognostic biomarkers and methods that deal with this type of data are well established (reviewed in [6–8]). However, since such genomic alterations are generally sparse in the population except for genes in core oncogenic processes such as the RAS pathway, e.g., KRAS with high mutation rate in LUAD, PAAD, and COAD [9–10], they have limited applicability and remain controversial for various reasons [11].

### Survival analysis with continuous variables

In contrast to the categorical assessment described above, many of the clinical determinants and genomic features such as age, smoking duration or gene expression represent continuous variables. The abundance of gene expression data has facilitated the validation of derived biomarkers in datasets from different sources [12–15] addressing a major challenge in the field. In addition, it seems that larger studies are needed to derive robust biomarkers, for example, to confirm whether a specific KRAS mutation might lead to a clinically relevant prognostic effect [11,16,17].

As such, there is a great interest in identifying gene expression biomarkers that show prognostic value for survival outcome. This effort will not only help uncover underlying mechanisms for the diseases of interest but also provide more reliable and feasible biomarkers for clinical application (reviewed in [18–19]).

Perhaps surprisingly, gene expression has shown great promise in producing better prognostic biomarkers than clinical factors. For example, gene expression-based classification of cancer patient samples showed a better association with survival outcome than histological classification [20–22]. However, many issues have emerged in applying gene expression-based survival analysis to clinical application. In a newly reported breast cancer study, the previously identified prognosis markers were not able to show anticipated association with survival outcome in new patient cohorts, likely due to the diverse collection of cancer patients including differences in the therapy or treatment methods between the old and new patient cohorts [23]. A similar problem was also found in another cancer study, where differential genes showed a correlation with outcome in a large dataset [24] but failed to be confirmed in a different dataset [25]. In addition to the variations in expression measurement, lack of accuracy and completeness of clinical information is a factor leading to the large variations in survival analysis results between datasets [13]. Beyond sampling and validation issues, such failures can also be partially due to the fact that many of the published studies did not follow basic statistical and/or analysis guidelines. Consequently, the choice of statistical models, as well as analysis methods and tools, are critical to derive robust biomarkers for prognosis of survival outcome [12–14,26].

### Available gene expression-based survival analysis methods and existing pitfalls

Many survival analysis methods which have been developed and applied to gene expression-based survival analysis have helped understand the outcome related underlying biology. A partial list of these methods includes gene expression cluster-based sample classification [27–29], multiple-gene decision tree-based sample classification [30], Cox regression model based semi-supervised method [31] and cancer subtype-oriented semi-supervised method [32]. Consequently, multiple genes or gene signatures have been derived as potential prognostic biomarkers. It is also very common to see gene expression subjected to survival analysis using a multivariate Cox regression model (or proportional hazards regression), which is the most widely used semiparametric survival model in the health sciences [3–4].

Using the Kaplan-Meier method and logrank test to help visually show the estimated survival functions and statistically assess the difference in survival outcome between groups respectively using derived gene expression-based prognostic biomarkers has become a popular approach. However, to our knowledge, there is no single report using them as a discovery tool to directly derive prognostic biomarkers, particularly in the case of gene expression data. Part of the reason for this is because of difficulty in classifying samples with continuous gene expression levels categorically. As a result, it is very common to cast gene expression from a continuous to a binary variable or represent it as a multi-segmented covariate to assess the prognostic impact on survival outcome. Median or quartile-based cutpoint approaches are commonly used to classify the samples into two or more groups to assess differences in their survival outcome in many publicly available survival analysis tools (reviewed in [5]). The same approach has resulted in many published reports as described below with the obvious problem that the cutpoint selection becomes critical.

Although the discovery of outcome-related biomarkers from gene expression data faces large challenges in validation, many analysis tools and databases that use the logrank test as the primary method have been made available to the medical research community. Many of these tools either claim to be or are intended to be biomarker validation tools. For example, there is a popular web-based tool (http://www.kmplot.com) that mainly focuses on Kaplan-Meier plots to compare two patient groups that are defined according to a median point (or quartile/tertile points) of expression of the selected gene [33–34]. This tool has been used to validate the association of survival outcome with genes of interest in some highly ranked publications [35–36]. Similarly, kmplot has been used for top quintile vs bottom quintile to compare the survival outcome as represented as a difference [37]. In addition, another web-based tool, PROGgene, is available for survival analysis of gene expression association with the patient outcome by bifurcating samples at the median expression of a gene of interest for multiple types of cancers [38].

However, methods that choose a limited set of predefined cutpoints fail to convey the behavior of survival association with expression along the expression gradient. Unfortunately, they also do not provide counsel to the user regarding what to do when only some of the selected cutpoints produce a significant association allowing for the possibility to distort the interpretation [6].

### An improvement—PrognoScan

A unique tool called PrognoScan is distinguished from other gene expression-based survival analysis tools in terms of how it chooses the cut-points for grouping the samples. PrognoScan also provides multiple datasets from different sources and/or different array platforms to cross-validate the results and enhance confidence. In addition, it employs a minimal *p*-value approach that helps uncover the optimal cutpoint in a continuous gene expression gradient, which is unlike other tools that use prior biological knowledge or assumptions, or fixed cutpoints to arbitrarily classify samples [39]. However, since the native use of this minimal *p*-value approach was found to be associated with a considerable inflation of the type I error rate (false positive rate [6]), a maximally selected chi-square statistics-based *p*-value correction method [40] was applied to the derived minimal *p*-value to obtain the final corrected *p*-value [39]. Thus, PrognoScan has provided a great advance compared to other methods, since it now considers the minimal *p*-values of the relevant logrank test across the entire set of all possible cutpoints along the gene expression gradient. However, in our view, since only the corrected minimal p-value is retrieved to represent the p-value for the association relation across the gradient, the valuable information regarding how the association behaves through the continuum is lost. In addition, whereas the correction of the derived minimal p-value in the PrognoScan method [39] was done by the maximally selected chi-square statistics-based p-value correction method [40], it was suggested previously that the probability of failing to detect a real association would be increased by the minimal p-value method [6].

### A new method for survival analysis—GradientScanSurv

In summary, as we will demonstrate in this report, many of the survival analysis tools and published studies that have utilized these tools may be sensitive to an analysis pitfall that can lead to an incorrect assessment of survival association of the data. With kmplot, the pitfall is that the user may be misled by fortuitously evaluating the only user-selected cutpoint that shows significance, or worse, by reporting only the point(s) that did. With PrognoScan and indeed kmplot as well, the pitfall is that with only a single readout for the association, the trend across the gradient is not made available. Our sense is that with these pitfalls, the robustness of the identified biomarkers, particularly in the context of a discovery approach has room for improvement. Therefore, we propose a novel Gradient-based Cutpoint Scan for Survival Analysis approach, we call GradientScanSurv, which extends the logrank test method from handling discrete variables to handling any continuous variable. Since our method provides a summary across the entire gradient with coupled statistical significance, we feel that this ameliorates the aforementioned pitfalls in many existing tools. The novel method appears to have better overall performance within the tested example datasets in uncovering and validating the association of gene expression with survival outcome.

## Materials and methods

### Datasets for performance comparison

Survival data and gene expression data from lung adenocarcinoma (LUAD) including microarray data were downloaded from the PrognoScan website (http://www.prognoscan.org/). Briefly, Unix curl command-based customized scripts were used to download all of the LUAD expression data and metadata for survival outcome, as well as the analysis results of the PrognoScan method from PrognoScan website for all RAS pathway genes. Customized R scripts were used to parse the downloaded data to be used for comparison. The survival data and RNAseq data from TCGA (The Cancer Genome Atlas) for selected tumor types were downloaded from the TCGA data portal (https://portal.gdc.cancer.gov/). Australian pancreatic adenocarcinoma (PAAD) cancer data was downloaded from the ICGC website (https://dcc.icgc.org/projects/PACA-AU) as AusPanc_Set dataset.

### Selected survival analysis methods for performance comparison

All RAS pathway genes have been run through customized R scripts that implemented each of the selected survival analysis methods including PrognoScan, univariate Cox Regression on gene expression, univariate Cox Regression using gene expression ranks, median cutpoint-based logrank test, and tertile cutpoint-based logrank test. The Australian pancreatic adenocarcinoma (PAAD) cancer data and TCGA data were analyzed in a similar way using customized R scripts that implemented the same methods.

The R code for the PrognoScan analysis method was obtained through email communications with the original author of PrognoScan [39], who referred to the original S-plus codes as a starting point for implementation of the functions for the PrognoScan methods using the appendix of a previously published paper [41]. The implemented functions for the PrognoScan method in R were tested on downloaded LUAD data from the PrognoScan website, and the derived results were verified to match those from the website.

Penalized linear model-based methods including Lasso and Elastic Net methods, which are Cox models with the added feature that allows them to be able to do variable selection, were implemented in customized R scripts using glmnet R package, and run on the TCGA data of different tumor types using multiple seeds (100) for multiple trials. The recurrent genes across multiple trials were collated into the aggregated lists based on a dynamic cutoff empirically determined for each tumor type with a minimal 85% percent times of all trials.

For multiple genes’ results such as results for all 225 RAS pathway genes, multiple comparisons were corrected by B-H (Benjamini & Hochberg method [42]) using the R function to obtain adjusted p-values for each of the RAS pathway genes. For a side-by-side comparison with PrognoScan, we compared selected genes on an individual basis and so no multiple test correction was applied.

### GradientScanSurv procedure

**Fig 1:**
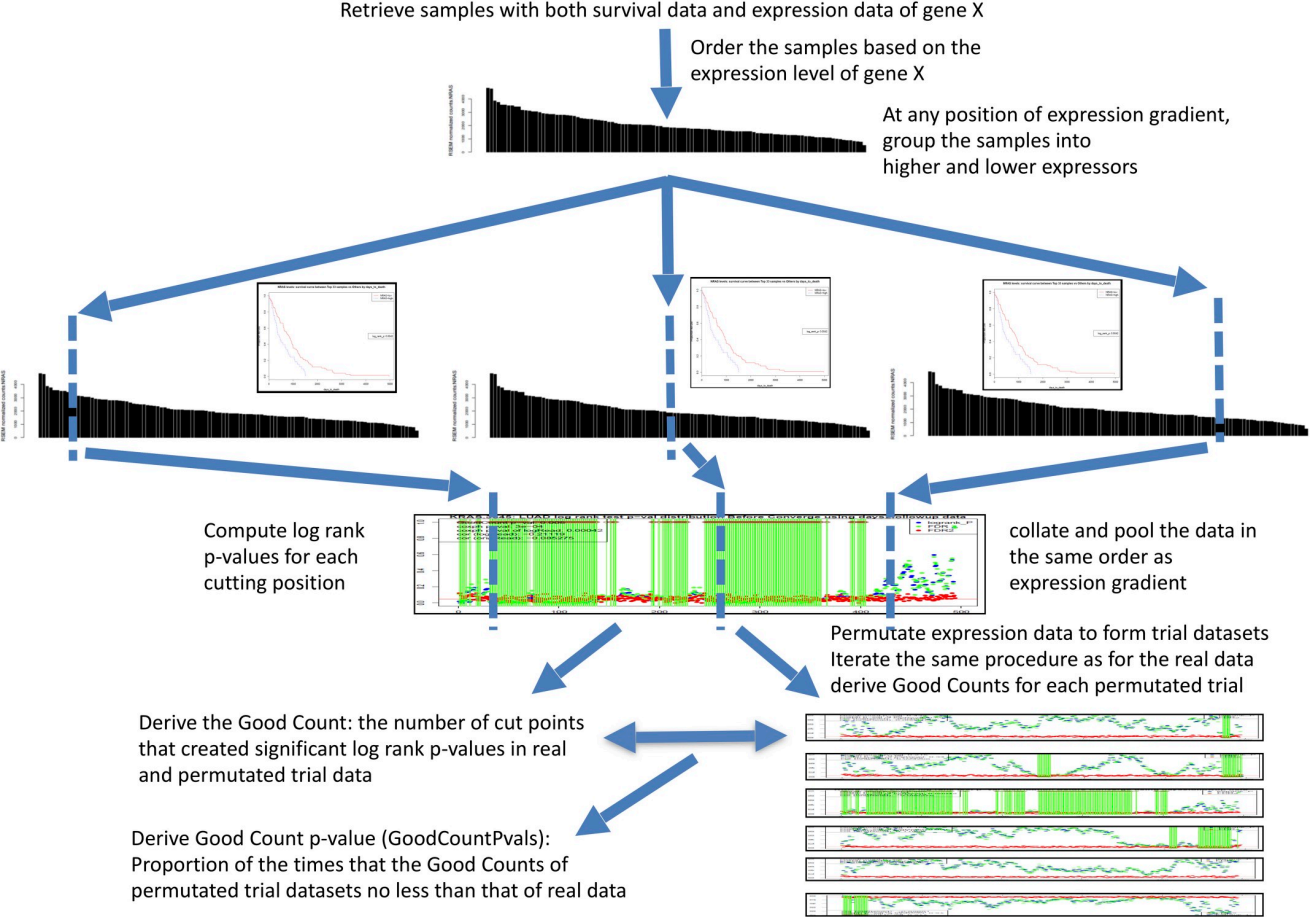
GradientScanSurv procedure for exhaustive association test of gene expression with survival outcome. Expression data was permutated to form trials of datasets (n> = 1000). GoodCounts were derived for each trial dataset and real dataset as the numbers of cutpoints that created significant logrank test p-values. From this, the GoodCount p-value (GoodCountPvals) is derived as the proportion of the times that the GoodCounts of permutated trial datasets is no less than that of real data. See Material and methods for details.

As illustrated in **Fig 1**, the GradientScanSurv method first ordered all the measured values of the input gene or biomarker across the gradient. Then at each cutpoint along the ordered gradient, a logrank test was used to derive a p-value to assess the difference of survival outcome between the two groups split by the corresponding cutpoint. These p-values are collected, assessed, and counted to obtain a GoodCount for the real data. GoodCount was defined as numbers of cutpoints where the corresponding separated two groups had significant p-value (p< = 0.05 as the default setting) for their logrank tests.

Suppose we observe n observations with the vector of survival outcome **y**_***n*×1**_ and censoring indicator **δ**_***n*×1**_. For gene expression j, we observe *n* subjects with gene expression value **x**_***j***_ = (x_1*j*_, *x*_2*j*_,…,*x*_*nj*_)^*T*^. Our approach first calculates the logrank test at each possible cutting point of the ordered gene expression values **x**_***j***_. To be specific, denote the ordered gene expression values as ${\tilde{x}}_{j}={\left(x_{(1)j},\;x_{(2)j},\ldots ,x_{(n)j}\right)}^{T}$, and let the correspondingly ordered outcome and censoring indicator as $\tilde{y}$ and $\tilde{\delta }$, we then test, for all possible cutpoints, the logrank test statistics and obtain the p-value.

$$logrank\;pvalue({\tilde{x}}_{j}\leq x_{(k)j},\tilde{y},\tilde{\delta })$$

This is essentially treating the binary indicator ${\tilde{x}}_{j}\leq x_{(k)j}$ as the group label in the logrank test.

Note that these logrank test p-values at each cutpoint do not directly provide grounds for significance for the overall association relation, but they do form the basis for the next level of aggregate statistics described below as GoodCount and GoodCount p-value that are indicative of a significance level of the overall association relationship. In our typical graph view, our method provides green lines as tentative significant cutpoints along the gradient, which provides not only the basic metrics for the next level of aggregate statistics but also the visual cue for the likelihood of the significance for the overall association (number and positions of the lines along the gradient).

A GoodCount is then defined as the number of significant tests (using ***α*** as the significance level, the default setting of 0.05) across all possible cutpoints:

$$GoodCount(x_{j},y,\delta )=\sum_{k=1}^{n-1}I\{logrank\;pvalue({\tilde{x}}_{j}\leq x_{(k)j},\tilde{y},\tilde{\delta })<\alpha \}$$

In order to obtain the p-value of this count statistic, we employ the bootstrap [43] approach: we first randomly permute the **x**_***j***_ values into $x_{j}^{*}$ by reshuffling. Then a new GoodCount value based on this permuted data is obtained: $\theta =GoodCount(x_{j}^{*},y,\delta )$. This procedure is then repeated M times and obtain the corresponding M GoodCount values {θ_1_,…θ_M_}. The bootstrapped p-value is obtained by checking how many of the permuted GoodCount values are larger than the original GoodCount value, i.e.,

$$GoodCountPval=\frac{1}{M}\sum_{m=1}^{M}I\{GoodCount(x_{j},y,\delta )\geq \theta_{m}\}$$

The GoodCount p-value (GoodCountPval) was defined as the proportion of the times that the GoodCounts of permutated datasets were no less than that of the original dataset. By comparing the GoodCount values from real data and those from permutated data, which work against each other and respond to the alpha level simultaneously, the derived GoodCount p-value is the final deterministic statistic for whether there is a significance of the association. Explicit derivation of the theoretical p-value under this situation could be difficult. Hence, we choose to use the robust bootstrapping method, to derive the GoodCount p-value for an unknown distribution.

Practically, to create the permutated datasets, the original survival data and expression data were permutated for at least M> = 1000 times by shuffling the expression data between sample names. The GoodCountPval is the key statistics produced by the GradientScanSurv method that is used to assess the significance of the association of the individual gene expression or biomarker with the survival outcome.

### ROC analysis procedure

ROC analysis was performed on the results of each survival analysis method under evaluation (see above) using independently derived datasets of the same disease from different sources as the Training Set and Testing Set respectively. Alternatively, shared positive gene lists from at least two of the methods from the Training Set were used as Truth lists for each method in the Testing Set data. To test whether the selection of which dataset was used as the Training Set and which was used as the Testing Set had an impact on the results, the datasets used for Training Set or Testing Set were switched so that any potential bias imposed would be tested and evaluated in our comparison. Specifically, PrognoScan LUAD data and TCGA LUAD data were downloaded and have been used separately as either the Training Set or the Testing Set in the ROC analysis respectively. Alternatively, the TCGA LUAD data was used as the Training Set and the PrognoScan LUAD data was used as the Testing Set. Thus, our aggregate results are composed of multiple datasets of PrognoScan LUAD data and multiple trials of analysis results using the TCGA LUAD dataset.

To make sure the results of the GradientScanSurv method were reliable and robust, multiple trials (100) of analysis results for GradientScanSurv method were obtained. Since other methods do not have a permutation test, their results for an input data set remain constant. Our comparison applied the results from multiple trials of GradientScanSurv with results from other methods analyzing the same TCGA data as described. There are also multiple PrognoScan datasets (total of 9) from various microarray platforms and derived from different studies. Each of these datasets was analyzed by all methods to obtain corresponding results, which were combined as the PrognoScan results of multiple trials.

Ultimately, the ROC analysis was then used to assess the performance of each method between these Training Sets and Testing Sets of multiple trials to derive the overall performance statistics for each method i.e., percentage of the total times that each of these selected methods being compared had the largest AUCs compared to other methods in each of the combined trials. In addition, the average AUCs were derived for comparison between the selected methods for each subset of total trials of data defined by each of the 9 PrognoScan datasets in combination with the 100 TCGA trials of data, simply because the PrognoScan datasets are much more diverse in platforms and data sources compared to trials of the same TCGA data.

The TCGA and PrognoScan data are independent datasets from different sources but derived from the same disease (LUAD, lung adenocarcinoma). The corresponding Training and Testing Sets were derived for a total of 225 RAS pathway genes for each method for each trial of the analysis. The analysis results of each trial are essentially adjusted p-values of each gene derived from each method that were subjected to multiple test correction by the Benjamini & Hochberg method [42].

The ROC analysis used to compare methods was based on the assumption that when we used independent datasets of the same disease for ROC analysis with one as the Training Set and the other as the Testing Set (e.g., TCGA or PrognoScan downloaded LUAD data respectively), if the method is better, we would get better AUCs in the ROC analysis, since we would expect more consistent results in the classification of Ras pathway genes either associated or not associated with survival outcome. We did the analysis this way since there is no valid truth set of survival data available for our purpose that reports which genes are truly associated with survival outcome for this particular disease.

By design, the GradientScanSurv method uses a permutation (bootstrap) method to derive the GoodCount *p*-value. Permutations naturally caused some variation in results from trials to trials. However, the method is not impacted by those fluctuations. The default setting for the number of permutations was 1000 that was identified as a practical level from our initial tests with many datasets that were observed to have relatively stable results.

Although functions of the pROC R package were used as the main procedure for ROC analysis and plotting, customized R scripts were used to collect the statistics from pROC analysis on multiple trials of both Training and Testing Sets for the purpose of performance comparison of the selected survival analysis methods.

## Results

### Examples of common pitfalls in web-accessible survival analysis tools and the biological studies that used them

We began by characterizing the pitfalls associated with methods that offer a limited set of predefined cutpoints that split the data into categories for subsequent survival association assessment. We observed several common pitfalls in many publicly available survival analysis web tools that are used for putative biomarker validation or analysis of the association between survival and gene expression data. These tools have been previously described [5] and have been used by researchers in the community with the analysis results presented in publications. Because of the frequency and potential impact of such cases, we feel it is important to bring their pitfalls to the attention of the community in more detail.

We detail two examples chosen as representative of the issues we identified that appeared in highly ranked journals [35–36], although many additional examples can be found (see **Table A in S1 File**). In no way does our selection reflect or judge those manuscripts other than to use them as examples of the potential pitfalls associated with these tools. In the original Fig 3B in [36] of the first study, the authors reported survival analysis results for stage II lung cancer patients with high and low KIAA1522 expression using the Kaplan-Meier Plotter website (kmplot.com, [5]). Their figure legend and main text of the first study [36] mentions only that they performed a survival analysis between KIAA1522 high expression and low expression patients, but did not provide any further details about how the samples were grouped into high and low expression categories. These details were also not provided in the methods section. To dig into the details, we went to the Kaplan-Meier Plotter website and used the KIAA1522 gene as input to run the analysis. For accuracy purpose, according to their figure legend of the first study [36], we selected only stage II lung cancer patients for the analysis. Fortunately, the KIAA1522 gene has only one Affymetrix probeset, 224746_at, for the database of lung cancer. The website provides 5 options to “Split patients by” - 5 possible cutpoints: lower quartile, lower tertile, median, higher tertile, and higher quartile for the logrank test analysis. We obtained the analysis results for all of the 5 options as shown in **Figure A in S1 File**. Interestingly, we found the result of one option (lower quartile) gave a very similar result to that reported in the original Fig 3B in [36] of the first study that was significant. However, all 4 of the other options gave non-significant results (**Figure A in S1 File**). Thus, it appears that the authors reported the only positive result of the 5 options and made no reference to the other possible outcomes. We are by no means condemning their work as they simply used the tool as advertised. However, this is a perfect example of how a user can become the victim of unintended consequences through inadvertent or otherwise selective parameter choices. This suggests the need for a more unbiased method for parameter selection in these survival association tools.

In the original Fig 6E in [35] of the second study, the result of Kaplan-Meier Plotter website (kmplot.com) on the RELN gene for lung adenocarcinoma (LUAD) is reported. In this study [35], the figure legend clearly specified that the median-based cutpoint was selected for the analysis. We wanted to explore the behavior of survival outcome differences using all possible cutpoints along the expression gradient. We applied our method (details below) to the same microarray dataset (the merged microarray data for LUAD was kindly provided by the authors of the Kaplan-Meier Plotter website) to analyze the association of RELN gene expression with survival. In fact, this dataset showed consistent significant logrank p-values at the cutpoints that split the samples across almost entire expression gradient (**Figure B in S1 File**). In contrast to the first study [36] where only a single option produced significance, here choosing any cutpoint around the median, quartile, and tertile cutpoints that were given as options on the Kaplan-Meier Plotter website showed similarly significant logrank test results.

To test the robustness of this result, we ran our method (see below) on the RELN gene using downloaded TCGA LUAD RNAseq data (**Figure C in S1 File**). Here, the logrank p-value is non-significant at either the median point or all of the quartile or tertile points, in contrast to the result using microarray data [35]. Thus, one can imagine that if the Kaplan-Meier Plotter website ran their analysis of the TCGA RNAseq data, there would not be any cutpoints from their available options that show a significant logrank test p-value, even though there are a lot of such cutpoints available that have significant p-values (vertical green lines on the **Figure C in S1 File**). Of course, consistency across different platforms would be desired of any useful biomarker and so this observation is worrisome if RELN indeed is associated with lung cancer survival.

More examples of common pitfalls of survival analysis in publicly available survival analysis web tools and biological studies that have used these tools are listed in **Table A in S1 File**. In summary, these observations suggested that using a limited set of predefined cutpoints such as median, quartile, tertile points overly simplifies the assessment of whether gene expression is associated with survival outcome. Further, the use of a small number of predefined cutpoints offered by the tools permits a user to either through luck or design to report only the particular cutpoint(s) that produces the desired result. This led us to propose a novel approach, termed GradientScanSurv, to assess and visually present the gene expression and survival relationships in a more comprehensive way by considering all the cutpoints along the expression gradient.

As a quick proof of concept, with respect to the RELN gene example just described, we do observe a significant GoodCountPval as 0.0405 using GradientScanSurv to analyze the TCGA RNAseq data (shown at the top left corner in **Figure C in S1 File**), indicative of the potential association of this gene with survival outcome (see below). This result is consistent with the microarray data result and would have been missed if using the methods of Kaplan-Meier Plotter website (kmplot.com) to analyze the TCGA RNAseq data.

### Introduction and overview of the GradientScanSurv method

Our novel method is designed to address the shortcomings encountered by the existing methods for analysis of the association between gene expression and survival outcome discussed above. We hypothesized that if there is a significant association of gene expression with survival outcome, there would be a significant number of cutpoints along the expression gradient that should show significant differences in survival outcome when compared with random chance. Given the possible variations in sample handling, tumor heterogeneity, gene expression measurement, or metadata collection, it seems unlikely that even if there exists an association between the gene expression and survival outcome, each cutpoint applied to split the samples into high and low expression groups, would result in a significant difference in survival outcome assessed by the logrank test method. However, it is reasonable to assume that a significant number of cutpoints with tentative significance in corresponding logrank tests would be more than expected by random chance.

This is the major hypothesis that fueled our motivation to develop the GradientScanSurv method (**Fig 1**; also see the Materials and methods section for the formal mathematical description of the procedure). It has been long recognized that more pre-specified cutpoints are recommended [6]. However, only our method exhaustively evaluates all of the cutpoints along the expression gradient and then aggregates their statistics using permutation. We feel this addresses the issues of all current approaches that have the pitfall of choosing from the predefined limited set of cutpoints. Our method is inspired by the Random Forest method [44], which is one of the most accurate machine learning methods to date. The rule at each splitting step of the tree construction is that random forest will search all cutting points exhaustively, and choose the best one. The advantage of such a procedure is that the method will be able to handle many different types of underlying model effects such as non-monotone effects, or even symmetric structures. As pointed out previously [45], the best cutting point is actually the point where the underlying function has the steepest slope, making the left and right groups the most distinct. Hence our GradientScanSurv method that considers all cutting points exhaustively but resolves into an aggregate statistic instead of taking the best one as the solution as implemented by the PrognoScan method (see below in the Side-by-side comparison with the PrognoScan method section) will be also able to benefit from similar advantages.

In addition, we introduce the GoodCount p-value (GoodCountPvals) metric as the key statistical assessment of whether there is a significant number of cutpoints along the expression gradient that shows a significant difference in survival outcome compared with a random chance that was assessed through expression data permutation-based trials of datasets (**Fig 1**). In addition, the graphical display of the GradientScanSurv results provides additional insights regarding the extent and direction of the association that facilitates the assessment of association significance (**Figure D in S1 File**). Most, if not all of the publicly available survival analysis web tools only provide directionality for the overall relationship between survival outcome and expression change direction (e.g., higher gene expression is associated with faster death) at the predetermined points by Kaplan-Meier plots. Our tool also provides visual cues including vertical green lines across the expression gradient for cutpoints with tentatively significant p-values from logrank tests and brown diamonds at either the top or at the bottom for directionality of the gene expression changes versus survival outcome. This provides a visual measure of the consistency of the direction across the gradient as well.

Many of the genomic feature-based biomarkers, especially transcript-based biomarkers discovered in survival analysis, lack of reproducibility and robustness when applied to different datasets. This represents the major technical obstacle in the clinical application of genome-based biomarkers [14]. One possible reason is that when only one or a few cutpoints are used to classify the samples, because of the limited view of the data, there are more difficulties later in the validation process. In contrast, the systematic and aggregate statistical scheme in our GradientScanSurv method can potentially remove this possible source of inconsistency, thereby providing higher confidence and statistical power in validation results.

### Side-by-side comparison with the PrognoScan method

As described in the introduction, the PrognoScan method addresses the selection of cutpoints from a predefined list by evaluating all possible cutpoints and returning a corrected p-value for the optimal cutpoint. In order to directly compare our method with the PrognoScan method, we ran the PrognoScan analysis on its website (www.abren.net/PrognoScan/) for genes from the RAS pathway such as NRAS, KRAS, and MAP2K1genes for Lung adenocarcinoma (LUAD) data. We then downloaded the raw data from the PrognoScan website for these genes and then ran our GradientScanSurv method so that we could compare the results side by side with those produced by the PrognoScan method. Interestingly, PrognoScan has multiple datasets for the same disease for the same genes, which allowed us to assess how consistent the results were for each method.

We chose to use LUAD data since LUAD is a subtype of lung cancer that has been clearly defined, whereas the other types of cancer data in PrognoScan such as COAD and OV have no clearly defined subtypes. Using this clearly defined subtype for LUAD data should increase the chance of validation and consistency in results between the multiple datasets. Of course, one of the difficulties in substantiating any survival association method is the lack of a definitive truth set. Therefore, we are forced to base our selections on known genes or pathways implicated in cancer for validation purposes.

**Fig 2:**
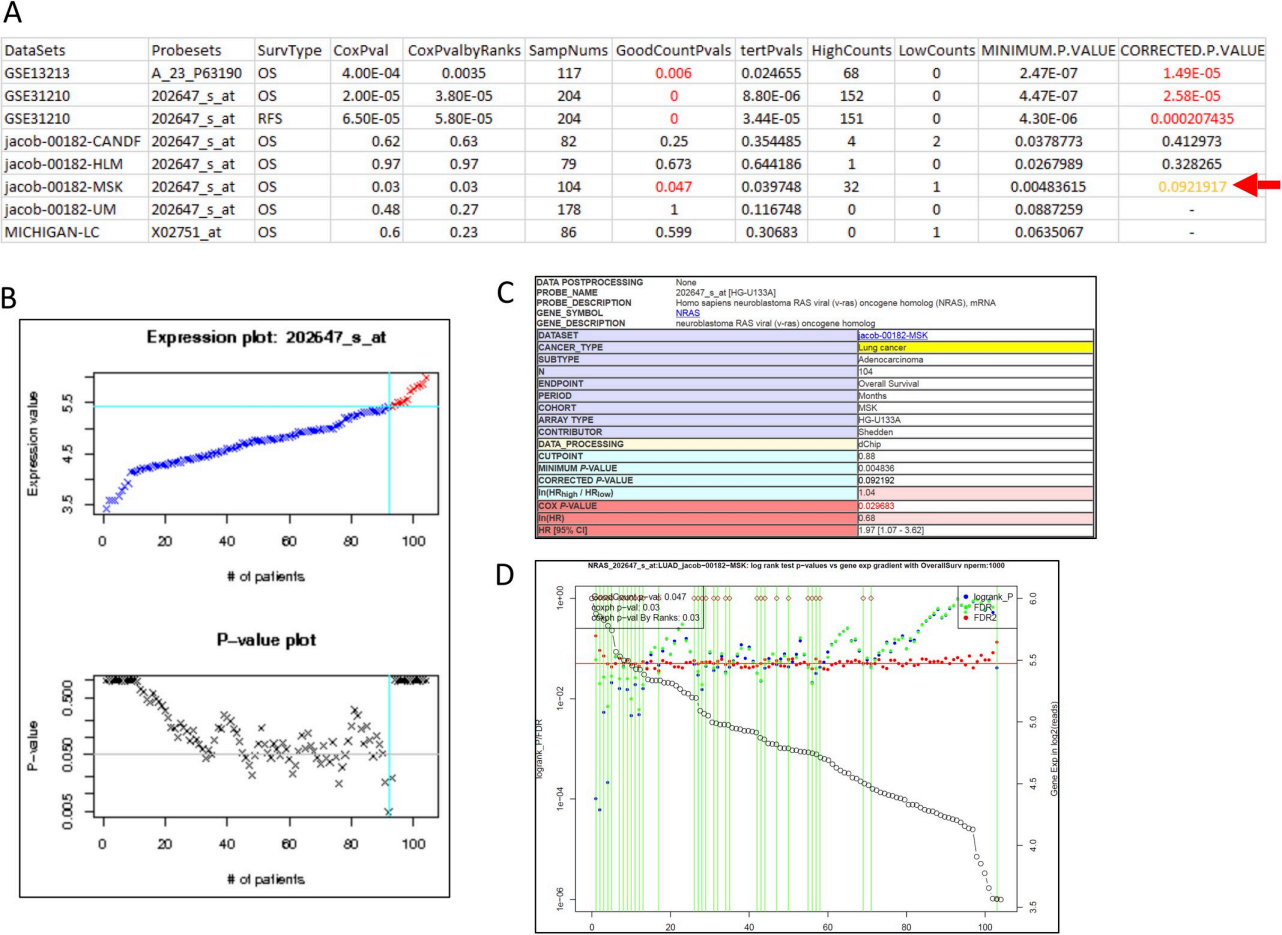
Comparison of GradientScanSurv with PrognoScan results for NRAS gene in LUAD datasets downloaded from PrognoScan website. (A). A joined table of GradientScanSurv results and direct results from the PrognoScan website for the NRAS gene in LUAD (lung adenocarcinoma) datasets from the PrognoScan website. The red arrow indicates a dataset where GradientScanSurv called a significant association with GoodCountPval = 0.047, but PrognoScan missed the call with CORRECTED.P.VALUE = 0.092. (B). Screenshot of PrognoScan’s expression gradient-based logrank p-values plot. The blue vertical line indicates where the minimal p-value is that was used for the final CORRECTED.P.VALUE. (C). Screenshot of the PrognoScan report with final CORRECTED.P.VALUE at 0.092. (D). GradientScanSurv gene expression gradient-based logrank p-values plot with GoodCountPval at 0.047. The green vertical lines indicate specific cut-points where the corresponding logrank p-values are significant at p-value< = 0.05. Univariant expression-based coxph p-value and expression-rank based coxph p-value are also reported here as in panel A. This result was consistent with the TCGA validation result in GoodCountPval (0.001). The plot of the results also illustrates that higher expression of NRAS is associated with faster death, which is indicated by the brown diamonds along the top part of the plots (Figure E in S1 File).

KRAS and NRAS mutations are known to be critical oncogenesis driver genes mutated in LUAD, although NRAS mutations are relatively rare [46]. NRAS expression has been shown to correlate with KRAS mutation status in LUAD samples from TCGA [47]. Therefore, its expression could be associated with survival outcome. As expected, we found that 4 out of 8 datasets of NRAS (with different probesets for the NRAS gene and from different sources) showed significant GoodCountPvals (p<0.05), whereas only 3 out of 8 datasets showed significant for PrognoScan results (**Fig 2A**). This is confirmed by the GradientScanSurv method with a very significant GoodCountPvals (= 0.001 with permutation n = 1000) (**Figure E in S1 File**) using the TCGA LUAD dataset with RNAseq data for expression values. This shows that even extending to data derived from a different platform (RNAseq), our method shows better consistency compared with the microarray platform for PrognoScan data. Given the sample size limitation of these datasets, platform differences, data source differences, the relatively high proportion of the multiple datasets showing consistent positive results we observed in our validation is very reasonable and of relatively high confidence.

For the dataset indicated by the red arrow in **Fig 2A**, where the GradientScanSurv method obtained a significant p-value that the PrognoScan method missed, the GradientScanSurv method accumulates the significant logrank test p-values that are scattered along the gradient making the GoodCountPvals significant (**Fig 2D**), whereas the PrognoScan method did not attain significance because the minimal p-value is of insufficient magnitude (**Fig 2B**) in spite of widespread cutpoints along the expression gradient showing significant p-values. This confirms the importance of aggregating statistics across all cutpoints along the expression gradient in the GradientScanSurv method rather than just using predefined cutpoints (even the minimal p-value cutpoint from PrognoScan) used by other methods.

We also compared another important RAS pathway gene, MAP2K1, which is downstream of RAS and may also be expected to be associated with survival outcome. We observed 3 out of 11 datasets that showed a significant association with survival outcome for MAP2K1 by the GradientScanSurv method (**Figures F and G in S1 File**), whereas only 1 out of 11 datasets showed a significant association with the PrognoScan method (**Figures F and G in S1 File**). This is also confirmed by the GradientScanSurv method with a significant GoodCountPvals (0.031 with permutation n = 1000) (**Figure H in S1 File**) with the TCGA LUAD dataset using the RNAseq platform for expression data. For the dataset indicated by arrows on **Figure F, panel A and Figure G, panel A in S1 File**, the GradientScanSurv method obtained a significant p-value whereas the PrognoScan method did not.

**Fig 3:**
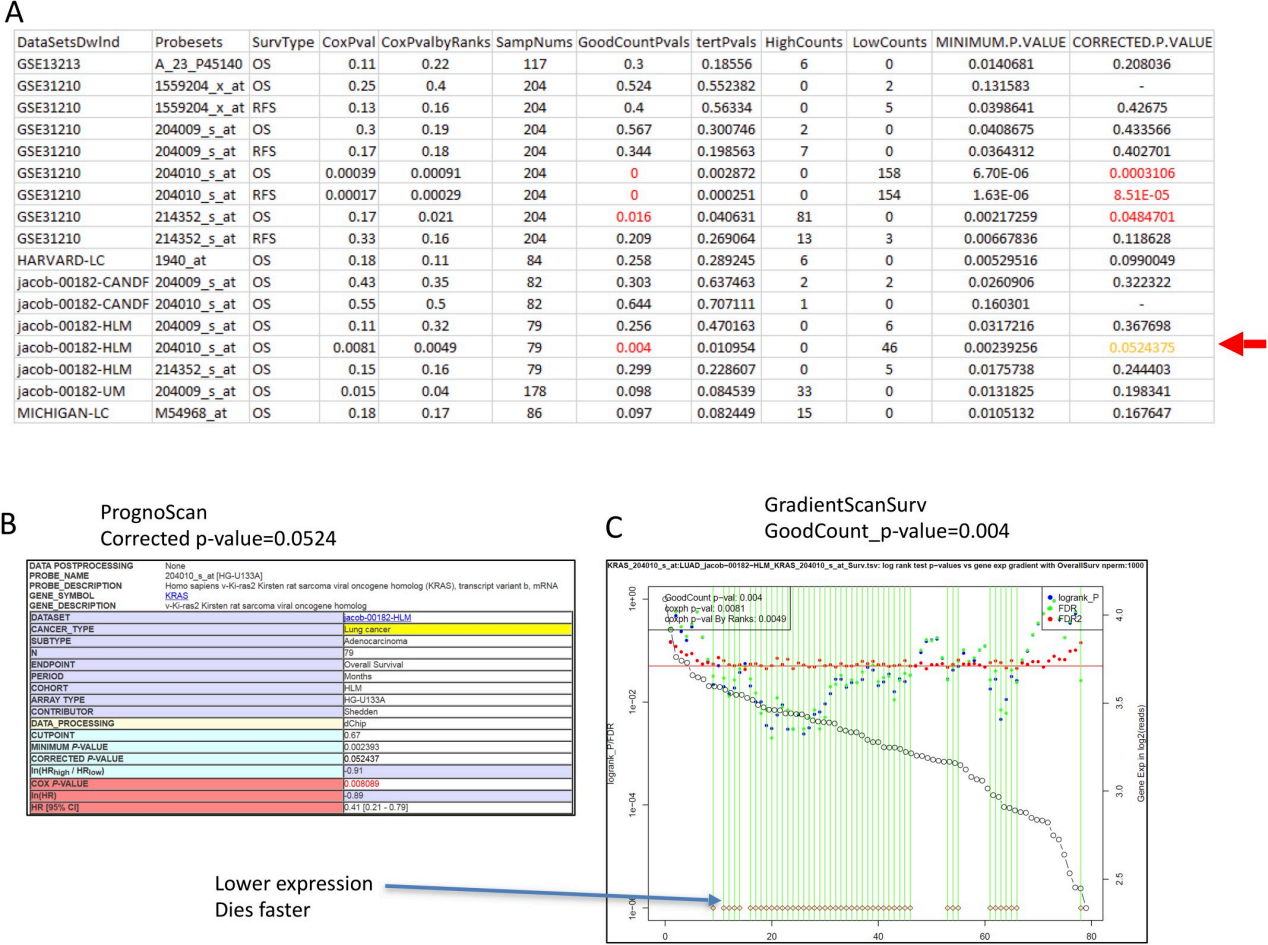
Comparison of GradientScanSurv with PrognoScan results for the KRAS gene in LUAD datasets downloaded from PrognoScan. (A). A joined table of GradientScanSurv results and direct results from the PrognoScan website for the KRAS gene in the same LUAD datasets. The red arrow indicates a dataset where GradientScanSurv called a significant association with GoodCountPval = 0.004, but PrognoScan barely missed the call with CORRECTED.P.VALUE = 0.0524 for dataset Jacob-00182-HLM with probeset 204010_s_at. (B). Screenshot of PrognoScan’s report for the dataset indicated by the red arrow in panel A. (C). GradientScanSurv gene expression gradient-based logrank p-values plot with GoodCountPval at 0.004 for the dataset indicated by the red arrow in panel A. See text for details.

At the mutation level, KRAS is believed to be one of the most critical oncogenic genes in LUAD. When we examined the association between KRAS expression and survival, we found that 4 of 17 datasets showed a significant association with survival outcome using our GradientScanSurv method (**Fig 3**). Conversely, only 3 out of 17 datasets showed a significant association with the PrognoScan method (**Fig 3**). This is also confirmed with the GradientScanSurv method by a significant GoodCountPvals (= 0.004 with permutation n = 1000) (**Figure I in S1 File**) using the TCGA LUAD dataset with the RNAseq platform for expression data. In addition, the cue from the GradientScanSurv plot shows that higher expression of the KRAS gene is associated with faster death (**Figure I in S1 File**), which is expected from the known RAS biology in LUAD cancer study [47].

Looking at different probes, however, the GradientScanSurv result using microarray data from PrognoScan showed that lower expression measured by KRAS probeset 204010_s_at is associated with faster death (**Fig 3C**). This is further supported by the GradientScanSurv result with two additional KRAS datasets measured by the same probeset 204010_s_at (**Figures J and K in S1 File**). In contrast, another dataset using a different probeset 214352_s_at that also measures KRAS gene expression showed a consistent result with TCGA result: higher expression of KRAS gene is associated with faster death (**Figure L in S1 File**). Interestingly, a dataset that used another probeset (204009_s_at) for the KRAS gene also showed the same direction at the cutpoints with significant logrank test p-values (**Figure M, panel C in S1 File**), where higher expression of KRAS gene is associated with faster death, even if the dataset overall did not show significant GoodCountPvals.

To resolve this seemingly contradictory observation for the different probesets of the KRAS gene, we used UCSC Genome Browser (https://genome.ucsc.edu/cgi-bin/hgTracks) to visualize their genomic locations within the KRAS locus that was provided by Affymetrix (http://www.affymetrix.com/analysis/index.affx). Interestingly, the two probesets that showed consistent results with TCGA result (204009_s_at and 214352_s_at) aligned with the last exon in KRAS (**Figure N in S1 File**), and they correlate very well with each other in the expression data of these datasets from PrognoScan (**Figure O in S1 File**). In contrast, the probeset 204010_s_at that showed the opposite direction with the TCGA result indeed is at the far end and off the last exon of the KRAS gene (**Figure N in S1 File**). In addition, this probeset correlated to a much lower extent with the other two probesets (**Figure O in S1 File**). It is very likely that the last probeset may not really measure the KRAS gene expression or is interfered with by a transcript from the gene on the other side of the KRAS locus. Thus, its directionality of survival change may be opposite to that of the real KRAS gene. Therefore, all of these GradientScanSurv results are consistent in their significance as well as in their change directions. This suggests that the ability to visualize the direction of the survival change by the GradientScanSurv method is valuable and useful in further validation of the analysis results. This allows for consistency of the survival status direction along the expression gradient to be visualized, a feature absent from all other methods that focus on predefined or limited cutpoints.

### Global and systematic performance comparison with other survival analysis methods on univariate gene expression

#### Approach 1: ROC analysis using recurrent genes as truth lists

The examples above demonstrate that on a limited basis, our new method appears to outperform PrognoScan with the tested datasets, but we wanted to perform a more systematic assessment of performance across multiple available methods. In order to test whether the GradientScanSurv method performs better than other survival analysis methods using univariate expression of each gene, receiver operating characteristic (ROC) analysis was performed on the same datasets for several commonly used methods in the field including PrognoScan, univariate Cox Regression on gene expression levels, univariate Cox Regression on ranks of gene expression, median cutpoint-based logrank test, and tertile cutpoint-based (top quantile vs bottom quantile) logrank tests. The last two methods are typical methods commonly used by Kaplan-Meier Plotter website (kmplot.com). Univariate Cox regression is also a commonly used method along with the Kaplan-Meier estimator and logrank test. Rank-based Cox regression is an alternative way to perform univariate Cox regression on gene expression but using the ranks of gene expression for regression instead of the absolute expression levels.

The available survival data and associated gene expression data are derived from diverse platforms and vary in sample sizes, which made the choice of validation survival datasets even harder. We decided to use independent survival datasets for the same disease to increase the robustness of result. We chose to use both TCGA LUAD data and LUAD data downloaded from the PrognoScan website. Due to the concerns with the impact of the sample size on survival analysis, instead of the common k-fold cross-validation scheme, we decided to use either the TCGA LUAD data or the downloaded LUAD data from the PrognoScan website as the Training Set, and then used the other data as the Testing Set for the ROC analysis. Since there are many PrognoScan LUAD datasets and the GradientScanSurv method uses permutations to derive p-values, we combined these different trials of TCGA results from the GradientScanSurv method and PrognoScan LUAD datasets into multiple trials of data (see the “ROC analysis procedure” in the Materials and methods section for more details). In addition, we focused only on genes from the RAS pathway (10, 46), which presumably play major roles in a variety of cancer types. Thus, these genes are more likely to represent biomarker genes that are associated with cancer patient survival outcome than randomly selected genes that may accidentally be called as positive hits [26].

**Fig 4:**
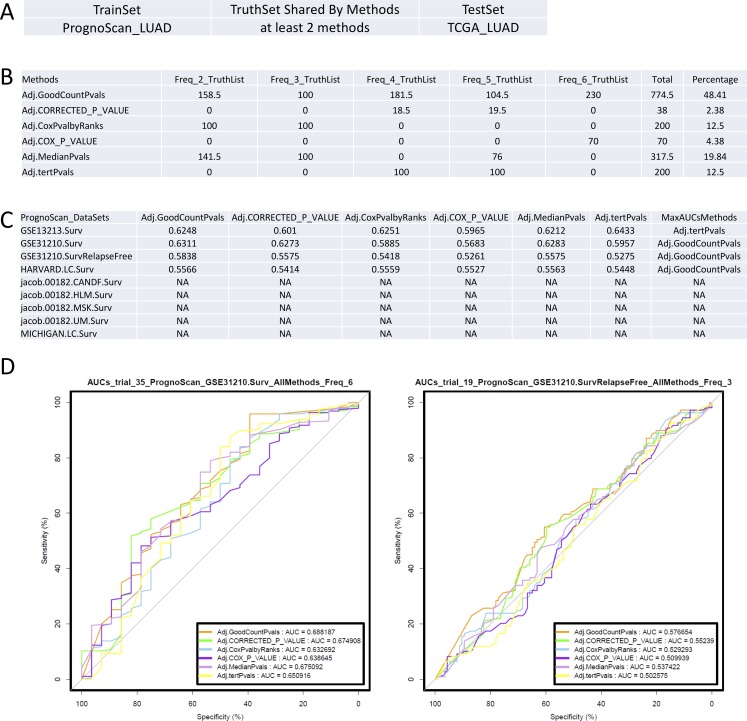
Performance comparison with ROC analysis by shared truth lists. (A). ROC analysis comparing all listed methods for the same set of truth gene lists that were shared amongst multiple truth lists derived from individual methods using PrognoScan LUAD data as the Training Set and TCGA LUAD data as the Testing Set. The settings table shows which datasets were used for the Training Sets and Testing Sets, as well as whether the Truth Set of genes derived from training are shared lists from at least 2 listed methods. The general ROC analysis procedure was described in the Materials and methods section. (B). Performance comparison table listing numbers of times/trials that a method has the largest AUC of ROC curves compared to other methods for the same set of truth gene lists (shared by at least 2 listed methods) and same trial of Testing Set dataset. All truth lists from Training Sets and the result lists derived from Testing Sets are at the adjusted p-value< = 0.05 for each method. The Freq_2_TruthList consists of the truth gene list shared by at least 2 of these selected methods; Freq_3_TruthList consists of the truth gene list shared by at least 3 of the selected methods etc.. Column “Total” summarizes the total counts for all scenarios (if a tie, each method would get 0.5 counts). Percentage lists the proportions of those counts. The details of each trial are shown in **Table C in S1 File**. (C). Average AUCs of ROC analysis for each method in each subset of total trials of data defined by 100 trials of TCGA data combined with one of the indicated PrognoScan datasets in column “PrognoScan_Datasets” (also see Materials and methods section for details). The last column”MaxAUCsMethods” shows the method with the maximal AUC for each row. (D). Examples of ROC plots showing AUCs of GradientScanSurv method are the largest in these trials.

We evaluated the performance of different methods using genes shared between methods as a Truth Set and also using genes identified by each individual method. In the first case, ROC analysis was first performed using the consensus gene lists identified and shared between the selected methods with datasets of Training Sets for validation by each of these individual methods in datasets of Testing Sets. Briefly, the PrognoScan LUAD data was first used as a Training Set to create a series of Truth Sets defined by being shared between the comparable lists produced by the different methods at different frequencies (See legend of **Fig 4** for details). Then, ROC analysis was performed on the TCGA LUAD data as the Testing Set (**Fig 4A**). Within the multiple trials of test data that were produced, the GradientScanSurv method has the largest percentage (48.41%) of total trials with the largest AUCs compared to other methods (**Fig 4B**). In addition, the GradientScanSurv method has the largest average AUCs compared to other methods in 3 out of 4 subsets of trials of data defined by 100 trials of TCGA data combined with one of the PrognoScan datasets, which have valid AUCs in ROC analysis (**Fig 4C**). It should be noted that the results are very different from trial to trial (**Tables B and C in S1 File**).

In order to test whether the selection of which dataset was used as the Training Set and which was used as the Testing Set impacted the results, the TCGA LUAD data was first used as the Training Set to create a series of Truth sets as shared Truth lists at different frequencies of positive lists derived from each of selected methods. As before, ROC analysis was then performed on the PrognoScan LUAD data as the Testing Set (**Figure P, panel A in S1 File**). In spite of smaller margins, the GradientScanSurv method still had the largest percentage (20.32%) of total trials with the largest AUCs compared to other methods (**Figure P, panel B in S1 File**). In addition, the GradientScanSurv method has the largest average AUCs compared to other methods in 4 out of 9 subsets of trials of data in combination with one of the PrognoScan datasets, which is more than any other methods (**Figure P, panel C in S1 File**). We used the Truth sets that were shared Truth lists from two or more of these methods to increase the reliability of the input Truth lists. Again, the GradientScanSurv method consistently performed the best in spite of variations between the datasets.

#### Approach 2: ROC analysis using each method to derive truth lists

**Fig 5:**
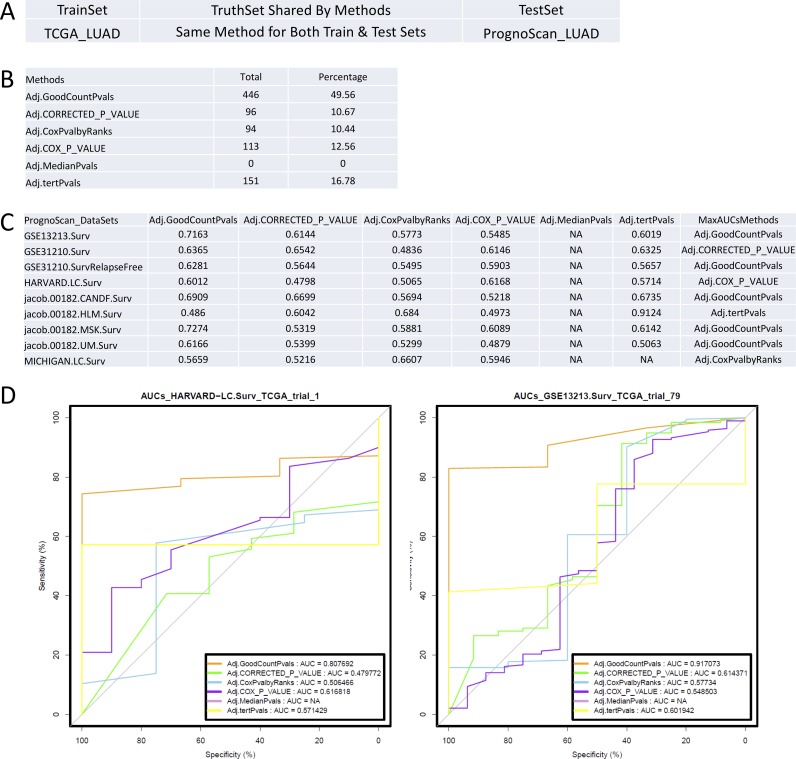
Performance comparison by ROC analysis with the same set of Training Set and Testing Set for the same method. (A). ROC analysis comparing all listed methods, each of which has been applied to the same set of Training Set and Testing Set for the same method. The settings table shows which datasets were used for the Training Set and Testing Set. The same method has been applied for the same set of TCGA LUAD data as Training Set and PrognoScan LUAD data as Testing Set. (B). Performance comparison table listing numbers of times/trials that a method has the largest AUCs of the ROC curves compared to other methods for the same set of Training Set and Testing Set datasets are shown in panel A. Column “Total” summarizes the total counts of all of the best trials of same Training Set and Testing Set datasets for each method listed in column Methods. Column “Percentage” lists the proportions of those counts. All truth lists derived from the Training Set and the result lists derived from Testing Set are at adjusted p-value< = 0.05 for each method. The details of each trial are shown in **Table E in S1 File**. (C). Average AUCs of ROC analysis for each method in each subset of total trials of data defined by 100 trials of TCGA data combined with one of the indicated PrognoScan datasets in column “PrognoScan_Datasets” (also see Materials and methods section for details). The last column”MaxAUCsMethods” shows the methods with the maximal AUC for each row. (D). Examples of ROC plots showing that the AUCs of the GradientScanSurv method are the largest in these trials. AUC = NA is due to the fact that the MedianPvals method has no positive calls in Training datasets at adjusted p-value< = 0.05.

In order to directly test which of the methods made more consistent calls with different datasets of the same disease, ROC analysis was performed using the individual gene lists identified by each of the selected methods with datasets of Training Sets for validation by each of these individual methods in datasets of the Testing Sets. Either TCGA LUAD data was used as the Training Set and PrognoScan LUAD data as the Testing Set, or vice versa. For the first scenario where TCGA LUAD data was used as the Training Set and the PrognoScan LUAD data as the Testing Set (**Fig 5A**), the GradientScanSurv method has the largest percentage (49.56%) of total trials with the largest AUCs compared to other methods (**Fig 5B**). In addition, the GradientScanSurv method has the largest average AUCs compared to other methods in 5 out of 9 subsets of trials of data defined by 100 trials of TCGA data combined with one of the PrognoScan datasets (**Fig 5C**). In this particular analysis, the Median cutpoint-based logrank test showed the worst performance with no single positive calls in the Training Set identified in contrast to all other methods (**Tables D and E in S1 File**).

For the second scenario where PrognoScan LUAD data was used as the Training Set and the TCGA LUAD data as the Testing Set (**Figure Q, panel A in S1 File**), the GradientScanSurv method still achieved the largest percentage (36.71%) of total trials with the largest AUCs compared to other methods (**Figure Q, panel B in S1 File**). In addition, the GradientScanSurv method has the largest average AUCs compared to other methods in 3 out of 7 subsets of trials of data defined by 100 trials of TCGA data combined with one of the PrognoScan datasets with valid AUCs in ROC analysis, which is more than that of any other methods (**Figure Q, panel C in S1 File**). A relatively large proportion of the trials with NA’s for most or all methods may imply that some datasets may have intrinsic issues such as the curation of survival data or technical issues on expression data (**Tables F and G in S1 File**) so that none of the methods could achieve any positive calls.

#### Approach 3: Alignment with presumed biology

In order to further compare the actual results in terms of their underlying biological meaning, we collected the shared genes identified by all of the methods from multiple trials of LUAD datasets combined from both TCGA and PrognoScan data. Listed in **Table H in S1 File** are the only cases where at least one of the methods produced shared genes that significantly overlapped with the gene lists from both TCGA data and one of the PrognoScan datasets assessed by a one-tailed Fisher’s exact test. We assume that the overlapping genes with statistical significance between the two datasets are more likely to reflect the true underlying biology rather than being impacted by unrelated factors. These unrelated factors include bad datasets due to technical issues such as microarray or RNAseq issues from either or both datasets, bad survival data annotation and curation etc. Only the GradientScanSurv and PrognoScan methods have cases from all of the trials showing significant overlap by the estimated p-values (**Table I in S1 File**), although in one case (shown in a row in bold in **Tables H and I in S1 File**), only the GradientScanSurv method has a significant p-value indicating that GradientScanSurv has the most consistent performance using the standard adjusted p-value cutoff (< = 0.05). In addition, NRAS was consistently identified by the GradientScanSurv method as well as by PrognoScan and Cox regression method (on gene expression), indicative of presumed biologically relevant results, since NRAS expression is correlated with KRAS mutational status in LUAD [47] and thus may contribute to oncogenesis.

To further evaluate the underlying biological relevance from the resulting gene lists from these methods using different data sources, we compared the shared genes derived from each of these methods between two independent pancreatic cancer (PAAD) datasets (**Table 1**): one from the TCGA PAAD dataset (TCGAPAAD_Set), and the other from Australian Pancreatic Cancer dataset (AusPanc_Set). Again, only the cases with significant overlapping genes between the two datasets were considered (**Table 1**). Interestingly, the NRAS gene was consistently found in the lists of multiple trials of data only from the GradientScanSurv method and only when there is a statistically significant overlap between the TCGAPAAD_Set and AusPanc_Set (**Table K in S1 File**). NRAS was only seen in the shared gene lists from the GradientScanSurv method but not from those of all other methods, indicative of its best performance in uncovering genes with likely biological relevance. In the case of PAAD, where more than 94% Pancreatic cancer patients harboring KRAS mutations [48], there is also a strong association between KRAS mutation status and increased level of expression of NRAS [47]. Therefore, finding NRAS as a positive gene for PAAD data by the GradientScanSurv method within these tested datasets is in many ways consistent with the expected biology and similarly, lack of its detection may represent a shortcoming to any method that does not show significance.

**Table 1:** Comparison of shared genes identified by selected methods on two independent PAAD datasets.

TCGAPAAD_Set	AusPanc_Set	Adj.GoodCountPvals	Adj.CORRECTED_P_VALUE	Adj.CoxPvalbyRanks	Adj.COX_P_VALUE	Adj.MedianPvals	Adj.tertPvals
trial_1	trial_1	**NRAS**;EGFR;CCNA2;MCM4;E2F7;CDK2;TK1	BUB1;MCM4	EGFR;BUB1;CCNA2;MCM4;E2F7;CDK2	EGFR;BUB1;CCNA2;MCM4;E2F7;CDK2;RASA1	BUB1;CCNA2;MCM4	CCNA2;MCM4
trial_1	trial_3	**NRAS;**EGFR;BUB1;CCNA2;MCM4;RALB;E2F7;CDK2;TK1	BUB1;MCM4	EGFR;BUB1;CCNA2;MCM4;E2F7;CDK2	EGFR;BUB1;CCNA2;MCM4;E2F7;CDK2;RASA1	BUB1;CCNA2;MCM4	CCNA2;MCM4
trial_1	trial_4	**NRAS;**EGFR;BUB1;CCNA2;MCM4;RALB;E2F7;CASP8;CDK2;TK1	BUB1;MCM4	EGFR;BUB1;CCNA2;MCM4;E2F7;CDK2	EGFR;BUB1;CCNA2;MCM4;E2F7;CDK2;RASA1	BUB1;CCNA2;MCM4	CCNA2;MCM4
trial_2	trial_1	**NRAS**;CCNA2;MCM4;EGFR;E2F7;CDK2;TK1	BUB1;MCM4	BUB1;CCNA2;MCM4;EGFR;E2F7;CDK2	BUB1;CCNA2;MCM4;EGFR;E2F7;CDK2;RASA1	BUB1;CCNA2;MCM4	CCNA2;MCM4
trial_2	trial_3	**NRAS**;BUB1;CCNA2;MCM4;RALB;EGFR;E2F7;CDK2;TK1	BUB1;MCM4	BUB1;CCNA2;MCM4;EGFR;E2F7;CDK2	BUB1;CCNA2;MCM4;EGFR;E2F7;CDK2;RASA1	BUB1;CCNA2;MCM4	CCNA2;MCM4
trial_2	trial_4	**NRAS**;BUB1;CCNA2;MCM4;RALB;EGFR;E2F7;CASP8;CDK2;TK1	BUB1;MCM4	BUB1;CCNA2;MCM4;EGFR;E2F7;CDK2	BUB1;CCNA2;MCM4;EGFR;E2F7;CDK2;RASA1	BUB1;CCNA2;MCM4	CCNA2;MCM4
trial_3	trial_1	**NRAS**;EGFR;CCNA2;E2F7;MCM4;CDK2;TK1	BUB1;MCM4	EGFR;BUB1;CCNA2;E2F7;MCM4;CDK2	EGFR;BUB1;CCNA2;E2F7;MCM4;CDK2;RASA1	BUB1;CCNA2;MCM4	CCNA2;MCM4
trial_3	trial_3	**NRAS**;EGFR;BUB1;CCNA2;E2F7;MCM4;RALB;CDK2;TK1	BUB1;MCM4	EGFR;BUB1;CCNA2;E2F7;MCM4;CDK2	EGFR;BUB1;CCNA2;E2F7;MCM4;CDK2;RASA1	BUB1;CCNA2;MCM4	CCNA2;MCM4
trial_3	trial_4	**NRAS**;EGFR;BUB1;CCNA2;E2F7;MCM4;RALB;CDK2;CASP8;TK1	BUB1;MCM4	EGFR;BUB1;CCNA2;E2F7;MCM4;CDK2	EGFR;BUB1;CCNA2;E2F7;MCM4;CDK2;RASA1	BUB1;CCNA2;MCM4	CCNA2;MCM4
trial_4	trial_1	**NRAS**;EGFR;CCNA2;MCM4;E2F7;TK1;CDK2	BUB1;MCM4	EGFR;BUB1;CCNA2;MCM4;E2F7;CDK2	EGFR;BUB1;CCNA2;MCM4;E2F7;CDK2;RASA1	BUB1;CCNA2;MCM4	CCNA2;MCM4
trial_4	trial_3	**NRAS**;EGFR;BUB1;CCNA2;MCM4;RALB;E2F7;TK1;CDK2	BUB1;MCM4	EGFR;BUB1;CCNA2;MCM4;E2F7;CDK2	EGFR;BUB1;CCNA2;MCM4;E2F7;CDK2;RASA1	BUB1;CCNA2;MCM4	CCNA2;MCM4
trial_4	trial_4	**NRAS**;EGFR;BUB1;CCNA2;MCM4;RALB;E2F7;TK1;CDK2;CASP8	BUB1;MCM4	EGFR;BUB1;CCNA2;MCM4;E2F7;CDK2	EGFR;BUB1;CCNA2;MCM4;E2F7;CDK2;RASA1	BUB1;CCNA2;MCM4	CCNA2;MCM4
trial_5	trial_1	**NRAS**;EGFR;CCNA2;E2F7;MCM4;CDK2;TK1	BUB1;MCM4	EGFR;BUB1;CCNA2;E2F7;MCM4;CDK2	EGFR;BUB1;CCNA2;E2F7;MCM4;CDK2;RASA1	BUB1;CCNA2;MCM4	CCNA2;MCM4
trial_5	trial_3	**NRAS**;EGFR;BUB1;CCNA2;E2F7;MCM4;RALB;CDK2;TK1	BUB1;MCM4	EGFR;BUB1;CCNA2;E2F7;MCM4;CDK2	EGFR;BUB1;CCNA2;E2F7;MCM4;CDK2;RASA1	BUB1;CCNA2;MCM4	CCNA2;MCM4
trial_5	trial_4	**NRAS**;EGFR;BUB1;CCNA2;E2F7;MCM4;RALB;CASP8;CDK2;TK1	BUB1;MCM4	EGFR;BUB1;CCNA2;E2F7;MCM4;CDK2	EGFR;BUB1;CCNA2;E2F7;MCM4;CDK2;RASA1	BUB1;CCNA2;MCM4	CCNA2;MCM4

(TCGA PAAD data and Australian Cancer Data). Only the trials that have significant *p*-values in their one-tailed Fisher’s exact tests for the GradientScanSurv (Adj.GoodCountPvals) method (rows in bold from **Table K in S1 File**) assessing the significance of overlapping genes are listed here. All other methods have the same *p*-values (for the same data) and so the same shared genes for all trials. The GradientScanSurv method produced different *p*-values for each trial due to its permutation step. The full table with all trials is in **Table L in S1 File**. Identified genes shared by both the TCGA PAAD data (TCGAPAAD_Set) and the Australian Pancreatic Cancer data (AusPanc_Set) at the cutoff using the adjusted p-value< = 0.05 respectively using the selected methods are shown. Since the GradientScanSurv method has a permutation step, multiple trials of GradientScanSurv analysis were used in combination with results from other methods to form trials. In addition, whenever the NRAS gene was identified in the trials of data by the GradientScanSurv method (all trials in this table), the overlap of the identified genes showed the significance of overlap between the two datasets for the GradientScanSurv method, which was also highlighted in bold in **Table K in S1 File.**

### Performance comparison with penalized linear model-based Elastic Net and Lasso methods

Since the selected univariate methods that were compared with the GradientScanSurv method consider genes one at a time, we also wanted to evaluate whether the GradientScanSurv method performed better than other model methods such as penalized linear model-based methods including Elastic Net [49] and Lasso methods [50]. These methods consider all variables together in the model, which is essentially a Cox model with added features that allow them to do the variable selection. The conventional Cox regression method can also take multiple variables at a time, but the Elastic Net and Lasso methods are much more comprehensive than the conventional Cox regression model. Penalized linear model-based methods such as Elastic Net are about trying to find the minimal set of variables that matter when considered as a group, whereas our method is looking at the variables individually, which make the direct comparison much harder and it may be debatable whether ROC analysis is an appropriate way to evaluate performance. Thus, we decided to compare the results of each method using the same dataset to discover underlying biological relevance based upon the biological knowledge available. It is not a perfect heads-up comparison but still should inform us about the potential of our method in uncovering the underlying biology based on our understanding of RAS biology, which our RAS program focuses on.

As expected and discussed above, NRAS was once again identified by both the GradientScanSurv and Lasso method in LUAD showing strong association with patient survival outcome (**Table 2**). However, the GradientScanSurv method identified the other two RAS genes (KRAS and HRAS) as positive genes in PAAD, which were missed by the Lasso method. Once again, with more than 94% Pancreatic cancer patients carrying KRAS mutation [48] and RAS genes (KRAS, NRAS, and HRAS) were observed to interact with each other through their mutations status and gene expression [47], it is highly expected that RAS genes could be highly associated with survival outcome. In addition, FOSL1 was identified in both LUAD and PAAD by our method but missed by the Lasso method (**Table 2**). Very similar results were observed for the Elastic Net method, which also missed the FOSL1 gene in both LUAD and PAAD results (**Table M in S1 File**). FOSL1 was recently identified as a novel downstream effector of KRAS and higher FOSL1 expression was associated with poor survival outcome in both lung and pancreatic cancer patients [51]. Therefore, we take the presence of FOSL1 in the GradientScanSurv positive lists and its absence in both Lasso and Elastic Net method to be indicative of more biologically relevant results from the GradientScanSurv method.

**Table 2:** Comparison of results by GradientScanSurv and Lasso methods on TCGA tumor data (adjusted p< = 0.05).

Types	Common_Genes_Count	GSS_Genes_Count	Lasso_Genes_Count	Common_Genes	GSS_Genes_only	Lasso_Genes_Only
BRCA	8	17	28	CCNA1;ERF;EXOC1;FLT3;ICMT;PLXNB1;RAC2;STK3	CCND2;IRS2;JUN;PTK2;PTPN11;ROCK2;STK11;**BRCA1**;FANCC	SAV1;RASAL1;EIF4EBP1;PIK3CA;RASAL3;FGFR1;CDKN1A;RHOB;DUSP6;CASP7;FGFR4;RPS6KB1;RPS6KA3;PRKAG1;RPS6KA6;RALGDS;SCRIB;PRKAA2;CASP3;RHEB
COAD	0	0	0			
LUAD	4	6	12	**NRAS**;ECT2;DUSP5;SHC1	CCNA2;**FOSL1**	E2F7;GRB10;TYMS;RALGDS;YAP1;ALK;PIK3CA;RASSF9
PAAD	2	64	2	EXOC7;MET	**KRAS;NRAS**;ALK;BARD1;BRCA2;BRIP1;BUB1;CASP8;CBLC;CCNA2;CCND1;CCND2;CDC6;CDK2;CDK6;E2F1;E2F7;ECT2;EGFR;EIF4EBP1;ERBB2;EXOC1;EXOC3;EXOC4;FANCA;FANCC;**FOSL1**;**HRAS**;KSR1;KSR2;MCM4;PAK2;PEBP1;PIK3CA;PIK3R2;PIN1;PLXNB1;PPP1CA;RAC1;RALA;RALB;RALBP1;RALGDS;RASA2;RASA3;RASAL2;RASSF9;RCE1;RET;RHOC;SAV1;STK3;TFDP2;TK1;TSC1;TSC2;TYMS;UNG;**YAP1**;DUSP6;FNTA;RPTOR	
READ	0	1	0		**MET**	

Comparison of results derived from GradientScanSurv (GSS) and Lasso methods with TCGA tuor data (adjusted p< = 0.05) for the association of survival outcome with the expression of RAS pathway genes for a few selected tumor types: BRCA, COAD, LUAD, PAAD, and READ, which more likely have RAS genes involved. For GSS gene lists, LUAD, PAAD, and BRCA used multiple trials of results—the genes shown here were selected as common genes shared by multiple trials: LUAD (4 of 5 trials); PAAD (4 of 5 trials); BRCA (2 of 3 trials). Genes in bold are discussed in the text.

We also noticed that there were many genes identified by the GradientScanSurv method in PAAD, many more than those detected by Lasso (**Table 2**) or Elastic Net method (**Table M in S1 File**). However, both the Lasso and Elastic Net methods identified many more genes in BRCA data than the GradientScanSurv method. This may suggest that the number of genes in the positive lists of each method are dataset-dependent rather than method-dependent. Similarly, although LUAD, PAAD, COAD, and READ are associated with RAS mutational status and oncogenic activity, COAD has no positive genes for all of the test methods. READ had only one gene (MET) identified by the GradientScanSurv method. Interestingly, high MET expression has been found to be related to a worse prognosis and mortality for colorectal cancer patients [52]. READ and COAD should be very similar considering their physical locations and the fact that they are both adenocarcinomas. However, the variations between COAD (0 positive hit) and READ (1 positive hit) could be impacted by multiple factors such as sample collection, subtype differences, data quality, gene mutation relationships with gene expression etc. One could speculate that the lack of positive hits in TCGA COAD survival analysis could be due to mixed subtypes amongst COAD patients, which are not clinically homogeneous [11].

In addition, the genes DUSP6 [53], PIK3CA [54] and YAP1[55] play critical roles in pancreatic cancer oncogenesis and tumor maintenance, which consequently impacts survival outcome. Therefore, finding these genes associated with survival outcome in PAAD is expected and makes sense given our current understanding of the tumor biology (**Table 2** and **Table M in S1 File**). All of these observations suggest that despite the caveats associated with this comparison, the GradientScanSurv method appears to have better detection power compared to Lasso and Elastic methods to uncover the biologically relevant genes that are associated with cancer patient survival outcomes.

## Discussion

Historically, survival analysis has been focused on three types of goals: outcome-related gene finding, class discovery, and supervised prediction [12]. In this report, we mainly concentrated on outcome-related gene finding with the hope of uncovering relevant underlying biological mechanisms.

The highlighted examples illustrate the common pitfalls in publicly available survival analysis and validation web tools as well as the biological studies that have used these tools. Looking through the literature, one can easily find examples of the Kaplan-Meier estimator method and logrank tests that applied a limited set of predefined cutpoints to separate samples into high and low categories of genes, biomarkers, or prognostic scores, etc. In most, if not all cases, there is no justification or rationale provided in the published studies regarding why the particular cutpoint(s) was chosen if in fact that information is provided at all. They also do not discuss whether using other cutpoints did or did not produce significance. The first example regarding the KIAA1522 gene expression illustrated an implied bias using Kaplan-Meier Plotter website resulting from the fact that the given options for the tool include only a limited set of pre-defined cutpoints. The second example regarding RELN gene expression especially with the TCGA RNASeq LUAD dataset showed how we could miss supporting evidence, leading to a conflicting conclusion. This results from the fact that by chance the relation does not have logrank test significance at the commonly applied cutpoint.

We have introduced these papers simply as examples of potential pitfalls when using the available tools in the community. Their use was not meant to criticize the authors of these two papers for using a popular survival analysis tool, but rather to precaution the field as to how pitfalls can exist in commonly used tools. We used these two examples to show how a limited selection of analysis results from used tools could impact research results. In any case, our goal was to expose these aspects in order to avoid recurrence of such cases.

Such common pitfalls exist widely in the research community simply because no method has been reported that correctly handles the extrapolation of continuous data to categorical analysis or that comprehensively derives a p-value to assess, uncover, and validate the association of gene expression with survival outcomes. Our GradientScanSurv method potentially fills this gap by enabling the logrank test method to handle continuous variables and systematically assess the overall association with survival outcome along the ordered gradient in a comprehensive way. In addition, the visualization cues presented in the GradientScanSurv plots as vertical green lines representing the cutpoints, at which tentative cutpoint-wise significances of survival outcome were observed, provide a novel and informative way to visually sense the extent of the association of the expression of the genes with survival outcome. Furthermore, the directionality of the changes in outcome related to the expression levels can be seen from the GradientScanSurv plots as well. These visual cues provide good overall indicators of the data along the expression gradient that can consolidate the GoodCountPval regarding how well the gene expression is associated with survival outcome.

The PrognoScan method that derives a corrected p-value from the minimal p-value obtained along the expression gradient, is probably the most comprehensive logrank test method-based approach prior to the development of our GradientScanSurv method. Its main drawback is that it fails to provide a comprehensive consideration of the overall behavior along the whole expression gradient on the survival difference between the high and low expression groups. Rather, it produces a minimal p-value that is derived from one, albeit optimal, cutpoint. Although PrognoScan does evaluate the continuum of cutpoints to identify the optimal cutpoint and then derive a corrected minimal p-value from that, it fails to convey information about the rest of the continuum to uncover the association of gene expression with survival outcome. We assert that the GradientScanSurv method presumably corrects this deficiency by assessing the overall behavior of data for all the cutpoints along the expression gradient for the extent of association between gene expression and survival outcome. The side-by-side comparisons between the GradientScanSurv method and the PrognoScan method showed that GradientScanSurv better tolerates variations in datasets from the same disease but derived from different sources. Further, a more extensive global comparison with other survival analysis methods showed that our GradientScanSurv method has better overall performance in terms of consistency in multiple datasets tested by ROC analysis. It also appears to provide more biologically relevant results by evaluating the positive genes shared within the analysis results from independent datasets of the same diseases using the same analysis method.

It is possible to conclude that our method categorizes a continuous variable and there are plenty of methods that treat continuous variables as continuous such as the Cox model etc. However, we have directly compared our method with Cox models that treat expression data as a continuous variable in the model or even treat the ranked orders of the expression data as a continuous variable and indeed showed that our method is better than Cox models in both cases. In our view, considering many covariates such as age, tumor size etc simultaneously would likely confound the impact of gene expression on survival outcome when using the Cox model. Our method simplifies the question by testing how strongly the expression of an individual gene is associated with survival outcome by considering one gene at a time so that only the strong potential biomarker(s) are captured to move forward with more robust validation with multiple factors.

In addition, we would like to point out that by considering all cutpoints, comparing them and combining their logrank test statistics to an aggregate statistic, our proposed method achieves something that cannot be done by simply categorizing a variable. On the other hand, nonparametric methods all inevitably involve tuning parameters, for example, in spline, we have to choose a number of tuning parameters, such as the degrees, the number of knots, etc, and tuning all these parameters is a much more involved task. In contrast, our method is extremely simple and straightforward, and it is able to detect most nonlinear relationships.

When we used ROC analysis for global performance comparison, we alternately used either TCGA LUAD data or PrognoScan LUAD data as the Training Set or Testing Set. Since these datasets are derived from the same disease, they can be used as Training and Testing datasets respectively for cross-validation. This is more stringent than k-fold cross-validation within each dataset as is usually done. We prefer this approach because sample size would be reduced in the Training and Testing datasets due to a further partitioning of the samples if k-fold cross-validation were used. Of course, the reduction in sample size would be expected to have a significant impact on survival analysis. Therefore, cross-validation within two independent datasets of the same disease keeps the sample size as large as in the Training Set and Testing Set respectively. It also adds more stringent criteria to the cross-validation due to the independent data sources and even different platforms and technologies that created the datasets. In addition, it leverages the much better understanding of these diseases for their impact of the genes in the RAS pathway that can be credited to the accumulated studies in the field over the years as well as the more recent RAS Initiative (https://www.cancer.gov/research/key-initiatives/ras). Thus, the analysis results can be consolidated with expected biological themes with much better confidence.

Although simulation data has been widely used in the field to compare performance between methods, simulation in this context would require a more detailed understanding of the associated biology. A statistical simulation would, of course, help us to understand the type 1 error and robustness, however, that approach would make very strong assumptions in the underlying data generator, such as the true functional form of the hazard, or sparsity etc. Hence it becomes difficult to evaluate whether this approach would be useful in this particular context.

In order to compare our method with the more recent appearance of penalized linear model-based methods such as Lasso and Elastic Net methods, we also did a comparison with these methods that consider multiple genes together in terms of their ability to capture biologically relevant results. We were surprised to observe that our GradientScanSurv method was able to uncover more meaningful results based on alignment with biological expectations derived from our understanding of RAS biology’s role in a variety of cancer types that has emerged in recent years. These penalized linear model-based methods mainly focus on linear relationships of genes and how they relate to survival outcome. It was implied that even co-regulated genes may not necessarily all be associated with survival outcome simultaneously despite an attempt to use a network-based approach to explore this aspect [56]. Our preliminary comparison study comparing GradientScanSurv and penalized linear model-based methods suggested that GradientScanSurv may be able to take into account these aspects and associations that are not linearly related and would have been missed from these linear model-based methods.

Our GradientScanSurv method assesses one gene at a time, which has Pros and Cons. An obvious Pro is that this method does not have to include other factors and other genes in the analysis model presumably diminishing a tendency to over-fit the model since most datasets have a limited number of patients. This simplifies the method to only consider one variable at a time, and also make its interpretation more straightforward [3]. The Con is that ignorance of other factors or other genes may miss consideration of the interaction(s) of these genes. For example, recent statistical theoretical analysis of the random survival forest [45, 57] model shows that the exhaustive cutpoint search can be potentially biased if the censoring distribution also depends on the covariates (https://arxiv.org/pdf/1707.09631v3.pdf), which may lead to the sub-optimal power of the proposed method. However, our comparison results may suggest the Pro may outweigh the Con side, since there are so many genes encoded by the genome, considering them altogether may make the model clumsy and hard to interpret. If we scan the genes one at a time with GradientScanSurv to collect a list, and then use these integration methods to consider the positive hit genes derived from the first-round of GradientScanSurv analysis later, this may be a feasible and reasonable way to study the interactions of these genes, but this is outside of scope of this study.

As mentioned earlier, gene expression cluster or gene signature-based survival analysis biomarkers have encountered issues of reproducibility and robustness [14]. Many of the gene signature biomarkers reported in the literature do not have enough validation in the first place. In contrast, the single gene expression gradient is a compelling place for us to look for potential outcome-related biomarkers. First, our expression gradient-based method classifies the patients based on their ranked order of the expression level, rather than the actual signal intensity, which is much more resistant to variations of measurement types (e.g., microarray probe intensity, RSEM value of RNAseq etc.). Consequently, a gene uncovered by our gradient-based method will be easier to use as a biomarker and easier to validate using different data sources that may be derived using different platforms or technologies. Secondly, the rank order of expression levels is independent of the data transformation and normalization methods used. In contrast, data transformation and normalization methods would very likely impact any of the signature or cluster-based survival analysis methods. Thirdly, our expression gradient-based method can be used to derive individual biomarker genes as a first round, with subsequent application of other methods to derive aggregate scores for each sample by combining these potential biomarker genes from the first round. From this, the gradient of these aggregate scores for all samples can be subsequently subjected to our gradient method for survival analysis for the 2^nd^ round. This procedure can be flexibly used for biomarker discovery at the individual gene level or at the aggregate level of multiple “good” biomarker genes. The aggregate biomarkers can be flexibly tested for permutated combinations of the identified potential individual biomarkers until better aggregate biomarkers are obtained.

Our method is not limited to application with gene expression data. In fact, any continuous variable can be used with our method for survival analysis for its association with outcome. The univariate Cox regression model is sensitive to the actual data range and scale, since as we tested the Cox regression model with either actual expression levels or ranks based on relative expression levels and these gave quite different results. This is why we also compared Cox regression results using the rank of gene expression in addition to using the actual expression intensities. Our method can be used to derive risk scores that classify the cancer patients either through a combination of risk scores of each potential gene or through aggregate risk scores of combined potential gene lists.

Taken together, our results show that the GradientScanSurv method can be used as either a validation or discovery tool for biomarkers of continuous variables. It produces better performance than all of the selected methods we compared for the selected example datasets. Our comparisons included most typically existing methods and commonly used public web tools in the field to directly assess the association of gene expression with survival outcome to our knowledge, although it is impossible to compare all of the tools in the field at one time. Our comparative analysis has also revealed the importance of carefully capturing survival data along with the associated metadata for ongoing genomic disease studies. The data we accessed have relatively high levels of sample drop out in terms of life-status reporting that significantly hampered our analysis and we look forward to applying this method to more complete datasets as they emerge.

## Supporting information

S1 File SupplementaryData.docx. Supporting information including **Tables A-M** and **Figures A-Q** that were pooled into one single Supplementary Data file.
(DOCX)

## References

[1] Clark TG, Bradburn MJ, Love SB, Altman DG. Survival analysis part I: basic concepts and first analyses. Br J Cancer 2003, 89(2):232–8. 10.1038/sj.bjc.6601118 12865907 10.1038/sj.bjc.6601118 PMC2394262

[2] Clark TG, Bradburn MJ, Love SB, Altman DG. Survival analysis part IV: further concepts and methods in survival analysis. Br J Cancer 2003, 89(5):781–6. 10.1038/sj.bjc.6601117 12942105 10.1038/sj.bjc.6601117 PMC2394469

[3] Bradburn MJ, Clark TG, Love SB, Altman DG. Survival analysis part II: multivariate data analysis—an introduction to concepts and methods. Br J Cancer 2003, 89(3):431–6. 10.1038/sj.bjc.6601119 12888808 10.1038/sj.bjc.6601119 PMC2394368

[4] Bradburn MJ, Clark TG, Love SB, Altman DG. Survival analysis Part III: multivariate data analysis—choosing a model and assessing its adequacy and fit. Br J Cancer 2003, 89(4):605–11. 10.1038/sj.bjc.6601120 12915864 10.1038/sj.bjc.6601120 PMC2376927

[5] Chen X, Sun X, Hoshida Y. Survival analysis tools in genomics research. Hum Genomics 2014, 8:21 10.1186/s40246-014-0021-z 25421963 10.1186/s40246-014-0021-z PMC4246473

[6] Altman DG, Lausen B, Sauerbrei W, Schumacher M. Dangers of using "optimal" cutpoints in the evaluation of prognostic factors. J Natl Cancer Inst 1994, 86(11):829–35. 8182763 10.1093/jnci/86.11.829

[7] Mehta S, Shelling A, Muthukaruppan A, Lasham A, Blenkiron C, Laking G et al Predictive and prognostic molecular markers for cancer medicine. Ther Adv Med Oncol. 2010, 2(2):125–48. 10.1177/1758834009360519 21789130 10.1177/1758834009360519 PMC3126011

[8] Bøvelstad HM, and Borgan O. Assessment of evaluation criteria for survival prediction from genomic data. Biom J. 2011, 53(2):202–16. 10.1002/bimj.201000048 21308723 10.1002/bimj.201000048

[9] Lawrence MS, Stojanov P, Polak P, Kryukov GV, Cibulskis K, Sivachenko A et al Mutational heterogeneity in cancer and the search for new cancer-associated genes. Nature. 2013, 499(7457):214–218. 10.1038/nature12213 23770567 10.1038/nature12213 PMC3919509

[10] Stephen AG, Esposito D, Bagni RK, and McCormick F. Dragging ras back in the ring. Cancer Cell 2014, 25(3):272–81. 10.1016/j.ccr.2014.02.017 24651010 10.1016/j.ccr.2014.02.017

[11] Yokota T. Are KRAS/BRAF mutations potent prognostic and/or predictive biomarkers in colorectal cancers? Anticancer Agents Med Chem. 2012, 12(2):163–71. 10.2174/187152012799014968 22043994 10.2174/187152012799014968 PMC3343383

[12] Dupuy A, and Simon RM. Critical review of published microarray studies for cancer outcome and guidelines on statistical analysis and reporting. J Natl Cancer Inst. 2007, 99(2):147–57. 10.1093/jnci/djk018 17227998 10.1093/jnci/djk018

[13] Kern SE. Why your new cancer biomarker may never work: recurrent patterns and remarkable diversity in biomarker failures. Cancer Res. 2012, 72(23):6097–101. 10.1158/0008-5472.CAN-12-3232 23172309 10.1158/0008-5472.CAN-12-3232 PMC3513583

[14] Koscielny S. Why most gene expression signatures of tumors have not been useful in the clinic. Sci Transl Med 2010, 2(14):14ps2 10.1126/scitranslmed.3000313 20371465 10.1126/scitranslmed.3000313

[15] Zhu CQ, and Tsao MS. Prognostic markers in lung cancer: is it ready for prime time? Transl Lung Cancer Res. 2014, 3(3):149–58. 10.3978/j.issn.2218-6751.2014.06.09 25806294 10.3978/j.issn.2218-6751.2014.06.09 PMC4367687

[16] Ein-Dor L, Zuk O, Domany E. Thousands of samples are needed to generate a robust gene list for predicting outcome in cancer. Proc Natl Acad Sci U S A. 2006, 103(15):5923–8. 10.1073/pnas.0601231103 16585533 10.1073/pnas.0601231103 PMC1458674

[17] Coppedè F, Lopomo A, Spisni R, Migliore L. Genetic and epigenetic biomarkers for diagnosis, prognosis and treatment of colorectal cancer. World J Gastroenterol. 2014, 20(4):943–56. 10.3748/wjg.v20.i4.943 24574767 10.3748/wjg.v20.i4.943 PMC3921546

[18] van Wieringena WN, Kun D, Hampel R, and Boulesteix AL. Survival prediction using gene expression data: A review and comparison. Computational Statistics & Data Analysis 2009, 53(5):1590–1603

[19] Prat A, Ellis MJ, Perou CM. Practical implications of gene-expression-based assays for breast oncologists. Nat Rev Clin Oncol. 2011, 9(1):48–57. 10.1038/nrclinonc.2011.178 22143140 10.1038/nrclinonc.2011.178 PMC3703639

[20] Ishibashi Y, Hanyu N, Nakada K, Suzuki Y, Yamamoto T, Yanaga K et al Profiling gene expression ratios of paired cancerous and normal tissue predicts relapse of esophageal squamous cell carcinoma. Cancer Res. 2003, 63(16):5159–64. 12941848

[21] Nutt CL, Mani DR, Betensky RA, Tamayo P, Cairncross JG, Ladd C et al Gene expression-based classification of malignant gliomas correlates better with survival than histological classification. Cancer Res. 2003, 63(7):1602–7. 12670911

[22] Chang HY, Nuyten DS, Sneddon JB, Hastie T, Tibshirani R, Sørlie T et al Robustness, scalability, and integration of a wound-response gene expression signature in predicting breast cancer survival. Proc Natl Acad Sci U S A. 2005, 102(10):3738–43. 10.1073/pnas.0409462102 15701700 10.1073/pnas.0409462102 PMC548329

[23] Ma XJ, Wang Z, Ryan PD, Isakoff SJ, Barmettler A, Fuller A et al A two-gene expression ratio predicts clinical outcome in breast cancer patients treated with tamoxifen. Cancer Cell 2004, 5(6):607–16. 10.1016/j.ccr.2004.05.015 15193263 10.1016/j.ccr.2004.05.015

[24] Dhanasekaran SM, Barrette TR, Ghosh D, Shah R, Varambally S, Kurachi K et al Delineation of prognostic biomarkers in prostate cancer. Nature 2001, 412(6849):822–6. 10.1038/35090585 11518967 10.1038/35090585

[25] Singh D, Febbo PG, Ross K, Jackson DG, Manola J, Ladd C et al Gene expression correlates of clinical prostate cancer behavior. Cancer Cell 2002, 1(2):203–9. 12086878 10.1016/s1535-6108(02)00030-2

[26] Venet D, Dumont JE, and Detours V. Most Random Gene Expression Signatures Are Significantly Associated with Breast Cancer Outcome. PLoS Comput Biol. 2011, 7(10): e1002240 10.1371/journal.pcbi.1002240 22028643 10.1371/journal.pcbi.1002240 PMC3197658

[27] Wigle DA, Jurisica I, Radulovich N, Pintilie M, Rossant J, Liu N et al Molecular profiling of non-small cell lung cancer and correlation with disease-free survival. Cancer Res. 2002, 62(11):3005–8. 12036904

[28] Beer DG, Kardia SL, Huang CC, Giordano TJ, Levin AM, Misek DE. Gene-expression profiles predict survival of patients with lung adenocarcinoma. Nat Med. 2002, 8(8):816–24. 10.1038/nm733 12118244 10.1038/nm733

[29] Pomeroy SL, Tamayo P, Gaasenbeek M, Sturla LM, Angelo M, McLaughlin ME et al Prediction of central nervous system embryonal tumour outcome based on gene expression. Nature 2002, 415(6870): 436–442. 10.1038/415436a 11807556 10.1038/415436a

[30] Chen HY, Yu SL, Chen CH, Chang GC, Chen CY, Yuan A et al A five-gene signature and clinical outcome in non-small-cell lung cancer. N Engl J Med. 2007, 356(1):11–20. 10.1056/NEJMoa060096 17202451 10.1056/NEJMoa060096

[31] Liang Y, Chai H, Liu XY, Xu ZB, Zhang H, Leung KS. Cancer survival analysis using semi-supervised learning method based on Cox and AFT models with L1/2 regularization. BMC Med Genomics 2016, 9:11 10.1186/s12920-016-0169-6 26932592 10.1186/s12920-016-0169-6 PMC4774162

[32] Bair E, Tibshirani R. Semi-supervised methods to predict patient survival from gene expression data. PLoS Biol. 2004, 2(4):E108 10.1371/journal.pbio.0020108 15094809 10.1371/journal.pbio.0020108 PMC387275

[33] Györffy B, Surowiak P, Budczies J, and Lanczky A, Online survival analysis software to assess the prognostic value of biomarkers using transcriptomic data in non-small-cell lung cancer. PLoS ONE 2013, 8(12):e82241 10.1371/journal.pone.0082241 24367507 10.1371/journal.pone.0082241 PMC3867325

[34] Mihály Z, Kormos M, Lánczky A, Dank M, Budczies J, Szász MA. et al A meta-analysis of gene expression-based biomarkers predicting outcome after tamoxifen treatment in breast cancer. Breast Cancer Res Treat. 2013, 140(2):219–32. 10.1007/s10549-013-2622-y 23836010 10.1007/s10549-013-2622-y

[35] Castellano E, Molina-Arcas M, Krygowska AA, East P, Warne P, Nicol A et al RAS signalling through PI3-Kinase controls cell migration via modulation of Reelin expression. Nat Commun. 2016, 7:11245 10.1038/ncomms11245 27071537 10.1038/ncomms11245 PMC4833863

[36] Liu YZ, Yang H, Cao J, Jiang YY, Hao JJ, Xu X et al KIAA1522 is a novel prognostic biomarker in patients with non-small cell lung cancer. Sci Rep. 2016, 6:24786 10.1038/srep24786 27098511 10.1038/srep24786 PMC4838871

[37] Muzumdar MD, Chen PY, Dorans KJ, Chung KM, Bhutkar A, Hong E et al Survival of pancreatic cancer cells lacking KRAS function. Nat Commun. 2017, 8(1):1090 10.1038/s41467-017-00942-5 29061961 10.1038/s41467-017-00942-5 PMC5653666

[38] Goswami CP, and Nakshatri H. PROGgene: gene expression based survival analysis web application for multiple cancers. J Clin Bioinforma 2013, 28;3(1):22 10.1186/2043-9113-3-22 PMC3875898 24165311

[39] Mizuno H, Kitada K, Nakai K, Sarai A. PrognoScan: a new database for meta-analysis of the prognostic value of genes. BMC Med Genomics 2009, 2:18. 10.1186/1755-8794-2-18 PMC2689870 19393097

[40] Miller R and Siegmund D. Maximally Selected Chi Square Statistics Biometrics 1982, 38(4):1011–1016

[41] Mazumdar M, and Glassman JR. Categorizing a prognostic variable: review of methods, code for easy implementation and applications to decision-making about cancer treatments. Stat Med. 2000, 19(1):113–32. 10623917 10.1002/(sici)1097-0258(20000115)19:1<113::aid-sim245>3.0.co;2-o

[42] Benjamini Y., and Hochberg Y. (1995). Controlling the false discovery rate: a practical and powerful approach to multiple testing. Journal of the Royal Statistical Society Series B, 57, 289–300

[43] Efron B. Bootstrap methods: another look at the jackknife In Breakthroughs in statistics, Springer, New York, NY; 1992 p. 569–593.

[44] Breiman Leo. Random forests. Machine learning 2001, 45(1):5–32.

[45] Ishwaran H. The effect of splitting on random forests. Machine Learning, 2015, 99:75–118. 10.1007/s10994-014-5451-2 28919667 10.1007/s10994-014-5451-2 PMC5599182

[46] Greulich H. The genomics of lung adenocarcinoma: opportunities for targeted therapies. Genes Cancer 2010, 1(12):1200–10. 10.1177/1947601911407324 21779443 10.1177/1947601911407324 PMC3092285

[47] Stephens RM, Yi M, Kessing B, Nissley DV, McCormick F. Tumor RAS Gene Expression Levels Are Influenced by the Mutational Status of RAS Genes and Both Upstream and Downstream RAS Pathway Genes. Cancer Informatics 2017, 16:1176935117711944. 10.1177/1176935117711944 PMC5467702 28634423

[48] Waters AM, and Der CJ. KRAS: the critical driver and therapeutic target for pancreatic cancer. Cold Spring Harbor Perspectives in Medicine. 12 11, 2017 10.1101/cshperspect.a031435 PMC5995645 29229669

[49] Zou H, and Hastie T. Regularization and Variable Selection via the Elastic Net. Journal of the Royal Statistical Society 2005, Series B: 301–320.

[50] Tibshirani R. Regression Shrinkage and Selection via the lasso. Journal of the Royal Statistical Society 1996, Series B (methodological). Wiley. 58 (1): 267–88.

[51] Vallejo A, Perurena N, Guruceaga E, Mazur PK, Martinez-Canarias S, Zandueta C et al An integrative approach unveils FOSL1 as an oncogene vulnerability in KRAS-driven lung and pancreatic cancer. Nat Commun. 2017, 8:14294 10.1038/ncomms14294 28220783 10.1038/ncomms14294 PMC5321758

[52] De Oliveira AT, Matos D, Logullo AF, DA Silva SR, Neto RA, Filho AL, et al MET Is highly expressed in advanced stages of colorectal cancer and indicates worse prognosis and mortality. Anticancer Res. 2009, 29(11):4807–11. 20032439

[53] Cancer Genome Atlas Research Network. Integrated Genomic Characterization of Pancreatic Ductal Adenocarcinoma. Cancer Cell 2017, 32(2):185–203.e13. 10.1016/j.ccell.2017.07.007 28810144 10.1016/j.ccell.2017.07.007 PMC5964983

[54] Payne SN, Maher ME, Tran NH, Van De Hey DR, Foley TM et al PIK3CA mutations can initiate pancreatic tumorigenesis and are targetable with PI3K inhibitors. Oncogenesis 2015, 4:e169 10.1038/oncsis.2015.28 26436951 10.1038/oncsis.2015.28 PMC4632089

[55] Kapoor A, Yao W, Ying H, Hua S, Liewen A, Q et al Yap1 activation enables bypass of oncogenic Kras addiction in pancreatic cancer. Cell 2014, 158(1):185–197 10.1016/j.cell.2014.06.003 24954535 10.1016/j.cell.2014.06.003 PMC4109295

[56] Zhang W, Ota T, Shridhar V, Chien J, Wu B, Kuang R. Network-based survival analysis reveals subnetwork signatures for predicting outcomes of ovarian cancer treatment. PLoS Comput Biol. 2013, 9(3):e1002975 10.1371/journal.pcbi.1002975 23555212 10.1371/journal.pcbi.1002975 PMC3605061

[57] Ishwaran H, Kogalur UB, Blackstone EH, and Lauer MS. Random survival forests. The annals of applied statistics 2008, 2(3): 841–860.

