# Supplementary material for: GradientScanSurv—An exhaustive association test method for gene expression data with censored survival outcome
Source: PLoS One. 2018 Dec 5;13(12):e0207590. doi: 10.1371/journal.pone.0207590 (PMC6281197; doi:10.1371/journal.pone.0207590)
Supplement: S1 File — Supporting information including Tables A-M and Figures A-Q that were pooled into one single Supplementary Data file. (DOCX) [file pone.0207590.s001.docx]

**GradientScanSurv—An exhaustive association test method for gene expression data with censored survival outcome**

Ming Yi^1^,*, Ruoqing Zhu^2^, Robert M. Stephens^1^

^1^NCI RAS Initiative, Cancer Research Technology Program, Frederick National Laboratory for Cancer Research, Frederick, MD, United States of America.

^2^Department of Statistics, University of Illinois Urbana-Champaign, Champaign, IL, United States of America

*Corresponding author

E-mail: [yiming@mail.nih.gov](mailto:yiming@mail.nih.gov).

Supporting info: Including **Tables A-M** and **Figures A-Q**

**Figure A.** Re-exploration of published data from a paper of highly-ranked journal. Re-exploration of original *Figure 3B* in the paper of the first study (see main text): Liu et al Sci Rep 2016, 6:24786 [36]. Kaplan-Meier Plotter website (kmplot.com[5,33]) was used to create the results of KIAA1522 gene (only one probeset 224746_at) at all available options for cutpoints to split patients by for stage II lung cancer patients (using version 2015). KIAA1522 gene was used as input and selected the 5 available cutpoint options as example and selected Stage 2 (n=320). (A). result of KIAA1522 gene using lower quartile as cutpoint to split patients by. (B). result of KIAA1522 gene using lower tertile as cutpoint to split patients by. (C). result of KIAA1522 gene using median as cutpoint to split patients by. (D). result of KIAA1522 gene using upper tertile as cutpoint to split patients by. (E). result of KIAA1522 gene using upper quartile as cutpoint to split patients by.

**Figure B.** Re-exploration of published data from another paper of highly-ranked journal. Re-exploration of the original *Figure 6e* in the paper of the second study (see main text): Castellano et al Nature Comm 2016, 7:11245 [35]. The survival outcome difference at all cutpoints along the gene expression gradient of RELN gene using visual scheme of proposed GradientSurvScan method was displayed. Original survival and expression data was kindly provided from the authors of a web tool KM-plotter (http:/kmplot.com [5,33]). Microarray data merged from GEO, TCGA and CaBig. The vertical green lines along the expression gradient at x-axis for all samples indicated significant survival outcome difference evaluated by logrank test between the higher expression group (samples left to cutpoint) vs lower expression group (samples right to cutpoint) separated at the corresponding cutpoints along the expression gradient. The red vertical line at the middle indicated the median cutpoint that the paper used (Castellano et al Nature Comm 2016, 7:11245 [35]).

**Figure C.** Exploration of TCGA LUAD dataset for survival outcome difference at all cutpoints along the gene expression gradient of RELN gene using visual scheme of proposed GradientSurvScan method. Analysis used downloaded TCGA LUAD RNAseq dataset and survival data. The vertical green lines along the expression gradient at x-axis for all samples indicated significant survival outcome difference evaluated by logrank test between the higher expression group (samples left to cutpoint) vs lower expression group (samples right to cutpoint) separated at the corresponding cutpoints along the expression gradient. The red vertical line at the middle indicated the median cutpoint that the paper used (the original *Figure 6e* in the paper Castellano et al Nature Comm 2016, 7:11245 [35]).

| **Table A. Examples of common pitfalls of survival analysis in public available survival analysis web tools and biological studies** | | | | | | |
| --- | --- | --- | --- | --- | --- | --- |
| Original Figures with Problems | Reference | Types of Publication and Journal | Variables | Choice or Strategy of Cutpoint for Validation | Problems of cutpoint | Explanation |
| Figure 3B | Liu et al 2016 [36] | Biological Research published in Nature Sci Report | KIAA1522 gene expression | lower quartile | only choose this cutpoint out of 5 options in the tool used | only use logrank test at fixed cutpoint to represent the overall association |
| Figure 6e | Castellano et al 2016 [35] | Biological Research published in Nature Commun. | REKN gene expression | Median | only choose this cutpoint out of 5 options in the tool used | only use logrank test at fixed cutpoint to represent the overall association |
| Figure 2A, 3, 4 | Aguirre-Gamboa et al 2013 [58] | Survival analysis web tool published in PLOS One | PI (Prognostic Index) | where the minimal logrank test p-value occurs | Fixed cutpoint at minimal logrank test p-value | only use logrank test at fixed cutpoint to represent the overall association |
| Figure 5A | Budczies et al 2012 [59] | Survival analysis web tool published in PLOS One | PgR gene expression | where the optimal cutoff is found | Optimal cut off point as the only cutpoint | only use optimal cutpoint to represent the overall association |
| Figure 1, 2 | Chen et al 2007 [30] | Biological Research published in N Engl J Med | 16-gene or 5-gene signature-based risk scores | separate the samples into high risk and low risk group based on decision tree | fixed cutpoint | only use logrank test at fixed cutpoint to represent the overall association |
| Figure 2A, 2C | Dave et al 2004 [60] | Biological Research published in N Engl J Med | PSP score or IPI scores | quartile | fixed cutpoint | only use logrank test at fixed cutpoint to represent the overall association |
| Figure 1B, 2 | Davicioni et al 2010 [61] | Biological Research published in J Clin Oncol | Metagene predictor scores | Tertile | fixed cutpoint | only use logrank test at fixed cutpoint to represent the overall association |
| Figure 2f, 3d | Dhanasekaran et al 2001 [62] | Biological Research published in Nature | hepsin gene or PIM1 gene expression | specific cutoff for low and high for hepsin and PIM1 not clearly specified | fixed cutpoint | only use logrank test at fixed cutpoint to represent the overall association |
| Figure 3 | Gui and Li 2005 [63] | Survival analysis method published in Pac Symp BioComput | estimated risk scores | mean | fixed cutpoint | only use logrank test at fixed cutpoint to represent the overall association |
| Figure 3, 4 | Goswami and Nakshatri 2013 [64] | Survival analysis web tool published in J Clin Bioinforma | gene expression or gene signature | median for single gene expression or mean of gene signature | fixed cutpoint | only use logrank test at fixed cutpoint to represent the overall association |
| Figure 2 | Gyorffy et al 2013 [33] | Survival analysis web tool published in PLoS ONE | gene expression | median | fixed cutpoint | only use logrank test at fixed cutpoint to represent the overall association |
| Figure 3B | Martinez-Ledesma et al 2015 [65] | Survival analysis method by network algorithm published in Nature Sci Report | prognostic index derived from expression of multi-genes | median | fixed cutpoint | only use logrank test at fixed cutpoint to represent the overall association |
| Figure 8e, 8f, 10b | Muzumdar et al 2017 [66] | Biological Research published in Nature Commun | Correlation scores by ssGSEA with combined knockout signature or with signatures from Bailey et al | quintile (top vs bottom) | fixed cutpoint | only use logrank test at fixed cutpoint to represent the overall association |
| Figure 4d | Robinovich et al 2015 [67] | Biological Research published in Nature | ASS1 and Citrin gene expression | Not clearly defined or described in main text or method except mentioning ASS1 low citrin high etc. | cutpoint not clearly defined or described | only use logrank test at fixed cutpoint to represent the overall association |
| Figure 2C, 2D | Ringner et al 2011 [68] | Survival analysis web tool published in PLOS One | CCNB1 gene expression | quantiles | fixed cutpoint | only use logrank test at fixed cutpoint to represent the overall association |
| Figure 4, 5 | Tang et al 2017 [69] | Survival analysis method by spike-and-slab lasso Cox model published in Bioinformatics | spike-and-slab lasso Cox model | median (percentile 0-50 vs percentile 50-100) | fixed cutpoint | only use logrank test at fixed cutpoint to represent the overall association |
| Figure 2, 3 | Wu and Stein 2012 [70] | Survival analysis method by network algorithm published in Genome Biol | module 2 expression | median | fixed cutpoint | only use logrank test at fixed cutpoint to represent the overall association |
| Figure 9 | Zhang et al 2013 [71] | Survival analysis method by network algorithm published in PLoS Computational Biology | FBN1 gene expression | median | fixed cutpoint | only use logrank test at fixed cutpoint to represent the overall association |
| Figure 2b | Nagy et al 2017 [72] | Biological Research published in Int J Cancer | KRAS gene expression | Lower and upper quartile | fixed cutpoint | only use logrank test at fixed cutpoint to represent the overall association |

**Table A.** Examples of common pitfalls of survival analysis in public available survival analysis web tools and biological studies

**Figure D.** Typical GradientScanSurv graphic result:

The number of vertical green lines (at which cutpoints there exist significant logrank test p-values) would reflect the extent of the association of gene expression with survival outcome. The brown diamonds either at the top side or at the bottom side of the plot would show the direction of the association with higher expressors would die faster or lower expressors would die faster respectively.

**Figure E.** Validation of NRAS result of GradientScanSurv on TCGA LUAD RNAseq dataset. The red arrow points to the report on GoodCountPval at 0.001. The Brown diamonds along the top part of the plot showed that NRAS higher expressors would die faster than those lower expressors, which is consistent with **Fig 2D**.

**Figure F.** Comparison of GradientScanSurv and PrognoScan Results of MAP2K1 gene in LUAD datasets downloaded from PrognoScan.

(A). Joined table of GradientScanSurv result that run on downloaded data from PrognoScan and direct result run from PrognoScan website for MAP2K1 gene in the same LUAD (lung adenocarcinoma) datasets. The red arrow indicated a dataset where GradientScanSurv called a significant association with GoodCountPval=0.01 at but PrognoScan missed the call with CORRECTED.P.VALUE=0.105. (B). Screenshot of PrognoScan result’s expression gradient-based logrank p-values plot. The blue vertical line indicated where the minimal p-value is that was used for final CORRECTED.P.VALUE. (C). Screenshot of PrognoScan result’s report with final CORRECTED.P.VALUE at 0.105. (D). GradientScanSurv gene expression gradient-based logrank p-values plot with GoodCountPval at 0.01. The green vertical lines indicated specific cut-points where corresponding logrank p-values are significant at p-value<=0.05. Univariant expression-based coxph p-value and expression-rank based coxph p-value by ranks are also reported here same as in Panel A**.** This result is consistent with TCGA validation result in p-value (0.001) and in that higher expressors of NRAS dies faster indicated by the brown diamonds along the top part of the plots.

**Figure G.** Comparison of GradientScanSurv and PrognoScan results for MAP2K1 gene in LUAD datasets downloaded from PrognoScan for dataset Jacob-00182-MSK. (A). Joined table of GradientScanSurv result that run on downloaded data from PrognoScan and direct result run from PrognoScan website for MAP2K1 gene in the same LUAD (lung adenocarcinoma) datasets. The red arrow indicated a dataset (Jacob-00182-MSK) where GradientScanSurv called a significant association with GoodCountPval=0.041 at but PrognoScan missed the call with CORRECTED.P.VALUE=0.066. This is the same as the dataset GSE31210 with RFS data. (B). Screenshot of PrognoScan result’s expression gradient-based logrank p-values plot. The blue vertical line indicated where the minimal p-value is that was used for final CORRECTED.P.VALUE. (C). Screenshot of PrognoScan result’s report with final CORRECTED.P.VALUE at 0.066. (D). GradientScanSurv gene expression gradient-based logrank p-values plot with GoodCountPval at 0.041. The green vertical lines indicated specific cut-points where corresponding logrank p-values are significant at p-value<=0.05. Univariant expression-based coxph p-value and expression-rank based coxph p-value by ranks are also reported here same as in Panel A. This result is consistent with TCGA validation result in p-value (0.001) and in that higher expressors of NRAS dies faster indicated by the brown diamonds along the top part of the plots.

**Figure H.** Validation of MAP2K1 result of GradientScanSurv on TCGA LUAD RNAseq dataset. The red arrow points to the report on GoodCountPval at 0.031. The Brown diamonds along the top part of the plot showed that MAP2K1 higher expressors would die faster than those lower expressors, which is consistent with **panel D in Figure F and panel D in Figure G.**

**Figure I.** Validation of KRAS result of GradientScanSurv on TCGA LUAD RNAseq dataset. The red arrow points to the report on GoodCountPval at 0.004. The Brown diamonds along the top part of the plot showed that KRAS higher expressors would die faster than those lower expressors, which seems opposite with **Fig 3C**. This would be due to the location and design of array probesets (see explanation in main text and following figures).

**Figure J**. Comparison of GradientScanSurv and PrognoScan results for KRAS gene in LUAD datasets downloaded from PrognoScan with focus on one dataset GSE31210 by probeset 204010_s_at for survival type OS (overall survival) indicated by the red arrow in panel A. (A). Joined table of GradientScanSurv result that run on downloaded data from PrognoScan and direct result run from PrognoScan website for KRAS gene in the same LUAD (lung adenocarcinoma) datasets. The arrow indicated a dataset where GradientScanSurv called a significant association with GoodCountPval=0 (<0.001 by n=1000 permutations), but PrognoScan also made the call with CORRECTED.P.VALUE=0.0003. (B). Screenshot of PrognoScan result’s report for dataset indicated by red arrow in panel A. (C). GradientScanSurv gene expression gradient-based logrank p-values plot with GoodCountPval at 0 (<0.001 by n=1000 permutations) for dataset indicated by red arrow in panel A. This result is consistent with TCGA Validation result in p-value (0.004). However, from this dataset, the plot showed lower expressors of KRAS (measured by probeset 204010_s_at) dies faster indicated by the brown diamonds along the top part of the plots, which is opposite with TCGA validation result on **Figure I**. This would be due to the location and design of array probesets (see explanation in main text).

**Figure K**. Comparison of GradientScanSurv and PrognoScan results for KRAS gene in LUAD datasets downloaded from PrognoScan with focus on one dataset GSE31210 by probeset 204010_s_at for survival type RFS (relapse-free survival) indicated by the red arrow in panel A. (A). Joined table of GradientScanSurv result that run on downloaded data from PrognoScan and direct result run from PrognoScan website for KRAS gene in the same LUAD (lung adenocarcinoma) datasets. The arrow indicated a dataset where GradientScanSurv called a significant association with GoodCountPval=0 (<0.001 by n=1000 permutations), but PrognoScan also made the call with CORRECTED.P.VALUE=0.000085. (B). Screenshot of PrognoScan result’s report for dataset indicated by red arrow in panel A. (C). GradientScanSurv gene expression gradient-based logrank p-values plot with GoodCountPval at 0 (<0.001 by n=1000 permutations) for dataset indicated by red arrow in panel A. This result is consistent with TCGA Validation result in p-value (0.004). However, from this dataset, the plot showed lower expressors of KRAS (measured by probeset 204010_s_at) dies faster indicated by the brown diamonds along the top part of the plots, which is opposite with TCGA validation result on

**Figure I.** This would be due to the location and design of array probesets (see explanation in main text).

**Figure L.** Comparison of GradientScanSurv and PrognoScan results for KRAS gene in LUAD datasets downloaded from PrognoScan with focus on one dataset GSE31210 by probeset 204010_s_at for survival type RFS (relapse-free survival) indicated by the red arrow in panel A. (A). Joined table of GradientScanSurv result that run on downloaded data from PrognoScan and direct result run from PrognoScan website for KRAS gene in the same LUAD (lung adenocarcinoma) datasets. The arrow indicated a dataset where GradientScanSurv called a significant association with GoodCountPval=0.016, but PrognoScan also made the call with CORRECTED.P.VALUE=0.048. (B). Screenshot of PrognoScan result’s report for dataset indicated by red arrow in panel A. (C). GradientScanSurv gene expression gradient-based logrank p-values plot with GoodCountPval at 0 (<0.001 by n=1000 permutations) for dataset indicated by red arrow in panel A. This result is consistent with TCGA Validation result in p-value (0.004). Also for this dataset, the plot showed higher expressors of KRAS (measured by probeset 214352_s_at) dies faster indicated by the brown diamonds along the top part of the plots, which is consistent with TCGA validation result on **Figure I**. This would be due to the location and design of array probesets (see explanation in main text).

**Figure M**. Comparison of GradientScanSurv and PrognoScan results for KRAS gene in LUAD datasets downloaded from PrognoScan with focus on one dataset GSE31210 by probeset 204009_s_at for survival type RFS (relapse-free survival) indicated by the red arrow in panel A. (A). Joined table of GradientScanSurv result that run on downloaded data from PrognoScan and direct result run from PrognoScan website for KRAS gene in the same LUAD (lung adenocarcinoma) datasets. The arrow indicated a dataset where GradientScanSurv called an insignificant association with GoodCountPval=0.344, but PrognoScan also insignificant with CORRECTED.P.VALUE=0.402. (B). Screenshot of PrognoScan result’s report for dataset indicated by red arrow in panel A. (C). GradientScanSurv gene expression gradient-based logrank p-values plot for dataset indicated by red arrow in panel A. Although overall GoodCountPval is not significant, but some cut-points did give significant logrank p-values and on those cut-points, higher expressors of KRAS (measured by probeset 204009_s_at) dies faster indicated by the brown diamonds along the top part of the plots, which is consistent with TCGA validation result on **Figure I**. This would be due to the location and design of array probesets (see explanation in main text).

**Figure N**. UCSC Genome Browser view of KRAS gene locus on probesets (204010_s_at, 204009_s_at, 214352_s_at) from array platform HG-U133_Plus2 that created the PrognoScan datasets (GSE31210 and Jacob-00182-HLM). The KRAS gene oriented from right to left and the array probesets (214352_s_at, 204009_s_at, 204010_s_at) are designed for corresponding regions of KRAS locus accordingly in that order. Due to good positive correlation between 214352s_at and 204009_s_at but not with 204010_s_at and the fact that 204010_s_at is at the far end of KRAS gene and the other two probesets are in the near end of the KRAS genes, it is possible that maybe 204010_s_at does not measure the direct transcript from KRAS but some feedback or residue transcript that behaved opposite to the transcripts measured by 204009_s_at and 214352_s_at. This may explain why we saw different directions of association relation between KRAS expression measured by these probesets vs survival outcome.

**Figure O.** Correlation matrix for probesets (including 204010_s_at, 204009_s_at, 214352_s_at) for KRAS gene in LUAD GSE31210 dataset downloaded from PrognoScan web site. These probesets are from array platform HG-U133_Plus2 that has been used for the GSE31210. total 204 patients.

| **Table B. Summary of the methods for each trial of tests with the top AUC comparing with other methods, from which Fig 4B is created** | | | |
| --- | --- | --- | --- |
| PrognoScan_TruthGeneLists | TCGA_Trials | TopAUCMethod | TopAUCMethodAUCs |
| GSE13213.Surv_AllMethods_Freq_2 | trial_1 | Adj.CoxPvalbyRanks | 0.674953 |
| GSE13213.Surv_AllMethods_Freq_3 | trial_1 | Adj.CoxPvalbyRanks | 0.687022 |
| GSE13213.Surv_AllMethods_Freq_4 | trial_1 | Adj.tertPvals | 0.681693 |
| GSE13213.Surv_AllMethods_Freq_5 | trial_1 | Adj.tertPvals | 0.624786 |
| GSE13213.Surv_AllMethods_Freq_6 | trial_1 | Adj.GoodCountPvals | 0.601506 |
| GSE13213.Surv_AllMethods_Freq_2 | trial_2 | Adj.CoxPvalbyRanks | 0.674953 |
| GSE13213.Surv_AllMethods_Freq_3 | trial_2 | Adj.CoxPvalbyRanks | 0.687022 |
| GSE13213.Surv_AllMethods_Freq_4 | trial_2 | Adj.tertPvals | 0.681693 |
| GSE13213.Surv_AllMethods_Freq_5 | trial_2 | Adj.tertPvals | 0.624786 |
| GSE13213.Surv_AllMethods_Freq_6 | trial_2 | Adj.COX_P_VALUE | 0.600208 |
| GSE13213.Surv_AllMethods_Freq_2 | trial_3 | Adj.CoxPvalbyRanks | 0.674953 |
| GSE13213.Surv_AllMethods_Freq_3 | trial_3 | Adj.CoxPvalbyRanks | 0.687022 |
| GSE13213.Surv_AllMethods_Freq_4 | trial_3 | Adj.tertPvals | 0.681693 |
| GSE13213.Surv_AllMethods_Freq_5 | trial_3 | Adj.tertPvals | 0.624786 |
| GSE13213.Surv_AllMethods_Freq_6 | trial_3 | Adj.COX_P_VALUE | 0.600208 |
| GSE13213.Surv_AllMethods_Freq_2 | trial_4 | Adj.CoxPvalbyRanks | 0.674953 |
| GSE13213.Surv_AllMethods_Freq_3 | trial_4 | Adj.CoxPvalbyRanks | 0.687022 |
| GSE13213.Surv_AllMethods_Freq_4 | trial_4 | Adj.tertPvals | 0.681693 |
| GSE13213.Surv_AllMethods_Freq_5 | trial_4 | Adj.tertPvals | 0.624786 |
| GSE13213.Surv_AllMethods_Freq_6 | trial_4 | Adj.COX_P_VALUE | 0.600208 |
| GSE13213.Surv_AllMethods_Freq_2 | trial_5 | Adj.CoxPvalbyRanks | 0.674953 |
| GSE13213.Surv_AllMethods_Freq_3 | trial_5 | Adj.CoxPvalbyRanks | 0.687022 |
| GSE13213.Surv_AllMethods_Freq_4 | trial_5 | Adj.tertPvals | 0.681693 |
| GSE13213.Surv_AllMethods_Freq_5 | trial_5 | Adj.tertPvals | 0.624786 |
| GSE13213.Surv_AllMethods_Freq_6 | trial_5 | Adj.COX_P_VALUE | 0.600208 |
| GSE13213.Surv_AllMethods_Freq_2 | trial_6 | Adj.CoxPvalbyRanks | 0.674953 |
| GSE13213.Surv_AllMethods_Freq_3 | trial_6 | Adj.CoxPvalbyRanks | 0.687022 |
| GSE13213.Surv_AllMethods_Freq_4 | trial_6 | Adj.tertPvals | 0.681693 |
| GSE13213.Surv_AllMethods_Freq_5 | trial_6 | Adj.tertPvals | 0.624786 |
| GSE13213.Surv_AllMethods_Freq_6 | trial_6 | Adj.COX_P_VALUE | 0.600208 |
| GSE13213.Surv_AllMethods_Freq_2 | trial_7 | Adj.CoxPvalbyRanks | 0.674953 |
| GSE13213.Surv_AllMethods_Freq_3 | trial_7 | Adj.CoxPvalbyRanks | 0.687022 |
| GSE13213.Surv_AllMethods_Freq_4 | trial_7 | Adj.tertPvals | 0.681693 |
| GSE13213.Surv_AllMethods_Freq_5 | trial_7 | Adj.tertPvals | 0.624786 |
| GSE13213.Surv_AllMethods_Freq_6 | trial_7 | Adj.GoodCountPvals | 0.603063 |
| GSE13213.Surv_AllMethods_Freq_2 | trial_8 | Adj.CoxPvalbyRanks | 0.674953 |
| GSE13213.Surv_AllMethods_Freq_3 | trial_8 | Adj.CoxPvalbyRanks | 0.687022 |
| GSE13213.Surv_AllMethods_Freq_4 | trial_8 | Adj.tertPvals | 0.681693 |
| GSE13213.Surv_AllMethods_Freq_5 | trial_8 | Adj.tertPvals | 0.624786 |
| GSE13213.Surv_AllMethods_Freq_6 | trial_8 | Adj.GoodCountPvals | 0.601246 |
| GSE13213.Surv_AllMethods_Freq_2 | trial_9 | Adj.CoxPvalbyRanks | 0.674953 |
| GSE13213.Surv_AllMethods_Freq_3 | trial_9 | Adj.CoxPvalbyRanks | 0.687022 |
| GSE13213.Surv_AllMethods_Freq_4 | trial_9 | Adj.tertPvals | 0.681693 |
| GSE13213.Surv_AllMethods_Freq_5 | trial_9 | Adj.tertPvals | 0.624786 |
| GSE13213.Surv_AllMethods_Freq_6 | trial_9 | Adj.COX_P_VALUE | 0.600208 |
| GSE13213.Surv_AllMethods_Freq_2 | trial_10 | Adj.CoxPvalbyRanks | 0.674953 |
| GSE13213.Surv_AllMethods_Freq_3 | trial_10 | Adj.CoxPvalbyRanks | 0.687022 |
| GSE13213.Surv_AllMethods_Freq_4 | trial_10 | Adj.tertPvals | 0.681693 |
| GSE13213.Surv_AllMethods_Freq_5 | trial_10 | Adj.tertPvals | 0.624786 |
| GSE13213.Surv_AllMethods_Freq_6 | trial_10 | Adj.COX_P_VALUE | 0.600208 |
| GSE13213.Surv_AllMethods_Freq_2 | trial_11 | Adj.CoxPvalbyRanks | 0.674953 |
| GSE13213.Surv_AllMethods_Freq_3 | trial_11 | Adj.CoxPvalbyRanks | 0.687022 |
| GSE13213.Surv_AllMethods_Freq_4 | trial_11 | Adj.tertPvals | 0.681693 |
| GSE13213.Surv_AllMethods_Freq_5 | trial_11 | Adj.tertPvals | 0.624786 |
| GSE13213.Surv_AllMethods_Freq_6 | trial_11 | Adj.GoodCountPvals | 0.601246 |
| GSE13213.Surv_AllMethods_Freq_2 | trial_12 | Adj.CoxPvalbyRanks | 0.674953 |
| GSE13213.Surv_AllMethods_Freq_3 | trial_12 | Adj.CoxPvalbyRanks | 0.687022 |
| GSE13213.Surv_AllMethods_Freq_4 | trial_12 | Adj.tertPvals | 0.681693 |
| GSE13213.Surv_AllMethods_Freq_5 | trial_12 | Adj.tertPvals | 0.624786 |
| GSE13213.Surv_AllMethods_Freq_6 | trial_12 | Adj.COX_P_VALUE | 0.600208 |
| GSE13213.Surv_AllMethods_Freq_2 | trial_13 | Adj.CoxPvalbyRanks | 0.674953 |
| GSE13213.Surv_AllMethods_Freq_3 | trial_13 | Adj.CoxPvalbyRanks | 0.687022 |
| GSE13213.Surv_AllMethods_Freq_4 | trial_13 | Adj.tertPvals | 0.681693 |
| GSE13213.Surv_AllMethods_Freq_5 | trial_13 | Adj.tertPvals | 0.624786 |
| GSE13213.Surv_AllMethods_Freq_6 | trial_13 | Adj.COX_P_VALUE | 0.600208 |
| GSE13213.Surv_AllMethods_Freq_2 | trial_14 | Adj.CoxPvalbyRanks | 0.674953 |
| GSE13213.Surv_AllMethods_Freq_3 | trial_14 | Adj.CoxPvalbyRanks | 0.687022 |
| GSE13213.Surv_AllMethods_Freq_4 | trial_14 | Adj.tertPvals | 0.681693 |
| GSE13213.Surv_AllMethods_Freq_5 | trial_14 | Adj.tertPvals | 0.624786 |
| GSE13213.Surv_AllMethods_Freq_6 | trial_14 | Adj.COX_P_VALUE | 0.600208 |
| GSE13213.Surv_AllMethods_Freq_2 | trial_15 | Adj.CoxPvalbyRanks | 0.674953 |
| GSE13213.Surv_AllMethods_Freq_3 | trial_15 | Adj.CoxPvalbyRanks | 0.687022 |
| GSE13213.Surv_AllMethods_Freq_4 | trial_15 | Adj.tertPvals | 0.681693 |
| GSE13213.Surv_AllMethods_Freq_5 | trial_15 | Adj.tertPvals | 0.624786 |
| GSE13213.Surv_AllMethods_Freq_6 | trial_15 | Adj.COX_P_VALUE | 0.600208 |
| GSE13213.Surv_AllMethods_Freq_2 | trial_16 | Adj.CoxPvalbyRanks | 0.674953 |
| GSE13213.Surv_AllMethods_Freq_3 | trial_16 | Adj.CoxPvalbyRanks | 0.687022 |
| GSE13213.Surv_AllMethods_Freq_4 | trial_16 | Adj.tertPvals | 0.681693 |
| GSE13213.Surv_AllMethods_Freq_5 | trial_16 | Adj.tertPvals | 0.624786 |
| GSE13213.Surv_AllMethods_Freq_6 | trial_16 | Adj.GoodCountPvals | 0.602285 |
| GSE13213.Surv_AllMethods_Freq_2 | trial_17 | Adj.CoxPvalbyRanks | 0.674953 |
| GSE13213.Surv_AllMethods_Freq_3 | trial_17 | Adj.CoxPvalbyRanks | 0.687022 |
| GSE13213.Surv_AllMethods_Freq_4 | trial_17 | Adj.tertPvals | 0.681693 |
| GSE13213.Surv_AllMethods_Freq_5 | trial_17 | Adj.tertPvals | 0.624786 |
| GSE13213.Surv_AllMethods_Freq_6 | trial_17 | Adj.COX_P_VALUE | 0.600208 |
| GSE13213.Surv_AllMethods_Freq_2 | trial_18 | Adj.CoxPvalbyRanks | 0.674953 |
| GSE13213.Surv_AllMethods_Freq_3 | trial_18 | Adj.CoxPvalbyRanks | 0.687022 |
| GSE13213.Surv_AllMethods_Freq_4 | trial_18 | Adj.tertPvals | 0.681693 |
| GSE13213.Surv_AllMethods_Freq_5 | trial_18 | Adj.tertPvals | 0.624786 |
| GSE13213.Surv_AllMethods_Freq_6 | trial_18 | Adj.COX_P_VALUE | 0.600208 |
| GSE13213.Surv_AllMethods_Freq_2 | trial_19 | Adj.CoxPvalbyRanks | 0.674953 |
| GSE13213.Surv_AllMethods_Freq_3 | trial_19 | Adj.CoxPvalbyRanks | 0.687022 |
| GSE13213.Surv_AllMethods_Freq_4 | trial_19 | Adj.tertPvals | 0.681693 |
| GSE13213.Surv_AllMethods_Freq_5 | trial_19 | Adj.tertPvals | 0.624786 |
| GSE13213.Surv_AllMethods_Freq_6 | trial_19 | Adj.GoodCountPvals | 0.602025 |
| GSE13213.Surv_AllMethods_Freq_2 | trial_20 | Adj.CoxPvalbyRanks | 0.674953 |
| GSE13213.Surv_AllMethods_Freq_3 | trial_20 | Adj.CoxPvalbyRanks | 0.687022 |
| GSE13213.Surv_AllMethods_Freq_4 | trial_20 | Adj.tertPvals | 0.681693 |
| GSE13213.Surv_AllMethods_Freq_5 | trial_20 | Adj.tertPvals | 0.624786 |
| GSE13213.Surv_AllMethods_Freq_6 | trial_20 | Adj.COX_P_VALUE | 0.600208 |
| GSE13213.Surv_AllMethods_Freq_2 | trial_21 | Adj.CoxPvalbyRanks | 0.674953 |
| GSE13213.Surv_AllMethods_Freq_3 | trial_21 | Adj.CoxPvalbyRanks | 0.687022 |
| GSE13213.Surv_AllMethods_Freq_4 | trial_21 | Adj.tertPvals | 0.681693 |
| GSE13213.Surv_AllMethods_Freq_5 | trial_21 | Adj.tertPvals | 0.624786 |
| GSE13213.Surv_AllMethods_Freq_6 | trial_21 | Adj.COX_P_VALUE | 0.600208 |
| GSE13213.Surv_AllMethods_Freq_2 | trial_22 | Adj.CoxPvalbyRanks | 0.674953 |
| GSE13213.Surv_AllMethods_Freq_3 | trial_22 | Adj.CoxPvalbyRanks | 0.687022 |
| GSE13213.Surv_AllMethods_Freq_4 | trial_22 | Adj.tertPvals | 0.681693 |
| GSE13213.Surv_AllMethods_Freq_5 | trial_22 | Adj.tertPvals | 0.624786 |
| GSE13213.Surv_AllMethods_Freq_6 | trial_22 | Adj.COX_P_VALUE | 0.600208 |
| GSE13213.Surv_AllMethods_Freq_2 | trial_23 | Adj.CoxPvalbyRanks | 0.674953 |
| GSE13213.Surv_AllMethods_Freq_3 | trial_23 | Adj.CoxPvalbyRanks | 0.687022 |
| GSE13213.Surv_AllMethods_Freq_4 | trial_23 | Adj.tertPvals | 0.681693 |
| GSE13213.Surv_AllMethods_Freq_5 | trial_23 | Adj.tertPvals | 0.624786 |
| GSE13213.Surv_AllMethods_Freq_6 | trial_23 | Adj.GoodCountPvals | 0.600987 |
| GSE13213.Surv_AllMethods_Freq_2 | trial_24 | Adj.CoxPvalbyRanks | 0.674953 |
| GSE13213.Surv_AllMethods_Freq_3 | trial_24 | Adj.CoxPvalbyRanks | 0.687022 |
| GSE13213.Surv_AllMethods_Freq_4 | trial_24 | Adj.tertPvals | 0.681693 |
| GSE13213.Surv_AllMethods_Freq_5 | trial_24 | Adj.tertPvals | 0.624786 |
| GSE13213.Surv_AllMethods_Freq_6 | trial_24 | Adj.GoodCountPvals | 0.602025 |
| GSE13213.Surv_AllMethods_Freq_2 | trial_25 | Adj.CoxPvalbyRanks | 0.674953 |
| GSE13213.Surv_AllMethods_Freq_3 | trial_25 | Adj.CoxPvalbyRanks | 0.687022 |
| GSE13213.Surv_AllMethods_Freq_4 | trial_25 | Adj.tertPvals | 0.681693 |
| GSE13213.Surv_AllMethods_Freq_5 | trial_25 | Adj.tertPvals | 0.624786 |
| GSE13213.Surv_AllMethods_Freq_6 | trial_25 | Adj.COX_P_VALUE | 0.600208 |
| GSE13213.Surv_AllMethods_Freq_2 | trial_26 | Adj.CoxPvalbyRanks | 0.674953 |
| GSE13213.Surv_AllMethods_Freq_3 | trial_26 | Adj.CoxPvalbyRanks | 0.687022 |
| GSE13213.Surv_AllMethods_Freq_4 | trial_26 | Adj.tertPvals | 0.681693 |
| GSE13213.Surv_AllMethods_Freq_5 | trial_26 | Adj.tertPvals | 0.624786 |
| GSE13213.Surv_AllMethods_Freq_6 | trial_26 | Adj.COX_P_VALUE | 0.600208 |
| GSE13213.Surv_AllMethods_Freq_2 | trial_27 | Adj.CoxPvalbyRanks | 0.674953 |
| GSE13213.Surv_AllMethods_Freq_3 | trial_27 | Adj.CoxPvalbyRanks | 0.687022 |
| GSE13213.Surv_AllMethods_Freq_4 | trial_27 | Adj.tertPvals | 0.681693 |
| GSE13213.Surv_AllMethods_Freq_5 | trial_27 | Adj.tertPvals | 0.624786 |
| GSE13213.Surv_AllMethods_Freq_6 | trial_27 | Adj.GoodCountPvals | 0.603063 |
| GSE13213.Surv_AllMethods_Freq_2 | trial_28 | Adj.CoxPvalbyRanks | 0.674953 |
| GSE13213.Surv_AllMethods_Freq_3 | trial_28 | Adj.CoxPvalbyRanks | 0.687022 |
| GSE13213.Surv_AllMethods_Freq_4 | trial_28 | Adj.tertPvals | 0.681693 |
| GSE13213.Surv_AllMethods_Freq_5 | trial_28 | Adj.tertPvals | 0.624786 |
| GSE13213.Surv_AllMethods_Freq_6 | trial_28 | Adj.COX_P_VALUE | 0.600208 |
| GSE13213.Surv_AllMethods_Freq_2 | trial_29 | Adj.CoxPvalbyRanks | 0.674953 |
| GSE13213.Surv_AllMethods_Freq_3 | trial_29 | Adj.CoxPvalbyRanks | 0.687022 |
| GSE13213.Surv_AllMethods_Freq_4 | trial_29 | Adj.tertPvals | 0.681693 |
| GSE13213.Surv_AllMethods_Freq_5 | trial_29 | Adj.tertPvals | 0.624786 |
| GSE13213.Surv_AllMethods_Freq_6 | trial_29 | Adj.COX_P_VALUE | 0.600208 |
| GSE13213.Surv_AllMethods_Freq_2 | trial_30 | Adj.CoxPvalbyRanks | 0.674953 |
| GSE13213.Surv_AllMethods_Freq_3 | trial_30 | Adj.CoxPvalbyRanks | 0.687022 |
| GSE13213.Surv_AllMethods_Freq_4 | trial_30 | Adj.tertPvals | 0.681693 |
| GSE13213.Surv_AllMethods_Freq_5 | trial_30 | Adj.tertPvals | 0.624786 |
| GSE13213.Surv_AllMethods_Freq_6 | trial_30 | Adj.COX_P_VALUE | 0.600208 |
| GSE13213.Surv_AllMethods_Freq_2 | trial_31 | Adj.CoxPvalbyRanks | 0.674953 |
| GSE13213.Surv_AllMethods_Freq_3 | trial_31 | Adj.CoxPvalbyRanks | 0.687022 |
| GSE13213.Surv_AllMethods_Freq_4 | trial_31 | Adj.tertPvals | 0.681693 |
| GSE13213.Surv_AllMethods_Freq_5 | trial_31 | Adj.tertPvals | 0.624786 |
| GSE13213.Surv_AllMethods_Freq_6 | trial_31 | Adj.COX_P_VALUE | 0.600208 |
| GSE13213.Surv_AllMethods_Freq_2 | trial_32 | Adj.CoxPvalbyRanks | 0.674953 |
| GSE13213.Surv_AllMethods_Freq_3 | trial_32 | Adj.CoxPvalbyRanks | 0.687022 |
| GSE13213.Surv_AllMethods_Freq_4 | trial_32 | Adj.tertPvals | 0.681693 |
| GSE13213.Surv_AllMethods_Freq_5 | trial_32 | Adj.tertPvals | 0.624786 |
| GSE13213.Surv_AllMethods_Freq_6 | trial_32 | Adj.COX_P_VALUE | 0.600208 |
| GSE13213.Surv_AllMethods_Freq_2 | trial_33 | Adj.CoxPvalbyRanks | 0.674953 |
| GSE13213.Surv_AllMethods_Freq_3 | trial_33 | Adj.CoxPvalbyRanks | 0.687022 |
| GSE13213.Surv_AllMethods_Freq_4 | trial_33 | Adj.tertPvals | 0.681693 |
| GSE13213.Surv_AllMethods_Freq_5 | trial_33 | Adj.tertPvals | 0.624786 |
| GSE13213.Surv_AllMethods_Freq_6 | trial_33 | Adj.COX_P_VALUE | 0.600208 |
| GSE13213.Surv_AllMethods_Freq_2 | trial_34 | Adj.CoxPvalbyRanks | 0.674953 |
| GSE13213.Surv_AllMethods_Freq_3 | trial_34 | Adj.CoxPvalbyRanks | 0.687022 |
| GSE13213.Surv_AllMethods_Freq_4 | trial_34 | Adj.tertPvals | 0.681693 |
| GSE13213.Surv_AllMethods_Freq_5 | trial_34 | Adj.tertPvals | 0.624786 |
| GSE13213.Surv_AllMethods_Freq_6 | trial_34 | Adj.COX_P_VALUE | 0.600208 |
| GSE13213.Surv_AllMethods_Freq_2 | trial_35 | Adj.CoxPvalbyRanks | 0.674953 |
| GSE13213.Surv_AllMethods_Freq_3 | trial_35 | Adj.CoxPvalbyRanks | 0.687022 |
| GSE13213.Surv_AllMethods_Freq_4 | trial_35 | Adj.tertPvals | 0.681693 |
| GSE13213.Surv_AllMethods_Freq_5 | trial_35 | Adj.tertPvals | 0.624786 |
| GSE13213.Surv_AllMethods_Freq_6 | trial_35 | Adj.COX_P_VALUE | 0.600208 |
| GSE13213.Surv_AllMethods_Freq_2 | trial_36 | Adj.CoxPvalbyRanks | 0.674953 |
| GSE13213.Surv_AllMethods_Freq_3 | trial_36 | Adj.CoxPvalbyRanks | 0.687022 |
| GSE13213.Surv_AllMethods_Freq_4 | trial_36 | Adj.tertPvals | 0.681693 |
| GSE13213.Surv_AllMethods_Freq_5 | trial_36 | Adj.tertPvals | 0.624786 |
| GSE13213.Surv_AllMethods_Freq_6 | trial_36 | Adj.COX_P_VALUE | 0.600208 |
| GSE13213.Surv_AllMethods_Freq_2 | trial_37 | Adj.CoxPvalbyRanks | 0.674953 |
| GSE13213.Surv_AllMethods_Freq_3 | trial_37 | Adj.CoxPvalbyRanks | 0.687022 |
| GSE13213.Surv_AllMethods_Freq_4 | trial_37 | Adj.tertPvals | 0.681693 |
| GSE13213.Surv_AllMethods_Freq_5 | trial_37 | Adj.tertPvals | 0.624786 |
| GSE13213.Surv_AllMethods_Freq_6 | trial_37 | Adj.COX_P_VALUE | 0.600208 |
| GSE13213.Surv_AllMethods_Freq_2 | trial_38 | Adj.CoxPvalbyRanks | 0.674953 |
| GSE13213.Surv_AllMethods_Freq_3 | trial_38 | Adj.CoxPvalbyRanks | 0.687022 |
| GSE13213.Surv_AllMethods_Freq_4 | trial_38 | Adj.tertPvals | 0.681693 |
| GSE13213.Surv_AllMethods_Freq_5 | trial_38 | Adj.tertPvals | 0.624786 |
| GSE13213.Surv_AllMethods_Freq_6 | trial_38 | Adj.COX_P_VALUE | 0.600208 |
| GSE13213.Surv_AllMethods_Freq_2 | trial_39 | Adj.CoxPvalbyRanks | 0.674953 |
| GSE13213.Surv_AllMethods_Freq_3 | trial_39 | Adj.CoxPvalbyRanks | 0.687022 |
| GSE13213.Surv_AllMethods_Freq_4 | trial_39 | Adj.tertPvals | 0.681693 |
| GSE13213.Surv_AllMethods_Freq_5 | trial_39 | Adj.tertPvals | 0.624786 |
| GSE13213.Surv_AllMethods_Freq_6 | trial_39 | Adj.GoodCountPvals | 0.6054 |
| GSE13213.Surv_AllMethods_Freq_2 | trial_40 | Adj.CoxPvalbyRanks | 0.674953 |
| GSE13213.Surv_AllMethods_Freq_3 | trial_40 | Adj.CoxPvalbyRanks | 0.687022 |
| GSE13213.Surv_AllMethods_Freq_4 | trial_40 | Adj.tertPvals | 0.681693 |
| GSE13213.Surv_AllMethods_Freq_5 | trial_40 | Adj.tertPvals | 0.624786 |
| GSE13213.Surv_AllMethods_Freq_6 | trial_40 | Adj.GoodCountPvals | 0.606179 |
| GSE13213.Surv_AllMethods_Freq_2 | trial_41 | Adj.CoxPvalbyRanks | 0.674953 |
| GSE13213.Surv_AllMethods_Freq_3 | trial_41 | Adj.CoxPvalbyRanks | 0.687022 |
| GSE13213.Surv_AllMethods_Freq_4 | trial_41 | Adj.tertPvals | 0.681693 |
| GSE13213.Surv_AllMethods_Freq_5 | trial_41 | Adj.tertPvals | 0.624786 |
| GSE13213.Surv_AllMethods_Freq_6 | trial_41 | Adj.GoodCountPvals | 0.601765 |
| GSE13213.Surv_AllMethods_Freq_2 | trial_42 | Adj.CoxPvalbyRanks | 0.674953 |
| GSE13213.Surv_AllMethods_Freq_3 | trial_42 | Adj.CoxPvalbyRanks | 0.687022 |
| GSE13213.Surv_AllMethods_Freq_4 | trial_42 | Adj.tertPvals | 0.681693 |
| GSE13213.Surv_AllMethods_Freq_5 | trial_42 | Adj.tertPvals | 0.624786 |
| GSE13213.Surv_AllMethods_Freq_6 | trial_42 | Adj.GoodCountPvals:Adj.COX_P_VALUE | 0.600208 |
| GSE13213.Surv_AllMethods_Freq_2 | trial_43 | Adj.CoxPvalbyRanks | 0.674953 |
| GSE13213.Surv_AllMethods_Freq_3 | trial_43 | Adj.CoxPvalbyRanks | 0.687022 |
| GSE13213.Surv_AllMethods_Freq_4 | trial_43 | Adj.tertPvals | 0.681693 |
| GSE13213.Surv_AllMethods_Freq_5 | trial_43 | Adj.tertPvals | 0.624786 |
| GSE13213.Surv_AllMethods_Freq_6 | trial_43 | Adj.COX_P_VALUE | 0.600208 |
| GSE13213.Surv_AllMethods_Freq_2 | trial_44 | Adj.CoxPvalbyRanks | 0.674953 |
| GSE13213.Surv_AllMethods_Freq_3 | trial_44 | Adj.CoxPvalbyRanks | 0.687022 |
| GSE13213.Surv_AllMethods_Freq_4 | trial_44 | Adj.tertPvals | 0.681693 |
| GSE13213.Surv_AllMethods_Freq_5 | trial_44 | Adj.tertPvals | 0.624786 |
| GSE13213.Surv_AllMethods_Freq_6 | trial_44 | Adj.COX_P_VALUE | 0.600208 |
| GSE13213.Surv_AllMethods_Freq_2 | trial_45 | Adj.CoxPvalbyRanks | 0.674953 |
| GSE13213.Surv_AllMethods_Freq_3 | trial_45 | Adj.CoxPvalbyRanks | 0.687022 |
| GSE13213.Surv_AllMethods_Freq_4 | trial_45 | Adj.tertPvals | 0.681693 |
| GSE13213.Surv_AllMethods_Freq_5 | trial_45 | Adj.tertPvals | 0.624786 |
| GSE13213.Surv_AllMethods_Freq_6 | trial_45 | Adj.GoodCountPvals | 0.601765 |
| GSE13213.Surv_AllMethods_Freq_2 | trial_46 | Adj.CoxPvalbyRanks | 0.674953 |
| GSE13213.Surv_AllMethods_Freq_3 | trial_46 | Adj.CoxPvalbyRanks | 0.687022 |
| GSE13213.Surv_AllMethods_Freq_4 | trial_46 | Adj.tertPvals | 0.681693 |
| GSE13213.Surv_AllMethods_Freq_5 | trial_46 | Adj.tertPvals | 0.624786 |
| GSE13213.Surv_AllMethods_Freq_6 | trial_46 | Adj.GoodCountPvals | 0.600467 |
| GSE13213.Surv_AllMethods_Freq_2 | trial_47 | Adj.CoxPvalbyRanks | 0.674953 |
| GSE13213.Surv_AllMethods_Freq_3 | trial_47 | Adj.CoxPvalbyRanks | 0.687022 |
| GSE13213.Surv_AllMethods_Freq_4 | trial_47 | Adj.tertPvals | 0.681693 |
| GSE13213.Surv_AllMethods_Freq_5 | trial_47 | Adj.tertPvals | 0.624786 |
| GSE13213.Surv_AllMethods_Freq_6 | trial_47 | Adj.COX_P_VALUE | 0.600208 |
| GSE13213.Surv_AllMethods_Freq_2 | trial_48 | Adj.CoxPvalbyRanks | 0.674953 |
| GSE13213.Surv_AllMethods_Freq_3 | trial_48 | Adj.CoxPvalbyRanks | 0.687022 |
| GSE13213.Surv_AllMethods_Freq_4 | trial_48 | Adj.tertPvals | 0.681693 |
| GSE13213.Surv_AllMethods_Freq_5 | trial_48 | Adj.tertPvals | 0.624786 |
| GSE13213.Surv_AllMethods_Freq_6 | trial_48 | Adj.GoodCountPvals:Adj.COX_P_VALUE | 0.600208 |
| GSE13213.Surv_AllMethods_Freq_2 | trial_49 | Adj.CoxPvalbyRanks | 0.674953 |
| GSE13213.Surv_AllMethods_Freq_3 | trial_49 | Adj.CoxPvalbyRanks | 0.687022 |
| GSE13213.Surv_AllMethods_Freq_4 | trial_49 | Adj.tertPvals | 0.681693 |
| GSE13213.Surv_AllMethods_Freq_5 | trial_49 | Adj.tertPvals | 0.624786 |
| GSE13213.Surv_AllMethods_Freq_6 | trial_49 | Adj.COX_P_VALUE | 0.600208 |
| GSE13213.Surv_AllMethods_Freq_2 | trial_50 | Adj.CoxPvalbyRanks | 0.674953 |
| GSE13213.Surv_AllMethods_Freq_3 | trial_50 | Adj.CoxPvalbyRanks | 0.687022 |
| GSE13213.Surv_AllMethods_Freq_4 | trial_50 | Adj.tertPvals | 0.681693 |
| GSE13213.Surv_AllMethods_Freq_5 | trial_50 | Adj.tertPvals | 0.624786 |
| GSE13213.Surv_AllMethods_Freq_6 | trial_50 | Adj.GoodCountPvals | 0.603063 |
| GSE13213.Surv_AllMethods_Freq_2 | trial_51 | Adj.CoxPvalbyRanks | 0.674953 |
| GSE13213.Surv_AllMethods_Freq_3 | trial_51 | Adj.CoxPvalbyRanks | 0.687022 |
| GSE13213.Surv_AllMethods_Freq_4 | trial_51 | Adj.tertPvals | 0.681693 |
| GSE13213.Surv_AllMethods_Freq_5 | trial_51 | Adj.tertPvals | 0.624786 |
| GSE13213.Surv_AllMethods_Freq_6 | trial_51 | Adj.COX_P_VALUE | 0.600208 |
| GSE13213.Surv_AllMethods_Freq_2 | trial_52 | Adj.CoxPvalbyRanks | 0.674953 |
| GSE13213.Surv_AllMethods_Freq_3 | trial_52 | Adj.CoxPvalbyRanks | 0.687022 |
| GSE13213.Surv_AllMethods_Freq_4 | trial_52 | Adj.tertPvals | 0.681693 |
| GSE13213.Surv_AllMethods_Freq_5 | trial_52 | Adj.tertPvals | 0.624786 |
| GSE13213.Surv_AllMethods_Freq_6 | trial_52 | Adj.GoodCountPvals | 0.601506 |
| GSE13213.Surv_AllMethods_Freq_2 | trial_53 | Adj.CoxPvalbyRanks | 0.674953 |
| GSE13213.Surv_AllMethods_Freq_3 | trial_53 | Adj.CoxPvalbyRanks | 0.687022 |
| GSE13213.Surv_AllMethods_Freq_4 | trial_53 | Adj.tertPvals | 0.681693 |
| GSE13213.Surv_AllMethods_Freq_5 | trial_53 | Adj.tertPvals | 0.624786 |
| GSE13213.Surv_AllMethods_Freq_6 | trial_53 | Adj.COX_P_VALUE | 0.600208 |
| GSE13213.Surv_AllMethods_Freq_2 | trial_54 | Adj.CoxPvalbyRanks | 0.674953 |
| GSE13213.Surv_AllMethods_Freq_3 | trial_54 | Adj.CoxPvalbyRanks | 0.687022 |
| GSE13213.Surv_AllMethods_Freq_4 | trial_54 | Adj.tertPvals | 0.681693 |
| GSE13213.Surv_AllMethods_Freq_5 | trial_54 | Adj.tertPvals | 0.624786 |
| GSE13213.Surv_AllMethods_Freq_6 | trial_54 | Adj.GoodCountPvals | 0.607477 |
| GSE13213.Surv_AllMethods_Freq_2 | trial_55 | Adj.CoxPvalbyRanks | 0.674953 |
| GSE13213.Surv_AllMethods_Freq_3 | trial_55 | Adj.CoxPvalbyRanks | 0.687022 |
| GSE13213.Surv_AllMethods_Freq_4 | trial_55 | Adj.tertPvals | 0.681693 |
| GSE13213.Surv_AllMethods_Freq_5 | trial_55 | Adj.tertPvals | 0.624786 |
| GSE13213.Surv_AllMethods_Freq_6 | trial_55 | Adj.GoodCountPvals | 0.603063 |
| GSE13213.Surv_AllMethods_Freq_2 | trial_56 | Adj.CoxPvalbyRanks | 0.674953 |
| GSE13213.Surv_AllMethods_Freq_3 | trial_56 | Adj.CoxPvalbyRanks | 0.687022 |
| GSE13213.Surv_AllMethods_Freq_4 | trial_56 | Adj.tertPvals | 0.681693 |
| GSE13213.Surv_AllMethods_Freq_5 | trial_56 | Adj.tertPvals | 0.624786 |
| GSE13213.Surv_AllMethods_Freq_6 | trial_56 | Adj.COX_P_VALUE | 0.600208 |
| GSE13213.Surv_AllMethods_Freq_2 | trial_57 | Adj.CoxPvalbyRanks | 0.674953 |
| GSE13213.Surv_AllMethods_Freq_3 | trial_57 | Adj.CoxPvalbyRanks | 0.687022 |
| GSE13213.Surv_AllMethods_Freq_4 | trial_57 | Adj.tertPvals | 0.681693 |
| GSE13213.Surv_AllMethods_Freq_5 | trial_57 | Adj.tertPvals | 0.624786 |
| GSE13213.Surv_AllMethods_Freq_6 | trial_57 | Adj.GoodCountPvals | 0.604102 |
| GSE13213.Surv_AllMethods_Freq_2 | trial_58 | Adj.CoxPvalbyRanks | 0.674953 |
| GSE13213.Surv_AllMethods_Freq_3 | trial_58 | Adj.CoxPvalbyRanks | 0.687022 |
| GSE13213.Surv_AllMethods_Freq_4 | trial_58 | Adj.tertPvals | 0.681693 |
| GSE13213.Surv_AllMethods_Freq_5 | trial_58 | Adj.tertPvals | 0.624786 |
| GSE13213.Surv_AllMethods_Freq_6 | trial_58 | Adj.COX_P_VALUE | 0.600208 |
| GSE13213.Surv_AllMethods_Freq_2 | trial_59 | Adj.CoxPvalbyRanks | 0.674953 |
| GSE13213.Surv_AllMethods_Freq_3 | trial_59 | Adj.CoxPvalbyRanks | 0.687022 |
| GSE13213.Surv_AllMethods_Freq_4 | trial_59 | Adj.tertPvals | 0.681693 |
| GSE13213.Surv_AllMethods_Freq_5 | trial_59 | Adj.tertPvals | 0.624786 |
| GSE13213.Surv_AllMethods_Freq_6 | trial_59 | Adj.GoodCountPvals | 0.602025 |
| GSE13213.Surv_AllMethods_Freq_2 | trial_60 | Adj.CoxPvalbyRanks | 0.674953 |
| GSE13213.Surv_AllMethods_Freq_3 | trial_60 | Adj.CoxPvalbyRanks | 0.687022 |
| GSE13213.Surv_AllMethods_Freq_4 | trial_60 | Adj.tertPvals | 0.681693 |
| GSE13213.Surv_AllMethods_Freq_5 | trial_60 | Adj.tertPvals | 0.624786 |
| GSE13213.Surv_AllMethods_Freq_6 | trial_60 | Adj.COX_P_VALUE | 0.600208 |
| GSE13213.Surv_AllMethods_Freq_2 | trial_61 | Adj.CoxPvalbyRanks | 0.674953 |
| GSE13213.Surv_AllMethods_Freq_3 | trial_61 | Adj.CoxPvalbyRanks | 0.687022 |
| GSE13213.Surv_AllMethods_Freq_4 | trial_61 | Adj.tertPvals | 0.681693 |
| GSE13213.Surv_AllMethods_Freq_5 | trial_61 | Adj.tertPvals | 0.624786 |
| GSE13213.Surv_AllMethods_Freq_6 | trial_61 | Adj.COX_P_VALUE | 0.600208 |
| GSE13213.Surv_AllMethods_Freq_2 | trial_62 | Adj.CoxPvalbyRanks | 0.674953 |
| GSE13213.Surv_AllMethods_Freq_3 | trial_62 | Adj.CoxPvalbyRanks | 0.687022 |
| GSE13213.Surv_AllMethods_Freq_4 | trial_62 | Adj.tertPvals | 0.681693 |
| GSE13213.Surv_AllMethods_Freq_5 | trial_62 | Adj.tertPvals | 0.624786 |
| GSE13213.Surv_AllMethods_Freq_6 | trial_62 | Adj.COX_P_VALUE | 0.600208 |
| GSE13213.Surv_AllMethods_Freq_2 | trial_63 | Adj.CoxPvalbyRanks | 0.674953 |
| GSE13213.Surv_AllMethods_Freq_3 | trial_63 | Adj.CoxPvalbyRanks | 0.687022 |
| GSE13213.Surv_AllMethods_Freq_4 | trial_63 | Adj.tertPvals | 0.681693 |
| GSE13213.Surv_AllMethods_Freq_5 | trial_63 | Adj.tertPvals | 0.624786 |
| GSE13213.Surv_AllMethods_Freq_6 | trial_63 | Adj.COX_P_VALUE | 0.600208 |
| GSE13213.Surv_AllMethods_Freq_2 | trial_64 | Adj.CoxPvalbyRanks | 0.674953 |
| GSE13213.Surv_AllMethods_Freq_3 | trial_64 | Adj.CoxPvalbyRanks | 0.687022 |
| GSE13213.Surv_AllMethods_Freq_4 | trial_64 | Adj.tertPvals | 0.681693 |
| GSE13213.Surv_AllMethods_Freq_5 | trial_64 | Adj.tertPvals | 0.624786 |
| GSE13213.Surv_AllMethods_Freq_6 | trial_64 | Adj.COX_P_VALUE | 0.600208 |
| GSE13213.Surv_AllMethods_Freq_2 | trial_65 | Adj.CoxPvalbyRanks | 0.674953 |
| GSE13213.Surv_AllMethods_Freq_3 | trial_65 | Adj.CoxPvalbyRanks | 0.687022 |
| GSE13213.Surv_AllMethods_Freq_4 | trial_65 | Adj.tertPvals | 0.681693 |
| GSE13213.Surv_AllMethods_Freq_5 | trial_65 | Adj.tertPvals | 0.624786 |
| GSE13213.Surv_AllMethods_Freq_6 | trial_65 | Adj.COX_P_VALUE | 0.600208 |
| GSE13213.Surv_AllMethods_Freq_2 | trial_66 | Adj.CoxPvalbyRanks | 0.674953 |
| GSE13213.Surv_AllMethods_Freq_3 | trial_66 | Adj.CoxPvalbyRanks | 0.687022 |
| GSE13213.Surv_AllMethods_Freq_4 | trial_66 | Adj.tertPvals | 0.681693 |
| GSE13213.Surv_AllMethods_Freq_5 | trial_66 | Adj.tertPvals | 0.624786 |
| GSE13213.Surv_AllMethods_Freq_6 | trial_66 | Adj.COX_P_VALUE | 0.600208 |
| GSE13213.Surv_AllMethods_Freq_2 | trial_67 | Adj.CoxPvalbyRanks | 0.674953 |
| GSE13213.Surv_AllMethods_Freq_3 | trial_67 | Adj.CoxPvalbyRanks | 0.687022 |
| GSE13213.Surv_AllMethods_Freq_4 | trial_67 | Adj.tertPvals | 0.681693 |
| GSE13213.Surv_AllMethods_Freq_5 | trial_67 | Adj.tertPvals | 0.624786 |
| GSE13213.Surv_AllMethods_Freq_6 | trial_67 | Adj.COX_P_VALUE | 0.600208 |
| GSE13213.Surv_AllMethods_Freq_2 | trial_68 | Adj.CoxPvalbyRanks | 0.674953 |
| GSE13213.Surv_AllMethods_Freq_3 | trial_68 | Adj.CoxPvalbyRanks | 0.687022 |
| GSE13213.Surv_AllMethods_Freq_4 | trial_68 | Adj.tertPvals | 0.681693 |
| GSE13213.Surv_AllMethods_Freq_5 | trial_68 | Adj.tertPvals | 0.624786 |
| GSE13213.Surv_AllMethods_Freq_6 | trial_68 | Adj.COX_P_VALUE | 0.600208 |
| GSE13213.Surv_AllMethods_Freq_2 | trial_69 | Adj.CoxPvalbyRanks | 0.674953 |
| GSE13213.Surv_AllMethods_Freq_3 | trial_69 | Adj.CoxPvalbyRanks | 0.687022 |
| GSE13213.Surv_AllMethods_Freq_4 | trial_69 | Adj.tertPvals | 0.681693 |
| GSE13213.Surv_AllMethods_Freq_5 | trial_69 | Adj.tertPvals | 0.624786 |
| GSE13213.Surv_AllMethods_Freq_6 | trial_69 | Adj.COX_P_VALUE | 0.600208 |
| GSE13213.Surv_AllMethods_Freq_2 | trial_70 | Adj.CoxPvalbyRanks | 0.674953 |
| GSE13213.Surv_AllMethods_Freq_3 | trial_70 | Adj.CoxPvalbyRanks | 0.687022 |
| GSE13213.Surv_AllMethods_Freq_4 | trial_70 | Adj.tertPvals | 0.681693 |
| GSE13213.Surv_AllMethods_Freq_5 | trial_70 | Adj.tertPvals | 0.624786 |
| GSE13213.Surv_AllMethods_Freq_6 | trial_70 | Adj.COX_P_VALUE | 0.600208 |
| GSE13213.Surv_AllMethods_Freq_2 | trial_71 | Adj.CoxPvalbyRanks | 0.674953 |
| GSE13213.Surv_AllMethods_Freq_3 | trial_71 | Adj.CoxPvalbyRanks | 0.687022 |
| GSE13213.Surv_AllMethods_Freq_4 | trial_71 | Adj.tertPvals | 0.681693 |
| GSE13213.Surv_AllMethods_Freq_5 | trial_71 | Adj.tertPvals | 0.624786 |
| GSE13213.Surv_AllMethods_Freq_6 | trial_71 | Adj.COX_P_VALUE | 0.600208 |
| GSE13213.Surv_AllMethods_Freq_2 | trial_72 | Adj.CoxPvalbyRanks | 0.674953 |
| GSE13213.Surv_AllMethods_Freq_3 | trial_72 | Adj.CoxPvalbyRanks | 0.687022 |
| GSE13213.Surv_AllMethods_Freq_4 | trial_72 | Adj.tertPvals | 0.681693 |
| GSE13213.Surv_AllMethods_Freq_5 | trial_72 | Adj.tertPvals | 0.624786 |
| GSE13213.Surv_AllMethods_Freq_6 | trial_72 | Adj.COX_P_VALUE | 0.600208 |
| GSE13213.Surv_AllMethods_Freq_2 | trial_73 | Adj.CoxPvalbyRanks | 0.674953 |
| GSE13213.Surv_AllMethods_Freq_3 | trial_73 | Adj.CoxPvalbyRanks | 0.687022 |
| GSE13213.Surv_AllMethods_Freq_4 | trial_73 | Adj.tertPvals | 0.681693 |
| GSE13213.Surv_AllMethods_Freq_5 | trial_73 | Adj.tertPvals | 0.624786 |
| GSE13213.Surv_AllMethods_Freq_6 | trial_73 | Adj.COX_P_VALUE | 0.600208 |
| GSE13213.Surv_AllMethods_Freq_2 | trial_74 | Adj.CoxPvalbyRanks | 0.674953 |
| GSE13213.Surv_AllMethods_Freq_3 | trial_74 | Adj.CoxPvalbyRanks | 0.687022 |
| GSE13213.Surv_AllMethods_Freq_4 | trial_74 | Adj.tertPvals | 0.681693 |
| GSE13213.Surv_AllMethods_Freq_5 | trial_74 | Adj.tertPvals | 0.624786 |
| GSE13213.Surv_AllMethods_Freq_6 | trial_74 | Adj.GoodCountPvals | 0.603063 |
| GSE13213.Surv_AllMethods_Freq_2 | trial_75 | Adj.CoxPvalbyRanks | 0.674953 |
| GSE13213.Surv_AllMethods_Freq_3 | trial_75 | Adj.CoxPvalbyRanks | 0.687022 |
| GSE13213.Surv_AllMethods_Freq_4 | trial_75 | Adj.tertPvals | 0.681693 |
| GSE13213.Surv_AllMethods_Freq_5 | trial_75 | Adj.tertPvals | 0.624786 |
| GSE13213.Surv_AllMethods_Freq_6 | trial_75 | Adj.GoodCountPvals | 0.602544 |
| GSE13213.Surv_AllMethods_Freq_2 | trial_76 | Adj.CoxPvalbyRanks | 0.674953 |
| GSE13213.Surv_AllMethods_Freq_3 | trial_76 | Adj.CoxPvalbyRanks | 0.687022 |
| GSE13213.Surv_AllMethods_Freq_4 | trial_76 | Adj.tertPvals | 0.681693 |
| GSE13213.Surv_AllMethods_Freq_5 | trial_76 | Adj.tertPvals | 0.624786 |
| GSE13213.Surv_AllMethods_Freq_6 | trial_76 | Adj.GoodCountPvals | 0.603323 |
| GSE13213.Surv_AllMethods_Freq_2 | trial_77 | Adj.CoxPvalbyRanks | 0.674953 |
| GSE13213.Surv_AllMethods_Freq_3 | trial_77 | Adj.CoxPvalbyRanks | 0.687022 |
| GSE13213.Surv_AllMethods_Freq_4 | trial_77 | Adj.tertPvals | 0.681693 |
| GSE13213.Surv_AllMethods_Freq_5 | trial_77 | Adj.tertPvals | 0.624786 |
| GSE13213.Surv_AllMethods_Freq_6 | trial_77 | Adj.GoodCountPvals | 0.602285 |
| GSE13213.Surv_AllMethods_Freq_2 | trial_78 | Adj.CoxPvalbyRanks | 0.674953 |
| GSE13213.Surv_AllMethods_Freq_3 | trial_78 | Adj.CoxPvalbyRanks | 0.687022 |
| GSE13213.Surv_AllMethods_Freq_4 | trial_78 | Adj.tertPvals | 0.681693 |
| GSE13213.Surv_AllMethods_Freq_5 | trial_78 | Adj.tertPvals | 0.624786 |
| GSE13213.Surv_AllMethods_Freq_6 | trial_78 | Adj.COX_P_VALUE | 0.600208 |
| GSE13213.Surv_AllMethods_Freq_2 | trial_79 | Adj.CoxPvalbyRanks | 0.674953 |
| GSE13213.Surv_AllMethods_Freq_3 | trial_79 | Adj.CoxPvalbyRanks | 0.687022 |
| GSE13213.Surv_AllMethods_Freq_4 | trial_79 | Adj.tertPvals | 0.681693 |
| GSE13213.Surv_AllMethods_Freq_5 | trial_79 | Adj.tertPvals | 0.624786 |
| GSE13213.Surv_AllMethods_Freq_6 | trial_79 | Adj.COX_P_VALUE | 0.600208 |
| GSE13213.Surv_AllMethods_Freq_2 | trial_80 | Adj.CoxPvalbyRanks | 0.674953 |
| GSE13213.Surv_AllMethods_Freq_3 | trial_80 | Adj.CoxPvalbyRanks | 0.687022 |
| GSE13213.Surv_AllMethods_Freq_4 | trial_80 | Adj.tertPvals | 0.681693 |
| GSE13213.Surv_AllMethods_Freq_5 | trial_80 | Adj.tertPvals | 0.624786 |
| GSE13213.Surv_AllMethods_Freq_6 | trial_80 | Adj.GoodCountPvals | 0.604881 |
| GSE13213.Surv_AllMethods_Freq_2 | trial_81 | Adj.CoxPvalbyRanks | 0.674953 |
| GSE13213.Surv_AllMethods_Freq_3 | trial_81 | Adj.CoxPvalbyRanks | 0.687022 |
| GSE13213.Surv_AllMethods_Freq_4 | trial_81 | Adj.tertPvals | 0.681693 |
| GSE13213.Surv_AllMethods_Freq_5 | trial_81 | Adj.tertPvals | 0.624786 |
| GSE13213.Surv_AllMethods_Freq_6 | trial_81 | Adj.COX_P_VALUE | 0.600208 |
| GSE13213.Surv_AllMethods_Freq_2 | trial_82 | Adj.CoxPvalbyRanks | 0.674953 |
| GSE13213.Surv_AllMethods_Freq_3 | trial_82 | Adj.CoxPvalbyRanks | 0.687022 |
| GSE13213.Surv_AllMethods_Freq_4 | trial_82 | Adj.tertPvals | 0.681693 |
| GSE13213.Surv_AllMethods_Freq_5 | trial_82 | Adj.tertPvals | 0.624786 |
| GSE13213.Surv_AllMethods_Freq_6 | trial_82 | Adj.COX_P_VALUE | 0.600208 |
| GSE13213.Surv_AllMethods_Freq_2 | trial_83 | Adj.CoxPvalbyRanks | 0.674953 |
| GSE13213.Surv_AllMethods_Freq_3 | trial_83 | Adj.CoxPvalbyRanks | 0.687022 |
| GSE13213.Surv_AllMethods_Freq_4 | trial_83 | Adj.tertPvals | 0.681693 |
| GSE13213.Surv_AllMethods_Freq_5 | trial_83 | Adj.tertPvals | 0.624786 |
| GSE13213.Surv_AllMethods_Freq_6 | trial_83 | Adj.COX_P_VALUE | 0.600208 |
| GSE13213.Surv_AllMethods_Freq_2 | trial_84 | Adj.CoxPvalbyRanks | 0.674953 |
| GSE13213.Surv_AllMethods_Freq_3 | trial_84 | Adj.CoxPvalbyRanks | 0.687022 |
| GSE13213.Surv_AllMethods_Freq_4 | trial_84 | Adj.tertPvals | 0.681693 |
| GSE13213.Surv_AllMethods_Freq_5 | trial_84 | Adj.tertPvals | 0.624786 |
| GSE13213.Surv_AllMethods_Freq_6 | trial_84 | Adj.GoodCountPvals | 0.602544 |
| GSE13213.Surv_AllMethods_Freq_2 | trial_85 | Adj.CoxPvalbyRanks | 0.674953 |
| GSE13213.Surv_AllMethods_Freq_3 | trial_85 | Adj.CoxPvalbyRanks | 0.687022 |
| GSE13213.Surv_AllMethods_Freq_4 | trial_85 | Adj.tertPvals | 0.681693 |
| GSE13213.Surv_AllMethods_Freq_5 | trial_85 | Adj.tertPvals | 0.624786 |
| GSE13213.Surv_AllMethods_Freq_6 | trial_85 | Adj.COX_P_VALUE | 0.600208 |
| GSE13213.Surv_AllMethods_Freq_2 | trial_86 | Adj.CoxPvalbyRanks | 0.674953 |
| GSE13213.Surv_AllMethods_Freq_3 | trial_86 | Adj.CoxPvalbyRanks | 0.687022 |
| GSE13213.Surv_AllMethods_Freq_4 | trial_86 | Adj.tertPvals | 0.681693 |
| GSE13213.Surv_AllMethods_Freq_5 | trial_86 | Adj.tertPvals | 0.624786 |
| GSE13213.Surv_AllMethods_Freq_6 | trial_86 | Adj.COX_P_VALUE | 0.600208 |
| GSE13213.Surv_AllMethods_Freq_2 | trial_87 | Adj.CoxPvalbyRanks | 0.674953 |
| GSE13213.Surv_AllMethods_Freq_3 | trial_87 | Adj.CoxPvalbyRanks | 0.687022 |
| GSE13213.Surv_AllMethods_Freq_4 | trial_87 | Adj.tertPvals | 0.681693 |
| GSE13213.Surv_AllMethods_Freq_5 | trial_87 | Adj.tertPvals | 0.624786 |
| GSE13213.Surv_AllMethods_Freq_6 | trial_87 | Adj.COX_P_VALUE | 0.600208 |
| GSE13213.Surv_AllMethods_Freq_2 | trial_88 | Adj.CoxPvalbyRanks | 0.674953 |
| GSE13213.Surv_AllMethods_Freq_3 | trial_88 | Adj.CoxPvalbyRanks | 0.687022 |
| GSE13213.Surv_AllMethods_Freq_4 | trial_88 | Adj.tertPvals | 0.681693 |
| GSE13213.Surv_AllMethods_Freq_5 | trial_88 | Adj.tertPvals | 0.624786 |
| GSE13213.Surv_AllMethods_Freq_6 | trial_88 | Adj.COX_P_VALUE | 0.600208 |
| GSE13213.Surv_AllMethods_Freq_2 | trial_89 | Adj.CoxPvalbyRanks | 0.674953 |
| GSE13213.Surv_AllMethods_Freq_3 | trial_89 | Adj.CoxPvalbyRanks | 0.687022 |
| GSE13213.Surv_AllMethods_Freq_4 | trial_89 | Adj.tertPvals | 0.681693 |
| GSE13213.Surv_AllMethods_Freq_5 | trial_89 | Adj.tertPvals | 0.624786 |
| GSE13213.Surv_AllMethods_Freq_6 | trial_89 | Adj.COX_P_VALUE | 0.600208 |
| GSE13213.Surv_AllMethods_Freq_2 | trial_90 | Adj.CoxPvalbyRanks | 0.674953 |
| GSE13213.Surv_AllMethods_Freq_3 | trial_90 | Adj.CoxPvalbyRanks | 0.687022 |
| GSE13213.Surv_AllMethods_Freq_4 | trial_90 | Adj.tertPvals | 0.681693 |
| GSE13213.Surv_AllMethods_Freq_5 | trial_90 | Adj.tertPvals | 0.624786 |
| GSE13213.Surv_AllMethods_Freq_6 | trial_90 | Adj.COX_P_VALUE | 0.600208 |
| GSE13213.Surv_AllMethods_Freq_2 | trial_91 | Adj.CoxPvalbyRanks | 0.674953 |
| GSE13213.Surv_AllMethods_Freq_3 | trial_91 | Adj.CoxPvalbyRanks | 0.687022 |
| GSE13213.Surv_AllMethods_Freq_4 | trial_91 | Adj.tertPvals | 0.681693 |
| GSE13213.Surv_AllMethods_Freq_5 | trial_91 | Adj.tertPvals | 0.624786 |
| GSE13213.Surv_AllMethods_Freq_6 | trial_91 | Adj.COX_P_VALUE | 0.600208 |
| GSE13213.Surv_AllMethods_Freq_2 | trial_92 | Adj.CoxPvalbyRanks | 0.674953 |
| GSE13213.Surv_AllMethods_Freq_3 | trial_92 | Adj.CoxPvalbyRanks | 0.687022 |
| GSE13213.Surv_AllMethods_Freq_4 | trial_92 | Adj.tertPvals | 0.681693 |
| GSE13213.Surv_AllMethods_Freq_5 | trial_92 | Adj.tertPvals | 0.624786 |
| GSE13213.Surv_AllMethods_Freq_6 | trial_92 | Adj.COX_P_VALUE | 0.600208 |
| GSE13213.Surv_AllMethods_Freq_2 | trial_93 | Adj.CoxPvalbyRanks | 0.674953 |
| GSE13213.Surv_AllMethods_Freq_3 | trial_93 | Adj.CoxPvalbyRanks | 0.687022 |
| GSE13213.Surv_AllMethods_Freq_4 | trial_93 | Adj.tertPvals | 0.681693 |
| GSE13213.Surv_AllMethods_Freq_5 | trial_93 | Adj.tertPvals | 0.624786 |
| GSE13213.Surv_AllMethods_Freq_6 | trial_93 | Adj.COX_P_VALUE | 0.600208 |
| GSE13213.Surv_AllMethods_Freq_2 | trial_94 | Adj.CoxPvalbyRanks | 0.674953 |
| GSE13213.Surv_AllMethods_Freq_3 | trial_94 | Adj.CoxPvalbyRanks | 0.687022 |
| GSE13213.Surv_AllMethods_Freq_4 | trial_94 | Adj.tertPvals | 0.681693 |
| GSE13213.Surv_AllMethods_Freq_5 | trial_94 | Adj.tertPvals | 0.624786 |
| GSE13213.Surv_AllMethods_Freq_6 | trial_94 | Adj.COX_P_VALUE | 0.600208 |
| GSE13213.Surv_AllMethods_Freq_2 | trial_95 | Adj.CoxPvalbyRanks | 0.674953 |
| GSE13213.Surv_AllMethods_Freq_3 | trial_95 | Adj.CoxPvalbyRanks | 0.687022 |
| GSE13213.Surv_AllMethods_Freq_4 | trial_95 | Adj.tertPvals | 0.681693 |
| GSE13213.Surv_AllMethods_Freq_5 | trial_95 | Adj.tertPvals | 0.624786 |
| GSE13213.Surv_AllMethods_Freq_6 | trial_95 | Adj.COX_P_VALUE | 0.600208 |
| GSE13213.Surv_AllMethods_Freq_2 | trial_96 | Adj.CoxPvalbyRanks | 0.674953 |
| GSE13213.Surv_AllMethods_Freq_3 | trial_96 | Adj.CoxPvalbyRanks | 0.687022 |
| GSE13213.Surv_AllMethods_Freq_4 | trial_96 | Adj.tertPvals | 0.681693 |
| GSE13213.Surv_AllMethods_Freq_5 | trial_96 | Adj.tertPvals | 0.624786 |
| GSE13213.Surv_AllMethods_Freq_6 | trial_96 | Adj.GoodCountPvals | 0.600467 |
| GSE13213.Surv_AllMethods_Freq_2 | trial_97 | Adj.CoxPvalbyRanks | 0.674953 |
| GSE13213.Surv_AllMethods_Freq_3 | trial_97 | Adj.CoxPvalbyRanks | 0.687022 |
| GSE13213.Surv_AllMethods_Freq_4 | trial_97 | Adj.tertPvals | 0.681693 |
| GSE13213.Surv_AllMethods_Freq_5 | trial_97 | Adj.tertPvals | 0.624786 |
| GSE13213.Surv_AllMethods_Freq_6 | trial_97 | Adj.GoodCountPvals | 0.601246 |
| GSE13213.Surv_AllMethods_Freq_2 | trial_98 | Adj.CoxPvalbyRanks | 0.674953 |
| GSE13213.Surv_AllMethods_Freq_3 | trial_98 | Adj.CoxPvalbyRanks | 0.687022 |
| GSE13213.Surv_AllMethods_Freq_4 | trial_98 | Adj.tertPvals | 0.681693 |
| GSE13213.Surv_AllMethods_Freq_5 | trial_98 | Adj.tertPvals | 0.624786 |
| GSE13213.Surv_AllMethods_Freq_6 | trial_98 | Adj.COX_P_VALUE | 0.600208 |
| GSE13213.Surv_AllMethods_Freq_2 | trial_99 | Adj.CoxPvalbyRanks | 0.674953 |
| GSE13213.Surv_AllMethods_Freq_3 | trial_99 | Adj.CoxPvalbyRanks | 0.687022 |
| GSE13213.Surv_AllMethods_Freq_4 | trial_99 | Adj.tertPvals | 0.681693 |
| GSE13213.Surv_AllMethods_Freq_5 | trial_99 | Adj.tertPvals | 0.624786 |
| GSE13213.Surv_AllMethods_Freq_6 | trial_99 | Adj.COX_P_VALUE | 0.600208 |
| GSE13213.Surv_AllMethods_Freq_2 | trial_100 | Adj.CoxPvalbyRanks | 0.674953 |
| GSE13213.Surv_AllMethods_Freq_3 | trial_100 | Adj.CoxPvalbyRanks | 0.687022 |
| GSE13213.Surv_AllMethods_Freq_4 | trial_100 | Adj.tertPvals | 0.681693 |
| GSE13213.Surv_AllMethods_Freq_5 | trial_100 | Adj.tertPvals | 0.624786 |
| GSE13213.Surv_AllMethods_Freq_6 | trial_100 | Adj.GoodCountPvals | 0.602804 |
| GSE31210.Surv_AllMethods_Freq_2 | trial_1 | Adj.MedianPvals | 0.613729 |
| GSE31210.Surv_AllMethods_Freq_3 | trial_1 | Adj.MedianPvals | 0.607754 |
| GSE31210.Surv_AllMethods_Freq_4 | trial_1 | Adj.GoodCountPvals | 0.627429 |
| GSE31210.Surv_AllMethods_Freq_5 | trial_1 | Adj.GoodCountPvals | 0.633146 |
| GSE31210.Surv_AllMethods_Freq_6 | trial_1 | Adj.GoodCountPvals | 0.687363 |
| GSE31210.Surv_AllMethods_Freq_2 | trial_2 | Adj.MedianPvals | 0.613729 |
| GSE31210.Surv_AllMethods_Freq_3 | trial_2 | Adj.MedianPvals | 0.607754 |
| GSE31210.Surv_AllMethods_Freq_4 | trial_2 | Adj.GoodCountPvals | 0.624504 |
| GSE31210.Surv_AllMethods_Freq_5 | trial_2 | Adj.GoodCountPvals | 0.631398 |
| GSE31210.Surv_AllMethods_Freq_6 | trial_2 | Adj.GoodCountPvals | 0.683516 |
| GSE31210.Surv_AllMethods_Freq_2 | trial_3 | Adj.MedianPvals | 0.613729 |
| GSE31210.Surv_AllMethods_Freq_3 | trial_3 | Adj.MedianPvals | 0.607754 |
| GSE31210.Surv_AllMethods_Freq_4 | trial_3 | Adj.GoodCountPvals | 0.626541 |
| GSE31210.Surv_AllMethods_Freq_5 | trial_3 | Adj.GoodCountPvals | 0.631086 |
| GSE31210.Surv_AllMethods_Freq_6 | trial_3 | Adj.GoodCountPvals | 0.687179 |
| GSE31210.Surv_AllMethods_Freq_2 | trial_4 | Adj.MedianPvals | 0.613729 |
| GSE31210.Surv_AllMethods_Freq_3 | trial_4 | Adj.MedianPvals | 0.607754 |
| GSE31210.Surv_AllMethods_Freq_4 | trial_4 | Adj.GoodCountPvals | 0.624608 |
| GSE31210.Surv_AllMethods_Freq_5 | trial_4 | Adj.GoodCountPvals | 0.630524 |
| GSE31210.Surv_AllMethods_Freq_6 | trial_4 | Adj.GoodCountPvals | 0.6837 |
| GSE31210.Surv_AllMethods_Freq_2 | trial_5 | Adj.MedianPvals | 0.613729 |
| GSE31210.Surv_AllMethods_Freq_3 | trial_5 | Adj.MedianPvals | 0.607754 |
| GSE31210.Surv_AllMethods_Freq_4 | trial_5 | Adj.GoodCountPvals | 0.627273 |
| GSE31210.Surv_AllMethods_Freq_5 | trial_5 | Adj.CORRECTED_P_VALUE | 0.629713 |
| GSE31210.Surv_AllMethods_Freq_6 | trial_5 | Adj.GoodCountPvals | 0.6837 |
| GSE31210.Surv_AllMethods_Freq_2 | trial_6 | Adj.MedianPvals | 0.613729 |
| GSE31210.Surv_AllMethods_Freq_3 | trial_6 | Adj.MedianPvals | 0.607754 |
| GSE31210.Surv_AllMethods_Freq_4 | trial_6 | Adj.GoodCountPvals | 0.625026 |
| GSE31210.Surv_AllMethods_Freq_5 | trial_6 | Adj.GoodCountPvals | 0.6299 |
| GSE31210.Surv_AllMethods_Freq_6 | trial_6 | Adj.GoodCountPvals | 0.682692 |
| GSE31210.Surv_AllMethods_Freq_2 | trial_7 | Adj.MedianPvals | 0.613729 |
| GSE31210.Surv_AllMethods_Freq_3 | trial_7 | Adj.MedianPvals | 0.607754 |
| GSE31210.Surv_AllMethods_Freq_4 | trial_7 | Adj.GoodCountPvals | 0.626855 |
| GSE31210.Surv_AllMethods_Freq_5 | trial_7 | Adj.GoodCountPvals | 0.632272 |
| GSE31210.Surv_AllMethods_Freq_6 | trial_7 | Adj.GoodCountPvals | 0.687546 |
| GSE31210.Surv_AllMethods_Freq_2 | trial_8 | Adj.MedianPvals | 0.613729 |
| GSE31210.Surv_AllMethods_Freq_3 | trial_8 | Adj.MedianPvals | 0.607754 |
| GSE31210.Surv_AllMethods_Freq_4 | trial_8 | Adj.GoodCountPvals | 0.628474 |
| GSE31210.Surv_AllMethods_Freq_5 | trial_8 | Adj.GoodCountPvals | 0.633895 |
| GSE31210.Surv_AllMethods_Freq_6 | trial_8 | Adj.GoodCountPvals | 0.690293 |
| GSE31210.Surv_AllMethods_Freq_2 | trial_9 | Adj.MedianPvals | 0.613729 |
| GSE31210.Surv_AllMethods_Freq_3 | trial_9 | Adj.MedianPvals | 0.607754 |
| GSE31210.Surv_AllMethods_Freq_4 | trial_9 | Adj.GoodCountPvals | 0.62675 |
| GSE31210.Surv_AllMethods_Freq_5 | trial_9 | Adj.GoodCountPvals | 0.63171 |
| GSE31210.Surv_AllMethods_Freq_6 | trial_9 | Adj.GoodCountPvals | 0.685348 |
| GSE31210.Surv_AllMethods_Freq_2 | trial_10 | Adj.MedianPvals | 0.613729 |
| GSE31210.Surv_AllMethods_Freq_3 | trial_10 | Adj.MedianPvals | 0.607754 |
| GSE31210.Surv_AllMethods_Freq_4 | trial_10 | Adj.GoodCountPvals | 0.625758 |
| GSE31210.Surv_AllMethods_Freq_5 | trial_10 | Adj.GoodCountPvals | 0.632522 |
| GSE31210.Surv_AllMethods_Freq_6 | trial_10 | Adj.GoodCountPvals | 0.68663 |
| GSE31210.Surv_AllMethods_Freq_2 | trial_11 | Adj.MedianPvals | 0.613729 |
| GSE31210.Surv_AllMethods_Freq_3 | trial_11 | Adj.MedianPvals | 0.607754 |
| GSE31210.Surv_AllMethods_Freq_4 | trial_11 | Adj.GoodCountPvals | 0.628004 |
| GSE31210.Surv_AllMethods_Freq_5 | trial_11 | Adj.GoodCountPvals | 0.635331 |
| GSE31210.Surv_AllMethods_Freq_6 | trial_11 | Adj.GoodCountPvals | 0.690018 |
| GSE31210.Surv_AllMethods_Freq_2 | trial_12 | Adj.MedianPvals | 0.613729 |
| GSE31210.Surv_AllMethods_Freq_3 | trial_12 | Adj.MedianPvals | 0.607754 |
| GSE31210.Surv_AllMethods_Freq_4 | trial_12 | Adj.GoodCountPvals | 0.625862 |
| GSE31210.Surv_AllMethods_Freq_5 | trial_12 | Adj.GoodCountPvals | 0.631835 |
| GSE31210.Surv_AllMethods_Freq_6 | trial_12 | Adj.GoodCountPvals | 0.686355 |
| GSE31210.Surv_AllMethods_Freq_2 | trial_13 | Adj.MedianPvals | 0.613729 |
| GSE31210.Surv_AllMethods_Freq_3 | trial_13 | Adj.MedianPvals | 0.607754 |
| GSE31210.Surv_AllMethods_Freq_4 | trial_13 | Adj.GoodCountPvals | 0.626385 |
| GSE31210.Surv_AllMethods_Freq_5 | trial_13 | Adj.GoodCountPvals | 0.6304 |
| GSE31210.Surv_AllMethods_Freq_6 | trial_13 | Adj.GoodCountPvals | 0.684249 |
| GSE31210.Surv_AllMethods_Freq_2 | trial_14 | Adj.MedianPvals | 0.613729 |
| GSE31210.Surv_AllMethods_Freq_3 | trial_14 | Adj.MedianPvals | 0.607754 |
| GSE31210.Surv_AllMethods_Freq_4 | trial_14 | Adj.GoodCountPvals | 0.62628 |
| GSE31210.Surv_AllMethods_Freq_5 | trial_14 | Adj.GoodCountPvals | 0.630774 |
| GSE31210.Surv_AllMethods_Freq_6 | trial_14 | Adj.GoodCountPvals | 0.685256 |
| GSE31210.Surv_AllMethods_Freq_2 | trial_15 | Adj.MedianPvals | 0.613729 |
| GSE31210.Surv_AllMethods_Freq_3 | trial_15 | Adj.MedianPvals | 0.607754 |
| GSE31210.Surv_AllMethods_Freq_4 | trial_15 | Adj.GoodCountPvals | 0.625601 |
| GSE31210.Surv_AllMethods_Freq_5 | trial_15 | Adj.GoodCountPvals | 0.633208 |
| GSE31210.Surv_AllMethods_Freq_6 | trial_15 | Adj.GoodCountPvals | 0.687179 |
| GSE31210.Surv_AllMethods_Freq_2 | trial_16 | Adj.MedianPvals | 0.613729 |
| GSE31210.Surv_AllMethods_Freq_3 | trial_16 | Adj.MedianPvals | 0.607754 |
| GSE31210.Surv_AllMethods_Freq_4 | trial_16 | Adj.GoodCountPvals | 0.625287 |
| GSE31210.Surv_AllMethods_Freq_5 | trial_16 | Adj.GoodCountPvals | 0.631586 |
| GSE31210.Surv_AllMethods_Freq_6 | trial_16 | Adj.GoodCountPvals | 0.686722 |
| GSE31210.Surv_AllMethods_Freq_2 | trial_17 | Adj.MedianPvals | 0.613729 |
| GSE31210.Surv_AllMethods_Freq_3 | trial_17 | Adj.MedianPvals | 0.607754 |
| GSE31210.Surv_AllMethods_Freq_4 | trial_17 | Adj.GoodCountPvals | 0.627325 |
| GSE31210.Surv_AllMethods_Freq_5 | trial_17 | Adj.GoodCountPvals | 0.633271 |
| GSE31210.Surv_AllMethods_Freq_6 | trial_17 | Adj.GoodCountPvals | 0.688462 |
| GSE31210.Surv_AllMethods_Freq_2 | trial_18 | Adj.MedianPvals | 0.613729 |
| GSE31210.Surv_AllMethods_Freq_3 | trial_18 | Adj.MedianPvals | 0.607754 |
| GSE31210.Surv_AllMethods_Freq_4 | trial_18 | Adj.GoodCountPvals | 0.625287 |
| GSE31210.Surv_AllMethods_Freq_5 | trial_18 | Adj.GoodCountPvals | 0.632834 |
| GSE31210.Surv_AllMethods_Freq_6 | trial_18 | Adj.GoodCountPvals | 0.687729 |
| GSE31210.Surv_AllMethods_Freq_2 | trial_19 | Adj.MedianPvals | 0.613729 |
| GSE31210.Surv_AllMethods_Freq_3 | trial_19 | Adj.MedianPvals | 0.607754 |
| GSE31210.Surv_AllMethods_Freq_4 | trial_19 | Adj.GoodCountPvals | 0.625287 |
| GSE31210.Surv_AllMethods_Freq_5 | trial_19 | Adj.GoodCountPvals | 0.631461 |
| GSE31210.Surv_AllMethods_Freq_6 | trial_19 | Adj.GoodCountPvals | 0.68663 |
| GSE31210.Surv_AllMethods_Freq_2 | trial_20 | Adj.MedianPvals | 0.613729 |
| GSE31210.Surv_AllMethods_Freq_3 | trial_20 | Adj.MedianPvals | 0.607754 |
| GSE31210.Surv_AllMethods_Freq_4 | trial_20 | Adj.GoodCountPvals | 0.627116 |
| GSE31210.Surv_AllMethods_Freq_5 | trial_20 | Adj.GoodCountPvals | 0.632397 |
| GSE31210.Surv_AllMethods_Freq_6 | trial_20 | Adj.GoodCountPvals | 0.685531 |
| GSE31210.Surv_AllMethods_Freq_2 | trial_21 | Adj.MedianPvals | 0.613729 |
| GSE31210.Surv_AllMethods_Freq_3 | trial_21 | Adj.MedianPvals | 0.607754 |
| GSE31210.Surv_AllMethods_Freq_4 | trial_21 | Adj.CORRECTED_P_VALUE | 0.624033 |
| GSE31210.Surv_AllMethods_Freq_5 | trial_21 | Adj.GoodCountPvals | 0.630462 |
| GSE31210.Surv_AllMethods_Freq_6 | trial_21 | Adj.GoodCountPvals | 0.684982 |
| GSE31210.Surv_AllMethods_Freq_2 | trial_22 | Adj.MedianPvals | 0.613729 |
| GSE31210.Surv_AllMethods_Freq_3 | trial_22 | Adj.MedianPvals | 0.607754 |
| GSE31210.Surv_AllMethods_Freq_4 | trial_22 | Adj.GoodCountPvals | 0.62675 |
| GSE31210.Surv_AllMethods_Freq_5 | trial_22 | Adj.GoodCountPvals | 0.63427 |
| GSE31210.Surv_AllMethods_Freq_6 | trial_22 | Adj.GoodCountPvals | 0.68837 |
| GSE31210.Surv_AllMethods_Freq_2 | trial_23 | Adj.MedianPvals | 0.613729 |
| GSE31210.Surv_AllMethods_Freq_3 | trial_23 | Adj.MedianPvals | 0.607754 |
| GSE31210.Surv_AllMethods_Freq_4 | trial_23 | Adj.GoodCountPvals | 0.626019 |
| GSE31210.Surv_AllMethods_Freq_5 | trial_23 | Adj.CORRECTED_P_VALUE | 0.629713 |
| GSE31210.Surv_AllMethods_Freq_6 | trial_23 | Adj.GoodCountPvals | 0.683425 |
| GSE31210.Surv_AllMethods_Freq_2 | trial_24 | Adj.MedianPvals | 0.613729 |
| GSE31210.Surv_AllMethods_Freq_3 | trial_24 | Adj.MedianPvals | 0.607754 |
| GSE31210.Surv_AllMethods_Freq_4 | trial_24 | Adj.GoodCountPvals | 0.624765 |
| GSE31210.Surv_AllMethods_Freq_5 | trial_24 | Adj.CORRECTED_P_VALUE | 0.629713 |
| GSE31210.Surv_AllMethods_Freq_6 | trial_24 | Adj.GoodCountPvals | 0.682967 |
| GSE31210.Surv_AllMethods_Freq_2 | trial_25 | Adj.MedianPvals | 0.613729 |
| GSE31210.Surv_AllMethods_Freq_3 | trial_25 | Adj.MedianPvals | 0.607754 |
| GSE31210.Surv_AllMethods_Freq_4 | trial_25 | Adj.GoodCountPvals | 0.627638 |
| GSE31210.Surv_AllMethods_Freq_5 | trial_25 | Adj.GoodCountPvals | 0.632147 |
| GSE31210.Surv_AllMethods_Freq_6 | trial_25 | Adj.GoodCountPvals | 0.688553 |
| GSE31210.Surv_AllMethods_Freq_2 | trial_26 | Adj.GoodCountPvals | 0.614021 |
| GSE31210.Surv_AllMethods_Freq_3 | trial_26 | Adj.MedianPvals | 0.607754 |
| GSE31210.Surv_AllMethods_Freq_4 | trial_26 | Adj.GoodCountPvals | 0.629833 |
| GSE31210.Surv_AllMethods_Freq_5 | trial_26 | Adj.GoodCountPvals | 0.63608 |
| GSE31210.Surv_AllMethods_Freq_6 | trial_26 | Adj.GoodCountPvals | 0.688462 |
| GSE31210.Surv_AllMethods_Freq_2 | trial_27 | Adj.MedianPvals | 0.613729 |
| GSE31210.Surv_AllMethods_Freq_3 | trial_27 | Adj.MedianPvals | 0.607754 |
| GSE31210.Surv_AllMethods_Freq_4 | trial_27 | Adj.CORRECTED_P_VALUE | 0.624033 |
| GSE31210.Surv_AllMethods_Freq_5 | trial_27 | Adj.CORRECTED_P_VALUE | 0.629713 |
| GSE31210.Surv_AllMethods_Freq_6 | trial_27 | Adj.GoodCountPvals | 0.683516 |
| GSE31210.Surv_AllMethods_Freq_2 | trial_28 | Adj.MedianPvals | 0.613729 |
| GSE31210.Surv_AllMethods_Freq_3 | trial_28 | Adj.MedianPvals | 0.607754 |
| GSE31210.Surv_AllMethods_Freq_4 | trial_28 | Adj.CORRECTED_P_VALUE | 0.624033 |
| GSE31210.Surv_AllMethods_Freq_5 | trial_28 | Adj.GoodCountPvals | 0.631835 |
| GSE31210.Surv_AllMethods_Freq_6 | trial_28 | Adj.GoodCountPvals | 0.684707 |
| GSE31210.Surv_AllMethods_Freq_2 | trial_29 | Adj.MedianPvals | 0.613729 |
| GSE31210.Surv_AllMethods_Freq_3 | trial_29 | Adj.MedianPvals | 0.607754 |
| GSE31210.Surv_AllMethods_Freq_4 | trial_29 | Adj.GoodCountPvals | 0.627586 |
| GSE31210.Surv_AllMethods_Freq_5 | trial_29 | Adj.GoodCountPvals | 0.632459 |
| GSE31210.Surv_AllMethods_Freq_6 | trial_29 | Adj.GoodCountPvals | 0.684341 |
| GSE31210.Surv_AllMethods_Freq_2 | trial_30 | Adj.MedianPvals | 0.613729 |
| GSE31210.Surv_AllMethods_Freq_3 | trial_30 | Adj.MedianPvals | 0.607754 |
| GSE31210.Surv_AllMethods_Freq_4 | trial_30 | Adj.GoodCountPvals | 0.624086 |
| GSE31210.Surv_AllMethods_Freq_5 | trial_30 | Adj.CORRECTED_P_VALUE | 0.629713 |
| GSE31210.Surv_AllMethods_Freq_6 | trial_30 | Adj.GoodCountPvals | 0.681227 |
| GSE31210.Surv_AllMethods_Freq_2 | trial_31 | Adj.MedianPvals | 0.613729 |
| GSE31210.Surv_AllMethods_Freq_3 | trial_31 | Adj.MedianPvals | 0.607754 |
| GSE31210.Surv_AllMethods_Freq_4 | trial_31 | Adj.CORRECTED_P_VALUE | 0.624033 |
| GSE31210.Surv_AllMethods_Freq_5 | trial_31 | Adj.GoodCountPvals | 0.630212 |
| GSE31210.Surv_AllMethods_Freq_6 | trial_31 | Adj.GoodCountPvals | 0.682784 |
| GSE31210.Surv_AllMethods_Freq_2 | trial_32 | Adj.MedianPvals | 0.613729 |
| GSE31210.Surv_AllMethods_Freq_3 | trial_32 | Adj.MedianPvals | 0.607754 |
| GSE31210.Surv_AllMethods_Freq_4 | trial_32 | Adj.GoodCountPvals | 0.625914 |
| GSE31210.Surv_AllMethods_Freq_5 | trial_32 | Adj.GoodCountPvals | 0.631898 |
| GSE31210.Surv_AllMethods_Freq_6 | trial_32 | Adj.GoodCountPvals | 0.685806 |
| GSE31210.Surv_AllMethods_Freq_2 | trial_33 | Adj.MedianPvals | 0.613729 |
| GSE31210.Surv_AllMethods_Freq_3 | trial_33 | Adj.MedianPvals | 0.607754 |
| GSE31210.Surv_AllMethods_Freq_4 | trial_33 | Adj.GoodCountPvals | 0.625444 |
| GSE31210.Surv_AllMethods_Freq_5 | trial_33 | Adj.CORRECTED_P_VALUE | 0.629713 |
| GSE31210.Surv_AllMethods_Freq_6 | trial_33 | Adj.GoodCountPvals | 0.682601 |
| GSE31210.Surv_AllMethods_Freq_2 | trial_34 | Adj.MedianPvals | 0.613729 |
| GSE31210.Surv_AllMethods_Freq_3 | trial_34 | Adj.MedianPvals | 0.607754 |
| GSE31210.Surv_AllMethods_Freq_4 | trial_34 | Adj.GoodCountPvals | 0.624974 |
| GSE31210.Surv_AllMethods_Freq_5 | trial_34 | Adj.GoodCountPvals | 0.631773 |
| GSE31210.Surv_AllMethods_Freq_6 | trial_34 | Adj.GoodCountPvals | 0.684982 |
| GSE31210.Surv_AllMethods_Freq_2 | trial_35 | Adj.MedianPvals | 0.613729 |
| GSE31210.Surv_AllMethods_Freq_3 | trial_35 | Adj.MedianPvals | 0.607754 |
| GSE31210.Surv_AllMethods_Freq_4 | trial_35 | Adj.GoodCountPvals | 0.628109 |
| GSE31210.Surv_AllMethods_Freq_5 | trial_35 | Adj.GoodCountPvals | 0.634956 |
| GSE31210.Surv_AllMethods_Freq_6 | trial_35 | Adj.GoodCountPvals | 0.688187 |
| GSE31210.Surv_AllMethods_Freq_2 | trial_36 | Adj.MedianPvals | 0.613729 |
| GSE31210.Surv_AllMethods_Freq_3 | trial_36 | Adj.MedianPvals | 0.607754 |
| GSE31210.Surv_AllMethods_Freq_4 | trial_36 | Adj.GoodCountPvals | 0.627064 |
| GSE31210.Surv_AllMethods_Freq_5 | trial_36 | Adj.GoodCountPvals | 0.633146 |
| GSE31210.Surv_AllMethods_Freq_6 | trial_36 | Adj.GoodCountPvals | 0.689103 |
| GSE31210.Surv_AllMethods_Freq_2 | trial_37 | Adj.MedianPvals | 0.613729 |
| GSE31210.Surv_AllMethods_Freq_3 | trial_37 | Adj.MedianPvals | 0.607754 |
| GSE31210.Surv_AllMethods_Freq_4 | trial_37 | Adj.GoodCountPvals | 0.625078 |
| GSE31210.Surv_AllMethods_Freq_5 | trial_37 | Adj.GoodCountPvals | 0.631024 |
| GSE31210.Surv_AllMethods_Freq_6 | trial_37 | Adj.GoodCountPvals | 0.686355 |
| GSE31210.Surv_AllMethods_Freq_2 | trial_38 | Adj.MedianPvals | 0.613729 |
| GSE31210.Surv_AllMethods_Freq_3 | trial_38 | Adj.MedianPvals | 0.607754 |
| GSE31210.Surv_AllMethods_Freq_4 | trial_38 | Adj.CORRECTED_P_VALUE | 0.624033 |
| GSE31210.Surv_AllMethods_Freq_5 | trial_38 | Adj.CORRECTED_P_VALUE | 0.629713 |
| GSE31210.Surv_AllMethods_Freq_6 | trial_38 | Adj.GoodCountPvals | 0.68141 |
| GSE31210.Surv_AllMethods_Freq_2 | trial_39 | Adj.MedianPvals | 0.613729 |
| GSE31210.Surv_AllMethods_Freq_3 | trial_39 | Adj.MedianPvals | 0.607754 |
| GSE31210.Surv_AllMethods_Freq_4 | trial_39 | Adj.CORRECTED_P_VALUE | 0.624033 |
| GSE31210.Surv_AllMethods_Freq_5 | trial_39 | Adj.CORRECTED_P_VALUE | 0.629713 |
| GSE31210.Surv_AllMethods_Freq_6 | trial_39 | Adj.GoodCountPvals | 0.681502 |
| GSE31210.Surv_AllMethods_Freq_2 | trial_40 | Adj.MedianPvals | 0.613729 |
| GSE31210.Surv_AllMethods_Freq_3 | trial_40 | Adj.MedianPvals | 0.607754 |
| GSE31210.Surv_AllMethods_Freq_4 | trial_40 | Adj.GoodCountPvals | 0.627011 |
| GSE31210.Surv_AllMethods_Freq_5 | trial_40 | Adj.GoodCountPvals | 0.633708 |
| GSE31210.Surv_AllMethods_Freq_6 | trial_40 | Adj.GoodCountPvals | 0.690659 |
| GSE31210.Surv_AllMethods_Freq_2 | trial_41 | Adj.MedianPvals | 0.613729 |
| GSE31210.Surv_AllMethods_Freq_3 | trial_41 | Adj.MedianPvals | 0.607754 |
| GSE31210.Surv_AllMethods_Freq_4 | trial_41 | Adj.GoodCountPvals | 0.62722 |
| GSE31210.Surv_AllMethods_Freq_5 | trial_41 | Adj.GoodCountPvals | 0.633208 |
| GSE31210.Surv_AllMethods_Freq_6 | trial_41 | Adj.GoodCountPvals | 0.686996 |
| GSE31210.Surv_AllMethods_Freq_2 | trial_42 | Adj.MedianPvals | 0.613729 |
| GSE31210.Surv_AllMethods_Freq_3 | trial_42 | Adj.MedianPvals | 0.607754 |
| GSE31210.Surv_AllMethods_Freq_4 | trial_42 | Adj.CORRECTED_P_VALUE | 0.624033 |
| GSE31210.Surv_AllMethods_Freq_5 | trial_42 | Adj.CORRECTED_P_VALUE | 0.629713 |
| GSE31210.Surv_AllMethods_Freq_6 | trial_42 | Adj.GoodCountPvals | 0.681136 |
| GSE31210.Surv_AllMethods_Freq_2 | trial_43 | Adj.MedianPvals | 0.613729 |
| GSE31210.Surv_AllMethods_Freq_3 | trial_43 | Adj.MedianPvals | 0.607754 |
| GSE31210.Surv_AllMethods_Freq_4 | trial_43 | Adj.CORRECTED_P_VALUE | 0.624033 |
| GSE31210.Surv_AllMethods_Freq_5 | trial_43 | Adj.CORRECTED_P_VALUE | 0.629713 |
| GSE31210.Surv_AllMethods_Freq_6 | trial_43 | Adj.GoodCountPvals | 0.681136 |
| GSE31210.Surv_AllMethods_Freq_2 | trial_44 | Adj.MedianPvals | 0.613729 |
| GSE31210.Surv_AllMethods_Freq_3 | trial_44 | Adj.MedianPvals | 0.607754 |
| GSE31210.Surv_AllMethods_Freq_4 | trial_44 | Adj.GoodCountPvals | 0.627429 |
| GSE31210.Surv_AllMethods_Freq_5 | trial_44 | Adj.GoodCountPvals | 0.630649 |
| GSE31210.Surv_AllMethods_Freq_6 | trial_44 | Adj.GoodCountPvals | 0.684066 |
| GSE31210.Surv_AllMethods_Freq_2 | trial_45 | Adj.MedianPvals | 0.613729 |
| GSE31210.Surv_AllMethods_Freq_3 | trial_45 | Adj.MedianPvals | 0.607754 |
| GSE31210.Surv_AllMethods_Freq_4 | trial_45 | Adj.GoodCountPvals | 0.625496 |
| GSE31210.Surv_AllMethods_Freq_5 | trial_45 | Adj.GoodCountPvals | 0.631523 |
| GSE31210.Surv_AllMethods_Freq_6 | trial_45 | Adj.GoodCountPvals | 0.685348 |
| GSE31210.Surv_AllMethods_Freq_2 | trial_46 | Adj.MedianPvals | 0.613729 |
| GSE31210.Surv_AllMethods_Freq_3 | trial_46 | Adj.MedianPvals | 0.607754 |
| GSE31210.Surv_AllMethods_Freq_4 | trial_46 | Adj.GoodCountPvals | 0.628683 |
| GSE31210.Surv_AllMethods_Freq_5 | trial_46 | Adj.GoodCountPvals | 0.633084 |
| GSE31210.Surv_AllMethods_Freq_6 | trial_46 | Adj.GoodCountPvals | 0.685623 |
| GSE31210.Surv_AllMethods_Freq_2 | trial_47 | Adj.MedianPvals | 0.613729 |
| GSE31210.Surv_AllMethods_Freq_3 | trial_47 | Adj.MedianPvals | 0.607754 |
| GSE31210.Surv_AllMethods_Freq_4 | trial_47 | Adj.GoodCountPvals | 0.624347 |
| GSE31210.Surv_AllMethods_Freq_5 | trial_47 | Adj.GoodCountPvals | 0.630524 |
| GSE31210.Surv_AllMethods_Freq_6 | trial_47 | Adj.GoodCountPvals | 0.685531 |
| GSE31210.Surv_AllMethods_Freq_2 | trial_48 | Adj.MedianPvals | 0.613729 |
| GSE31210.Surv_AllMethods_Freq_3 | trial_48 | Adj.MedianPvals | 0.607754 |
| GSE31210.Surv_AllMethods_Freq_4 | trial_48 | Adj.GoodCountPvals | 0.627586 |
| GSE31210.Surv_AllMethods_Freq_5 | trial_48 | Adj.GoodCountPvals | 0.634395 |
| GSE31210.Surv_AllMethods_Freq_6 | trial_48 | Adj.GoodCountPvals | 0.690018 |
| GSE31210.Surv_AllMethods_Freq_2 | trial_49 | Adj.MedianPvals | 0.613729 |
| GSE31210.Surv_AllMethods_Freq_3 | trial_49 | Adj.MedianPvals | 0.607754 |
| GSE31210.Surv_AllMethods_Freq_4 | trial_49 | Adj.GoodCountPvals | 0.627325 |
| GSE31210.Surv_AllMethods_Freq_5 | trial_49 | Adj.GoodCountPvals | 0.633333 |
| GSE31210.Surv_AllMethods_Freq_6 | trial_49 | Adj.GoodCountPvals | 0.686996 |
| GSE31210.Surv_AllMethods_Freq_2 | trial_50 | Adj.MedianPvals | 0.613729 |
| GSE31210.Surv_AllMethods_Freq_3 | trial_50 | Adj.MedianPvals | 0.607754 |
| GSE31210.Surv_AllMethods_Freq_4 | trial_50 | Adj.GoodCountPvals | 0.625287 |
| GSE31210.Surv_AllMethods_Freq_5 | trial_50 | Adj.GoodCountPvals | 0.632022 |
| GSE31210.Surv_AllMethods_Freq_6 | trial_50 | Adj.GoodCountPvals | 0.686905 |
| GSE31210.Surv_AllMethods_Freq_2 | trial_51 | Adj.MedianPvals | 0.613729 |
| GSE31210.Surv_AllMethods_Freq_3 | trial_51 | Adj.MedianPvals | 0.607754 |
| GSE31210.Surv_AllMethods_Freq_4 | trial_51 | Adj.GoodCountPvals | 0.62581 |
| GSE31210.Surv_AllMethods_Freq_5 | trial_51 | Adj.GoodCountPvals | 0.633333 |
| GSE31210.Surv_AllMethods_Freq_6 | trial_51 | Adj.GoodCountPvals | 0.687821 |
| GSE31210.Surv_AllMethods_Freq_2 | trial_52 | Adj.MedianPvals | 0.613729 |
| GSE31210.Surv_AllMethods_Freq_3 | trial_52 | Adj.MedianPvals | 0.607754 |
| GSE31210.Surv_AllMethods_Freq_4 | trial_52 | Adj.GoodCountPvals | 0.62675 |
| GSE31210.Surv_AllMethods_Freq_5 | trial_52 | Adj.GoodCountPvals | 0.631523 |
| GSE31210.Surv_AllMethods_Freq_6 | trial_52 | Adj.GoodCountPvals | 0.686264 |
| GSE31210.Surv_AllMethods_Freq_2 | trial_53 | Adj.MedianPvals | 0.613729 |
| GSE31210.Surv_AllMethods_Freq_3 | trial_53 | Adj.MedianPvals | 0.607754 |
| GSE31210.Surv_AllMethods_Freq_4 | trial_53 | Adj.GoodCountPvals | 0.627795 |
| GSE31210.Surv_AllMethods_Freq_5 | trial_53 | Adj.GoodCountPvals | 0.634769 |
| GSE31210.Surv_AllMethods_Freq_6 | trial_53 | Adj.GoodCountPvals | 0.68956 |
| GSE31210.Surv_AllMethods_Freq_2 | trial_54 | Adj.MedianPvals | 0.613729 |
| GSE31210.Surv_AllMethods_Freq_3 | trial_54 | Adj.MedianPvals | 0.607754 |
| GSE31210.Surv_AllMethods_Freq_4 | trial_54 | Adj.CORRECTED_P_VALUE | 0.624033 |
| GSE31210.Surv_AllMethods_Freq_5 | trial_54 | Adj.GoodCountPvals | 0.6304 |
| GSE31210.Surv_AllMethods_Freq_6 | trial_54 | Adj.GoodCountPvals | 0.685348 |
| GSE31210.Surv_AllMethods_Freq_2 | trial_55 | Adj.MedianPvals | 0.613729 |
| GSE31210.Surv_AllMethods_Freq_3 | trial_55 | Adj.MedianPvals | 0.607754 |
| GSE31210.Surv_AllMethods_Freq_4 | trial_55 | Adj.CORRECTED_P_VALUE | 0.624033 |
| GSE31210.Surv_AllMethods_Freq_5 | trial_55 | Adj.CORRECTED_P_VALUE | 0.629713 |
| GSE31210.Surv_AllMethods_Freq_6 | trial_55 | Adj.GoodCountPvals | 0.682784 |
| GSE31210.Surv_AllMethods_Freq_2 | trial_56 | Adj.MedianPvals | 0.613729 |
| GSE31210.Surv_AllMethods_Freq_3 | trial_56 | Adj.MedianPvals | 0.607754 |
| GSE31210.Surv_AllMethods_Freq_4 | trial_56 | Adj.GoodCountPvals | 0.62466 |
| GSE31210.Surv_AllMethods_Freq_5 | trial_56 | Adj.GoodCountPvals | 0.631398 |
| GSE31210.Surv_AllMethods_Freq_6 | trial_56 | Adj.GoodCountPvals | 0.687729 |
| GSE31210.Surv_AllMethods_Freq_2 | trial_57 | Adj.MedianPvals | 0.613729 |
| GSE31210.Surv_AllMethods_Freq_3 | trial_57 | Adj.MedianPvals | 0.607754 |
| GSE31210.Surv_AllMethods_Freq_4 | trial_57 | Adj.GoodCountPvals | 0.625078 |
| GSE31210.Surv_AllMethods_Freq_5 | trial_57 | Adj.GoodCountPvals | 0.631336 |
| GSE31210.Surv_AllMethods_Freq_6 | trial_57 | Adj.GoodCountPvals | 0.686905 |
| GSE31210.Surv_AllMethods_Freq_2 | trial_58 | Adj.MedianPvals | 0.613729 |
| GSE31210.Surv_AllMethods_Freq_3 | trial_58 | Adj.MedianPvals | 0.607754 |
| GSE31210.Surv_AllMethods_Freq_4 | trial_58 | Adj.CORRECTED_P_VALUE | 0.624033 |
| GSE31210.Surv_AllMethods_Freq_5 | trial_58 | Adj.CORRECTED_P_VALUE | 0.629713 |
| GSE31210.Surv_AllMethods_Freq_6 | trial_58 | Adj.GoodCountPvals | 0.681685 |
| GSE31210.Surv_AllMethods_Freq_2 | trial_59 | Adj.MedianPvals | 0.613729 |
| GSE31210.Surv_AllMethods_Freq_3 | trial_59 | Adj.MedianPvals | 0.607754 |
| GSE31210.Surv_AllMethods_Freq_4 | trial_59 | Adj.GoodCountPvals | 0.62931 |
| GSE31210.Surv_AllMethods_Freq_5 | trial_59 | Adj.GoodCountPvals | 0.635518 |
| GSE31210.Surv_AllMethods_Freq_6 | trial_59 | Adj.GoodCountPvals | 0.691667 |
| GSE31210.Surv_AllMethods_Freq_2 | trial_60 | Adj.MedianPvals | 0.613729 |
| GSE31210.Surv_AllMethods_Freq_3 | trial_60 | Adj.MedianPvals | 0.607754 |
| GSE31210.Surv_AllMethods_Freq_4 | trial_60 | Adj.GoodCountPvals | 0.62628 |
| GSE31210.Surv_AllMethods_Freq_5 | trial_60 | Adj.GoodCountPvals | 0.63377 |
| GSE31210.Surv_AllMethods_Freq_6 | trial_60 | Adj.GoodCountPvals | 0.686355 |
| GSE31210.Surv_AllMethods_Freq_2 | trial_61 | Adj.GoodCountPvals | 0.613924 |
| GSE31210.Surv_AllMethods_Freq_3 | trial_61 | Adj.MedianPvals | 0.607754 |
| GSE31210.Surv_AllMethods_Freq_4 | trial_61 | Adj.GoodCountPvals | 0.629833 |
| GSE31210.Surv_AllMethods_Freq_5 | trial_61 | Adj.GoodCountPvals | 0.635206 |
| GSE31210.Surv_AllMethods_Freq_6 | trial_61 | Adj.GoodCountPvals | 0.692216 |
| GSE31210.Surv_AllMethods_Freq_2 | trial_62 | Adj.MedianPvals | 0.613729 |
| GSE31210.Surv_AllMethods_Freq_3 | trial_62 | Adj.MedianPvals | 0.607754 |
| GSE31210.Surv_AllMethods_Freq_4 | trial_62 | Adj.GoodCountPvals | 0.625392 |
| GSE31210.Surv_AllMethods_Freq_5 | trial_62 | Adj.GoodCountPvals | 0.632397 |
| GSE31210.Surv_AllMethods_Freq_6 | trial_62 | Adj.GoodCountPvals | 0.684707 |
| GSE31210.Surv_AllMethods_Freq_2 | trial_63 | Adj.MedianPvals | 0.613729 |
| GSE31210.Surv_AllMethods_Freq_3 | trial_63 | Adj.MedianPvals | 0.607754 |
| GSE31210.Surv_AllMethods_Freq_4 | trial_63 | Adj.GoodCountPvals | 0.627064 |
| GSE31210.Surv_AllMethods_Freq_5 | trial_63 | Adj.GoodCountPvals | 0.633208 |
| GSE31210.Surv_AllMethods_Freq_6 | trial_63 | Adj.GoodCountPvals | 0.686355 |
| GSE31210.Surv_AllMethods_Freq_2 | trial_64 | Adj.MedianPvals | 0.613729 |
| GSE31210.Surv_AllMethods_Freq_3 | trial_64 | Adj.MedianPvals | 0.607754 |
| GSE31210.Surv_AllMethods_Freq_4 | trial_64 | Adj.GoodCountPvals | 0.626228 |
| GSE31210.Surv_AllMethods_Freq_5 | trial_64 | Adj.GoodCountPvals | 0.63171 |
| GSE31210.Surv_AllMethods_Freq_6 | trial_64 | Adj.GoodCountPvals | 0.687454 |
| GSE31210.Surv_AllMethods_Freq_2 | trial_65 | Adj.MedianPvals | 0.613729 |
| GSE31210.Surv_AllMethods_Freq_3 | trial_65 | Adj.MedianPvals | 0.607754 |
| GSE31210.Surv_AllMethods_Freq_4 | trial_65 | Adj.GoodCountPvals | 0.626907 |
| GSE31210.Surv_AllMethods_Freq_5 | trial_65 | Adj.GoodCountPvals | 0.632335 |
| GSE31210.Surv_AllMethods_Freq_6 | trial_65 | Adj.GoodCountPvals | 0.685806 |
| GSE31210.Surv_AllMethods_Freq_2 | trial_66 | Adj.MedianPvals | 0.613729 |
| GSE31210.Surv_AllMethods_Freq_3 | trial_66 | Adj.MedianPvals | 0.607754 |
| GSE31210.Surv_AllMethods_Freq_4 | trial_66 | Adj.GoodCountPvals | 0.624765 |
| GSE31210.Surv_AllMethods_Freq_5 | trial_66 | Adj.GoodCountPvals | 0.630649 |
| GSE31210.Surv_AllMethods_Freq_6 | trial_66 | Adj.GoodCountPvals | 0.686447 |
| GSE31210.Surv_AllMethods_Freq_2 | trial_67 | Adj.MedianPvals | 0.613729 |
| GSE31210.Surv_AllMethods_Freq_3 | trial_67 | Adj.MedianPvals | 0.607754 |
| GSE31210.Surv_AllMethods_Freq_4 | trial_67 | Adj.GoodCountPvals | 0.627168 |
| GSE31210.Surv_AllMethods_Freq_5 | trial_67 | Adj.GoodCountPvals | 0.634582 |
| GSE31210.Surv_AllMethods_Freq_6 | trial_67 | Adj.GoodCountPvals | 0.690568 |
| GSE31210.Surv_AllMethods_Freq_2 | trial_68 | Adj.MedianPvals | 0.613729 |
| GSE31210.Surv_AllMethods_Freq_3 | trial_68 | Adj.MedianPvals | 0.607754 |
| GSE31210.Surv_AllMethods_Freq_4 | trial_68 | Adj.GoodCountPvals | 0.627273 |
| GSE31210.Surv_AllMethods_Freq_5 | trial_68 | Adj.GoodCountPvals | 0.633084 |
| GSE31210.Surv_AllMethods_Freq_6 | trial_68 | Adj.GoodCountPvals | 0.689286 |
| GSE31210.Surv_AllMethods_Freq_2 | trial_69 | Adj.MedianPvals | 0.613729 |
| GSE31210.Surv_AllMethods_Freq_3 | trial_69 | Adj.MedianPvals | 0.607754 |
| GSE31210.Surv_AllMethods_Freq_4 | trial_69 | Adj.GoodCountPvals | 0.626176 |
| GSE31210.Surv_AllMethods_Freq_5 | trial_69 | Adj.GoodCountPvals | 0.631898 |
| GSE31210.Surv_AllMethods_Freq_6 | trial_69 | Adj.GoodCountPvals | 0.686447 |
| GSE31210.Surv_AllMethods_Freq_2 | trial_70 | Adj.MedianPvals | 0.613729 |
| GSE31210.Surv_AllMethods_Freq_3 | trial_70 | Adj.MedianPvals | 0.607754 |
| GSE31210.Surv_AllMethods_Freq_4 | trial_70 | Adj.GoodCountPvals | 0.626071 |
| GSE31210.Surv_AllMethods_Freq_5 | trial_70 | Adj.GoodCountPvals | 0.634332 |
| GSE31210.Surv_AllMethods_Freq_6 | trial_70 | Adj.GoodCountPvals | 0.688095 |
| GSE31210.Surv_AllMethods_Freq_2 | trial_71 | Adj.MedianPvals | 0.613729 |
| GSE31210.Surv_AllMethods_Freq_3 | trial_71 | Adj.MedianPvals | 0.607754 |
| GSE31210.Surv_AllMethods_Freq_4 | trial_71 | Adj.GoodCountPvals | 0.62466 |
| GSE31210.Surv_AllMethods_Freq_5 | trial_71 | Adj.CORRECTED_P_VALUE | 0.629713 |
| GSE31210.Surv_AllMethods_Freq_6 | trial_71 | Adj.GoodCountPvals | 0.68315 |
| GSE31210.Surv_AllMethods_Freq_2 | trial_72 | Adj.MedianPvals | 0.613729 |
| GSE31210.Surv_AllMethods_Freq_3 | trial_72 | Adj.MedianPvals | 0.607754 |
| GSE31210.Surv_AllMethods_Freq_4 | trial_72 | Adj.CORRECTED_P_VALUE | 0.624033 |
| GSE31210.Surv_AllMethods_Freq_5 | trial_72 | Adj.GoodCountPvals:Adj.CORRECTED_P_VALUE | 0.629713 |
| GSE31210.Surv_AllMethods_Freq_6 | trial_72 | Adj.GoodCountPvals | 0.680861 |
| GSE31210.Surv_AllMethods_Freq_2 | trial_73 | Adj.MedianPvals | 0.613729 |
| GSE31210.Surv_AllMethods_Freq_3 | trial_73 | Adj.MedianPvals | 0.607754 |
| GSE31210.Surv_AllMethods_Freq_4 | trial_73 | Adj.CORRECTED_P_VALUE | 0.624033 |
| GSE31210.Surv_AllMethods_Freq_5 | trial_73 | Adj.CORRECTED_P_VALUE | 0.629713 |
| GSE31210.Surv_AllMethods_Freq_6 | trial_73 | Adj.GoodCountPvals | 0.682601 |
| GSE31210.Surv_AllMethods_Freq_2 | trial_74 | Adj.MedianPvals | 0.613729 |
| GSE31210.Surv_AllMethods_Freq_3 | trial_74 | Adj.MedianPvals | 0.607754 |
| GSE31210.Surv_AllMethods_Freq_4 | trial_74 | Adj.GoodCountPvals | 0.62675 |
| GSE31210.Surv_AllMethods_Freq_5 | trial_74 | Adj.GoodCountPvals | 0.633458 |
| GSE31210.Surv_AllMethods_Freq_6 | trial_74 | Adj.GoodCountPvals | 0.687271 |
| GSE31210.Surv_AllMethods_Freq_2 | trial_75 | Adj.MedianPvals | 0.613729 |
| GSE31210.Surv_AllMethods_Freq_3 | trial_75 | Adj.MedianPvals | 0.607754 |
| GSE31210.Surv_AllMethods_Freq_4 | trial_75 | Adj.GoodCountPvals | 0.62884 |
| GSE31210.Surv_AllMethods_Freq_5 | trial_75 | Adj.GoodCountPvals | 0.634457 |
| GSE31210.Surv_AllMethods_Freq_6 | trial_75 | Adj.GoodCountPvals | 0.69011 |
| GSE31210.Surv_AllMethods_Freq_2 | trial_76 | Adj.MedianPvals | 0.613729 |
| GSE31210.Surv_AllMethods_Freq_3 | trial_76 | Adj.MedianPvals | 0.607754 |
| GSE31210.Surv_AllMethods_Freq_4 | trial_76 | Adj.GoodCountPvals | 0.627325 |
| GSE31210.Surv_AllMethods_Freq_5 | trial_76 | Adj.GoodCountPvals | 0.632772 |
| GSE31210.Surv_AllMethods_Freq_6 | trial_76 | Adj.GoodCountPvals | 0.685256 |
| GSE31210.Surv_AllMethods_Freq_2 | trial_77 | Adj.MedianPvals | 0.613729 |
| GSE31210.Surv_AllMethods_Freq_3 | trial_77 | Adj.MedianPvals | 0.607754 |
| GSE31210.Surv_AllMethods_Freq_4 | trial_77 | Adj.GoodCountPvals | 0.624451 |
| GSE31210.Surv_AllMethods_Freq_5 | trial_77 | Adj.GoodCountPvals | 0.630649 |
| GSE31210.Surv_AllMethods_Freq_6 | trial_77 | Adj.GoodCountPvals | 0.685073 |
| GSE31210.Surv_AllMethods_Freq_2 | trial_78 | Adj.MedianPvals | 0.613729 |
| GSE31210.Surv_AllMethods_Freq_3 | trial_78 | Adj.MedianPvals | 0.607754 |
| GSE31210.Surv_AllMethods_Freq_4 | trial_78 | Adj.GoodCountPvals | 0.625131 |
| GSE31210.Surv_AllMethods_Freq_5 | trial_78 | Adj.GoodCountPvals | 0.631898 |
| GSE31210.Surv_AllMethods_Freq_6 | trial_78 | Adj.GoodCountPvals | 0.686264 |
| GSE31210.Surv_AllMethods_Freq_2 | trial_79 | Adj.MedianPvals | 0.613729 |
| GSE31210.Surv_AllMethods_Freq_3 | trial_79 | Adj.MedianPvals | 0.607754 |
| GSE31210.Surv_AllMethods_Freq_4 | trial_79 | Adj.GoodCountPvals | 0.626541 |
| GSE31210.Surv_AllMethods_Freq_5 | trial_79 | Adj.GoodCountPvals | 0.63402 |
| GSE31210.Surv_AllMethods_Freq_6 | trial_79 | Adj.GoodCountPvals | 0.688187 |
| GSE31210.Surv_AllMethods_Freq_2 | trial_80 | Adj.MedianPvals | 0.613729 |
| GSE31210.Surv_AllMethods_Freq_3 | trial_80 | Adj.MedianPvals | 0.607754 |
| GSE31210.Surv_AllMethods_Freq_4 | trial_80 | Adj.CORRECTED_P_VALUE | 0.624033 |
| GSE31210.Surv_AllMethods_Freq_5 | trial_80 | Adj.CORRECTED_P_VALUE | 0.629713 |
| GSE31210.Surv_AllMethods_Freq_6 | trial_80 | Adj.GoodCountPvals | 0.684158 |
| GSE31210.Surv_AllMethods_Freq_2 | trial_81 | Adj.MedianPvals | 0.613729 |
| GSE31210.Surv_AllMethods_Freq_3 | trial_81 | Adj.MedianPvals | 0.607754 |
| GSE31210.Surv_AllMethods_Freq_4 | trial_81 | Adj.GoodCountPvals | 0.624974 |
| GSE31210.Surv_AllMethods_Freq_5 | trial_81 | Adj.GoodCountPvals | 0.632959 |
| GSE31210.Surv_AllMethods_Freq_6 | trial_81 | Adj.GoodCountPvals | 0.684158 |
| GSE31210.Surv_AllMethods_Freq_2 | trial_82 | Adj.MedianPvals | 0.613729 |
| GSE31210.Surv_AllMethods_Freq_3 | trial_82 | Adj.MedianPvals | 0.607754 |
| GSE31210.Surv_AllMethods_Freq_4 | trial_82 | Adj.GoodCountPvals | 0.62628 |
| GSE31210.Surv_AllMethods_Freq_5 | trial_82 | Adj.GoodCountPvals | 0.633958 |
| GSE31210.Surv_AllMethods_Freq_6 | trial_82 | Adj.GoodCountPvals | 0.68837 |
| GSE31210.Surv_AllMethods_Freq_2 | trial_83 | Adj.GoodCountPvals | 0.614752 |
| GSE31210.Surv_AllMethods_Freq_3 | trial_83 | Adj.MedianPvals | 0.607754 |
| GSE31210.Surv_AllMethods_Freq_4 | trial_83 | Adj.GoodCountPvals | 0.629676 |
| GSE31210.Surv_AllMethods_Freq_5 | trial_83 | Adj.GoodCountPvals | 0.635019 |
| GSE31210.Surv_AllMethods_Freq_6 | trial_83 | Adj.GoodCountPvals | 0.690476 |
| GSE31210.Surv_AllMethods_Freq_2 | trial_84 | Adj.MedianPvals | 0.613729 |
| GSE31210.Surv_AllMethods_Freq_3 | trial_84 | Adj.MedianPvals | 0.607754 |
| GSE31210.Surv_AllMethods_Freq_4 | trial_84 | Adj.CORRECTED_P_VALUE | 0.624033 |
| GSE31210.Surv_AllMethods_Freq_5 | trial_84 | Adj.CORRECTED_P_VALUE | 0.629713 |
| GSE31210.Surv_AllMethods_Freq_6 | trial_84 | Adj.GoodCountPvals | 0.684158 |
| GSE31210.Surv_AllMethods_Freq_2 | trial_85 | Adj.MedianPvals | 0.613729 |
| GSE31210.Surv_AllMethods_Freq_3 | trial_85 | Adj.MedianPvals | 0.607754 |
| GSE31210.Surv_AllMethods_Freq_4 | trial_85 | Adj.GoodCountPvals | 0.626803 |
| GSE31210.Surv_AllMethods_Freq_5 | trial_85 | Adj.GoodCountPvals | 0.63221 |
| GSE31210.Surv_AllMethods_Freq_6 | trial_85 | Adj.GoodCountPvals | 0.686447 |
| GSE31210.Surv_AllMethods_Freq_2 | trial_86 | Adj.MedianPvals | 0.613729 |
| GSE31210.Surv_AllMethods_Freq_3 | trial_86 | Adj.MedianPvals | 0.607754 |
| GSE31210.Surv_AllMethods_Freq_4 | trial_86 | Adj.CORRECTED_P_VALUE | 0.624033 |
| GSE31210.Surv_AllMethods_Freq_5 | trial_86 | Adj.GoodCountPvals | 0.629963 |
| GSE31210.Surv_AllMethods_Freq_6 | trial_86 | Adj.GoodCountPvals | 0.680037 |
| GSE31210.Surv_AllMethods_Freq_2 | trial_87 | Adj.MedianPvals | 0.613729 |
| GSE31210.Surv_AllMethods_Freq_3 | trial_87 | Adj.MedianPvals | 0.607754 |
| GSE31210.Surv_AllMethods_Freq_4 | trial_87 | Adj.GoodCountPvals | 0.624451 |
| GSE31210.Surv_AllMethods_Freq_5 | trial_87 | Adj.CORRECTED_P_VALUE | 0.629713 |
| GSE31210.Surv_AllMethods_Freq_6 | trial_87 | Adj.GoodCountPvals | 0.68141 |
| GSE31210.Surv_AllMethods_Freq_2 | trial_88 | Adj.MedianPvals | 0.613729 |
| GSE31210.Surv_AllMethods_Freq_3 | trial_88 | Adj.MedianPvals | 0.607754 |
| GSE31210.Surv_AllMethods_Freq_4 | trial_88 | Adj.GoodCountPvals | 0.628056 |
| GSE31210.Surv_AllMethods_Freq_5 | trial_88 | Adj.GoodCountPvals | 0.635955 |
| GSE31210.Surv_AllMethods_Freq_6 | trial_88 | Adj.GoodCountPvals | 0.689927 |
| GSE31210.Surv_AllMethods_Freq_2 | trial_89 | Adj.MedianPvals | 0.613729 |
| GSE31210.Surv_AllMethods_Freq_3 | trial_89 | Adj.MedianPvals | 0.607754 |
| GSE31210.Surv_AllMethods_Freq_4 | trial_89 | Adj.GoodCountPvals:Adj.CORRECTED_P_VALUE | 0.624033 |
| GSE31210.Surv_AllMethods_Freq_5 | trial_89 | Adj.CORRECTED_P_VALUE | 0.629713 |
| GSE31210.Surv_AllMethods_Freq_6 | trial_89 | Adj.GoodCountPvals | 0.682143 |
| GSE31210.Surv_AllMethods_Freq_2 | trial_90 | Adj.MedianPvals | 0.613729 |
| GSE31210.Surv_AllMethods_Freq_3 | trial_90 | Adj.MedianPvals | 0.607754 |
| GSE31210.Surv_AllMethods_Freq_4 | trial_90 | Adj.GoodCountPvals | 0.627116 |
| GSE31210.Surv_AllMethods_Freq_5 | trial_90 | Adj.GoodCountPvals | 0.633958 |
| GSE31210.Surv_AllMethods_Freq_6 | trial_90 | Adj.GoodCountPvals | 0.692857 |
| GSE31210.Surv_AllMethods_Freq_2 | trial_91 | Adj.MedianPvals | 0.613729 |
| GSE31210.Surv_AllMethods_Freq_3 | trial_91 | Adj.MedianPvals | 0.607754 |
| GSE31210.Surv_AllMethods_Freq_4 | trial_91 | Adj.GoodCountPvals | 0.625758 |
| GSE31210.Surv_AllMethods_Freq_5 | trial_91 | Adj.GoodCountPvals | 0.632896 |
| GSE31210.Surv_AllMethods_Freq_6 | trial_91 | Adj.GoodCountPvals | 0.685348 |
| GSE31210.Surv_AllMethods_Freq_2 | trial_92 | Adj.MedianPvals | 0.613729 |
| GSE31210.Surv_AllMethods_Freq_3 | trial_92 | Adj.MedianPvals | 0.607754 |
| GSE31210.Surv_AllMethods_Freq_4 | trial_92 | Adj.GoodCountPvals | 0.628213 |
| GSE31210.Surv_AllMethods_Freq_5 | trial_92 | Adj.GoodCountPvals | 0.633458 |
| GSE31210.Surv_AllMethods_Freq_6 | trial_92 | Adj.GoodCountPvals | 0.687546 |
| GSE31210.Surv_AllMethods_Freq_2 | trial_93 | Adj.MedianPvals | 0.613729 |
| GSE31210.Surv_AllMethods_Freq_3 | trial_93 | Adj.MedianPvals | 0.607754 |
| GSE31210.Surv_AllMethods_Freq_4 | trial_93 | Adj.GoodCountPvals | 0.624399 |
| GSE31210.Surv_AllMethods_Freq_5 | trial_93 | Adj.GoodCountPvals | 0.631086 |
| GSE31210.Surv_AllMethods_Freq_6 | trial_93 | Adj.GoodCountPvals | 0.68489 |
| GSE31210.Surv_AllMethods_Freq_2 | trial_94 | Adj.MedianPvals | 0.613729 |
| GSE31210.Surv_AllMethods_Freq_3 | trial_94 | Adj.MedianPvals | 0.607754 |
| GSE31210.Surv_AllMethods_Freq_4 | trial_94 | Adj.GoodCountPvals | 0.624974 |
| GSE31210.Surv_AllMethods_Freq_5 | trial_94 | Adj.GoodCountPvals | 0.631461 |
| GSE31210.Surv_AllMethods_Freq_6 | trial_94 | Adj.GoodCountPvals | 0.684982 |
| GSE31210.Surv_AllMethods_Freq_2 | trial_95 | Adj.GoodCountPvals | 0.613827 |
| GSE31210.Surv_AllMethods_Freq_3 | trial_95 | Adj.MedianPvals | 0.607754 |
| GSE31210.Surv_AllMethods_Freq_4 | trial_95 | Adj.GoodCountPvals | 0.629101 |
| GSE31210.Surv_AllMethods_Freq_5 | trial_95 | Adj.GoodCountPvals | 0.636454 |
| GSE31210.Surv_AllMethods_Freq_6 | trial_95 | Adj.GoodCountPvals | 0.690476 |
| GSE31210.Surv_AllMethods_Freq_2 | trial_96 | Adj.MedianPvals | 0.613729 |
| GSE31210.Surv_AllMethods_Freq_3 | trial_96 | Adj.MedianPvals | 0.607754 |
| GSE31210.Surv_AllMethods_Freq_4 | trial_96 | Adj.GoodCountPvals | 0.62466 |
| GSE31210.Surv_AllMethods_Freq_5 | trial_96 | Adj.GoodCountPvals | 0.629963 |
| GSE31210.Surv_AllMethods_Freq_6 | trial_96 | Adj.GoodCountPvals | 0.686264 |
| GSE31210.Surv_AllMethods_Freq_2 | trial_97 | Adj.MedianPvals | 0.613729 |
| GSE31210.Surv_AllMethods_Freq_3 | trial_97 | Adj.MedianPvals | 0.607754 |
| GSE31210.Surv_AllMethods_Freq_4 | trial_97 | Adj.CORRECTED_P_VALUE | 0.624033 |
| GSE31210.Surv_AllMethods_Freq_5 | trial_97 | Adj.GoodCountPvals | 0.631149 |
| GSE31210.Surv_AllMethods_Freq_6 | trial_97 | Adj.GoodCountPvals | 0.686264 |
| GSE31210.Surv_AllMethods_Freq_2 | trial_98 | Adj.MedianPvals | 0.613729 |
| GSE31210.Surv_AllMethods_Freq_3 | trial_98 | Adj.MedianPvals | 0.607754 |
| GSE31210.Surv_AllMethods_Freq_4 | trial_98 | Adj.GoodCountPvals | 0.626646 |
| GSE31210.Surv_AllMethods_Freq_5 | trial_98 | Adj.GoodCountPvals | 0.630774 |
| GSE31210.Surv_AllMethods_Freq_6 | trial_98 | Adj.GoodCountPvals | 0.686447 |
| GSE31210.Surv_AllMethods_Freq_2 | trial_99 | Adj.MedianPvals | 0.613729 |
| GSE31210.Surv_AllMethods_Freq_3 | trial_99 | Adj.MedianPvals | 0.607754 |
| GSE31210.Surv_AllMethods_Freq_4 | trial_99 | Adj.GoodCountPvals | 0.62419 |
| GSE31210.Surv_AllMethods_Freq_5 | trial_99 | Adj.GoodCountPvals | 0.631024 |
| GSE31210.Surv_AllMethods_Freq_6 | trial_99 | Adj.GoodCountPvals | 0.683791 |
| GSE31210.Surv_AllMethods_Freq_2 | trial_100 | Adj.MedianPvals | 0.613729 |
| GSE31210.Surv_AllMethods_Freq_3 | trial_100 | Adj.MedianPvals | 0.607754 |
| GSE31210.Surv_AllMethods_Freq_4 | trial_100 | Adj.CORRECTED_P_VALUE | 0.624033 |
| GSE31210.Surv_AllMethods_Freq_5 | trial_100 | Adj.CORRECTED_P_VALUE | 0.629713 |
| GSE31210.Surv_AllMethods_Freq_6 | trial_100 | Adj.GoodCountPvals | 0.686264 |
| GSE31210.SurvRelapseFree_AllMethods_Freq_2 | trial_1 | Adj.GoodCountPvals | 0.567756 |
| GSE31210.SurvRelapseFree_AllMethods_Freq_3 | trial_1 | Adj.GoodCountPvals | 0.578062 |
| GSE31210.SurvRelapseFree_AllMethods_Freq_4 | trial_1 | Adj.GoodCountPvals | 0.575201 |
| GSE31210.SurvRelapseFree_AllMethods_Freq_5 | trial_1 | Adj.GoodCountPvals:Adj.MedianPvals | 0.585567 |
| GSE31210.SurvRelapseFree_AllMethods_Freq_6 | trial_1 | Adj.GoodCountPvals | 0.616586 |
| GSE31210.SurvRelapseFree_AllMethods_Freq_2 | trial_2 | Adj.GoodCountPvals | 0.565981 |
| GSE31210.SurvRelapseFree_AllMethods_Freq_3 | trial_2 | Adj.GoodCountPvals | 0.576734 |
| GSE31210.SurvRelapseFree_AllMethods_Freq_4 | trial_2 | Adj.GoodCountPvals | 0.572786 |
| GSE31210.SurvRelapseFree_AllMethods_Freq_5 | trial_2 | Adj.MedianPvals | 0.585567 |
| GSE31210.SurvRelapseFree_AllMethods_Freq_6 | trial_2 | Adj.GoodCountPvals | 0.614438 |
| GSE31210.SurvRelapseFree_AllMethods_Freq_2 | trial_3 | Adj.GoodCountPvals | 0.570541 |
| GSE31210.SurvRelapseFree_AllMethods_Freq_3 | trial_3 | Adj.GoodCountPvals | 0.580517 |
| GSE31210.SurvRelapseFree_AllMethods_Freq_4 | trial_3 | Adj.GoodCountPvals | 0.576973 |
| GSE31210.SurvRelapseFree_AllMethods_Freq_5 | trial_3 | Adj.GoodCountPvals | 0.587171 |
| GSE31210.SurvRelapseFree_AllMethods_Freq_6 | trial_3 | Adj.GoodCountPvals | 0.617481 |
| GSE31210.SurvRelapseFree_AllMethods_Freq_2 | trial_4 | Adj.GoodCountPvals | 0.569169 |
| GSE31210.SurvRelapseFree_AllMethods_Freq_3 | trial_4 | Adj.GoodCountPvals | 0.579068 |
| GSE31210.SurvRelapseFree_AllMethods_Freq_4 | trial_4 | Adj.GoodCountPvals | 0.575161 |
| GSE31210.SurvRelapseFree_AllMethods_Freq_5 | trial_4 | Adj.MedianPvals | 0.585567 |
| GSE31210.SurvRelapseFree_AllMethods_Freq_6 | trial_4 | Adj.GoodCountPvals | 0.616676 |
| GSE31210.SurvRelapseFree_AllMethods_Freq_2 | trial_5 | Adj.GoodCountPvals | 0.567514 |
| GSE31210.SurvRelapseFree_AllMethods_Freq_3 | trial_5 | Adj.GoodCountPvals | 0.57762 |
| GSE31210.SurvRelapseFree_AllMethods_Freq_4 | trial_5 | Adj.GoodCountPvals | 0.574114 |
| GSE31210.SurvRelapseFree_AllMethods_Freq_5 | trial_5 | Adj.MedianPvals | 0.585567 |
| GSE31210.SurvRelapseFree_AllMethods_Freq_6 | trial_5 | Adj.GoodCountPvals | 0.616407 |
| GSE31210.SurvRelapseFree_AllMethods_Freq_2 | trial_6 | Adj.GoodCountPvals | 0.568079 |
| GSE31210.SurvRelapseFree_AllMethods_Freq_3 | trial_6 | Adj.GoodCountPvals | 0.578505 |
| GSE31210.SurvRelapseFree_AllMethods_Freq_4 | trial_6 | Adj.GoodCountPvals | 0.575805 |
| GSE31210.SurvRelapseFree_AllMethods_Freq_5 | trial_6 | Adj.GoodCountPvals | 0.586513 |
| GSE31210.SurvRelapseFree_AllMethods_Freq_6 | trial_6 | Adj.GoodCountPvals | 0.617123 |
| GSE31210.SurvRelapseFree_AllMethods_Freq_2 | trial_7 | Adj.GoodCountPvals | 0.567837 |
| GSE31210.SurvRelapseFree_AllMethods_Freq_3 | trial_7 | Adj.GoodCountPvals | 0.577982 |
| GSE31210.SurvRelapseFree_AllMethods_Freq_4 | trial_7 | Adj.GoodCountPvals | 0.573792 |
| GSE31210.SurvRelapseFree_AllMethods_Freq_5 | trial_7 | Adj.MedianPvals | 0.585567 |
| GSE31210.SurvRelapseFree_AllMethods_Freq_6 | trial_7 | Adj.GoodCountPvals | 0.615154 |
| GSE31210.SurvRelapseFree_AllMethods_Freq_2 | trial_8 | Adj.GoodCountPvals | 0.566425 |
| GSE31210.SurvRelapseFree_AllMethods_Freq_3 | trial_8 | Adj.GoodCountPvals | 0.576614 |
| GSE31210.SurvRelapseFree_AllMethods_Freq_4 | trial_8 | Adj.GoodCountPvals | 0.572343 |
| GSE31210.SurvRelapseFree_AllMethods_Freq_5 | trial_8 | Adj.MedianPvals | 0.585567 |
| GSE31210.SurvRelapseFree_AllMethods_Freq_6 | trial_8 | Adj.GoodCountPvals | 0.615064 |
| GSE31210.SurvRelapseFree_AllMethods_Freq_2 | trial_9 | Adj.GoodCountPvals | 0.569169 |
| GSE31210.SurvRelapseFree_AllMethods_Freq_3 | trial_9 | Adj.GoodCountPvals | 0.579913 |
| GSE31210.SurvRelapseFree_AllMethods_Freq_4 | trial_9 | Adj.GoodCountPvals | 0.575926 |
| GSE31210.SurvRelapseFree_AllMethods_Freq_5 | trial_9 | Adj.MedianPvals | 0.585567 |
| GSE31210.SurvRelapseFree_AllMethods_Freq_6 | trial_9 | Adj.GoodCountPvals | 0.616318 |
| GSE31210.SurvRelapseFree_AllMethods_Freq_2 | trial_10 | Adj.GoodCountPvals | 0.568483 |
| GSE31210.SurvRelapseFree_AllMethods_Freq_3 | trial_10 | Adj.GoodCountPvals | 0.578143 |
| GSE31210.SurvRelapseFree_AllMethods_Freq_4 | trial_10 | Adj.GoodCountPvals | 0.574316 |
| GSE31210.SurvRelapseFree_AllMethods_Freq_5 | trial_10 | Adj.MedianPvals | 0.585567 |
| GSE31210.SurvRelapseFree_AllMethods_Freq_6 | trial_10 | Adj.GoodCountPvals | 0.614572 |
| GSE31210.SurvRelapseFree_AllMethods_Freq_2 | trial_11 | Adj.GoodCountPvals | 0.56937 |
| GSE31210.SurvRelapseFree_AllMethods_Freq_3 | trial_11 | Adj.GoodCountPvals | 0.579953 |
| GSE31210.SurvRelapseFree_AllMethods_Freq_4 | trial_11 | Adj.GoodCountPvals | 0.577134 |
| GSE31210.SurvRelapseFree_AllMethods_Freq_5 | trial_11 | Adj.GoodCountPvals | 0.587788 |
| GSE31210.SurvRelapseFree_AllMethods_Freq_6 | trial_11 | Adj.GoodCountPvals | 0.619048 |
| GSE31210.SurvRelapseFree_AllMethods_Freq_2 | trial_12 | Adj.GoodCountPvals | 0.568523 |
| GSE31210.SurvRelapseFree_AllMethods_Freq_3 | trial_12 | Adj.GoodCountPvals | 0.579028 |
| GSE31210.SurvRelapseFree_AllMethods_Freq_4 | trial_12 | Adj.GoodCountPvals | 0.574799 |
| GSE31210.SurvRelapseFree_AllMethods_Freq_5 | trial_12 | Adj.MedianPvals | 0.585567 |
| GSE31210.SurvRelapseFree_AllMethods_Freq_6 | trial_12 | Adj.GoodCountPvals | 0.614841 |
| GSE31210.SurvRelapseFree_AllMethods_Freq_2 | trial_13 | Adj.GoodCountPvals | 0.565456 |
| GSE31210.SurvRelapseFree_AllMethods_Freq_3 | trial_13 | Adj.GoodCountPvals | 0.575769 |
| GSE31210.SurvRelapseFree_AllMethods_Freq_4 | trial_13 | Adj.GoodCountPvals | 0.572303 |
| GSE31210.SurvRelapseFree_AllMethods_Freq_5 | trial_13 | Adj.MedianPvals | 0.585567 |
| GSE31210.SurvRelapseFree_AllMethods_Freq_6 | trial_13 | Adj.GoodCountPvals | 0.614662 |
| GSE31210.SurvRelapseFree_AllMethods_Freq_2 | trial_14 | Adj.GoodCountPvals | 0.568039 |
| GSE31210.SurvRelapseFree_AllMethods_Freq_3 | trial_14 | Adj.GoodCountPvals | 0.578545 |
| GSE31210.SurvRelapseFree_AllMethods_Freq_4 | trial_14 | Adj.GoodCountPvals | 0.57496 |
| GSE31210.SurvRelapseFree_AllMethods_Freq_5 | trial_14 | Adj.MedianPvals | 0.585567 |
| GSE31210.SurvRelapseFree_AllMethods_Freq_6 | trial_14 | Adj.GoodCountPvals | 0.614796 |
| GSE31210.SurvRelapseFree_AllMethods_Freq_2 | trial_15 | Adj.GoodCountPvals | 0.566586 |
| GSE31210.SurvRelapseFree_AllMethods_Freq_3 | trial_15 | Adj.GoodCountPvals | 0.576412 |
| GSE31210.SurvRelapseFree_AllMethods_Freq_4 | trial_15 | Adj.GoodCountPvals | 0.573349 |
| GSE31210.SurvRelapseFree_AllMethods_Freq_5 | trial_15 | Adj.MedianPvals | 0.585567 |
| GSE31210.SurvRelapseFree_AllMethods_Freq_6 | trial_15 | Adj.GoodCountPvals | 0.613767 |
| GSE31210.SurvRelapseFree_AllMethods_Freq_2 | trial_16 | Adj.GoodCountPvals | 0.56816 |
| GSE31210.SurvRelapseFree_AllMethods_Freq_3 | trial_16 | Adj.GoodCountPvals | 0.578545 |
| GSE31210.SurvRelapseFree_AllMethods_Freq_4 | trial_16 | Adj.GoodCountPvals | 0.574638 |
| GSE31210.SurvRelapseFree_AllMethods_Freq_5 | trial_16 | Adj.MedianPvals | 0.585567 |
| GSE31210.SurvRelapseFree_AllMethods_Freq_6 | trial_16 | Adj.GoodCountPvals | 0.616541 |
| GSE31210.SurvRelapseFree_AllMethods_Freq_2 | trial_17 | Adj.GoodCountPvals | 0.56929 |
| GSE31210.SurvRelapseFree_AllMethods_Freq_3 | trial_17 | Adj.GoodCountPvals | 0.579229 |
| GSE31210.SurvRelapseFree_AllMethods_Freq_4 | trial_17 | Adj.GoodCountPvals | 0.57649 |
| GSE31210.SurvRelapseFree_AllMethods_Freq_5 | trial_17 | Adj.GoodCountPvals | 0.586637 |
| GSE31210.SurvRelapseFree_AllMethods_Freq_6 | trial_17 | Adj.GoodCountPvals | 0.61672 |
| GSE31210.SurvRelapseFree_AllMethods_Freq_2 | trial_18 | Adj.GoodCountPvals | 0.567111 |
| GSE31210.SurvRelapseFree_AllMethods_Freq_3 | trial_18 | Adj.GoodCountPvals | 0.577298 |
| GSE31210.SurvRelapseFree_AllMethods_Freq_4 | trial_18 | Adj.GoodCountPvals | 0.573108 |
| GSE31210.SurvRelapseFree_AllMethods_Freq_5 | trial_18 | Adj.MedianPvals | 0.585567 |
| GSE31210.SurvRelapseFree_AllMethods_Freq_6 | trial_18 | Adj.GoodCountPvals | 0.616228 |
| GSE31210.SurvRelapseFree_AllMethods_Freq_2 | trial_19 | Adj.GoodCountPvals | 0.565981 |
| GSE31210.SurvRelapseFree_AllMethods_Freq_3 | trial_19 | Adj.GoodCountPvals | 0.576654 |
| GSE31210.SurvRelapseFree_AllMethods_Freq_4 | trial_19 | Adj.GoodCountPvals | 0.573873 |
| GSE31210.SurvRelapseFree_AllMethods_Freq_5 | trial_19 | Adj.MedianPvals | 0.585567 |
| GSE31210.SurvRelapseFree_AllMethods_Freq_6 | trial_19 | Adj.GoodCountPvals | 0.614125 |
| GSE31210.SurvRelapseFree_AllMethods_Freq_2 | trial_20 | Adj.GoodCountPvals | 0.567474 |
| GSE31210.SurvRelapseFree_AllMethods_Freq_3 | trial_20 | Adj.GoodCountPvals | 0.577941 |
| GSE31210.SurvRelapseFree_AllMethods_Freq_4 | trial_20 | Adj.GoodCountPvals | 0.574074 |
| GSE31210.SurvRelapseFree_AllMethods_Freq_5 | trial_20 | Adj.MedianPvals | 0.585567 |
| GSE31210.SurvRelapseFree_AllMethods_Freq_6 | trial_20 | Adj.GoodCountPvals | 0.614841 |
| GSE31210.SurvRelapseFree_AllMethods_Freq_2 | trial_21 | Adj.GoodCountPvals | 0.565052 |
| GSE31210.SurvRelapseFree_AllMethods_Freq_3 | trial_21 | Adj.GoodCountPvals | 0.575205 |
| GSE31210.SurvRelapseFree_AllMethods_Freq_4 | trial_21 | Adj.GoodCountPvals | 0.572262 |
| GSE31210.SurvRelapseFree_AllMethods_Freq_5 | trial_21 | Adj.MedianPvals | 0.585567 |
| GSE31210.SurvRelapseFree_AllMethods_Freq_6 | trial_21 | Adj.GoodCountPvals | 0.61399 |
| GSE31210.SurvRelapseFree_AllMethods_Freq_2 | trial_22 | Adj.GoodCountPvals | 0.566586 |
| GSE31210.SurvRelapseFree_AllMethods_Freq_3 | trial_22 | Adj.GoodCountPvals | 0.576694 |
| GSE31210.SurvRelapseFree_AllMethods_Freq_4 | trial_22 | Adj.GoodCountPvals | 0.574074 |
| GSE31210.SurvRelapseFree_AllMethods_Freq_5 | trial_22 | Adj.MedianPvals | 0.585567 |
| GSE31210.SurvRelapseFree_AllMethods_Freq_6 | trial_22 | Adj.GoodCountPvals | 0.612513 |
| GSE31210.SurvRelapseFree_AllMethods_Freq_2 | trial_23 | Adj.GoodCountPvals | 0.566303 |
| GSE31210.SurvRelapseFree_AllMethods_Freq_3 | trial_23 | Adj.GoodCountPvals | 0.576694 |
| GSE31210.SurvRelapseFree_AllMethods_Freq_4 | trial_23 | Adj.GoodCountPvals | 0.573953 |
| GSE31210.SurvRelapseFree_AllMethods_Freq_5 | trial_23 | Adj.MedianPvals | 0.585567 |
| GSE31210.SurvRelapseFree_AllMethods_Freq_6 | trial_23 | Adj.GoodCountPvals | 0.617078 |
| GSE31210.SurvRelapseFree_AllMethods_Freq_2 | trial_24 | Adj.GoodCountPvals | 0.5659 |
| GSE31210.SurvRelapseFree_AllMethods_Freq_3 | trial_24 | Adj.GoodCountPvals | 0.576332 |
| GSE31210.SurvRelapseFree_AllMethods_Freq_4 | trial_24 | Adj.GoodCountPvals | 0.572424 |
| GSE31210.SurvRelapseFree_AllMethods_Freq_5 | trial_24 | Adj.MedianPvals | 0.585567 |
| GSE31210.SurvRelapseFree_AllMethods_Freq_6 | trial_24 | Adj.GoodCountPvals | 0.614169 |
| GSE31210.SurvRelapseFree_AllMethods_Freq_2 | trial_25 | Adj.GoodCountPvals | 0.566465 |
| GSE31210.SurvRelapseFree_AllMethods_Freq_3 | trial_25 | Adj.GoodCountPvals | 0.577338 |
| GSE31210.SurvRelapseFree_AllMethods_Freq_4 | trial_25 | Adj.GoodCountPvals | 0.573752 |
| GSE31210.SurvRelapseFree_AllMethods_Freq_5 | trial_25 | Adj.MedianPvals | 0.585567 |
| GSE31210.SurvRelapseFree_AllMethods_Freq_6 | trial_25 | Adj.GoodCountPvals | 0.615781 |
| GSE31210.SurvRelapseFree_AllMethods_Freq_2 | trial_26 | Adj.GoodCountPvals | 0.569613 |
| GSE31210.SurvRelapseFree_AllMethods_Freq_3 | trial_26 | Adj.GoodCountPvals | 0.579994 |
| GSE31210.SurvRelapseFree_AllMethods_Freq_4 | trial_26 | Adj.GoodCountPvals | 0.575322 |
| GSE31210.SurvRelapseFree_AllMethods_Freq_5 | trial_26 | Adj.MedianPvals | 0.585567 |
| GSE31210.SurvRelapseFree_AllMethods_Freq_6 | trial_26 | Adj.GoodCountPvals | 0.615646 |
| GSE31210.SurvRelapseFree_AllMethods_Freq_2 | trial_27 | Adj.GoodCountPvals | 0.564528 |
| GSE31210.SurvRelapseFree_AllMethods_Freq_3 | trial_27 | Adj.GoodCountPvals | 0.574038 |
| GSE31210.SurvRelapseFree_AllMethods_Freq_4 | trial_27 | Adj.GoodCountPvals | 0.570733 |
| GSE31210.SurvRelapseFree_AllMethods_Freq_5 | trial_27 | Adj.MedianPvals | 0.585567 |
| GSE31210.SurvRelapseFree_AllMethods_Freq_6 | trial_27 | Adj.GoodCountPvals | 0.612782 |
| GSE31210.SurvRelapseFree_AllMethods_Freq_2 | trial_28 | Adj.GoodCountPvals | 0.563882 |
| GSE31210.SurvRelapseFree_AllMethods_Freq_3 | trial_28 | Adj.GoodCountPvals | 0.574119 |
| GSE31210.SurvRelapseFree_AllMethods_Freq_4 | trial_28 | Adj.GoodCountPvals | 0.571176 |
| GSE31210.SurvRelapseFree_AllMethods_Freq_5 | trial_28 | Adj.MedianPvals | 0.585567 |
| GSE31210.SurvRelapseFree_AllMethods_Freq_6 | trial_28 | Adj.GoodCountPvals | 0.612692 |
| GSE31210.SurvRelapseFree_AllMethods_Freq_2 | trial_29 | Adj.GoodCountPvals | 0.568765 |
| GSE31210.SurvRelapseFree_AllMethods_Freq_3 | trial_29 | Adj.GoodCountPvals | 0.578625 |
| GSE31210.SurvRelapseFree_AllMethods_Freq_4 | trial_29 | Adj.GoodCountPvals | 0.575966 |
| GSE31210.SurvRelapseFree_AllMethods_Freq_5 | trial_29 | Adj.GoodCountPvals | 0.585855 |
| GSE31210.SurvRelapseFree_AllMethods_Freq_6 | trial_29 | Adj.GoodCountPvals | 0.615378 |
| GSE31210.SurvRelapseFree_AllMethods_Freq_2 | trial_30 | Adj.GoodCountPvals | 0.568321 |
| GSE31210.SurvRelapseFree_AllMethods_Freq_3 | trial_30 | Adj.GoodCountPvals | 0.577418 |
| GSE31210.SurvRelapseFree_AllMethods_Freq_4 | trial_30 | Adj.GoodCountPvals | 0.573873 |
| GSE31210.SurvRelapseFree_AllMethods_Freq_5 | trial_30 | Adj.MedianPvals | 0.585567 |
| GSE31210.SurvRelapseFree_AllMethods_Freq_6 | trial_30 | Adj.GoodCountPvals | 0.614975 |
| GSE31210.SurvRelapseFree_AllMethods_Freq_2 | trial_31 | Adj.GoodCountPvals | 0.566384 |
| GSE31210.SurvRelapseFree_AllMethods_Freq_3 | trial_31 | Adj.GoodCountPvals | 0.577056 |
| GSE31210.SurvRelapseFree_AllMethods_Freq_4 | trial_31 | Adj.GoodCountPvals | 0.57339 |
| GSE31210.SurvRelapseFree_AllMethods_Freq_5 | trial_31 | Adj.MedianPvals | 0.585567 |
| GSE31210.SurvRelapseFree_AllMethods_Freq_6 | trial_31 | Adj.GoodCountPvals | 0.613319 |
| GSE31210.SurvRelapseFree_AllMethods_Freq_2 | trial_32 | Adj.GoodCountPvals | 0.56594 |
| GSE31210.SurvRelapseFree_AllMethods_Freq_3 | trial_32 | Adj.GoodCountPvals | 0.576453 |
| GSE31210.SurvRelapseFree_AllMethods_Freq_4 | trial_32 | Adj.GoodCountPvals | 0.57347 |
| GSE31210.SurvRelapseFree_AllMethods_Freq_5 | trial_32 | Adj.MedianPvals | 0.585567 |
| GSE31210.SurvRelapseFree_AllMethods_Freq_6 | trial_32 | Adj.GoodCountPvals | 0.613095 |
| GSE31210.SurvRelapseFree_AllMethods_Freq_2 | trial_33 | Adj.GoodCountPvals | 0.567474 |
| GSE31210.SurvRelapseFree_AllMethods_Freq_3 | trial_33 | Adj.GoodCountPvals | 0.578183 |
| GSE31210.SurvRelapseFree_AllMethods_Freq_4 | trial_33 | Adj.GoodCountPvals | 0.575443 |
| GSE31210.SurvRelapseFree_AllMethods_Freq_5 | trial_33 | Adj.GoodCountPvals | 0.587747 |
| GSE31210.SurvRelapseFree_AllMethods_Freq_6 | trial_33 | Adj.GoodCountPvals | 0.617526 |
| GSE31210.SurvRelapseFree_AllMethods_Freq_2 | trial_34 | Adj.GoodCountPvals | 0.568846 |
| GSE31210.SurvRelapseFree_AllMethods_Freq_3 | trial_34 | Adj.GoodCountPvals | 0.578827 |
| GSE31210.SurvRelapseFree_AllMethods_Freq_4 | trial_34 | Adj.GoodCountPvals | 0.574517 |
| GSE31210.SurvRelapseFree_AllMethods_Freq_5 | trial_34 | Adj.MedianPvals | 0.585567 |
| GSE31210.SurvRelapseFree_AllMethods_Freq_6 | trial_34 | Adj.GoodCountPvals | 0.613722 |
| GSE31210.SurvRelapseFree_AllMethods_Freq_2 | trial_35 | Adj.GoodCountPvals | 0.569814 |
| GSE31210.SurvRelapseFree_AllMethods_Freq_3 | trial_35 | Adj.GoodCountPvals | 0.579953 |
| GSE31210.SurvRelapseFree_AllMethods_Freq_4 | trial_35 | Adj.GoodCountPvals | 0.575886 |
| GSE31210.SurvRelapseFree_AllMethods_Freq_5 | trial_35 | Adj.MedianPvals | 0.585567 |
| GSE31210.SurvRelapseFree_AllMethods_Freq_6 | trial_35 | Adj.GoodCountPvals | 0.616452 |
| GSE31210.SurvRelapseFree_AllMethods_Freq_2 | trial_36 | Adj.GoodCountPvals | 0.566182 |
| GSE31210.SurvRelapseFree_AllMethods_Freq_3 | trial_36 | Adj.GoodCountPvals | 0.576131 |
| GSE31210.SurvRelapseFree_AllMethods_Freq_4 | trial_36 | Adj.GoodCountPvals | 0.57347 |
| GSE31210.SurvRelapseFree_AllMethods_Freq_5 | trial_36 | Adj.GoodCountPvals | 0.58602 |
| GSE31210.SurvRelapseFree_AllMethods_Freq_6 | trial_36 | Adj.GoodCountPvals | 0.619048 |
| GSE31210.SurvRelapseFree_AllMethods_Freq_2 | trial_37 | Adj.GoodCountPvals | 0.566707 |
| GSE31210.SurvRelapseFree_AllMethods_Freq_3 | trial_37 | Adj.GoodCountPvals | 0.577177 |
| GSE31210.SurvRelapseFree_AllMethods_Freq_4 | trial_37 | Adj.GoodCountPvals | 0.572826 |
| GSE31210.SurvRelapseFree_AllMethods_Freq_5 | trial_37 | Adj.MedianPvals | 0.585567 |
| GSE31210.SurvRelapseFree_AllMethods_Freq_6 | trial_37 | Adj.GoodCountPvals | 0.612737 |
| GSE31210.SurvRelapseFree_AllMethods_Freq_2 | trial_38 | Adj.GoodCountPvals | 0.566344 |
| GSE31210.SurvRelapseFree_AllMethods_Freq_3 | trial_38 | Adj.GoodCountPvals | 0.576694 |
| GSE31210.SurvRelapseFree_AllMethods_Freq_4 | trial_38 | Adj.GoodCountPvals | 0.573551 |
| GSE31210.SurvRelapseFree_AllMethods_Freq_5 | trial_38 | Adj.MedianPvals | 0.585567 |
| GSE31210.SurvRelapseFree_AllMethods_Freq_6 | trial_38 | Adj.GoodCountPvals | 0.613543 |
| GSE31210.SurvRelapseFree_AllMethods_Freq_2 | trial_39 | Adj.GoodCountPvals | 0.563842 |
| GSE31210.SurvRelapseFree_AllMethods_Freq_3 | trial_39 | Adj.GoodCountPvals | 0.5744 |
| GSE31210.SurvRelapseFree_AllMethods_Freq_4 | trial_39 | Adj.GoodCountPvals | 0.570974 |
| GSE31210.SurvRelapseFree_AllMethods_Freq_5 | trial_39 | Adj.MedianPvals | 0.585567 |
| GSE31210.SurvRelapseFree_AllMethods_Freq_6 | trial_39 | Adj.GoodCountPvals | 0.6122 |
| GSE31210.SurvRelapseFree_AllMethods_Freq_2 | trial_40 | Adj.GoodCountPvals | 0.565214 |
| GSE31210.SurvRelapseFree_AllMethods_Freq_3 | trial_40 | Adj.GoodCountPvals | 0.575688 |
| GSE31210.SurvRelapseFree_AllMethods_Freq_4 | trial_40 | Adj.GoodCountPvals | 0.571457 |
| GSE31210.SurvRelapseFree_AllMethods_Freq_5 | trial_40 | Adj.MedianPvals | 0.585567 |
| GSE31210.SurvRelapseFree_AllMethods_Freq_6 | trial_40 | Adj.GoodCountPvals | 0.613767 |
| GSE31210.SurvRelapseFree_AllMethods_Freq_2 | trial_41 | Adj.GoodCountPvals | 0.568563 |
| GSE31210.SurvRelapseFree_AllMethods_Freq_3 | trial_41 | Adj.GoodCountPvals | 0.578746 |
| GSE31210.SurvRelapseFree_AllMethods_Freq_4 | trial_41 | Adj.GoodCountPvals | 0.575523 |
| GSE31210.SurvRelapseFree_AllMethods_Freq_5 | trial_41 | Adj.GoodCountPvals | 0.587212 |
| GSE31210.SurvRelapseFree_AllMethods_Freq_6 | trial_41 | Adj.GoodCountPvals | 0.617929 |
| GSE31210.SurvRelapseFree_AllMethods_Freq_2 | trial_42 | Adj.GoodCountPvals | 0.567353 |
| GSE31210.SurvRelapseFree_AllMethods_Freq_3 | trial_42 | Adj.GoodCountPvals | 0.577338 |
| GSE31210.SurvRelapseFree_AllMethods_Freq_4 | trial_42 | Adj.GoodCountPvals | 0.572826 |
| GSE31210.SurvRelapseFree_AllMethods_Freq_5 | trial_42 | Adj.MedianPvals | 0.585567 |
| GSE31210.SurvRelapseFree_AllMethods_Freq_6 | trial_42 | Adj.GoodCountPvals | 0.612021 |
| GSE31210.SurvRelapseFree_AllMethods_Freq_2 | trial_43 | Adj.GoodCountPvals | 0.565698 |
| GSE31210.SurvRelapseFree_AllMethods_Freq_3 | trial_43 | Adj.GoodCountPvals | 0.576171 |
| GSE31210.SurvRelapseFree_AllMethods_Freq_4 | trial_43 | Adj.GoodCountPvals | 0.573108 |
| GSE31210.SurvRelapseFree_AllMethods_Freq_5 | trial_43 | Adj.MedianPvals | 0.585567 |
| GSE31210.SurvRelapseFree_AllMethods_Freq_6 | trial_43 | Adj.GoodCountPvals | 0.611797 |
| GSE31210.SurvRelapseFree_AllMethods_Freq_2 | trial_44 | Adj.GoodCountPvals | 0.570016 |
| GSE31210.SurvRelapseFree_AllMethods_Freq_3 | trial_44 | Adj.GoodCountPvals | 0.58112 |
| GSE31210.SurvRelapseFree_AllMethods_Freq_4 | trial_44 | Adj.GoodCountPvals | 0.576973 |
| GSE31210.SurvRelapseFree_AllMethods_Freq_5 | trial_44 | Adj.GoodCountPvals | 0.587336 |
| GSE31210.SurvRelapseFree_AllMethods_Freq_6 | trial_44 | Adj.GoodCountPvals | 0.617213 |
| GSE31210.SurvRelapseFree_AllMethods_Freq_2 | trial_45 | Adj.GoodCountPvals | 0.566747 |
| GSE31210.SurvRelapseFree_AllMethods_Freq_3 | trial_45 | Adj.GoodCountPvals | 0.577056 |
| GSE31210.SurvRelapseFree_AllMethods_Freq_4 | trial_45 | Adj.GoodCountPvals | 0.573188 |
| GSE31210.SurvRelapseFree_AllMethods_Freq_5 | trial_45 | Adj.MedianPvals | 0.585567 |
| GSE31210.SurvRelapseFree_AllMethods_Freq_6 | trial_45 | Adj.GoodCountPvals | 0.612469 |
| GSE31210.SurvRelapseFree_AllMethods_Freq_2 | trial_46 | Adj.GoodCountPvals | 0.568563 |
| GSE31210.SurvRelapseFree_AllMethods_Freq_3 | trial_46 | Adj.GoodCountPvals | 0.578827 |
| GSE31210.SurvRelapseFree_AllMethods_Freq_4 | trial_46 | Adj.GoodCountPvals | 0.575081 |
| GSE31210.SurvRelapseFree_AllMethods_Freq_5 | trial_46 | Adj.MedianPvals | 0.585567 |
| GSE31210.SurvRelapseFree_AllMethods_Freq_6 | trial_46 | Adj.GoodCountPvals | 0.613856 |
| GSE31210.SurvRelapseFree_AllMethods_Freq_2 | trial_47 | Adj.GoodCountPvals | 0.564528 |
| GSE31210.SurvRelapseFree_AllMethods_Freq_3 | trial_47 | Adj.GoodCountPvals | 0.575326 |
| GSE31210.SurvRelapseFree_AllMethods_Freq_4 | trial_47 | Adj.GoodCountPvals | 0.572182 |
| GSE31210.SurvRelapseFree_AllMethods_Freq_5 | trial_47 | Adj.MedianPvals | 0.585567 |
| GSE31210.SurvRelapseFree_AllMethods_Freq_6 | trial_47 | Adj.GoodCountPvals | 0.615378 |
| GSE31210.SurvRelapseFree_AllMethods_Freq_2 | trial_48 | Adj.GoodCountPvals | 0.567998 |
| GSE31210.SurvRelapseFree_AllMethods_Freq_3 | trial_48 | Adj.GoodCountPvals | 0.578102 |
| GSE31210.SurvRelapseFree_AllMethods_Freq_4 | trial_48 | Adj.GoodCountPvals | 0.574235 |
| GSE31210.SurvRelapseFree_AllMethods_Freq_5 | trial_48 | Adj.MedianPvals | 0.585567 |
| GSE31210.SurvRelapseFree_AllMethods_Freq_6 | trial_48 | Adj.GoodCountPvals | 0.616273 |
| GSE31210.SurvRelapseFree_AllMethods_Freq_2 | trial_49 | Adj.GoodCountPvals | 0.570581 |
| GSE31210.SurvRelapseFree_AllMethods_Freq_3 | trial_49 | Adj.GoodCountPvals | 0.580678 |
| GSE31210.SurvRelapseFree_AllMethods_Freq_4 | trial_49 | Adj.GoodCountPvals | 0.577496 |
| GSE31210.SurvRelapseFree_AllMethods_Freq_5 | trial_49 | Adj.GoodCountPvals | 0.587788 |
| GSE31210.SurvRelapseFree_AllMethods_Freq_6 | trial_49 | Adj.GoodCountPvals | 0.616049 |
| GSE31210.SurvRelapseFree_AllMethods_Freq_2 | trial_50 | Adj.GoodCountPvals | 0.568241 |
| GSE31210.SurvRelapseFree_AllMethods_Freq_3 | trial_50 | Adj.GoodCountPvals | 0.577539 |
| GSE31210.SurvRelapseFree_AllMethods_Freq_4 | trial_50 | Adj.GoodCountPvals | 0.574356 |
| GSE31210.SurvRelapseFree_AllMethods_Freq_5 | trial_50 | Adj.MedianPvals | 0.585567 |
| GSE31210.SurvRelapseFree_AllMethods_Freq_6 | trial_50 | Adj.GoodCountPvals | 0.613901 |
| GSE31210.SurvRelapseFree_AllMethods_Freq_2 | trial_51 | Adj.GoodCountPvals | 0.565496 |
| GSE31210.SurvRelapseFree_AllMethods_Freq_3 | trial_51 | Adj.GoodCountPvals | 0.57601 |
| GSE31210.SurvRelapseFree_AllMethods_Freq_4 | trial_51 | Adj.GoodCountPvals | 0.572061 |
| GSE31210.SurvRelapseFree_AllMethods_Freq_5 | trial_51 | Adj.MedianPvals | 0.585567 |
| GSE31210.SurvRelapseFree_AllMethods_Freq_6 | trial_51 | Adj.GoodCountPvals | 0.614438 |
| GSE31210.SurvRelapseFree_AllMethods_Freq_2 | trial_52 | Adj.GoodCountPvals | 0.566263 |
| GSE31210.SurvRelapseFree_AllMethods_Freq_3 | trial_52 | Adj.GoodCountPvals | 0.576171 |
| GSE31210.SurvRelapseFree_AllMethods_Freq_4 | trial_52 | Adj.GoodCountPvals | 0.572705 |
| GSE31210.SurvRelapseFree_AllMethods_Freq_5 | trial_52 | Adj.MedianPvals | 0.585567 |
| GSE31210.SurvRelapseFree_AllMethods_Freq_6 | trial_52 | Adj.GoodCountPvals | 0.615378 |
| GSE31210.SurvRelapseFree_AllMethods_Freq_2 | trial_53 | Adj.GoodCountPvals | 0.56816 |
| GSE31210.SurvRelapseFree_AllMethods_Freq_3 | trial_53 | Adj.GoodCountPvals | 0.578706 |
| GSE31210.SurvRelapseFree_AllMethods_Freq_4 | trial_53 | Adj.GoodCountPvals | 0.574638 |
| GSE31210.SurvRelapseFree_AllMethods_Freq_5 | trial_53 | Adj.MedianPvals | 0.585567 |
| GSE31210.SurvRelapseFree_AllMethods_Freq_6 | trial_53 | Adj.GoodCountPvals | 0.616631 |
| GSE31210.SurvRelapseFree_AllMethods_Freq_2 | trial_54 | Adj.GoodCountPvals | 0.568967 |
| GSE31210.SurvRelapseFree_AllMethods_Freq_3 | trial_54 | Adj.GoodCountPvals | 0.57931 |
| GSE31210.SurvRelapseFree_AllMethods_Freq_4 | trial_54 | Adj.GoodCountPvals | 0.575845 |
| GSE31210.SurvRelapseFree_AllMethods_Freq_5 | trial_54 | Adj.GoodCountPvals | 0.585732 |
| GSE31210.SurvRelapseFree_AllMethods_Freq_6 | trial_54 | Adj.GoodCountPvals | 0.616676 |
| GSE31210.SurvRelapseFree_AllMethods_Freq_2 | trial_55 | Adj.GoodCountPvals | 0.566747 |
| GSE31210.SurvRelapseFree_AllMethods_Freq_3 | trial_55 | Adj.GoodCountPvals | 0.577177 |
| GSE31210.SurvRelapseFree_AllMethods_Freq_4 | trial_55 | Adj.GoodCountPvals | 0.572907 |
| GSE31210.SurvRelapseFree_AllMethods_Freq_5 | trial_55 | Adj.MedianPvals | 0.585567 |
| GSE31210.SurvRelapseFree_AllMethods_Freq_6 | trial_55 | Adj.GoodCountPvals | 0.614617 |
| GSE31210.SurvRelapseFree_AllMethods_Freq_2 | trial_56 | Adj.GoodCountPvals | 0.567918 |
| GSE31210.SurvRelapseFree_AllMethods_Freq_3 | trial_56 | Adj.GoodCountPvals | 0.578706 |
| GSE31210.SurvRelapseFree_AllMethods_Freq_4 | trial_56 | Adj.GoodCountPvals | 0.575242 |
| GSE31210.SurvRelapseFree_AllMethods_Freq_5 | trial_56 | Adj.MedianPvals | 0.585567 |
| GSE31210.SurvRelapseFree_AllMethods_Freq_6 | trial_56 | Adj.GoodCountPvals | 0.613677 |
| GSE31210.SurvRelapseFree_AllMethods_Freq_2 | trial_57 | Adj.GoodCountPvals | 0.568684 |
| GSE31210.SurvRelapseFree_AllMethods_Freq_3 | trial_57 | Adj.GoodCountPvals | 0.578344 |
| GSE31210.SurvRelapseFree_AllMethods_Freq_4 | trial_57 | Adj.GoodCountPvals | 0.573873 |
| GSE31210.SurvRelapseFree_AllMethods_Freq_5 | trial_57 | Adj.MedianPvals | 0.585567 |
| GSE31210.SurvRelapseFree_AllMethods_Freq_6 | trial_57 | Adj.GoodCountPvals | 0.615243 |
| GSE31210.SurvRelapseFree_AllMethods_Freq_2 | trial_58 | Adj.GoodCountPvals | 0.566182 |
| GSE31210.SurvRelapseFree_AllMethods_Freq_3 | trial_58 | Adj.GoodCountPvals | 0.576412 |
| GSE31210.SurvRelapseFree_AllMethods_Freq_4 | trial_58 | Adj.GoodCountPvals | 0.573309 |
| GSE31210.SurvRelapseFree_AllMethods_Freq_5 | trial_58 | Adj.MedianPvals | 0.585567 |
| GSE31210.SurvRelapseFree_AllMethods_Freq_6 | trial_58 | Adj.GoodCountPvals | 0.614035 |
| GSE31210.SurvRelapseFree_AllMethods_Freq_2 | trial_59 | Adj.GoodCountPvals | 0.571429 |
| GSE31210.SurvRelapseFree_AllMethods_Freq_3 | trial_59 | Adj.GoodCountPvals | 0.581442 |
| GSE31210.SurvRelapseFree_AllMethods_Freq_4 | trial_59 | Adj.GoodCountPvals | 0.57806 |
| GSE31210.SurvRelapseFree_AllMethods_Freq_5 | trial_59 | Adj.GoodCountPvals | 0.586883 |
| GSE31210.SurvRelapseFree_AllMethods_Freq_6 | trial_59 | Adj.GoodCountPvals | 0.617213 |
| GSE31210.SurvRelapseFree_AllMethods_Freq_2 | trial_60 | Adj.GoodCountPvals | 0.567918 |
| GSE31210.SurvRelapseFree_AllMethods_Freq_3 | trial_60 | Adj.GoodCountPvals | 0.577821 |
| GSE31210.SurvRelapseFree_AllMethods_Freq_4 | trial_60 | Adj.GoodCountPvals | 0.57496 |
| GSE31210.SurvRelapseFree_AllMethods_Freq_5 | trial_60 | Adj.MedianPvals | 0.585567 |
| GSE31210.SurvRelapseFree_AllMethods_Freq_6 | trial_60 | Adj.GoodCountPvals | 0.616094 |
| GSE31210.SurvRelapseFree_AllMethods_Freq_2 | trial_61 | Adj.GoodCountPvals | 0.568402 |
| GSE31210.SurvRelapseFree_AllMethods_Freq_3 | trial_61 | Adj.GoodCountPvals | 0.578947 |
| GSE31210.SurvRelapseFree_AllMethods_Freq_4 | trial_61 | Adj.GoodCountPvals | 0.575725 |
| GSE31210.SurvRelapseFree_AllMethods_Freq_5 | trial_61 | Adj.GoodCountPvals | 0.587541 |
| GSE31210.SurvRelapseFree_AllMethods_Freq_6 | trial_61 | Adj.GoodCountPvals | 0.620435 |
| GSE31210.SurvRelapseFree_AllMethods_Freq_2 | trial_62 | Adj.GoodCountPvals | 0.565052 |
| GSE31210.SurvRelapseFree_AllMethods_Freq_3 | trial_62 | Adj.GoodCountPvals | 0.5744 |
| GSE31210.SurvRelapseFree_AllMethods_Freq_4 | trial_62 | Adj.GoodCountPvals | 0.570531 |
| GSE31210.SurvRelapseFree_AllMethods_Freq_5 | trial_62 | Adj.MedianPvals | 0.585567 |
| GSE31210.SurvRelapseFree_AllMethods_Freq_6 | trial_62 | Adj.GoodCountPvals | 0.6122 |
| GSE31210.SurvRelapseFree_AllMethods_Freq_2 | trial_63 | Adj.GoodCountPvals | 0.567514 |
| GSE31210.SurvRelapseFree_AllMethods_Freq_3 | trial_63 | Adj.GoodCountPvals | 0.577901 |
| GSE31210.SurvRelapseFree_AllMethods_Freq_4 | trial_63 | Adj.GoodCountPvals | 0.574195 |
| GSE31210.SurvRelapseFree_AllMethods_Freq_5 | trial_63 | Adj.MedianPvals | 0.585567 |
| GSE31210.SurvRelapseFree_AllMethods_Freq_6 | trial_63 | Adj.GoodCountPvals | 0.61587 |
| GSE31210.SurvRelapseFree_AllMethods_Freq_2 | trial_64 | Adj.GoodCountPvals | 0.569855 |
| GSE31210.SurvRelapseFree_AllMethods_Freq_3 | trial_64 | Adj.GoodCountPvals | 0.579631 |
| GSE31210.SurvRelapseFree_AllMethods_Freq_4 | trial_64 | Adj.GoodCountPvals | 0.577254 |
| GSE31210.SurvRelapseFree_AllMethods_Freq_5 | trial_64 | Adj.GoodCountPvals | 0.587623 |
| GSE31210.SurvRelapseFree_AllMethods_Freq_6 | trial_64 | Adj.GoodCountPvals | 0.618242 |
| GSE31210.SurvRelapseFree_AllMethods_Freq_2 | trial_65 | Adj.GoodCountPvals | 0.566949 |
| GSE31210.SurvRelapseFree_AllMethods_Freq_3 | trial_65 | Adj.GoodCountPvals | 0.577539 |
| GSE31210.SurvRelapseFree_AllMethods_Freq_4 | trial_65 | Adj.GoodCountPvals | 0.574919 |
| GSE31210.SurvRelapseFree_AllMethods_Freq_5 | trial_65 | Adj.MedianPvals | 0.585567 |
| GSE31210.SurvRelapseFree_AllMethods_Freq_6 | trial_65 | Adj.GoodCountPvals | 0.617078 |
| GSE31210.SurvRelapseFree_AllMethods_Freq_2 | trial_66 | Adj.GoodCountPvals | 0.564487 |
| GSE31210.SurvRelapseFree_AllMethods_Freq_3 | trial_66 | Adj.GoodCountPvals | 0.574843 |
| GSE31210.SurvRelapseFree_AllMethods_Freq_4 | trial_66 | Adj.GoodCountPvals | 0.571779 |
| GSE31210.SurvRelapseFree_AllMethods_Freq_5 | trial_66 | Adj.MedianPvals | 0.585567 |
| GSE31210.SurvRelapseFree_AllMethods_Freq_6 | trial_66 | Adj.GoodCountPvals | 0.614169 |
| GSE31210.SurvRelapseFree_AllMethods_Freq_2 | trial_67 | Adj.GoodCountPvals | 0.565295 |
| GSE31210.SurvRelapseFree_AllMethods_Freq_3 | trial_67 | Adj.GoodCountPvals | 0.575366 |
| GSE31210.SurvRelapseFree_AllMethods_Freq_4 | trial_67 | Adj.GoodCountPvals | 0.572142 |
| GSE31210.SurvRelapseFree_AllMethods_Freq_5 | trial_67 | Adj.MedianPvals | 0.585567 |
| GSE31210.SurvRelapseFree_AllMethods_Freq_6 | trial_67 | Adj.GoodCountPvals | 0.61408 |
| GSE31210.SurvRelapseFree_AllMethods_Freq_2 | trial_68 | Adj.GoodCountPvals | 0.568604 |
| GSE31210.SurvRelapseFree_AllMethods_Freq_3 | trial_68 | Adj.GoodCountPvals | 0.578907 |
| GSE31210.SurvRelapseFree_AllMethods_Freq_4 | trial_68 | Adj.GoodCountPvals | 0.575201 |
| GSE31210.SurvRelapseFree_AllMethods_Freq_5 | trial_68 | Adj.GoodCountPvals | 0.586308 |
| GSE31210.SurvRelapseFree_AllMethods_Freq_6 | trial_68 | Adj.GoodCountPvals | 0.617526 |
| GSE31210.SurvRelapseFree_AllMethods_Freq_2 | trial_69 | Adj.GoodCountPvals | 0.567353 |
| GSE31210.SurvRelapseFree_AllMethods_Freq_3 | trial_69 | Adj.GoodCountPvals | 0.57778 |
| GSE31210.SurvRelapseFree_AllMethods_Freq_4 | trial_69 | Adj.GoodCountPvals | 0.573913 |
| GSE31210.SurvRelapseFree_AllMethods_Freq_5 | trial_69 | Adj.MedianPvals | 0.585567 |
| GSE31210.SurvRelapseFree_AllMethods_Freq_6 | trial_69 | Adj.GoodCountPvals | 0.616183 |
| GSE31210.SurvRelapseFree_AllMethods_Freq_2 | trial_70 | Adj.GoodCountPvals | 0.568402 |
| GSE31210.SurvRelapseFree_AllMethods_Freq_3 | trial_70 | Adj.GoodCountPvals | 0.578545 |
| GSE31210.SurvRelapseFree_AllMethods_Freq_4 | trial_70 | Adj.GoodCountPvals | 0.574839 |
| GSE31210.SurvRelapseFree_AllMethods_Freq_5 | trial_70 | Adj.MedianPvals | 0.585567 |
| GSE31210.SurvRelapseFree_AllMethods_Freq_6 | trial_70 | Adj.GoodCountPvals | 0.615288 |
| GSE31210.SurvRelapseFree_AllMethods_Freq_2 | trial_71 | Adj.GoodCountPvals | 0.56933 |
| GSE31210.SurvRelapseFree_AllMethods_Freq_3 | trial_71 | Adj.GoodCountPvals | 0.579873 |
| GSE31210.SurvRelapseFree_AllMethods_Freq_4 | trial_71 | Adj.GoodCountPvals | 0.576167 |
| GSE31210.SurvRelapseFree_AllMethods_Freq_5 | trial_71 | Adj.GoodCountPvals | 0.586266 |
| GSE31210.SurvRelapseFree_AllMethods_Freq_6 | trial_71 | Adj.GoodCountPvals | 0.616004 |
| GSE31210.SurvRelapseFree_AllMethods_Freq_2 | trial_72 | Adj.GoodCountPvals | 0.566102 |
| GSE31210.SurvRelapseFree_AllMethods_Freq_3 | trial_72 | Adj.GoodCountPvals | 0.576935 |
| GSE31210.SurvRelapseFree_AllMethods_Freq_4 | trial_72 | Adj.GoodCountPvals | 0.57343 |
| GSE31210.SurvRelapseFree_AllMethods_Freq_5 | trial_72 | Adj.MedianPvals | 0.585567 |
| GSE31210.SurvRelapseFree_AllMethods_Freq_6 | trial_72 | Adj.GoodCountPvals | 0.611484 |
| GSE31210.SurvRelapseFree_AllMethods_Freq_2 | trial_73 | Adj.GoodCountPvals | 0.565214 |
| GSE31210.SurvRelapseFree_AllMethods_Freq_3 | trial_73 | Adj.GoodCountPvals | 0.575527 |
| GSE31210.SurvRelapseFree_AllMethods_Freq_4 | trial_73 | Adj.GoodCountPvals | 0.572021 |
| GSE31210.SurvRelapseFree_AllMethods_Freq_5 | trial_73 | Adj.MedianPvals | 0.585567 |
| GSE31210.SurvRelapseFree_AllMethods_Freq_6 | trial_73 | Adj.GoodCountPvals | 0.61323 |
| GSE31210.SurvRelapseFree_AllMethods_Freq_2 | trial_74 | Adj.GoodCountPvals | 0.568119 |
| GSE31210.SurvRelapseFree_AllMethods_Freq_3 | trial_74 | Adj.GoodCountPvals | 0.578022 |
| GSE31210.SurvRelapseFree_AllMethods_Freq_4 | trial_74 | Adj.GoodCountPvals | 0.574074 |
| GSE31210.SurvRelapseFree_AllMethods_Freq_5 | trial_74 | Adj.MedianPvals | 0.585567 |
| GSE31210.SurvRelapseFree_AllMethods_Freq_6 | trial_74 | Adj.GoodCountPvals | 0.613767 |
| GSE31210.SurvRelapseFree_AllMethods_Freq_2 | trial_75 | Adj.GoodCountPvals | 0.571953 |
| GSE31210.SurvRelapseFree_AllMethods_Freq_3 | trial_75 | Adj.GoodCountPvals | 0.582529 |
| GSE31210.SurvRelapseFree_AllMethods_Freq_4 | trial_75 | Adj.GoodCountPvals | 0.577979 |
| GSE31210.SurvRelapseFree_AllMethods_Freq_5 | trial_75 | Adj.GoodCountPvals | 0.588322 |
| GSE31210.SurvRelapseFree_AllMethods_Freq_6 | trial_75 | Adj.GoodCountPvals | 0.61954 |
| GSE31210.SurvRelapseFree_AllMethods_Freq_2 | trial_76 | Adj.GoodCountPvals | 0.567918 |
| GSE31210.SurvRelapseFree_AllMethods_Freq_3 | trial_76 | Adj.GoodCountPvals | 0.578143 |
| GSE31210.SurvRelapseFree_AllMethods_Freq_4 | trial_76 | Adj.GoodCountPvals | 0.574316 |
| GSE31210.SurvRelapseFree_AllMethods_Freq_5 | trial_76 | Adj.MedianPvals | 0.585567 |
| GSE31210.SurvRelapseFree_AllMethods_Freq_6 | trial_76 | Adj.GoodCountPvals | 0.614259 |
| GSE31210.SurvRelapseFree_AllMethods_Freq_2 | trial_77 | Adj.GoodCountPvals | 0.566747 |
| GSE31210.SurvRelapseFree_AllMethods_Freq_3 | trial_77 | Adj.GoodCountPvals | 0.577016 |
| GSE31210.SurvRelapseFree_AllMethods_Freq_4 | trial_77 | Adj.GoodCountPvals | 0.57339 |
| GSE31210.SurvRelapseFree_AllMethods_Freq_5 | trial_77 | Adj.MedianPvals | 0.585567 |
| GSE31210.SurvRelapseFree_AllMethods_Freq_6 | trial_77 | Adj.GoodCountPvals | 0.615243 |
| GSE31210.SurvRelapseFree_AllMethods_Freq_2 | trial_78 | Adj.GoodCountPvals | 0.569048 |
| GSE31210.SurvRelapseFree_AllMethods_Freq_3 | trial_78 | Adj.GoodCountPvals | 0.578907 |
| GSE31210.SurvRelapseFree_AllMethods_Freq_4 | trial_78 | Adj.GoodCountPvals | 0.575564 |
| GSE31210.SurvRelapseFree_AllMethods_Freq_5 | trial_78 | Adj.MedianPvals | 0.585567 |
| GSE31210.SurvRelapseFree_AllMethods_Freq_6 | trial_78 | Adj.GoodCountPvals | 0.614483 |
| GSE31210.SurvRelapseFree_AllMethods_Freq_2 | trial_79 | Adj.GoodCountPvals | 0.568119 |
| GSE31210.SurvRelapseFree_AllMethods_Freq_3 | trial_79 | Adj.GoodCountPvals | 0.578384 |
| GSE31210.SurvRelapseFree_AllMethods_Freq_4 | trial_79 | Adj.GoodCountPvals | 0.573792 |
| GSE31210.SurvRelapseFree_AllMethods_Freq_5 | trial_79 | Adj.MedianPvals | 0.585567 |
| GSE31210.SurvRelapseFree_AllMethods_Freq_6 | trial_79 | Adj.GoodCountPvals | 0.615467 |
| GSE31210.SurvRelapseFree_AllMethods_Freq_2 | trial_80 | Adj.GoodCountPvals | 0.560613 |
| GSE31210.SurvRelapseFree_AllMethods_Freq_3 | trial_80 | Adj.GoodCountPvals | 0.571825 |
| GSE31210.SurvRelapseFree_AllMethods_Freq_4 | trial_80 | Adj.GoodCountPvals | 0.568438 |
| GSE31210.SurvRelapseFree_AllMethods_Freq_5 | trial_80 | Adj.MedianPvals | 0.585567 |
| GSE31210.SurvRelapseFree_AllMethods_Freq_6 | trial_80 | Adj.GoodCountPvals | 0.612424 |
| GSE31210.SurvRelapseFree_AllMethods_Freq_2 | trial_81 | Adj.GoodCountPvals | 0.568927 |
| GSE31210.SurvRelapseFree_AllMethods_Freq_3 | trial_81 | Adj.GoodCountPvals | 0.578505 |
| GSE31210.SurvRelapseFree_AllMethods_Freq_4 | trial_81 | Adj.GoodCountPvals | 0.57504 |
| GSE31210.SurvRelapseFree_AllMethods_Freq_5 | trial_81 | Adj.MedianPvals | 0.585567 |
| GSE31210.SurvRelapseFree_AllMethods_Freq_6 | trial_81 | Adj.GoodCountPvals | 0.615691 |
| GSE31210.SurvRelapseFree_AllMethods_Freq_2 | trial_82 | Adj.GoodCountPvals | 0.569814 |
| GSE31210.SurvRelapseFree_AllMethods_Freq_3 | trial_82 | Adj.GoodCountPvals | 0.57931 |
| GSE31210.SurvRelapseFree_AllMethods_Freq_4 | trial_82 | Adj.GoodCountPvals | 0.575805 |
| GSE31210.SurvRelapseFree_AllMethods_Freq_5 | trial_82 | Adj.GoodCountPvals | 0.586102 |
| GSE31210.SurvRelapseFree_AllMethods_Freq_6 | trial_82 | Adj.GoodCountPvals | 0.616944 |
| GSE31210.SurvRelapseFree_AllMethods_Freq_2 | trial_83 | Adj.GoodCountPvals | 0.570379 |
| GSE31210.SurvRelapseFree_AllMethods_Freq_3 | trial_83 | Adj.GoodCountPvals | 0.580637 |
| GSE31210.SurvRelapseFree_AllMethods_Freq_4 | trial_83 | Adj.GoodCountPvals | 0.576409 |
| GSE31210.SurvRelapseFree_AllMethods_Freq_5 | trial_83 | Adj.GoodCountPvals | 0.585814 |
| GSE31210.SurvRelapseFree_AllMethods_Freq_6 | trial_83 | Adj.GoodCountPvals | 0.616541 |
| GSE31210.SurvRelapseFree_AllMethods_Freq_2 | trial_84 | Adj.GoodCountPvals | 0.566747 |
| GSE31210.SurvRelapseFree_AllMethods_Freq_3 | trial_84 | Adj.GoodCountPvals | 0.576654 |
| GSE31210.SurvRelapseFree_AllMethods_Freq_4 | trial_84 | Adj.GoodCountPvals | 0.571739 |
| GSE31210.SurvRelapseFree_AllMethods_Freq_5 | trial_84 | Adj.MedianPvals | 0.585567 |
| GSE31210.SurvRelapseFree_AllMethods_Freq_6 | trial_84 | Adj.GoodCountPvals | 0.613632 |
| GSE31210.SurvRelapseFree_AllMethods_Freq_2 | trial_85 | Adj.GoodCountPvals | 0.5659 |
| GSE31210.SurvRelapseFree_AllMethods_Freq_3 | trial_85 | Adj.GoodCountPvals | 0.576412 |
| GSE31210.SurvRelapseFree_AllMethods_Freq_4 | trial_85 | Adj.GoodCountPvals | 0.573269 |
| GSE31210.SurvRelapseFree_AllMethods_Freq_5 | trial_85 | Adj.MedianPvals | 0.585567 |
| GSE31210.SurvRelapseFree_AllMethods_Freq_6 | trial_85 | Adj.GoodCountPvals | 0.615915 |
| GSE31210.SurvRelapseFree_AllMethods_Freq_2 | trial_86 | Adj.GoodCountPvals | 0.568765 |
| GSE31210.SurvRelapseFree_AllMethods_Freq_3 | trial_86 | Adj.GoodCountPvals | 0.578625 |
| GSE31210.SurvRelapseFree_AllMethods_Freq_4 | trial_86 | Adj.GoodCountPvals | 0.574758 |
| GSE31210.SurvRelapseFree_AllMethods_Freq_5 | trial_86 | Adj.MedianPvals | 0.585567 |
| GSE31210.SurvRelapseFree_AllMethods_Freq_6 | trial_86 | Adj.GoodCountPvals | 0.613274 |
| GSE31210.SurvRelapseFree_AllMethods_Freq_2 | trial_87 | Adj.GoodCountPvals | 0.567151 |
| GSE31210.SurvRelapseFree_AllMethods_Freq_3 | trial_87 | Adj.GoodCountPvals | 0.57762 |
| GSE31210.SurvRelapseFree_AllMethods_Freq_4 | trial_87 | Adj.GoodCountPvals | 0.575483 |
| GSE31210.SurvRelapseFree_AllMethods_Freq_5 | trial_87 | Adj.MedianPvals | 0.585567 |
| GSE31210.SurvRelapseFree_AllMethods_Freq_6 | trial_87 | Adj.GoodCountPvals | 0.614841 |
| GSE31210.SurvRelapseFree_AllMethods_Freq_2 | trial_88 | Adj.GoodCountPvals | 0.569935 |
| GSE31210.SurvRelapseFree_AllMethods_Freq_3 | trial_88 | Adj.GoodCountPvals | 0.580114 |
| GSE31210.SurvRelapseFree_AllMethods_Freq_4 | trial_88 | Adj.GoodCountPvals | 0.575604 |
| GSE31210.SurvRelapseFree_AllMethods_Freq_5 | trial_88 | Adj.GoodCountPvals | 0.586554 |
| GSE31210.SurvRelapseFree_AllMethods_Freq_6 | trial_88 | Adj.GoodCountPvals | 0.618511 |
| GSE31210.SurvRelapseFree_AllMethods_Freq_2 | trial_89 | Adj.GoodCountPvals | 0.568563 |
| GSE31210.SurvRelapseFree_AllMethods_Freq_3 | trial_89 | Adj.GoodCountPvals | 0.578746 |
| GSE31210.SurvRelapseFree_AllMethods_Freq_4 | trial_89 | Adj.GoodCountPvals | 0.575242 |
| GSE31210.SurvRelapseFree_AllMethods_Freq_5 | trial_89 | Adj.GoodCountPvals | 0.585691 |
| GSE31210.SurvRelapseFree_AllMethods_Freq_6 | trial_89 | Adj.GoodCountPvals | 0.615825 |
| GSE31210.SurvRelapseFree_AllMethods_Freq_2 | trial_90 | Adj.GoodCountPvals | 0.567958 |
| GSE31210.SurvRelapseFree_AllMethods_Freq_3 | trial_90 | Adj.GoodCountPvals | 0.578062 |
| GSE31210.SurvRelapseFree_AllMethods_Freq_4 | trial_90 | Adj.GoodCountPvals | 0.575201 |
| GSE31210.SurvRelapseFree_AllMethods_Freq_5 | trial_90 | Adj.MedianPvals | 0.585567 |
| GSE31210.SurvRelapseFree_AllMethods_Freq_6 | trial_90 | Adj.GoodCountPvals | 0.616676 |
| GSE31210.SurvRelapseFree_AllMethods_Freq_2 | trial_91 | Adj.GoodCountPvals | 0.567797 |
| GSE31210.SurvRelapseFree_AllMethods_Freq_3 | trial_91 | Adj.GoodCountPvals | 0.577982 |
| GSE31210.SurvRelapseFree_AllMethods_Freq_4 | trial_91 | Adj.GoodCountPvals | 0.575161 |
| GSE31210.SurvRelapseFree_AllMethods_Freq_5 | trial_91 | Adj.MedianPvals | 0.585567 |
| GSE31210.SurvRelapseFree_AllMethods_Freq_6 | trial_91 | Adj.GoodCountPvals | 0.616228 |
| GSE31210.SurvRelapseFree_AllMethods_Freq_2 | trial_92 | Adj.GoodCountPvals | 0.567837 |
| GSE31210.SurvRelapseFree_AllMethods_Freq_3 | trial_92 | Adj.GoodCountPvals | 0.57778 |
| GSE31210.SurvRelapseFree_AllMethods_Freq_4 | trial_92 | Adj.GoodCountPvals | 0.573309 |
| GSE31210.SurvRelapseFree_AllMethods_Freq_5 | trial_92 | Adj.GoodCountPvals:Adj.MedianPvals | 0.585567 |
| GSE31210.SurvRelapseFree_AllMethods_Freq_6 | trial_92 | Adj.GoodCountPvals | 0.61672 |
| GSE31210.SurvRelapseFree_AllMethods_Freq_2 | trial_93 | Adj.GoodCountPvals | 0.57042 |
| GSE31210.SurvRelapseFree_AllMethods_Freq_3 | trial_93 | Adj.GoodCountPvals | 0.579953 |
| GSE31210.SurvRelapseFree_AllMethods_Freq_4 | trial_93 | Adj.GoodCountPvals | 0.575644 |
| GSE31210.SurvRelapseFree_AllMethods_Freq_5 | trial_93 | Adj.GoodCountPvals | 0.585938 |
| GSE31210.SurvRelapseFree_AllMethods_Freq_6 | trial_93 | Adj.GoodCountPvals | 0.616183 |
| GSE31210.SurvRelapseFree_AllMethods_Freq_2 | trial_94 | Adj.GoodCountPvals | 0.568321 |
| GSE31210.SurvRelapseFree_AllMethods_Freq_3 | trial_94 | Adj.GoodCountPvals | 0.578505 |
| GSE31210.SurvRelapseFree_AllMethods_Freq_4 | trial_94 | Adj.GoodCountPvals | 0.574436 |
| GSE31210.SurvRelapseFree_AllMethods_Freq_5 | trial_94 | Adj.MedianPvals | 0.585567 |
| GSE31210.SurvRelapseFree_AllMethods_Freq_6 | trial_94 | Adj.GoodCountPvals | 0.613588 |
| GSE31210.SurvRelapseFree_AllMethods_Freq_2 | trial_95 | Adj.GoodCountPvals | 0.568846 |
| GSE31210.SurvRelapseFree_AllMethods_Freq_3 | trial_95 | Adj.GoodCountPvals | 0.579068 |
| GSE31210.SurvRelapseFree_AllMethods_Freq_4 | trial_95 | Adj.GoodCountPvals | 0.575886 |
| GSE31210.SurvRelapseFree_AllMethods_Freq_5 | trial_95 | Adj.GoodCountPvals | 0.586719 |
| GSE31210.SurvRelapseFree_AllMethods_Freq_6 | trial_95 | Adj.GoodCountPvals | 0.617839 |
| GSE31210.SurvRelapseFree_AllMethods_Freq_2 | trial_96 | Adj.GoodCountPvals | 0.567918 |
| GSE31210.SurvRelapseFree_AllMethods_Freq_3 | trial_96 | Adj.GoodCountPvals | 0.578102 |
| GSE31210.SurvRelapseFree_AllMethods_Freq_4 | trial_96 | Adj.GoodCountPvals | 0.574396 |
| GSE31210.SurvRelapseFree_AllMethods_Freq_5 | trial_96 | Adj.MedianPvals | 0.585567 |
| GSE31210.SurvRelapseFree_AllMethods_Freq_6 | trial_96 | Adj.GoodCountPvals | 0.614975 |
| GSE31210.SurvRelapseFree_AllMethods_Freq_2 | trial_97 | Adj.GoodCountPvals | 0.566707 |
| GSE31210.SurvRelapseFree_AllMethods_Freq_3 | trial_97 | Adj.GoodCountPvals | 0.577016 |
| GSE31210.SurvRelapseFree_AllMethods_Freq_4 | trial_97 | Adj.GoodCountPvals | 0.572907 |
| GSE31210.SurvRelapseFree_AllMethods_Freq_5 | trial_97 | Adj.MedianPvals | 0.585567 |
| GSE31210.SurvRelapseFree_AllMethods_Freq_6 | trial_97 | Adj.GoodCountPvals | 0.614214 |
| GSE31210.SurvRelapseFree_AllMethods_Freq_2 | trial_98 | Adj.GoodCountPvals | 0.567756 |
| GSE31210.SurvRelapseFree_AllMethods_Freq_3 | trial_98 | Adj.GoodCountPvals | 0.577378 |
| GSE31210.SurvRelapseFree_AllMethods_Freq_4 | trial_98 | Adj.GoodCountPvals | 0.573551 |
| GSE31210.SurvRelapseFree_AllMethods_Freq_5 | trial_98 | Adj.MedianPvals | 0.585567 |
| GSE31210.SurvRelapseFree_AllMethods_Freq_6 | trial_98 | Adj.GoodCountPvals | 0.615154 |
| GSE31210.SurvRelapseFree_AllMethods_Freq_2 | trial_99 | Adj.GoodCountPvals | 0.565295 |
| GSE31210.SurvRelapseFree_AllMethods_Freq_3 | trial_99 | Adj.GoodCountPvals | 0.575527 |
| GSE31210.SurvRelapseFree_AllMethods_Freq_4 | trial_99 | Adj.GoodCountPvals | 0.572987 |
| GSE31210.SurvRelapseFree_AllMethods_Freq_5 | trial_99 | Adj.MedianPvals | 0.585567 |
| GSE31210.SurvRelapseFree_AllMethods_Freq_6 | trial_99 | Adj.GoodCountPvals | 0.613364 |
| GSE31210.SurvRelapseFree_AllMethods_Freq_2 | trial_100 | Adj.GoodCountPvals | 0.566909 |
| GSE31210.SurvRelapseFree_AllMethods_Freq_3 | trial_100 | Adj.GoodCountPvals | 0.577499 |
| GSE31210.SurvRelapseFree_AllMethods_Freq_4 | trial_100 | Adj.GoodCountPvals | 0.574275 |
| GSE31210.SurvRelapseFree_AllMethods_Freq_5 | trial_100 | Adj.MedianPvals | 0.585567 |
| GSE31210.SurvRelapseFree_AllMethods_Freq_6 | trial_100 | Adj.GoodCountPvals | 0.613006 |
| HARVARD-LC.Surv_AllMethods_Freq_2 | trial_1 | Adj.GoodCountPvals | 0.789593 |
| HARVARD-LC.Surv_AllMethods_Freq_2 | trial_2 | Adj.GoodCountPvals | 0.799774 |
| HARVARD-LC.Surv_AllMethods_Freq_2 | trial_3 | Adj.MedianPvals | 0.781674 |
| HARVARD-LC.Surv_AllMethods_Freq_2 | trial_4 | Adj.GoodCountPvals | 0.797511 |
| HARVARD-LC.Surv_AllMethods_Freq_2 | trial_5 | Adj.MedianPvals | 0.781674 |
| HARVARD-LC.Surv_AllMethods_Freq_2 | trial_6 | Adj.MedianPvals | 0.781674 |
| HARVARD-LC.Surv_AllMethods_Freq_2 | trial_7 | Adj.MedianPvals | 0.781674 |
| HARVARD-LC.Surv_AllMethods_Freq_2 | trial_8 | Adj.MedianPvals | 0.781674 |
| HARVARD-LC.Surv_AllMethods_Freq_2 | trial_9 | Adj.GoodCountPvals | 0.788462 |
| HARVARD-LC.Surv_AllMethods_Freq_2 | trial_10 | Adj.GoodCountPvals | 0.789593 |
| HARVARD-LC.Surv_AllMethods_Freq_2 | trial_11 | Adj.GoodCountPvals | 0.789593 |
| HARVARD-LC.Surv_AllMethods_Freq_2 | trial_12 | Adj.GoodCountPvals:Adj.MedianPvals | 0.781674 |
| HARVARD-LC.Surv_AllMethods_Freq_2 | trial_13 | Adj.GoodCountPvals | 0.785068 |
| HARVARD-LC.Surv_AllMethods_Freq_2 | trial_14 | Adj.MedianPvals | 0.781674 |
| HARVARD-LC.Surv_AllMethods_Freq_2 | trial_15 | Adj.MedianPvals | 0.781674 |
| HARVARD-LC.Surv_AllMethods_Freq_2 | trial_16 | Adj.MedianPvals | 0.781674 |
| HARVARD-LC.Surv_AllMethods_Freq_2 | trial_17 | Adj.MedianPvals | 0.781674 |
| HARVARD-LC.Surv_AllMethods_Freq_2 | trial_18 | Adj.GoodCountPvals | 0.786199 |
| HARVARD-LC.Surv_AllMethods_Freq_2 | trial_19 | Adj.GoodCountPvals | 0.808824 |
| HARVARD-LC.Surv_AllMethods_Freq_2 | trial_20 | Adj.MedianPvals | 0.781674 |
| HARVARD-LC.Surv_AllMethods_Freq_2 | trial_21 | Adj.GoodCountPvals | 0.789593 |
| HARVARD-LC.Surv_AllMethods_Freq_2 | trial_22 | Adj.GoodCountPvals | 0.78733 |
| HARVARD-LC.Surv_AllMethods_Freq_2 | trial_23 | Adj.MedianPvals | 0.781674 |
| HARVARD-LC.Surv_AllMethods_Freq_2 | trial_24 | Adj.GoodCountPvals | 0.789593 |
| HARVARD-LC.Surv_AllMethods_Freq_2 | trial_25 | Adj.GoodCountPvals | 0.788462 |
| HARVARD-LC.Surv_AllMethods_Freq_2 | trial_26 | Adj.GoodCountPvals | 0.786199 |
| HARVARD-LC.Surv_AllMethods_Freq_2 | trial_27 | Adj.GoodCountPvals | 0.792986 |
| HARVARD-LC.Surv_AllMethods_Freq_2 | trial_28 | Adj.MedianPvals | 0.781674 |
| HARVARD-LC.Surv_AllMethods_Freq_2 | trial_29 | Adj.GoodCountPvals | 0.788462 |
| HARVARD-LC.Surv_AllMethods_Freq_2 | trial_30 | Adj.MedianPvals | 0.781674 |
| HARVARD-LC.Surv_AllMethods_Freq_2 | trial_31 | Adj.GoodCountPvals | 0.785068 |
| HARVARD-LC.Surv_AllMethods_Freq_2 | trial_32 | Adj.GoodCountPvals | 0.795249 |
| HARVARD-LC.Surv_AllMethods_Freq_2 | trial_33 | Adj.GoodCountPvals | 0.806561 |
| HARVARD-LC.Surv_AllMethods_Freq_2 | trial_34 | Adj.GoodCountPvals | 0.789593 |
| HARVARD-LC.Surv_AllMethods_Freq_2 | trial_35 | Adj.MedianPvals | 0.781674 |
| HARVARD-LC.Surv_AllMethods_Freq_2 | trial_36 | Adj.GoodCountPvals | 0.799774 |
| HARVARD-LC.Surv_AllMethods_Freq_2 | trial_37 | Adj.GoodCountPvals | 0.791855 |
| HARVARD-LC.Surv_AllMethods_Freq_2 | trial_38 | Adj.MedianPvals | 0.781674 |
| HARVARD-LC.Surv_AllMethods_Freq_2 | trial_39 | Adj.GoodCountPvals | 0.78733 |
| HARVARD-LC.Surv_AllMethods_Freq_2 | trial_40 | Adj.MedianPvals | 0.781674 |
| HARVARD-LC.Surv_AllMethods_Freq_2 | trial_41 | Adj.GoodCountPvals | 0.785068 |
| HARVARD-LC.Surv_AllMethods_Freq_2 | trial_42 | Adj.MedianPvals | 0.781674 |
| HARVARD-LC.Surv_AllMethods_Freq_2 | trial_43 | Adj.GoodCountPvals | 0.799774 |
| HARVARD-LC.Surv_AllMethods_Freq_2 | trial_44 | Adj.MedianPvals | 0.781674 |
| HARVARD-LC.Surv_AllMethods_Freq_2 | trial_45 | Adj.MedianPvals | 0.781674 |
| HARVARD-LC.Surv_AllMethods_Freq_2 | trial_46 | Adj.GoodCountPvals | 0.786199 |
| HARVARD-LC.Surv_AllMethods_Freq_2 | trial_47 | Adj.MedianPvals | 0.781674 |
| HARVARD-LC.Surv_AllMethods_Freq_2 | trial_48 | Adj.GoodCountPvals | 0.792986 |
| HARVARD-LC.Surv_AllMethods_Freq_2 | trial_49 | Adj.GoodCountPvals:Adj.MedianPvals | 0.781674 |
| HARVARD-LC.Surv_AllMethods_Freq_2 | trial_50 | Adj.MedianPvals | 0.781674 |
| HARVARD-LC.Surv_AllMethods_Freq_2 | trial_51 | Adj.GoodCountPvals | 0.788462 |
| HARVARD-LC.Surv_AllMethods_Freq_2 | trial_52 | Adj.GoodCountPvals | 0.790724 |
| HARVARD-LC.Surv_AllMethods_Freq_2 | trial_53 | Adj.GoodCountPvals | 0.78733 |
| HARVARD-LC.Surv_AllMethods_Freq_2 | trial_54 | Adj.GoodCountPvals | 0.785068 |
| HARVARD-LC.Surv_AllMethods_Freq_2 | trial_55 | Adj.GoodCountPvals | 0.795249 |
| HARVARD-LC.Surv_AllMethods_Freq_2 | trial_56 | Adj.MedianPvals | 0.781674 |
| HARVARD-LC.Surv_AllMethods_Freq_2 | trial_57 | Adj.GoodCountPvals | 0.782805 |
| HARVARD-LC.Surv_AllMethods_Freq_2 | trial_58 | Adj.GoodCountPvals | 0.798643 |
| HARVARD-LC.Surv_AllMethods_Freq_2 | trial_59 | Adj.MedianPvals | 0.781674 |
| HARVARD-LC.Surv_AllMethods_Freq_2 | trial_60 | Adj.MedianPvals | 0.781674 |
| HARVARD-LC.Surv_AllMethods_Freq_2 | trial_61 | Adj.GoodCountPvals | 0.79638 |
| HARVARD-LC.Surv_AllMethods_Freq_2 | trial_62 | Adj.MedianPvals | 0.781674 |
| HARVARD-LC.Surv_AllMethods_Freq_2 | trial_63 | Adj.MedianPvals | 0.781674 |
| HARVARD-LC.Surv_AllMethods_Freq_2 | trial_64 | Adj.GoodCountPvals | 0.788462 |
| HARVARD-LC.Surv_AllMethods_Freq_2 | trial_65 | Adj.GoodCountPvals | 0.795249 |
| HARVARD-LC.Surv_AllMethods_Freq_2 | trial_66 | Adj.MedianPvals | 0.781674 |
| HARVARD-LC.Surv_AllMethods_Freq_2 | trial_67 | Adj.GoodCountPvals | 0.785068 |
| HARVARD-LC.Surv_AllMethods_Freq_2 | trial_68 | Adj.GoodCountPvals | 0.802036 |
| HARVARD-LC.Surv_AllMethods_Freq_2 | trial_69 | Adj.GoodCountPvals | 0.789593 |
| HARVARD-LC.Surv_AllMethods_Freq_2 | trial_70 | Adj.MedianPvals | 0.781674 |
| HARVARD-LC.Surv_AllMethods_Freq_2 | trial_71 | Adj.GoodCountPvals | 0.78733 |
| HARVARD-LC.Surv_AllMethods_Freq_2 | trial_72 | Adj.GoodCountPvals | 0.788462 |
| HARVARD-LC.Surv_AllMethods_Freq_2 | trial_73 | Adj.GoodCountPvals | 0.795249 |
| HARVARD-LC.Surv_AllMethods_Freq_2 | trial_74 | Adj.MedianPvals | 0.781674 |
| HARVARD-LC.Surv_AllMethods_Freq_2 | trial_75 | Adj.GoodCountPvals | 0.786199 |
| HARVARD-LC.Surv_AllMethods_Freq_2 | trial_76 | Adj.GoodCountPvals | 0.788462 |
| HARVARD-LC.Surv_AllMethods_Freq_2 | trial_77 | Adj.MedianPvals | 0.781674 |
| HARVARD-LC.Surv_AllMethods_Freq_2 | trial_78 | Adj.MedianPvals | 0.781674 |
| HARVARD-LC.Surv_AllMethods_Freq_2 | trial_79 | Adj.MedianPvals | 0.781674 |
| HARVARD-LC.Surv_AllMethods_Freq_2 | trial_80 | Adj.MedianPvals | 0.781674 |
| HARVARD-LC.Surv_AllMethods_Freq_2 | trial_81 | Adj.GoodCountPvals:Adj.MedianPvals | 0.781674 |
| HARVARD-LC.Surv_AllMethods_Freq_2 | trial_82 | Adj.GoodCountPvals | 0.806561 |
| HARVARD-LC.Surv_AllMethods_Freq_2 | trial_83 | Adj.MedianPvals | 0.781674 |
| HARVARD-LC.Surv_AllMethods_Freq_2 | trial_84 | Adj.MedianPvals | 0.781674 |
| HARVARD-LC.Surv_AllMethods_Freq_2 | trial_85 | Adj.GoodCountPvals | 0.786199 |
| HARVARD-LC.Surv_AllMethods_Freq_2 | trial_86 | Adj.MedianPvals | 0.781674 |
| HARVARD-LC.Surv_AllMethods_Freq_2 | trial_87 | Adj.GoodCountPvals | 0.78733 |
| HARVARD-LC.Surv_AllMethods_Freq_2 | trial_88 | Adj.MedianPvals | 0.781674 |
| HARVARD-LC.Surv_AllMethods_Freq_2 | trial_89 | Adj.GoodCountPvals | 0.794118 |
| HARVARD-LC.Surv_AllMethods_Freq_2 | trial_90 | Adj.MedianPvals | 0.781674 |
| HARVARD-LC.Surv_AllMethods_Freq_2 | trial_91 | Adj.GoodCountPvals | 0.789593 |
| HARVARD-LC.Surv_AllMethods_Freq_2 | trial_92 | Adj.MedianPvals | 0.781674 |
| HARVARD-LC.Surv_AllMethods_Freq_2 | trial_93 | Adj.MedianPvals | 0.781674 |
| HARVARD-LC.Surv_AllMethods_Freq_2 | trial_94 | Adj.MedianPvals | 0.781674 |
| HARVARD-LC.Surv_AllMethods_Freq_2 | trial_95 | Adj.GoodCountPvals | 0.78733 |
| HARVARD-LC.Surv_AllMethods_Freq_2 | trial_96 | Adj.MedianPvals | 0.781674 |
| HARVARD-LC.Surv_AllMethods_Freq_2 | trial_97 | Adj.MedianPvals | 0.781674 |
| HARVARD-LC.Surv_AllMethods_Freq_2 | trial_98 | Adj.GoodCountPvals | 0.792986 |
| HARVARD-LC.Surv_AllMethods_Freq_2 | trial_99 | Adj.GoodCountPvals | 0.791855 |
| HARVARD-LC.Surv_AllMethods_Freq_2 | trial_100 | Adj.MedianPvals | 0.781674 |

**Table B**. Summary of the methods for each trial of tests with the top AUC comparing with other methods, from which **Fig 4B** is created. (total 100 trials of TCGA results with 9 PrognoScan datasets as described in Materials and methods section; data trials with all NAs for their AUCs (**Table C**) were not considered)

Column “PrognoScan_TruthGeneLists”: Truth Gene Lists from each indicated PrognoScan LUAD datasets (GSE13213, GSE31210 etc) with shared gene lists from the methods in comparison with the indicated frequency (Freq_2 for shared by 2 of method lists; Freq_2 for shared by 3 of method lists, and so on so forth). Column “TCGA_Trials”: trials derived from TCGA LUAD data (different trials of permutations in our method). Column “TopAUCMethod”: the method with the top or largest AUC for each of the combined truth gene list of PrognoScan datasets at different shared frequency (Freq_2, Freq_3 etc) and each of the TCGA_Trials in each row. If two methods were tied, both methods were listed. Column “TopAUCMethodAUCs”: the actual AUC for the method with the top or largest AUC.

| **Table C. AUCs of all methods for each trial of tests, from which S2 Table is created** | | | | | | | |
| --- | --- | --- | --- | --- | --- | --- | --- |
| PrognoScan_TruthGeneLists | TCGA  _Trials | Adj.GoodCountPvals | Adj.CORRECTED_P_VALUE | Adj.CoxPvalbyRanks | Adj.COX_P_VALUE | Adj.MedianPvals | Adj.tertPvals |
| GSE13213.Surv_AllMethods_Freq_2 | trial_1 | 0.658805 | 0.621616 | 0.674953 | 0.617462 | 0.658068 | 0.669727 |
| GSE13213.Surv_AllMethods_Freq_3 | trial_1 | 0.670697 | 0.637978 | 0.687022 | 0.624932 | 0.665096 | 0.677801 |
| GSE13213.Surv_AllMethods_Freq_4 | trial_1 | 0.653282 | 0.620725 | 0.663212 | 0.597064 | 0.631261 | 0.681693 |
| GSE13213.Surv_AllMethods_Freq_5 | trial_1 | 0.550257 | 0.541262 | 0.58538 | 0.542833 | 0.600228 | 0.624786 |
| GSE13213.Surv_AllMethods_Freq_6 | trial_1 | 0.601506 | 0.583333 | 0.515057 | 0.600208 | 0.551402 | 0.562305 |
| GSE13213.Surv_AllMethods_Freq_2 | trial_2 | 0.658738 | 0.621616 | 0.674953 | 0.617462 | 0.658068 | 0.669727 |
| GSE13213.Surv_AllMethods_Freq_3 | trial_2 | 0.671107 | 0.637978 | 0.687022 | 0.624932 | 0.665096 | 0.677801 |
| GSE13213.Surv_AllMethods_Freq_4 | trial_2 | 0.650173 | 0.620725 | 0.663212 | 0.597064 | 0.631261 | 0.681693 |
| GSE13213.Surv_AllMethods_Freq_5 | trial_2 | 0.547973 | 0.541262 | 0.58538 | 0.542833 | 0.600228 | 0.624786 |
| GSE13213.Surv_AllMethods_Freq_6 | trial_2 | 0.599429 | 0.583333 | 0.515057 | 0.600208 | 0.551402 | 0.562305 |
| GSE13213.Surv_AllMethods_Freq_2 | trial_3 | 0.657196 | 0.621616 | 0.674953 | 0.617462 | 0.658068 | 0.669727 |
| GSE13213.Surv_AllMethods_Freq_3 | trial_3 | 0.668989 | 0.637978 | 0.687022 | 0.624932 | 0.665096 | 0.677801 |
| GSE13213.Surv_AllMethods_Freq_4 | trial_3 | 0.649223 | 0.620725 | 0.663212 | 0.597064 | 0.631261 | 0.681693 |
| GSE13213.Surv_AllMethods_Freq_5 | trial_3 | 0.546545 | 0.541262 | 0.58538 | 0.542833 | 0.600228 | 0.624786 |
| GSE13213.Surv_AllMethods_Freq_6 | trial_3 | 0.599429 | 0.583333 | 0.515057 | 0.600208 | 0.551402 | 0.562305 |
| GSE13213.Surv_AllMethods_Freq_2 | trial_4 | 0.659341 | 0.621616 | 0.674953 | 0.617462 | 0.658068 | 0.669727 |
| GSE13213.Surv_AllMethods_Freq_3 | trial_4 | 0.671243 | 0.637978 | 0.687022 | 0.624932 | 0.665096 | 0.677801 |
| GSE13213.Surv_AllMethods_Freq_4 | trial_4 | 0.653972 | 0.620725 | 0.663212 | 0.597064 | 0.631261 | 0.681693 |
| GSE13213.Surv_AllMethods_Freq_5 | trial_4 | 0.553684 | 0.541262 | 0.58538 | 0.542833 | 0.600228 | 0.624786 |
| GSE13213.Surv_AllMethods_Freq_6 | trial_4 | 0.59891 | 0.583333 | 0.515057 | 0.600208 | 0.551402 | 0.562305 |
| GSE13213.Surv_AllMethods_Freq_2 | trial_5 | 0.655923 | 0.621616 | 0.674953 | 0.617462 | 0.658068 | 0.669727 |
| GSE13213.Surv_AllMethods_Freq_3 | trial_5 | 0.668511 | 0.637978 | 0.687022 | 0.624932 | 0.665096 | 0.677801 |
| GSE13213.Surv_AllMethods_Freq_4 | trial_5 | 0.649741 | 0.620725 | 0.663212 | 0.597064 | 0.631261 | 0.681693 |
| GSE13213.Surv_AllMethods_Freq_5 | trial_5 | 0.548115 | 0.541262 | 0.58538 | 0.542833 | 0.600228 | 0.624786 |
| GSE13213.Surv_AllMethods_Freq_6 | trial_5 | 0.596314 | 0.583333 | 0.515057 | 0.600208 | 0.551402 | 0.562305 |
| GSE13213.Surv_AllMethods_Freq_2 | trial_6 | 0.656325 | 0.621616 | 0.674953 | 0.617462 | 0.658068 | 0.669727 |
| GSE13213.Surv_AllMethods_Freq_3 | trial_6 | 0.668511 | 0.637978 | 0.687022 | 0.624932 | 0.665096 | 0.677801 |
| GSE13213.Surv_AllMethods_Freq_4 | trial_6 | 0.651209 | 0.620725 | 0.663212 | 0.597064 | 0.631261 | 0.681693 |
| GSE13213.Surv_AllMethods_Freq_5 | trial_6 | 0.5504 | 0.541262 | 0.58538 | 0.542833 | 0.600228 | 0.624786 |
| GSE13213.Surv_AllMethods_Freq_6 | trial_6 | 0.596573 | 0.583333 | 0.515057 | 0.600208 | 0.551402 | 0.562305 |
| GSE13213.Surv_AllMethods_Freq_2 | trial_7 | 0.656526 | 0.621616 | 0.674953 | 0.617462 | 0.658068 | 0.669727 |
| GSE13213.Surv_AllMethods_Freq_3 | trial_7 | 0.668784 | 0.637978 | 0.687022 | 0.624932 | 0.665096 | 0.677801 |
| GSE13213.Surv_AllMethods_Freq_4 | trial_7 | 0.649309 | 0.620725 | 0.663212 | 0.597064 | 0.631261 | 0.681693 |
| GSE13213.Surv_AllMethods_Freq_5 | trial_7 | 0.544974 | 0.541262 | 0.58538 | 0.542833 | 0.600228 | 0.624786 |
| GSE13213.Surv_AllMethods_Freq_6 | trial_7 | 0.603063 | 0.583333 | 0.515057 | 0.600208 | 0.551402 | 0.562305 |
| GSE13213.Surv_AllMethods_Freq_2 | trial_8 | 0.657666 | 0.621616 | 0.674953 | 0.617462 | 0.658068 | 0.669727 |
| GSE13213.Surv_AllMethods_Freq_3 | trial_8 | 0.670014 | 0.637978 | 0.687022 | 0.624932 | 0.665096 | 0.677801 |
| GSE13213.Surv_AllMethods_Freq_4 | trial_8 | 0.652763 | 0.620725 | 0.663212 | 0.597064 | 0.631261 | 0.681693 |
| GSE13213.Surv_AllMethods_Freq_5 | trial_8 | 0.449457 | 0.541262 | 0.58538 | 0.542833 | 0.600228 | 0.624786 |
| GSE13213.Surv_AllMethods_Freq_6 | trial_8 | 0.601246 | 0.583333 | 0.515057 | 0.600208 | 0.551402 | 0.562305 |
| GSE13213.Surv_AllMethods_Freq_2 | trial_9 | 0.65914 | 0.621616 | 0.674953 | 0.617462 | 0.658068 | 0.669727 |
| GSE13213.Surv_AllMethods_Freq_3 | trial_9 | 0.671858 | 0.637978 | 0.687022 | 0.624932 | 0.665096 | 0.677801 |
| GSE13213.Surv_AllMethods_Freq_4 | trial_9 | 0.652159 | 0.620725 | 0.663212 | 0.597064 | 0.631261 | 0.681693 |
| GSE13213.Surv_AllMethods_Freq_5 | trial_9 | 0.55297 | 0.541262 | 0.58538 | 0.542833 | 0.600228 | 0.624786 |
| GSE13213.Surv_AllMethods_Freq_6 | trial_9 | 0.597092 | 0.583333 | 0.515057 | 0.600208 | 0.551402 | 0.562305 |
| GSE13213.Surv_AllMethods_Freq_2 | trial_10 | 0.658068 | 0.621616 | 0.674953 | 0.617462 | 0.658068 | 0.669727 |
| GSE13213.Surv_AllMethods_Freq_3 | trial_10 | 0.670697 | 0.637978 | 0.687022 | 0.624932 | 0.665096 | 0.677801 |
| GSE13213.Surv_AllMethods_Freq_4 | trial_10 | 0.653368 | 0.620725 | 0.663212 | 0.597064 | 0.631261 | 0.681693 |
| GSE13213.Surv_AllMethods_Freq_5 | trial_10 | 0.553398 | 0.541262 | 0.58538 | 0.542833 | 0.600228 | 0.624786 |
| GSE13213.Surv_AllMethods_Freq_6 | trial_10 | 0.5919 | 0.583333 | 0.515057 | 0.600208 | 0.551402 | 0.562305 |
| GSE13213.Surv_AllMethods_Freq_2 | trial_11 | 0.656459 | 0.621616 | 0.674953 | 0.617462 | 0.658068 | 0.669727 |
| GSE13213.Surv_AllMethods_Freq_3 | trial_11 | 0.668784 | 0.637978 | 0.687022 | 0.624932 | 0.665096 | 0.677801 |
| GSE13213.Surv_AllMethods_Freq_4 | trial_11 | 0.648791 | 0.620725 | 0.663212 | 0.597064 | 0.631261 | 0.681693 |
| GSE13213.Surv_AllMethods_Freq_5 | trial_11 | 0.544974 | 0.541262 | 0.58538 | 0.542833 | 0.600228 | 0.624786 |
| GSE13213.Surv_AllMethods_Freq_6 | trial_11 | 0.601246 | 0.583333 | 0.515057 | 0.600208 | 0.551402 | 0.562305 |
| GSE13213.Surv_AllMethods_Freq_2 | trial_12 | 0.660145 | 0.621616 | 0.674953 | 0.617462 | 0.658068 | 0.669727 |
| GSE13213.Surv_AllMethods_Freq_3 | trial_12 | 0.672473 | 0.637978 | 0.687022 | 0.624932 | 0.665096 | 0.677801 |
| GSE13213.Surv_AllMethods_Freq_4 | trial_12 | 0.654059 | 0.620725 | 0.663212 | 0.597064 | 0.631261 | 0.681693 |
| GSE13213.Surv_AllMethods_Freq_5 | trial_12 | 0.552684 | 0.541262 | 0.58538 | 0.542833 | 0.600228 | 0.624786 |
| GSE13213.Surv_AllMethods_Freq_6 | trial_12 | 0.593977 | 0.583333 | 0.515057 | 0.600208 | 0.551402 | 0.562305 |
| GSE13213.Surv_AllMethods_Freq_2 | trial_13 | 0.655588 | 0.621616 | 0.674953 | 0.617462 | 0.658068 | 0.669727 |
| GSE13213.Surv_AllMethods_Freq_3 | trial_13 | 0.667828 | 0.637978 | 0.687022 | 0.624932 | 0.665096 | 0.677801 |
| GSE13213.Surv_AllMethods_Freq_4 | trial_13 | 0.647841 | 0.620725 | 0.663212 | 0.597064 | 0.631261 | 0.681693 |
| GSE13213.Surv_AllMethods_Freq_5 | trial_13 | 0.548115 | 0.541262 | 0.58538 | 0.542833 | 0.600228 | 0.624786 |
| GSE13213.Surv_AllMethods_Freq_6 | trial_13 | 0.599948 | 0.583333 | 0.515057 | 0.600208 | 0.551402 | 0.562305 |
| GSE13213.Surv_AllMethods_Freq_2 | trial_14 | 0.65532 | 0.621616 | 0.674953 | 0.617462 | 0.658068 | 0.669727 |
| GSE13213.Surv_AllMethods_Freq_3 | trial_14 | 0.66776 | 0.637978 | 0.687022 | 0.624932 | 0.665096 | 0.677801 |
| GSE13213.Surv_AllMethods_Freq_4 | trial_14 | 0.647927 | 0.620725 | 0.663212 | 0.597064 | 0.631261 | 0.681693 |
| GSE13213.Surv_AllMethods_Freq_5 | trial_14 | 0.548115 | 0.541262 | 0.58538 | 0.542833 | 0.600228 | 0.624786 |
| GSE13213.Surv_AllMethods_Freq_6 | trial_14 | 0.599169 | 0.583333 | 0.515057 | 0.600208 | 0.551402 | 0.562305 |
| GSE13213.Surv_AllMethods_Freq_2 | trial_15 | 0.657733 | 0.621616 | 0.674953 | 0.617462 | 0.658068 | 0.669727 |
| GSE13213.Surv_AllMethods_Freq_3 | trial_15 | 0.669945 | 0.637978 | 0.687022 | 0.624932 | 0.665096 | 0.677801 |
| GSE13213.Surv_AllMethods_Freq_4 | trial_15 | 0.650432 | 0.620725 | 0.663212 | 0.597064 | 0.631261 | 0.681693 |
| GSE13213.Surv_AllMethods_Freq_5 | trial_15 | 0.549686 | 0.541262 | 0.58538 | 0.542833 | 0.600228 | 0.624786 |
| GSE13213.Surv_AllMethods_Freq_6 | trial_15 | 0.596314 | 0.583333 | 0.515057 | 0.600208 | 0.551402 | 0.562305 |
| GSE13213.Surv_AllMethods_Freq_2 | trial_16 | 0.654851 | 0.621616 | 0.674953 | 0.617462 | 0.658068 | 0.669727 |
| GSE13213.Surv_AllMethods_Freq_3 | trial_16 | 0.667077 | 0.637978 | 0.687022 | 0.624932 | 0.665096 | 0.677801 |
| GSE13213.Surv_AllMethods_Freq_4 | trial_16 | 0.647841 | 0.620725 | 0.663212 | 0.597064 | 0.631261 | 0.681693 |
| GSE13213.Surv_AllMethods_Freq_5 | trial_16 | 0.546117 | 0.541262 | 0.58538 | 0.542833 | 0.600228 | 0.624786 |
| GSE13213.Surv_AllMethods_Freq_6 | trial_16 | 0.602285 | 0.583333 | 0.515057 | 0.600208 | 0.551402 | 0.562305 |
| GSE13213.Surv_AllMethods_Freq_2 | trial_17 | 0.655119 | 0.621616 | 0.674953 | 0.617462 | 0.658068 | 0.669727 |
| GSE13213.Surv_AllMethods_Freq_3 | trial_17 | 0.667213 | 0.637978 | 0.687022 | 0.624932 | 0.665096 | 0.677801 |
| GSE13213.Surv_AllMethods_Freq_4 | trial_17 | 0.647582 | 0.620725 | 0.663212 | 0.597064 | 0.631261 | 0.681693 |
| GSE13213.Surv_AllMethods_Freq_5 | trial_17 | 0.550828 | 0.541262 | 0.58538 | 0.542833 | 0.600228 | 0.624786 |
| GSE13213.Surv_AllMethods_Freq_6 | trial_17 | 0.594756 | 0.583333 | 0.515057 | 0.600208 | 0.551402 | 0.562305 |
| GSE13213.Surv_AllMethods_Freq_2 | trial_18 | 0.655856 | 0.621616 | 0.674953 | 0.617462 | 0.658068 | 0.669727 |
| GSE13213.Surv_AllMethods_Freq_3 | trial_18 | 0.66776 | 0.637978 | 0.687022 | 0.624932 | 0.665096 | 0.677801 |
| GSE13213.Surv_AllMethods_Freq_4 | trial_18 | 0.649914 | 0.620725 | 0.663212 | 0.597064 | 0.631261 | 0.681693 |
| GSE13213.Surv_AllMethods_Freq_5 | trial_18 | 0.552541 | 0.541262 | 0.58538 | 0.542833 | 0.600228 | 0.624786 |
| GSE13213.Surv_AllMethods_Freq_6 | trial_18 | 0.591121 | 0.583333 | 0.515057 | 0.600208 | 0.551402 | 0.562305 |
| GSE13213.Surv_AllMethods_Freq_2 | trial_19 | 0.654047 | 0.621616 | 0.674953 | 0.617462 | 0.658068 | 0.669727 |
| GSE13213.Surv_AllMethods_Freq_3 | trial_19 | 0.666257 | 0.637978 | 0.687022 | 0.624932 | 0.665096 | 0.677801 |
| GSE13213.Surv_AllMethods_Freq_4 | trial_19 | 0.647064 | 0.620725 | 0.663212 | 0.597064 | 0.631261 | 0.681693 |
| GSE13213.Surv_AllMethods_Freq_5 | trial_19 | 0.544689 | 0.541262 | 0.58538 | 0.542833 | 0.600228 | 0.624786 |
| GSE13213.Surv_AllMethods_Freq_6 | trial_19 | 0.602025 | 0.583333 | 0.515057 | 0.600208 | 0.551402 | 0.562305 |
| GSE13213.Surv_AllMethods_Freq_2 | trial_20 | 0.65733 | 0.621616 | 0.674953 | 0.617462 | 0.658068 | 0.669727 |
| GSE13213.Surv_AllMethods_Freq_3 | trial_20 | 0.669809 | 0.637978 | 0.687022 | 0.624932 | 0.665096 | 0.677801 |
| GSE13213.Surv_AllMethods_Freq_4 | trial_20 | 0.65285 | 0.620725 | 0.663212 | 0.597064 | 0.631261 | 0.681693 |
| GSE13213.Surv_AllMethods_Freq_5 | trial_20 | 0.554826 | 0.541262 | 0.58538 | 0.542833 | 0.600228 | 0.624786 |
| GSE13213.Surv_AllMethods_Freq_6 | trial_20 | 0.597612 | 0.583333 | 0.515057 | 0.600208 | 0.551402 | 0.562305 |
| GSE13213.Surv_AllMethods_Freq_2 | trial_21 | 0.659408 | 0.621616 | 0.674953 | 0.617462 | 0.658068 | 0.669727 |
| GSE13213.Surv_AllMethods_Freq_3 | trial_21 | 0.671721 | 0.637978 | 0.687022 | 0.624932 | 0.665096 | 0.677801 |
| GSE13213.Surv_AllMethods_Freq_4 | trial_21 | 0.65285 | 0.620725 | 0.663212 | 0.597064 | 0.631261 | 0.681693 |
| GSE13213.Surv_AllMethods_Freq_5 | trial_21 | 0.553255 | 0.541262 | 0.58538 | 0.542833 | 0.600228 | 0.624786 |
| GSE13213.Surv_AllMethods_Freq_6 | trial_21 | 0.59865 | 0.583333 | 0.515057 | 0.600208 | 0.551402 | 0.562305 |
| GSE13213.Surv_AllMethods_Freq_2 | trial_22 | 0.657196 | 0.621616 | 0.674953 | 0.617462 | 0.658068 | 0.669727 |
| GSE13213.Surv_AllMethods_Freq_3 | trial_22 | 0.669126 | 0.637978 | 0.687022 | 0.624932 | 0.665096 | 0.677801 |
| GSE13213.Surv_AllMethods_Freq_4 | trial_22 | 0.649741 | 0.620725 | 0.663212 | 0.597064 | 0.631261 | 0.681693 |
| GSE13213.Surv_AllMethods_Freq_5 | trial_22 | 0.550971 | 0.541262 | 0.58538 | 0.542833 | 0.600228 | 0.624786 |
| GSE13213.Surv_AllMethods_Freq_6 | trial_22 | 0.599429 | 0.583333 | 0.515057 | 0.600208 | 0.551402 | 0.562305 |
| GSE13213.Surv_AllMethods_Freq_2 | trial_23 | 0.654315 | 0.621616 | 0.674953 | 0.617462 | 0.658068 | 0.669727 |
| GSE13213.Surv_AllMethods_Freq_3 | trial_23 | 0.66694 | 0.637978 | 0.687022 | 0.624932 | 0.665096 | 0.677801 |
| GSE13213.Surv_AllMethods_Freq_4 | trial_23 | 0.646891 | 0.620725 | 0.663212 | 0.597064 | 0.631261 | 0.681693 |
| GSE13213.Surv_AllMethods_Freq_5 | trial_23 | 0.5504 | 0.541262 | 0.58538 | 0.542833 | 0.600228 | 0.624786 |
| GSE13213.Surv_AllMethods_Freq_6 | trial_23 | 0.600987 | 0.583333 | 0.515057 | 0.600208 | 0.551402 | 0.562305 |
| GSE13213.Surv_AllMethods_Freq_2 | trial_24 | 0.658269 | 0.621616 | 0.674953 | 0.617462 | 0.658068 | 0.669727 |
| GSE13213.Surv_AllMethods_Freq_3 | trial_24 | 0.671038 | 0.637978 | 0.687022 | 0.624932 | 0.665096 | 0.677801 |
| GSE13213.Surv_AllMethods_Freq_4 | trial_24 | 0.649136 | 0.620725 | 0.663212 | 0.597064 | 0.631261 | 0.681693 |
| GSE13213.Surv_AllMethods_Freq_5 | trial_24 | 0.547401 | 0.541262 | 0.58538 | 0.542833 | 0.600228 | 0.624786 |
| GSE13213.Surv_AllMethods_Freq_6 | trial_24 | 0.602025 | 0.583333 | 0.515057 | 0.600208 | 0.551402 | 0.562305 |
| GSE13213.Surv_AllMethods_Freq_2 | trial_25 | 0.658001 | 0.621616 | 0.674953 | 0.617462 | 0.658068 | 0.669727 |
| GSE13213.Surv_AllMethods_Freq_3 | trial_25 | 0.670355 | 0.637978 | 0.687022 | 0.624932 | 0.665096 | 0.677801 |
| GSE13213.Surv_AllMethods_Freq_4 | trial_25 | 0.649223 | 0.620725 | 0.663212 | 0.597064 | 0.631261 | 0.681693 |
| GSE13213.Surv_AllMethods_Freq_5 | trial_25 | 0.547544 | 0.541262 | 0.58538 | 0.542833 | 0.600228 | 0.624786 |
| GSE13213.Surv_AllMethods_Freq_6 | trial_25 | 0.59839 | 0.583333 | 0.515057 | 0.600208 | 0.551402 | 0.562305 |
| GSE13213.Surv_AllMethods_Freq_2 | trial_26 | 0.660078 | 0.621616 | 0.674953 | 0.617462 | 0.658068 | 0.669727 |
| GSE13213.Surv_AllMethods_Freq_3 | trial_26 | 0.672814 | 0.637978 | 0.687022 | 0.624932 | 0.665096 | 0.677801 |
| GSE13213.Surv_AllMethods_Freq_4 | trial_26 | 0.653195 | 0.620725 | 0.663212 | 0.597064 | 0.631261 | 0.681693 |
| GSE13213.Surv_AllMethods_Freq_5 | trial_26 | 0.552399 | 0.541262 | 0.58538 | 0.542833 | 0.600228 | 0.624786 |
| GSE13213.Surv_AllMethods_Freq_6 | trial_26 | 0.597352 | 0.583333 | 0.515057 | 0.600208 | 0.551402 | 0.562305 |
| GSE13213.Surv_AllMethods_Freq_2 | trial_27 | 0.65264 | 0.621616 | 0.674953 | 0.617462 | 0.658068 | 0.669727 |
| GSE13213.Surv_AllMethods_Freq_3 | trial_27 | 0.664754 | 0.637978 | 0.687022 | 0.624932 | 0.665096 | 0.677801 |
| GSE13213.Surv_AllMethods_Freq_4 | trial_27 | 0.643782 | 0.620725 | 0.663212 | 0.597064 | 0.631261 | 0.681693 |
| GSE13213.Surv_AllMethods_Freq_5 | trial_27 | 0.544118 | 0.541262 | 0.58538 | 0.542833 | 0.600228 | 0.624786 |
| GSE13213.Surv_AllMethods_Freq_6 | trial_27 | 0.603063 | 0.583333 | 0.515057 | 0.600208 | 0.551402 | 0.562305 |
| GSE13213.Surv_AllMethods_Freq_2 | trial_28 | 0.656325 | 0.621616 | 0.674953 | 0.617462 | 0.658068 | 0.669727 |
| GSE13213.Surv_AllMethods_Freq_3 | trial_28 | 0.668579 | 0.637978 | 0.687022 | 0.624932 | 0.665096 | 0.677801 |
| GSE13213.Surv_AllMethods_Freq_4 | trial_28 | 0.649827 | 0.620725 | 0.663212 | 0.597064 | 0.631261 | 0.681693 |
| GSE13213.Surv_AllMethods_Freq_5 | trial_28 | 0.549686 | 0.541262 | 0.58538 | 0.542833 | 0.600228 | 0.624786 |
| GSE13213.Surv_AllMethods_Freq_6 | trial_28 | 0.595535 | 0.583333 | 0.515057 | 0.600208 | 0.551402 | 0.562305 |
| GSE13213.Surv_AllMethods_Freq_2 | trial_29 | 0.656392 | 0.621616 | 0.674953 | 0.617462 | 0.658068 | 0.669727 |
| GSE13213.Surv_AllMethods_Freq_3 | trial_29 | 0.668306 | 0.637978 | 0.687022 | 0.624932 | 0.665096 | 0.677801 |
| GSE13213.Surv_AllMethods_Freq_4 | trial_29 | 0.650173 | 0.620725 | 0.663212 | 0.597064 | 0.631261 | 0.681693 |
| GSE13213.Surv_AllMethods_Freq_5 | trial_29 | 0.548829 | 0.541262 | 0.58538 | 0.542833 | 0.600228 | 0.624786 |
| GSE13213.Surv_AllMethods_Freq_6 | trial_29 | 0.597092 | 0.583333 | 0.515057 | 0.600208 | 0.551402 | 0.562305 |
| GSE13213.Surv_AllMethods_Freq_2 | trial_30 | 0.662892 | 0.621616 | 0.674953 | 0.617462 | 0.658068 | 0.669727 |
| GSE13213.Surv_AllMethods_Freq_3 | trial_30 | 0.675205 | 0.637978 | 0.687022 | 0.624932 | 0.665096 | 0.677801 |
| GSE13213.Surv_AllMethods_Freq_4 | trial_30 | 0.658463 | 0.620725 | 0.663212 | 0.597064 | 0.631261 | 0.681693 |
| GSE13213.Surv_AllMethods_Freq_5 | trial_30 | 0.555111 | 0.541262 | 0.58538 | 0.542833 | 0.600228 | 0.624786 |
| GSE13213.Surv_AllMethods_Freq_6 | trial_30 | 0.594496 | 0.583333 | 0.515057 | 0.600208 | 0.551402 | 0.562305 |
| GSE13213.Surv_AllMethods_Freq_2 | trial_31 | 0.660346 | 0.621616 | 0.674953 | 0.617462 | 0.658068 | 0.669727 |
| GSE13213.Surv_AllMethods_Freq_3 | trial_31 | 0.672609 | 0.637978 | 0.687022 | 0.624932 | 0.665096 | 0.677801 |
| GSE13213.Surv_AllMethods_Freq_4 | trial_31 | 0.652936 | 0.620725 | 0.663212 | 0.597064 | 0.631261 | 0.681693 |
| GSE13213.Surv_AllMethods_Freq_5 | trial_31 | 0.552256 | 0.541262 | 0.58538 | 0.542833 | 0.600228 | 0.624786 |
| GSE13213.Surv_AllMethods_Freq_6 | trial_31 | 0.596054 | 0.583333 | 0.515057 | 0.600208 | 0.551402 | 0.562305 |
| GSE13213.Surv_AllMethods_Freq_2 | trial_32 | 0.657934 | 0.621616 | 0.674953 | 0.617462 | 0.658068 | 0.669727 |
| GSE13213.Surv_AllMethods_Freq_3 | trial_32 | 0.670082 | 0.637978 | 0.687022 | 0.624932 | 0.665096 | 0.677801 |
| GSE13213.Surv_AllMethods_Freq_4 | trial_32 | 0.64905 | 0.620725 | 0.663212 | 0.597064 | 0.631261 | 0.681693 |
| GSE13213.Surv_AllMethods_Freq_5 | trial_32 | 0.552541 | 0.541262 | 0.58538 | 0.542833 | 0.600228 | 0.624786 |
| GSE13213.Surv_AllMethods_Freq_6 | trial_32 | 0.599429 | 0.583333 | 0.515057 | 0.600208 | 0.551402 | 0.562305 |
| GSE13213.Surv_AllMethods_Freq_2 | trial_33 | 0.662021 | 0.621616 | 0.674953 | 0.617462 | 0.658068 | 0.669727 |
| GSE13213.Surv_AllMethods_Freq_3 | trial_33 | 0.674385 | 0.637978 | 0.687022 | 0.624932 | 0.665096 | 0.677801 |
| GSE13213.Surv_AllMethods_Freq_4 | trial_33 | 0.654318 | 0.620725 | 0.663212 | 0.597064 | 0.631261 | 0.681693 |
| GSE13213.Surv_AllMethods_Freq_5 | trial_33 | 0.550971 | 0.541262 | 0.58538 | 0.542833 | 0.600228 | 0.624786 |
| GSE13213.Surv_AllMethods_Freq_6 | trial_33 | 0.599169 | 0.583333 | 0.515057 | 0.600208 | 0.551402 | 0.562305 |
| GSE13213.Surv_AllMethods_Freq_2 | trial_34 | 0.657934 | 0.621616 | 0.674953 | 0.617462 | 0.658068 | 0.669727 |
| GSE13213.Surv_AllMethods_Freq_3 | trial_34 | 0.670082 | 0.637978 | 0.687022 | 0.624932 | 0.665096 | 0.677801 |
| GSE13213.Surv_AllMethods_Freq_4 | trial_34 | 0.650432 | 0.620725 | 0.663212 | 0.597064 | 0.631261 | 0.681693 |
| GSE13213.Surv_AllMethods_Freq_5 | trial_34 | 0.552684 | 0.541262 | 0.58538 | 0.542833 | 0.600228 | 0.624786 |
| GSE13213.Surv_AllMethods_Freq_6 | trial_34 | 0.595794 | 0.583333 | 0.515057 | 0.600208 | 0.551402 | 0.562305 |
| GSE13213.Surv_AllMethods_Freq_2 | trial_35 | 0.655722 | 0.621616 | 0.674953 | 0.617462 | 0.658068 | 0.669727 |
| GSE13213.Surv_AllMethods_Freq_3 | trial_35 | 0.667896 | 0.637978 | 0.687022 | 0.624932 | 0.665096 | 0.677801 |
| GSE13213.Surv_AllMethods_Freq_4 | trial_35 | 0.647755 | 0.620725 | 0.663212 | 0.597064 | 0.631261 | 0.681693 |
| GSE13213.Surv_AllMethods_Freq_5 | trial_35 | 0.549686 | 0.541262 | 0.58538 | 0.542833 | 0.600228 | 0.624786 |
| GSE13213.Surv_AllMethods_Freq_6 | trial_35 | 0.597092 | 0.583333 | 0.515057 | 0.600208 | 0.551402 | 0.562305 |
| GSE13213.Surv_AllMethods_Freq_2 | trial_36 | 0.65532 | 0.621616 | 0.674953 | 0.617462 | 0.658068 | 0.669727 |
| GSE13213.Surv_AllMethods_Freq_3 | trial_36 | 0.668033 | 0.637978 | 0.687022 | 0.624932 | 0.665096 | 0.677801 |
| GSE13213.Surv_AllMethods_Freq_4 | trial_36 | 0.648359 | 0.620725 | 0.663212 | 0.597064 | 0.631261 | 0.681693 |
| GSE13213.Surv_AllMethods_Freq_5 | trial_36 | 0.546545 | 0.541262 | 0.58538 | 0.542833 | 0.600228 | 0.624786 |
| GSE13213.Surv_AllMethods_Freq_6 | trial_36 | 0.599948 | 0.583333 | 0.515057 | 0.600208 | 0.551402 | 0.562305 |
| GSE13213.Surv_AllMethods_Freq_2 | trial_37 | 0.657531 | 0.621616 | 0.674953 | 0.617462 | 0.658068 | 0.669727 |
| GSE13213.Surv_AllMethods_Freq_3 | trial_37 | 0.670287 | 0.637978 | 0.687022 | 0.624932 | 0.665096 | 0.677801 |
| GSE13213.Surv_AllMethods_Freq_4 | trial_37 | 0.648964 | 0.620725 | 0.663212 | 0.597064 | 0.631261 | 0.681693 |
| GSE13213.Surv_AllMethods_Freq_5 | trial_37 | 0.549543 | 0.541262 | 0.58538 | 0.542833 | 0.600228 | 0.624786 |
| GSE13213.Surv_AllMethods_Freq_6 | trial_37 | 0.599429 | 0.583333 | 0.515057 | 0.600208 | 0.551402 | 0.562305 |
| GSE13213.Surv_AllMethods_Freq_2 | trial_38 | 0.658872 | 0.621616 | 0.674953 | 0.617462 | 0.658068 | 0.669727 |
| GSE13213.Surv_AllMethods_Freq_3 | trial_38 | 0.670765 | 0.637978 | 0.687022 | 0.624932 | 0.665096 | 0.677801 |
| GSE13213.Surv_AllMethods_Freq_4 | trial_38 | 0.648877 | 0.620725 | 0.663212 | 0.597064 | 0.631261 | 0.681693 |
| GSE13213.Surv_AllMethods_Freq_5 | trial_38 | 0.550828 | 0.541262 | 0.58538 | 0.542833 | 0.600228 | 0.624786 |
| GSE13213.Surv_AllMethods_Freq_6 | trial_38 | 0.59839 | 0.583333 | 0.515057 | 0.600208 | 0.551402 | 0.562305 |
| GSE13213.Surv_AllMethods_Freq_2 | trial_39 | 0.657531 | 0.621616 | 0.674953 | 0.617462 | 0.658068 | 0.669727 |
| GSE13213.Surv_AllMethods_Freq_3 | trial_39 | 0.669945 | 0.637978 | 0.687022 | 0.624932 | 0.665096 | 0.677801 |
| GSE13213.Surv_AllMethods_Freq_4 | trial_39 | 0.649136 | 0.620725 | 0.663212 | 0.597064 | 0.631261 | 0.681693 |
| GSE13213.Surv_AllMethods_Freq_5 | trial_39 | 0.547116 | 0.541262 | 0.58538 | 0.542833 | 0.600228 | 0.624786 |
| GSE13213.Surv_AllMethods_Freq_6 | trial_39 | 0.6054 | 0.583333 | 0.515057 | 0.600208 | 0.551402 | 0.562305 |
| GSE13213.Surv_AllMethods_Freq_2 | trial_40 | 0.654382 | 0.621616 | 0.674953 | 0.617462 | 0.658068 | 0.669727 |
| GSE13213.Surv_AllMethods_Freq_3 | trial_40 | 0.666598 | 0.637978 | 0.687022 | 0.624932 | 0.665096 | 0.677801 |
| GSE13213.Surv_AllMethods_Freq_4 | trial_40 | 0.646632 | 0.620725 | 0.663212 | 0.597064 | 0.631261 | 0.681693 |
| GSE13213.Surv_AllMethods_Freq_5 | trial_40 | 0.545974 | 0.541262 | 0.58538 | 0.542833 | 0.600228 | 0.624786 |
| GSE13213.Surv_AllMethods_Freq_6 | trial_40 | 0.606179 | 0.583333 | 0.515057 | 0.600208 | 0.551402 | 0.562305 |
| GSE13213.Surv_AllMethods_Freq_2 | trial_41 | 0.654784 | 0.621616 | 0.674953 | 0.617462 | 0.658068 | 0.669727 |
| GSE13213.Surv_AllMethods_Freq_3 | trial_41 | 0.667486 | 0.637978 | 0.687022 | 0.624932 | 0.665096 | 0.677801 |
| GSE13213.Surv_AllMethods_Freq_4 | trial_41 | 0.646114 | 0.620725 | 0.663212 | 0.597064 | 0.631261 | 0.681693 |
| GSE13213.Surv_AllMethods_Freq_5 | trial_41 | 0.546688 | 0.541262 | 0.58538 | 0.542833 | 0.600228 | 0.624786 |
| GSE13213.Surv_AllMethods_Freq_6 | trial_41 | 0.601765 | 0.583333 | 0.515057 | 0.600208 | 0.551402 | 0.562305 |
| GSE13213.Surv_AllMethods_Freq_2 | trial_42 | 0.65465 | 0.621616 | 0.674953 | 0.617462 | 0.658068 | 0.669727 |
| GSE13213.Surv_AllMethods_Freq_3 | trial_42 | 0.666462 | 0.637978 | 0.687022 | 0.624932 | 0.665096 | 0.677801 |
| GSE13213.Surv_AllMethods_Freq_4 | trial_42 | 0.645682 | 0.620725 | 0.663212 | 0.597064 | 0.631261 | 0.681693 |
| GSE13213.Surv_AllMethods_Freq_5 | trial_42 | 0.5504 | 0.541262 | 0.58538 | 0.542833 | 0.600228 | 0.624786 |
| GSE13213.Surv_AllMethods_Freq_6 | trial_42 | 0.600208 | 0.583333 | 0.515057 | 0.600208 | 0.551402 | 0.562305 |
| GSE13213.Surv_AllMethods_Freq_2 | trial_43 | 0.657062 | 0.621616 | 0.674953 | 0.617462 | 0.658068 | 0.669727 |
| GSE13213.Surv_AllMethods_Freq_3 | trial_43 | 0.669262 | 0.637978 | 0.687022 | 0.624932 | 0.665096 | 0.677801 |
| GSE13213.Surv_AllMethods_Freq_4 | trial_43 | 0.648618 | 0.620725 | 0.663212 | 0.597064 | 0.631261 | 0.681693 |
| GSE13213.Surv_AllMethods_Freq_5 | trial_43 | 0.545545 | 0.541262 | 0.58538 | 0.542833 | 0.600228 | 0.624786 |
| GSE13213.Surv_AllMethods_Freq_6 | trial_43 | 0.599948 | 0.583333 | 0.515057 | 0.600208 | 0.551402 | 0.562305 |
| GSE13213.Surv_AllMethods_Freq_2 | trial_44 | 0.659609 | 0.621616 | 0.674953 | 0.617462 | 0.658068 | 0.669727 |
| GSE13213.Surv_AllMethods_Freq_3 | trial_44 | 0.671516 | 0.637978 | 0.687022 | 0.624932 | 0.665096 | 0.677801 |
| GSE13213.Surv_AllMethods_Freq_4 | trial_44 | 0.651036 | 0.620725 | 0.663212 | 0.597064 | 0.631261 | 0.681693 |
| GSE13213.Surv_AllMethods_Freq_5 | trial_44 | 0.551542 | 0.541262 | 0.58538 | 0.542833 | 0.600228 | 0.624786 |
| GSE13213.Surv_AllMethods_Freq_6 | trial_44 | 0.597352 | 0.583333 | 0.515057 | 0.600208 | 0.551402 | 0.562305 |
| GSE13213.Surv_AllMethods_Freq_2 | trial_45 | 0.655052 | 0.621616 | 0.674953 | 0.617462 | 0.658068 | 0.669727 |
| GSE13213.Surv_AllMethods_Freq_3 | trial_45 | 0.667281 | 0.637978 | 0.687022 | 0.624932 | 0.665096 | 0.677801 |
| GSE13213.Surv_AllMethods_Freq_4 | trial_45 | 0.646546 | 0.620725 | 0.663212 | 0.597064 | 0.631261 | 0.681693 |
| GSE13213.Surv_AllMethods_Freq_5 | trial_45 | 0.54526 | 0.541262 | 0.58538 | 0.542833 | 0.600228 | 0.624786 |
| GSE13213.Surv_AllMethods_Freq_6 | trial_45 | 0.601765 | 0.583333 | 0.515057 | 0.600208 | 0.551402 | 0.562305 |
| GSE13213.Surv_AllMethods_Freq_2 | trial_46 | 0.658604 | 0.621616 | 0.674953 | 0.617462 | 0.658068 | 0.669727 |
| GSE13213.Surv_AllMethods_Freq_3 | trial_46 | 0.670833 | 0.637978 | 0.687022 | 0.624932 | 0.665096 | 0.677801 |
| GSE13213.Surv_AllMethods_Freq_4 | trial_46 | 0.651382 | 0.620725 | 0.663212 | 0.597064 | 0.631261 | 0.681693 |
| GSE13213.Surv_AllMethods_Freq_5 | trial_46 | 0.548401 | 0.541262 | 0.58538 | 0.542833 | 0.600228 | 0.624786 |
| GSE13213.Surv_AllMethods_Freq_6 | trial_46 | 0.600467 | 0.583333 | 0.515057 | 0.600208 | 0.551402 | 0.562305 |
| GSE13213.Surv_AllMethods_Freq_2 | trial_47 | 0.65733 | 0.621616 | 0.674953 | 0.617462 | 0.658068 | 0.669727 |
| GSE13213.Surv_AllMethods_Freq_3 | trial_47 | 0.669467 | 0.637978 | 0.687022 | 0.624932 | 0.665096 | 0.677801 |
| GSE13213.Surv_AllMethods_Freq_4 | trial_47 | 0.652677 | 0.620725 | 0.663212 | 0.597064 | 0.631261 | 0.681693 |
| GSE13213.Surv_AllMethods_Freq_5 | trial_47 | 0.553255 | 0.541262 | 0.58538 | 0.542833 | 0.600228 | 0.624786 |
| GSE13213.Surv_AllMethods_Freq_6 | trial_47 | 0.594496 | 0.583333 | 0.515057 | 0.600208 | 0.551402 | 0.562305 |
| GSE13213.Surv_AllMethods_Freq_2 | trial_48 | 0.659475 | 0.621616 | 0.674953 | 0.617462 | 0.658068 | 0.669727 |
| GSE13213.Surv_AllMethods_Freq_3 | trial_48 | 0.671721 | 0.637978 | 0.687022 | 0.624932 | 0.665096 | 0.677801 |
| GSE13213.Surv_AllMethods_Freq_4 | trial_48 | 0.656563 | 0.620725 | 0.663212 | 0.597064 | 0.631261 | 0.681693 |
| GSE13213.Surv_AllMethods_Freq_5 | trial_48 | 0.55454 | 0.541262 | 0.58538 | 0.542833 | 0.600228 | 0.624786 |
| GSE13213.Surv_AllMethods_Freq_6 | trial_48 | 0.600208 | 0.583333 | 0.515057 | 0.600208 | 0.551402 | 0.562305 |
| GSE13213.Surv_AllMethods_Freq_2 | trial_49 | 0.659207 | 0.621616 | 0.674953 | 0.617462 | 0.658068 | 0.669727 |
| GSE13213.Surv_AllMethods_Freq_3 | trial_49 | 0.671038 | 0.637978 | 0.687022 | 0.624932 | 0.665096 | 0.677801 |
| GSE13213.Surv_AllMethods_Freq_4 | trial_49 | 0.650604 | 0.620725 | 0.663212 | 0.597064 | 0.631261 | 0.681693 |
| GSE13213.Surv_AllMethods_Freq_5 | trial_49 | 0.552684 | 0.541262 | 0.58538 | 0.542833 | 0.600228 | 0.624786 |
| GSE13213.Surv_AllMethods_Freq_6 | trial_49 | 0.595535 | 0.583333 | 0.515057 | 0.600208 | 0.551402 | 0.562305 |
| GSE13213.Surv_AllMethods_Freq_2 | trial_50 | 0.656191 | 0.621616 | 0.674953 | 0.617462 | 0.658068 | 0.669727 |
| GSE13213.Surv_AllMethods_Freq_3 | trial_50 | 0.668238 | 0.637978 | 0.687022 | 0.624932 | 0.665096 | 0.677801 |
| GSE13213.Surv_AllMethods_Freq_4 | trial_50 | 0.648705 | 0.620725 | 0.663212 | 0.597064 | 0.631261 | 0.681693 |
| GSE13213.Surv_AllMethods_Freq_5 | trial_50 | 0.550828 | 0.541262 | 0.58538 | 0.542833 | 0.600228 | 0.624786 |
| GSE13213.Surv_AllMethods_Freq_6 | trial_50 | 0.603063 | 0.583333 | 0.515057 | 0.600208 | 0.551402 | 0.562305 |
| GSE13213.Surv_AllMethods_Freq_2 | trial_51 | 0.657263 | 0.621616 | 0.674953 | 0.617462 | 0.658068 | 0.669727 |
| GSE13213.Surv_AllMethods_Freq_3 | trial_51 | 0.669331 | 0.637978 | 0.687022 | 0.624932 | 0.665096 | 0.677801 |
| GSE13213.Surv_AllMethods_Freq_4 | trial_51 | 0.649827 | 0.620725 | 0.663212 | 0.597064 | 0.631261 | 0.681693 |
| GSE13213.Surv_AllMethods_Freq_5 | trial_51 | 0.553113 | 0.541262 | 0.58538 | 0.542833 | 0.600228 | 0.624786 |
| GSE13213.Surv_AllMethods_Freq_6 | trial_51 | 0.599169 | 0.583333 | 0.515057 | 0.600208 | 0.551402 | 0.562305 |
| GSE13213.Surv_AllMethods_Freq_2 | trial_52 | 0.657531 | 0.621616 | 0.674953 | 0.617462 | 0.658068 | 0.669727 |
| GSE13213.Surv_AllMethods_Freq_3 | trial_52 | 0.669331 | 0.637978 | 0.687022 | 0.624932 | 0.665096 | 0.677801 |
| GSE13213.Surv_AllMethods_Freq_4 | trial_52 | 0.6481 | 0.620725 | 0.663212 | 0.597064 | 0.631261 | 0.681693 |
| GSE13213.Surv_AllMethods_Freq_5 | trial_52 | 0.548258 | 0.541262 | 0.58538 | 0.542833 | 0.600228 | 0.624786 |
| GSE13213.Surv_AllMethods_Freq_6 | trial_52 | 0.601506 | 0.583333 | 0.515057 | 0.600208 | 0.551402 | 0.562305 |
| GSE13213.Surv_AllMethods_Freq_2 | trial_53 | 0.658939 | 0.621616 | 0.674953 | 0.617462 | 0.658068 | 0.669727 |
| GSE13213.Surv_AllMethods_Freq_3 | trial_53 | 0.671311 | 0.637978 | 0.687022 | 0.624932 | 0.665096 | 0.677801 |
| GSE13213.Surv_AllMethods_Freq_4 | trial_53 | 0.652245 | 0.620725 | 0.663212 | 0.597064 | 0.631261 | 0.681693 |
| GSE13213.Surv_AllMethods_Freq_5 | trial_53 | 0.550114 | 0.541262 | 0.58538 | 0.542833 | 0.600228 | 0.624786 |
| GSE13213.Surv_AllMethods_Freq_6 | trial_53 | 0.599688 | 0.583333 | 0.515057 | 0.600208 | 0.551402 | 0.562305 |
| GSE13213.Surv_AllMethods_Freq_2 | trial_54 | 0.654248 | 0.621616 | 0.674953 | 0.617462 | 0.658068 | 0.669727 |
| GSE13213.Surv_AllMethods_Freq_3 | trial_54 | 0.666667 | 0.637978 | 0.687022 | 0.624932 | 0.665096 | 0.677801 |
| GSE13213.Surv_AllMethods_Freq_4 | trial_54 | 0.646459 | 0.620725 | 0.663212 | 0.597064 | 0.631261 | 0.681693 |
| GSE13213.Surv_AllMethods_Freq_5 | trial_54 | 0.547973 | 0.541262 | 0.58538 | 0.542833 | 0.600228 | 0.624786 |
| GSE13213.Surv_AllMethods_Freq_6 | trial_54 | 0.607477 | 0.583333 | 0.515057 | 0.600208 | 0.551402 | 0.562305 |
| GSE13213.Surv_AllMethods_Freq_2 | trial_55 | 0.657867 | 0.621616 | 0.674953 | 0.617462 | 0.658068 | 0.669727 |
| GSE13213.Surv_AllMethods_Freq_3 | trial_55 | 0.67015 | 0.637978 | 0.687022 | 0.624932 | 0.665096 | 0.677801 |
| GSE13213.Surv_AllMethods_Freq_4 | trial_55 | 0.648273 | 0.620725 | 0.663212 | 0.597064 | 0.631261 | 0.681693 |
| GSE13213.Surv_AllMethods_Freq_5 | trial_55 | 0.547259 | 0.541262 | 0.58538 | 0.542833 | 0.600228 | 0.624786 |
| GSE13213.Surv_AllMethods_Freq_6 | trial_55 | 0.603063 | 0.583333 | 0.515057 | 0.600208 | 0.551402 | 0.562305 |
| GSE13213.Surv_AllMethods_Freq_2 | trial_56 | 0.653176 | 0.621616 | 0.674953 | 0.617462 | 0.658068 | 0.669727 |
| GSE13213.Surv_AllMethods_Freq_3 | trial_56 | 0.665164 | 0.637978 | 0.687022 | 0.624932 | 0.665096 | 0.677801 |
| GSE13213.Surv_AllMethods_Freq_4 | trial_56 | 0.645941 | 0.620725 | 0.663212 | 0.597064 | 0.631261 | 0.681693 |
| GSE13213.Surv_AllMethods_Freq_5 | trial_56 | 0.54783 | 0.541262 | 0.58538 | 0.542833 | 0.600228 | 0.624786 |
| GSE13213.Surv_AllMethods_Freq_6 | trial_56 | 0.59891 | 0.583333 | 0.515057 | 0.600208 | 0.551402 | 0.562305 |
| GSE13213.Surv_AllMethods_Freq_2 | trial_57 | 0.654985 | 0.621616 | 0.674953 | 0.617462 | 0.658068 | 0.669727 |
| GSE13213.Surv_AllMethods_Freq_3 | trial_57 | 0.667623 | 0.637978 | 0.687022 | 0.624932 | 0.665096 | 0.677801 |
| GSE13213.Surv_AllMethods_Freq_4 | trial_57 | 0.648187 | 0.620725 | 0.663212 | 0.597064 | 0.631261 | 0.681693 |
| GSE13213.Surv_AllMethods_Freq_5 | trial_57 | 0.543975 | 0.541262 | 0.58538 | 0.542833 | 0.600228 | 0.624786 |
| GSE13213.Surv_AllMethods_Freq_6 | trial_57 | 0.604102 | 0.583333 | 0.515057 | 0.600208 | 0.551402 | 0.562305 |
| GSE13213.Surv_AllMethods_Freq_2 | trial_58 | 0.660882 | 0.621616 | 0.674953 | 0.617462 | 0.658068 | 0.669727 |
| GSE13213.Surv_AllMethods_Freq_3 | trial_58 | 0.673019 | 0.637978 | 0.687022 | 0.624932 | 0.665096 | 0.677801 |
| GSE13213.Surv_AllMethods_Freq_4 | trial_58 | 0.652418 | 0.620725 | 0.663212 | 0.597064 | 0.631261 | 0.681693 |
| GSE13213.Surv_AllMethods_Freq_5 | trial_58 | 0.552399 | 0.541262 | 0.58538 | 0.542833 | 0.600228 | 0.624786 |
| GSE13213.Surv_AllMethods_Freq_6 | trial_58 | 0.597352 | 0.583333 | 0.515057 | 0.600208 | 0.551402 | 0.562305 |
| GSE13213.Surv_AllMethods_Freq_2 | trial_59 | 0.660748 | 0.621616 | 0.674953 | 0.617462 | 0.658068 | 0.669727 |
| GSE13213.Surv_AllMethods_Freq_3 | trial_59 | 0.672951 | 0.637978 | 0.687022 | 0.624932 | 0.665096 | 0.677801 |
| GSE13213.Surv_AllMethods_Freq_4 | trial_59 | 0.652073 | 0.620725 | 0.663212 | 0.597064 | 0.631261 | 0.681693 |
| GSE13213.Surv_AllMethods_Freq_5 | trial_59 | 0.551828 | 0.541262 | 0.58538 | 0.542833 | 0.600228 | 0.624786 |
| GSE13213.Surv_AllMethods_Freq_6 | trial_59 | 0.602025 | 0.583333 | 0.515057 | 0.600208 | 0.551402 | 0.562305 |
| GSE13213.Surv_AllMethods_Freq_2 | trial_60 | 0.656124 | 0.621616 | 0.674953 | 0.617462 | 0.658068 | 0.669727 |
| GSE13213.Surv_AllMethods_Freq_3 | trial_60 | 0.668238 | 0.637978 | 0.687022 | 0.624932 | 0.665096 | 0.677801 |
| GSE13213.Surv_AllMethods_Freq_4 | trial_60 | 0.648187 | 0.620725 | 0.663212 | 0.597064 | 0.631261 | 0.681693 |
| GSE13213.Surv_AllMethods_Freq_5 | trial_60 | 0.54783 | 0.541262 | 0.58538 | 0.542833 | 0.600228 | 0.624786 |
| GSE13213.Surv_AllMethods_Freq_6 | trial_60 | 0.599688 | 0.583333 | 0.515057 | 0.600208 | 0.551402 | 0.562305 |
| GSE13213.Surv_AllMethods_Freq_2 | trial_61 | 0.653645 | 0.621616 | 0.674953 | 0.617462 | 0.658068 | 0.669727 |
| GSE13213.Surv_AllMethods_Freq_3 | trial_61 | 0.665847 | 0.637978 | 0.687022 | 0.624932 | 0.665096 | 0.677801 |
| GSE13213.Surv_AllMethods_Freq_4 | trial_61 | 0.648273 | 0.620725 | 0.663212 | 0.597064 | 0.631261 | 0.681693 |
| GSE13213.Surv_AllMethods_Freq_5 | trial_61 | 0.5504 | 0.541262 | 0.58538 | 0.542833 | 0.600228 | 0.624786 |
| GSE13213.Surv_AllMethods_Freq_6 | trial_61 | 0.597092 | 0.583333 | 0.515057 | 0.600208 | 0.551402 | 0.562305 |
| GSE13213.Surv_AllMethods_Freq_2 | trial_62 | 0.6578 | 0.621616 | 0.674953 | 0.617462 | 0.658068 | 0.669727 |
| GSE13213.Surv_AllMethods_Freq_3 | trial_62 | 0.669604 | 0.637978 | 0.687022 | 0.624932 | 0.665096 | 0.677801 |
| GSE13213.Surv_AllMethods_Freq_4 | trial_62 | 0.6481 | 0.620725 | 0.663212 | 0.597064 | 0.631261 | 0.681693 |
| GSE13213.Surv_AllMethods_Freq_5 | trial_62 | 0.549543 | 0.541262 | 0.58538 | 0.542833 | 0.600228 | 0.624786 |
| GSE13213.Surv_AllMethods_Freq_6 | trial_62 | 0.5919 | 0.583333 | 0.515057 | 0.600208 | 0.551402 | 0.562305 |
| GSE13213.Surv_AllMethods_Freq_2 | trial_63 | 0.658135 | 0.621616 | 0.674953 | 0.617462 | 0.658068 | 0.669727 |
| GSE13213.Surv_AllMethods_Freq_3 | trial_63 | 0.670902 | 0.637978 | 0.687022 | 0.624932 | 0.665096 | 0.677801 |
| GSE13213.Surv_AllMethods_Freq_4 | trial_63 | 0.649223 | 0.620725 | 0.663212 | 0.597064 | 0.631261 | 0.681693 |
| GSE13213.Surv_AllMethods_Freq_5 | trial_63 | 0.549258 | 0.541262 | 0.58538 | 0.542833 | 0.600228 | 0.624786 |
| GSE13213.Surv_AllMethods_Freq_6 | trial_63 | 0.59865 | 0.583333 | 0.515057 | 0.600208 | 0.551402 | 0.562305 |
| GSE13213.Surv_AllMethods_Freq_2 | trial_64 | 0.657062 | 0.621616 | 0.674953 | 0.617462 | 0.658068 | 0.669727 |
| GSE13213.Surv_AllMethods_Freq_3 | trial_64 | 0.669331 | 0.637978 | 0.687022 | 0.624932 | 0.665096 | 0.677801 |
| GSE13213.Surv_AllMethods_Freq_4 | trial_64 | 0.649396 | 0.620725 | 0.663212 | 0.597064 | 0.631261 | 0.681693 |
| GSE13213.Surv_AllMethods_Freq_5 | trial_64 | 0.553541 | 0.541262 | 0.58538 | 0.542833 | 0.600228 | 0.624786 |
| GSE13213.Surv_AllMethods_Freq_6 | trial_64 | 0.596833 | 0.583333 | 0.515057 | 0.600208 | 0.551402 | 0.562305 |
| GSE13213.Surv_AllMethods_Freq_2 | trial_65 | 0.654315 | 0.621616 | 0.674953 | 0.617462 | 0.658068 | 0.669727 |
| GSE13213.Surv_AllMethods_Freq_3 | trial_65 | 0.666393 | 0.637978 | 0.687022 | 0.624932 | 0.665096 | 0.677801 |
| GSE13213.Surv_AllMethods_Freq_4 | trial_65 | 0.647496 | 0.620725 | 0.663212 | 0.597064 | 0.631261 | 0.681693 |
| GSE13213.Surv_AllMethods_Freq_5 | trial_65 | 0.547687 | 0.541262 | 0.58538 | 0.542833 | 0.600228 | 0.624786 |
| GSE13213.Surv_AllMethods_Freq_6 | trial_65 | 0.59839 | 0.583333 | 0.515057 | 0.600208 | 0.551402 | 0.562305 |
| GSE13213.Surv_AllMethods_Freq_2 | trial_66 | 0.65981 | 0.621616 | 0.674953 | 0.617462 | 0.658068 | 0.669727 |
| GSE13213.Surv_AllMethods_Freq_3 | trial_66 | 0.672131 | 0.637978 | 0.687022 | 0.624932 | 0.665096 | 0.677801 |
| GSE13213.Surv_AllMethods_Freq_4 | trial_66 | 0.654145 | 0.620725 | 0.663212 | 0.597064 | 0.631261 | 0.681693 |
| GSE13213.Surv_AllMethods_Freq_5 | trial_66 | 0.553113 | 0.541262 | 0.58538 | 0.542833 | 0.600228 | 0.624786 |
| GSE13213.Surv_AllMethods_Freq_6 | trial_66 | 0.59891 | 0.583333 | 0.515057 | 0.600208 | 0.551402 | 0.562305 |
| GSE13213.Surv_AllMethods_Freq_2 | trial_67 | 0.659073 | 0.621616 | 0.674953 | 0.617462 | 0.658068 | 0.669727 |
| GSE13213.Surv_AllMethods_Freq_3 | trial_67 | 0.67138 | 0.637978 | 0.687022 | 0.624932 | 0.665096 | 0.677801 |
| GSE13213.Surv_AllMethods_Freq_4 | trial_67 | 0.650864 | 0.620725 | 0.663212 | 0.597064 | 0.631261 | 0.681693 |
| GSE13213.Surv_AllMethods_Freq_5 | trial_67 | 0.552541 | 0.541262 | 0.58538 | 0.542833 | 0.600228 | 0.624786 |
| GSE13213.Surv_AllMethods_Freq_6 | trial_67 | 0.593458 | 0.583333 | 0.515057 | 0.600208 | 0.551402 | 0.562305 |
| GSE13213.Surv_AllMethods_Freq_2 | trial_68 | 0.656794 | 0.621616 | 0.674953 | 0.617462 | 0.658068 | 0.669727 |
| GSE13213.Surv_AllMethods_Freq_3 | trial_68 | 0.669057 | 0.637978 | 0.687022 | 0.624932 | 0.665096 | 0.677801 |
| GSE13213.Surv_AllMethods_Freq_4 | trial_68 | 0.648964 | 0.620725 | 0.663212 | 0.597064 | 0.631261 | 0.681693 |
| GSE13213.Surv_AllMethods_Freq_5 | trial_68 | 0.547401 | 0.541262 | 0.58538 | 0.542833 | 0.600228 | 0.624786 |
| GSE13213.Surv_AllMethods_Freq_6 | trial_68 | 0.599688 | 0.583333 | 0.515057 | 0.600208 | 0.551402 | 0.562305 |
| GSE13213.Surv_AllMethods_Freq_2 | trial_69 | 0.655789 | 0.621616 | 0.674953 | 0.617462 | 0.658068 | 0.669727 |
| GSE13213.Surv_AllMethods_Freq_3 | trial_69 | 0.668443 | 0.637978 | 0.687022 | 0.624932 | 0.665096 | 0.677801 |
| GSE13213.Surv_AllMethods_Freq_4 | trial_69 | 0.650864 | 0.620725 | 0.663212 | 0.597064 | 0.631261 | 0.681693 |
| GSE13213.Surv_AllMethods_Freq_5 | trial_69 | 0.552256 | 0.541262 | 0.58538 | 0.542833 | 0.600228 | 0.624786 |
| GSE13213.Surv_AllMethods_Freq_6 | trial_69 | 0.597352 | 0.583333 | 0.515057 | 0.600208 | 0.551402 | 0.562305 |
| GSE13213.Surv_AllMethods_Freq_2 | trial_70 | 0.653779 | 0.621616 | 0.674953 | 0.617462 | 0.658068 | 0.669727 |
| GSE13213.Surv_AllMethods_Freq_3 | trial_70 | 0.666052 | 0.637978 | 0.687022 | 0.624932 | 0.665096 | 0.677801 |
| GSE13213.Surv_AllMethods_Freq_4 | trial_70 | 0.647755 | 0.620725 | 0.663212 | 0.597064 | 0.631261 | 0.681693 |
| GSE13213.Surv_AllMethods_Freq_5 | trial_70 | 0.549971 | 0.541262 | 0.58538 | 0.542833 | 0.600228 | 0.624786 |
| GSE13213.Surv_AllMethods_Freq_6 | trial_70 | 0.597092 | 0.583333 | 0.515057 | 0.600208 | 0.551402 | 0.562305 |
| GSE13213.Surv_AllMethods_Freq_2 | trial_71 | 0.658805 | 0.621616 | 0.674953 | 0.617462 | 0.658068 | 0.669727 |
| GSE13213.Surv_AllMethods_Freq_3 | trial_71 | 0.67138 | 0.637978 | 0.687022 | 0.624932 | 0.665096 | 0.677801 |
| GSE13213.Surv_AllMethods_Freq_4 | trial_71 | 0.652245 | 0.620725 | 0.663212 | 0.597064 | 0.631261 | 0.681693 |
| GSE13213.Surv_AllMethods_Freq_5 | trial_71 | 0.551828 | 0.541262 | 0.58538 | 0.542833 | 0.600228 | 0.624786 |
| GSE13213.Surv_AllMethods_Freq_6 | trial_71 | 0.596573 | 0.583333 | 0.515057 | 0.600208 | 0.551402 | 0.562305 |
| GSE13213.Surv_AllMethods_Freq_2 | trial_72 | 0.658202 | 0.621616 | 0.674953 | 0.617462 | 0.658068 | 0.669727 |
| GSE13213.Surv_AllMethods_Freq_3 | trial_72 | 0.670014 | 0.637978 | 0.687022 | 0.624932 | 0.665096 | 0.677801 |
| GSE13213.Surv_AllMethods_Freq_4 | trial_72 | 0.653195 | 0.620725 | 0.663212 | 0.597064 | 0.631261 | 0.681693 |
| GSE13213.Surv_AllMethods_Freq_5 | trial_72 | 0.556396 | 0.541262 | 0.58538 | 0.542833 | 0.600228 | 0.624786 |
| GSE13213.Surv_AllMethods_Freq_6 | trial_72 | 0.595275 | 0.583333 | 0.515057 | 0.600208 | 0.551402 | 0.562305 |
| GSE13213.Surv_AllMethods_Freq_2 | trial_73 | 0.658001 | 0.621616 | 0.674953 | 0.617462 | 0.658068 | 0.669727 |
| GSE13213.Surv_AllMethods_Freq_3 | trial_73 | 0.670287 | 0.637978 | 0.687022 | 0.624932 | 0.665096 | 0.677801 |
| GSE13213.Surv_AllMethods_Freq_4 | trial_73 | 0.651813 | 0.620725 | 0.663212 | 0.597064 | 0.631261 | 0.681693 |
| GSE13213.Surv_AllMethods_Freq_5 | trial_73 | 0.553255 | 0.541262 | 0.58538 | 0.542833 | 0.600228 | 0.624786 |
| GSE13213.Surv_AllMethods_Freq_6 | trial_73 | 0.595275 | 0.583333 | 0.515057 | 0.600208 | 0.551402 | 0.562305 |
| GSE13213.Surv_AllMethods_Freq_2 | trial_74 | 0.654784 | 0.621616 | 0.674953 | 0.617462 | 0.658068 | 0.669727 |
| GSE13213.Surv_AllMethods_Freq_3 | trial_74 | 0.667008 | 0.637978 | 0.687022 | 0.624932 | 0.665096 | 0.677801 |
| GSE13213.Surv_AllMethods_Freq_4 | trial_74 | 0.648359 | 0.620725 | 0.663212 | 0.597064 | 0.631261 | 0.681693 |
| GSE13213.Surv_AllMethods_Freq_5 | trial_74 | 0.54783 | 0.541262 | 0.58538 | 0.542833 | 0.600228 | 0.624786 |
| GSE13213.Surv_AllMethods_Freq_6 | trial_74 | 0.603063 | 0.583333 | 0.515057 | 0.600208 | 0.551402 | 0.562305 |
| GSE13213.Surv_AllMethods_Freq_2 | trial_75 | 0.657464 | 0.621616 | 0.674953 | 0.617462 | 0.658068 | 0.669727 |
| GSE13213.Surv_AllMethods_Freq_3 | trial_75 | 0.669672 | 0.637978 | 0.687022 | 0.624932 | 0.665096 | 0.677801 |
| GSE13213.Surv_AllMethods_Freq_4 | trial_75 | 0.6481 | 0.620725 | 0.663212 | 0.597064 | 0.631261 | 0.681693 |
| GSE13213.Surv_AllMethods_Freq_5 | trial_75 | 0.54783 | 0.541262 | 0.58538 | 0.542833 | 0.600228 | 0.624786 |
| GSE13213.Surv_AllMethods_Freq_6 | trial_75 | 0.602544 | 0.583333 | 0.515057 | 0.600208 | 0.551402 | 0.562305 |
| GSE13213.Surv_AllMethods_Freq_2 | trial_76 | 0.655722 | 0.621616 | 0.674953 | 0.617462 | 0.658068 | 0.669727 |
| GSE13213.Surv_AllMethods_Freq_3 | trial_76 | 0.668033 | 0.637978 | 0.687022 | 0.624932 | 0.665096 | 0.677801 |
| GSE13213.Surv_AllMethods_Freq_4 | trial_76 | 0.6462 | 0.620725 | 0.663212 | 0.597064 | 0.631261 | 0.681693 |
| GSE13213.Surv_AllMethods_Freq_5 | trial_76 | 0.549686 | 0.541262 | 0.58538 | 0.542833 | 0.600228 | 0.624786 |
| GSE13213.Surv_AllMethods_Freq_6 | trial_76 | 0.603323 | 0.583333 | 0.515057 | 0.600208 | 0.551402 | 0.562305 |
| GSE13213.Surv_AllMethods_Freq_2 | trial_77 | 0.654047 | 0.621616 | 0.674953 | 0.617462 | 0.658068 | 0.669727 |
| GSE13213.Surv_AllMethods_Freq_3 | trial_77 | 0.665915 | 0.637978 | 0.687022 | 0.624932 | 0.665096 | 0.677801 |
| GSE13213.Surv_AllMethods_Freq_4 | trial_77 | 0.648964 | 0.620725 | 0.663212 | 0.597064 | 0.631261 | 0.681693 |
| GSE13213.Surv_AllMethods_Freq_5 | trial_77 | 0.543975 | 0.541262 | 0.58538 | 0.542833 | 0.600228 | 0.624786 |
| GSE13213.Surv_AllMethods_Freq_6 | trial_77 | 0.602285 | 0.583333 | 0.515057 | 0.600208 | 0.551402 | 0.562305 |
| GSE13213.Surv_AllMethods_Freq_2 | trial_78 | 0.657196 | 0.621616 | 0.674953 | 0.617462 | 0.658068 | 0.669727 |
| GSE13213.Surv_AllMethods_Freq_3 | trial_78 | 0.669262 | 0.637978 | 0.687022 | 0.624932 | 0.665096 | 0.677801 |
| GSE13213.Surv_AllMethods_Freq_4 | trial_78 | 0.648618 | 0.620725 | 0.663212 | 0.597064 | 0.631261 | 0.681693 |
| GSE13213.Surv_AllMethods_Freq_5 | trial_78 | 0.546259 | 0.541262 | 0.58538 | 0.542833 | 0.600228 | 0.624786 |
| GSE13213.Surv_AllMethods_Freq_6 | trial_78 | 0.599429 | 0.583333 | 0.515057 | 0.600208 | 0.551402 | 0.562305 |
| GSE13213.Surv_AllMethods_Freq_2 | trial_79 | 0.657263 | 0.621616 | 0.674953 | 0.617462 | 0.658068 | 0.669727 |
| GSE13213.Surv_AllMethods_Freq_3 | trial_79 | 0.669399 | 0.637978 | 0.687022 | 0.624932 | 0.665096 | 0.677801 |
| GSE13213.Surv_AllMethods_Freq_4 | trial_79 | 0.64905 | 0.620725 | 0.663212 | 0.597064 | 0.631261 | 0.681693 |
| GSE13213.Surv_AllMethods_Freq_5 | trial_79 | 0.550971 | 0.541262 | 0.58538 | 0.542833 | 0.600228 | 0.624786 |
| GSE13213.Surv_AllMethods_Freq_6 | trial_79 | 0.597352 | 0.583333 | 0.515057 | 0.600208 | 0.551402 | 0.562305 |
| GSE13213.Surv_AllMethods_Freq_2 | trial_80 | 0.654315 | 0.621616 | 0.674953 | 0.617462 | 0.658068 | 0.669727 |
| GSE13213.Surv_AllMethods_Freq_3 | trial_80 | 0.666667 | 0.637978 | 0.687022 | 0.624932 | 0.665096 | 0.677801 |
| GSE13213.Surv_AllMethods_Freq_4 | trial_80 | 0.6481 | 0.620725 | 0.663212 | 0.597064 | 0.631261 | 0.681693 |
| GSE13213.Surv_AllMethods_Freq_5 | trial_80 | 0.546545 | 0.541262 | 0.58538 | 0.542833 | 0.600228 | 0.624786 |
| GSE13213.Surv_AllMethods_Freq_6 | trial_80 | 0.604881 | 0.583333 | 0.515057 | 0.600208 | 0.551402 | 0.562305 |
| GSE13213.Surv_AllMethods_Freq_2 | trial_81 | 0.656325 | 0.621616 | 0.674953 | 0.617462 | 0.658068 | 0.669727 |
| GSE13213.Surv_AllMethods_Freq_3 | trial_81 | 0.668716 | 0.637978 | 0.687022 | 0.624932 | 0.665096 | 0.677801 |
| GSE13213.Surv_AllMethods_Freq_4 | trial_81 | 0.648964 | 0.620725 | 0.663212 | 0.597064 | 0.631261 | 0.681693 |
| GSE13213.Surv_AllMethods_Freq_5 | trial_81 | 0.546688 | 0.541262 | 0.58538 | 0.542833 | 0.600228 | 0.624786 |
| GSE13213.Surv_AllMethods_Freq_6 | trial_81 | 0.599429 | 0.583333 | 0.515057 | 0.600208 | 0.551402 | 0.562305 |
| GSE13213.Surv_AllMethods_Freq_2 | trial_82 | 0.65847 | 0.621616 | 0.674953 | 0.617462 | 0.658068 | 0.669727 |
| GSE13213.Surv_AllMethods_Freq_3 | trial_82 | 0.67056 | 0.637978 | 0.687022 | 0.624932 | 0.665096 | 0.677801 |
| GSE13213.Surv_AllMethods_Freq_4 | trial_82 | 0.651727 | 0.620725 | 0.663212 | 0.597064 | 0.631261 | 0.681693 |
| GSE13213.Surv_AllMethods_Freq_5 | trial_82 | 0.553398 | 0.541262 | 0.58538 | 0.542833 | 0.600228 | 0.624786 |
| GSE13213.Surv_AllMethods_Freq_6 | trial_82 | 0.597871 | 0.583333 | 0.515057 | 0.600208 | 0.551402 | 0.562305 |
| GSE13213.Surv_AllMethods_Freq_2 | trial_83 | 0.655186 | 0.621616 | 0.674953 | 0.617462 | 0.658068 | 0.669727 |
| GSE13213.Surv_AllMethods_Freq_3 | trial_83 | 0.667213 | 0.637978 | 0.687022 | 0.624932 | 0.665096 | 0.677801 |
| GSE13213.Surv_AllMethods_Freq_4 | trial_83 | 0.648446 | 0.620725 | 0.663212 | 0.597064 | 0.631261 | 0.681693 |
| GSE13213.Surv_AllMethods_Freq_5 | trial_83 | 0.551256 | 0.541262 | 0.58538 | 0.542833 | 0.600228 | 0.624786 |
| GSE13213.Surv_AllMethods_Freq_6 | trial_83 | 0.59839 | 0.583333 | 0.515057 | 0.600208 | 0.551402 | 0.562305 |
| GSE13213.Surv_AllMethods_Freq_2 | trial_84 | 0.656392 | 0.621616 | 0.674953 | 0.617462 | 0.658068 | 0.669727 |
| GSE13213.Surv_AllMethods_Freq_3 | trial_84 | 0.669057 | 0.637978 | 0.687022 | 0.624932 | 0.665096 | 0.677801 |
| GSE13213.Surv_AllMethods_Freq_4 | trial_84 | 0.647668 | 0.620725 | 0.663212 | 0.597064 | 0.631261 | 0.681693 |
| GSE13213.Surv_AllMethods_Freq_5 | trial_84 | 0.550828 | 0.541262 | 0.58538 | 0.542833 | 0.600228 | 0.624786 |
| GSE13213.Surv_AllMethods_Freq_6 | trial_84 | 0.602544 | 0.583333 | 0.515057 | 0.600208 | 0.551402 | 0.562305 |
| GSE13213.Surv_AllMethods_Freq_2 | trial_85 | 0.655722 | 0.621616 | 0.674953 | 0.617462 | 0.658068 | 0.669727 |
| GSE13213.Surv_AllMethods_Freq_3 | trial_85 | 0.667964 | 0.637978 | 0.687022 | 0.624932 | 0.665096 | 0.677801 |
| GSE13213.Surv_AllMethods_Freq_4 | trial_85 | 0.647841 | 0.620725 | 0.663212 | 0.597064 | 0.631261 | 0.681693 |
| GSE13213.Surv_AllMethods_Freq_5 | trial_85 | 0.549115 | 0.541262 | 0.58538 | 0.542833 | 0.600228 | 0.624786 |
| GSE13213.Surv_AllMethods_Freq_6 | trial_85 | 0.596054 | 0.583333 | 0.515057 | 0.600208 | 0.551402 | 0.562305 |
| GSE13213.Surv_AllMethods_Freq_2 | trial_86 | 0.652841 | 0.621616 | 0.674953 | 0.617462 | 0.658068 | 0.669727 |
| GSE13213.Surv_AllMethods_Freq_3 | trial_86 | 0.665301 | 0.637978 | 0.687022 | 0.624932 | 0.665096 | 0.677801 |
| GSE13213.Surv_AllMethods_Freq_4 | trial_86 | 0.645596 | 0.620725 | 0.663212 | 0.597064 | 0.631261 | 0.681693 |
| GSE13213.Surv_AllMethods_Freq_5 | trial_86 | 0.5504 | 0.541262 | 0.58538 | 0.542833 | 0.600228 | 0.624786 |
| GSE13213.Surv_AllMethods_Freq_6 | trial_86 | 0.593718 | 0.583333 | 0.515057 | 0.600208 | 0.551402 | 0.562305 |
| GSE13213.Surv_AllMethods_Freq_2 | trial_87 | 0.661217 | 0.621616 | 0.674953 | 0.617462 | 0.658068 | 0.669727 |
| GSE13213.Surv_AllMethods_Freq_3 | trial_87 | 0.673156 | 0.637978 | 0.687022 | 0.624932 | 0.665096 | 0.677801 |
| GSE13213.Surv_AllMethods_Freq_4 | trial_87 | 0.652677 | 0.620725 | 0.663212 | 0.597064 | 0.631261 | 0.681693 |
| GSE13213.Surv_AllMethods_Freq_5 | trial_87 | 0.552684 | 0.541262 | 0.58538 | 0.542833 | 0.600228 | 0.624786 |
| GSE13213.Surv_AllMethods_Freq_6 | trial_87 | 0.592939 | 0.583333 | 0.515057 | 0.600208 | 0.551402 | 0.562305 |
| GSE13213.Surv_AllMethods_Freq_2 | trial_88 | 0.656727 | 0.621616 | 0.674953 | 0.617462 | 0.658068 | 0.669727 |
| GSE13213.Surv_AllMethods_Freq_3 | trial_88 | 0.669057 | 0.637978 | 0.687022 | 0.624932 | 0.665096 | 0.677801 |
| GSE13213.Surv_AllMethods_Freq_4 | trial_88 | 0.650691 | 0.620725 | 0.663212 | 0.597064 | 0.631261 | 0.681693 |
| GSE13213.Surv_AllMethods_Freq_5 | trial_88 | 0.549686 | 0.541262 | 0.58538 | 0.542833 | 0.600228 | 0.624786 |
| GSE13213.Surv_AllMethods_Freq_6 | trial_88 | 0.595794 | 0.583333 | 0.515057 | 0.600208 | 0.551402 | 0.562305 |
| GSE13213.Surv_AllMethods_Freq_2 | trial_89 | 0.657263 | 0.621616 | 0.674953 | 0.617462 | 0.658068 | 0.669727 |
| GSE13213.Surv_AllMethods_Freq_3 | trial_89 | 0.669536 | 0.637978 | 0.687022 | 0.624932 | 0.665096 | 0.677801 |
| GSE13213.Surv_AllMethods_Freq_4 | trial_89 | 0.648705 | 0.620725 | 0.663212 | 0.597064 | 0.631261 | 0.681693 |
| GSE13213.Surv_AllMethods_Freq_5 | trial_89 | 0.551114 | 0.541262 | 0.58538 | 0.542833 | 0.600228 | 0.624786 |
| GSE13213.Surv_AllMethods_Freq_6 | trial_89 | 0.599948 | 0.583333 | 0.515057 | 0.600208 | 0.551402 | 0.562305 |
| GSE13213.Surv_AllMethods_Freq_2 | trial_90 | 0.659877 | 0.621616 | 0.674953 | 0.617462 | 0.658068 | 0.669727 |
| GSE13213.Surv_AllMethods_Freq_3 | trial_90 | 0.672199 | 0.637978 | 0.687022 | 0.624932 | 0.665096 | 0.677801 |
| GSE13213.Surv_AllMethods_Freq_4 | trial_90 | 0.652677 | 0.620725 | 0.663212 | 0.597064 | 0.631261 | 0.681693 |
| GSE13213.Surv_AllMethods_Freq_5 | trial_90 | 0.550828 | 0.541262 | 0.58538 | 0.542833 | 0.600228 | 0.624786 |
| GSE13213.Surv_AllMethods_Freq_6 | trial_90 | 0.594756 | 0.583333 | 0.515057 | 0.600208 | 0.551402 | 0.562305 |
| GSE13213.Surv_AllMethods_Freq_2 | trial_91 | 0.656928 | 0.621616 | 0.674953 | 0.617462 | 0.658068 | 0.669727 |
| GSE13213.Surv_AllMethods_Freq_3 | trial_91 | 0.669126 | 0.637978 | 0.687022 | 0.624932 | 0.665096 | 0.677801 |
| GSE13213.Surv_AllMethods_Freq_4 | trial_91 | 0.651295 | 0.620725 | 0.663212 | 0.597064 | 0.631261 | 0.681693 |
| GSE13213.Surv_AllMethods_Freq_5 | trial_91 | 0.553255 | 0.541262 | 0.58538 | 0.542833 | 0.600228 | 0.624786 |
| GSE13213.Surv_AllMethods_Freq_6 | trial_91 | 0.590343 | 0.583333 | 0.515057 | 0.600208 | 0.551402 | 0.562305 |
| GSE13213.Surv_AllMethods_Freq_2 | trial_92 | 0.658537 | 0.621616 | 0.674953 | 0.617462 | 0.658068 | 0.669727 |
| GSE13213.Surv_AllMethods_Freq_3 | trial_92 | 0.671175 | 0.637978 | 0.687022 | 0.624932 | 0.665096 | 0.677801 |
| GSE13213.Surv_AllMethods_Freq_4 | trial_92 | 0.650864 | 0.620725 | 0.663212 | 0.597064 | 0.631261 | 0.681693 |
| GSE13213.Surv_AllMethods_Freq_5 | trial_92 | 0.553255 | 0.541262 | 0.58538 | 0.542833 | 0.600228 | 0.624786 |
| GSE13213.Surv_AllMethods_Freq_6 | trial_92 | 0.595275 | 0.583333 | 0.515057 | 0.600208 | 0.551402 | 0.562305 |
| GSE13213.Surv_AllMethods_Freq_2 | trial_93 | 0.658336 | 0.621616 | 0.674953 | 0.617462 | 0.658068 | 0.669727 |
| GSE13213.Surv_AllMethods_Freq_3 | trial_93 | 0.670628 | 0.637978 | 0.687022 | 0.624932 | 0.665096 | 0.677801 |
| GSE13213.Surv_AllMethods_Freq_4 | trial_93 | 0.651554 | 0.620725 | 0.663212 | 0.597064 | 0.631261 | 0.681693 |
| GSE13213.Surv_AllMethods_Freq_5 | trial_93 | 0.551256 | 0.541262 | 0.58538 | 0.542833 | 0.600228 | 0.624786 |
| GSE13213.Surv_AllMethods_Freq_6 | trial_93 | 0.595794 | 0.583333 | 0.515057 | 0.600208 | 0.551402 | 0.562305 |
| GSE13213.Surv_AllMethods_Freq_2 | trial_94 | 0.657196 | 0.621616 | 0.674953 | 0.617462 | 0.658068 | 0.669727 |
| GSE13213.Surv_AllMethods_Freq_3 | trial_94 | 0.669399 | 0.637978 | 0.687022 | 0.624932 | 0.665096 | 0.677801 |
| GSE13213.Surv_AllMethods_Freq_4 | trial_94 | 0.652073 | 0.620725 | 0.663212 | 0.597064 | 0.631261 | 0.681693 |
| GSE13213.Surv_AllMethods_Freq_5 | trial_94 | 0.549686 | 0.541262 | 0.58538 | 0.542833 | 0.600228 | 0.624786 |
| GSE13213.Surv_AllMethods_Freq_6 | trial_94 | 0.59891 | 0.583333 | 0.515057 | 0.600208 | 0.551402 | 0.562305 |
| GSE13213.Surv_AllMethods_Freq_2 | trial_95 | 0.658068 | 0.621616 | 0.674953 | 0.617462 | 0.658068 | 0.669727 |
| GSE13213.Surv_AllMethods_Freq_3 | trial_95 | 0.670423 | 0.637978 | 0.687022 | 0.624932 | 0.665096 | 0.677801 |
| GSE13213.Surv_AllMethods_Freq_4 | trial_95 | 0.651727 | 0.620725 | 0.663212 | 0.597064 | 0.631261 | 0.681693 |
| GSE13213.Surv_AllMethods_Freq_5 | trial_95 | 0.550685 | 0.541262 | 0.58538 | 0.542833 | 0.600228 | 0.624786 |
| GSE13213.Surv_AllMethods_Freq_6 | trial_95 | 0.59865 | 0.583333 | 0.515057 | 0.600208 | 0.551402 | 0.562305 |
| GSE13213.Surv_AllMethods_Freq_2 | trial_96 | 0.660882 | 0.621616 | 0.674953 | 0.617462 | 0.658068 | 0.669727 |
| GSE13213.Surv_AllMethods_Freq_3 | trial_96 | 0.673019 | 0.637978 | 0.687022 | 0.624932 | 0.665096 | 0.677801 |
| GSE13213.Surv_AllMethods_Freq_4 | trial_96 | 0.653886 | 0.620725 | 0.663212 | 0.597064 | 0.631261 | 0.681693 |
| GSE13213.Surv_AllMethods_Freq_5 | trial_96 | 0.553826 | 0.541262 | 0.58538 | 0.542833 | 0.600228 | 0.624786 |
| GSE13213.Surv_AllMethods_Freq_6 | trial_96 | 0.600467 | 0.583333 | 0.515057 | 0.600208 | 0.551402 | 0.562305 |
| GSE13213.Surv_AllMethods_Freq_2 | trial_97 | 0.656526 | 0.621616 | 0.674953 | 0.617462 | 0.658068 | 0.669727 |
| GSE13213.Surv_AllMethods_Freq_3 | trial_97 | 0.668306 | 0.637978 | 0.687022 | 0.624932 | 0.665096 | 0.677801 |
| GSE13213.Surv_AllMethods_Freq_4 | trial_97 | 0.648791 | 0.620725 | 0.663212 | 0.597064 | 0.631261 | 0.681693 |
| GSE13213.Surv_AllMethods_Freq_5 | trial_97 | 0.549686 | 0.541262 | 0.58538 | 0.542833 | 0.600228 | 0.624786 |
| GSE13213.Surv_AllMethods_Freq_6 | trial_97 | 0.601246 | 0.583333 | 0.515057 | 0.600208 | 0.551402 | 0.562305 |
| GSE13213.Surv_AllMethods_Freq_2 | trial_98 | 0.657062 | 0.621616 | 0.674953 | 0.617462 | 0.658068 | 0.669727 |
| GSE13213.Surv_AllMethods_Freq_3 | trial_98 | 0.669126 | 0.637978 | 0.687022 | 0.624932 | 0.665096 | 0.677801 |
| GSE13213.Surv_AllMethods_Freq_4 | trial_98 | 0.647323 | 0.620725 | 0.663212 | 0.597064 | 0.631261 | 0.681693 |
| GSE13213.Surv_AllMethods_Freq_5 | trial_98 | 0.553113 | 0.541262 | 0.58538 | 0.542833 | 0.600228 | 0.624786 |
| GSE13213.Surv_AllMethods_Freq_6 | trial_98 | 0.597871 | 0.583333 | 0.515057 | 0.600208 | 0.551402 | 0.562305 |
| GSE13213.Surv_AllMethods_Freq_2 | trial_99 | 0.655655 | 0.621616 | 0.674953 | 0.617462 | 0.658068 | 0.669727 |
| GSE13213.Surv_AllMethods_Freq_3 | trial_99 | 0.667964 | 0.637978 | 0.687022 | 0.624932 | 0.665096 | 0.677801 |
| GSE13213.Surv_AllMethods_Freq_4 | trial_99 | 0.649309 | 0.620725 | 0.663212 | 0.597064 | 0.631261 | 0.681693 |
| GSE13213.Surv_AllMethods_Freq_5 | trial_99 | 0.547259 | 0.541262 | 0.58538 | 0.542833 | 0.600228 | 0.624786 |
| GSE13213.Surv_AllMethods_Freq_6 | trial_99 | 0.599688 | 0.583333 | 0.515057 | 0.600208 | 0.551402 | 0.562305 |
| GSE13213.Surv_AllMethods_Freq_2 | trial_100 | 0.654784 | 0.621616 | 0.674953 | 0.617462 | 0.658068 | 0.669727 |
| GSE13213.Surv_AllMethods_Freq_3 | trial_100 | 0.666598 | 0.637978 | 0.687022 | 0.624932 | 0.665096 | 0.677801 |
| GSE13213.Surv_AllMethods_Freq_4 | trial_100 | 0.645855 | 0.620725 | 0.663212 | 0.597064 | 0.631261 | 0.681693 |
| GSE13213.Surv_AllMethods_Freq_5 | trial_100 | 0.545545 | 0.541262 | 0.58538 | 0.542833 | 0.600228 | 0.624786 |
| GSE13213.Surv_AllMethods_Freq_6 | trial_100 | 0.602804 | 0.583333 | 0.515057 | 0.600208 | 0.551402 | 0.562305 |
| GSE31210.Surv_AllMethods_Freq_2 | trial_1 | 0.612463 | 0.608277 | 0.577361 | 0.547225 | 0.613729 | 0.576144 |
| GSE31210.Surv_AllMethods_Freq_3 | trial_1 | 0.602742 | 0.599744 | 0.568151 | 0.537441 | 0.607754 | 0.567364 |
| GSE31210.Surv_AllMethods_Freq_4 | trial_1 | 0.627429 | 0.624033 | 0.582863 | 0.558673 | 0.615517 | 0.581818 |
| GSE31210.Surv_AllMethods_Freq_5 | trial_1 | 0.633146 | 0.629713 | 0.581273 | 0.559551 | 0.629213 | 0.602434 |
| GSE31210.Surv_AllMethods_Freq_6 | trial_1 | 0.687363 | 0.674908 | 0.632692 | 0.638645 | 0.675092 | 0.650916 |
| GSE31210.Surv_AllMethods_Freq_2 | trial_2 | 0.609007 | 0.608277 | 0.577361 | 0.547225 | 0.613729 | 0.576144 |
| GSE31210.Surv_AllMethods_Freq_3 | trial_2 | 0.599695 | 0.599744 | 0.568151 | 0.537441 | 0.607754 | 0.567364 |
| GSE31210.Surv_AllMethods_Freq_4 | trial_2 | 0.624504 | 0.624033 | 0.582863 | 0.558673 | 0.615517 | 0.581818 |
| GSE31210.Surv_AllMethods_Freq_5 | trial_2 | 0.631398 | 0.629713 | 0.581273 | 0.559551 | 0.629213 | 0.602434 |
| GSE31210.Surv_AllMethods_Freq_6 | trial_2 | 0.683516 | 0.674908 | 0.632692 | 0.638645 | 0.675092 | 0.650916 |
| GSE31210.Surv_AllMethods_Freq_2 | trial_3 | 0.611441 | 0.608277 | 0.577361 | 0.547225 | 0.613729 | 0.576144 |
| GSE31210.Surv_AllMethods_Freq_3 | trial_3 | 0.602054 | 0.599744 | 0.568151 | 0.537441 | 0.607754 | 0.567364 |
| GSE31210.Surv_AllMethods_Freq_4 | trial_3 | 0.626541 | 0.624033 | 0.582863 | 0.558673 | 0.615517 | 0.581818 |
| GSE31210.Surv_AllMethods_Freq_5 | trial_3 | 0.631086 | 0.629713 | 0.581273 | 0.559551 | 0.629213 | 0.602434 |
| GSE31210.Surv_AllMethods_Freq_6 | trial_3 | 0.687179 | 0.674908 | 0.632692 | 0.638645 | 0.675092 | 0.650916 |
| GSE31210.Surv_AllMethods_Freq_2 | trial_4 | 0.610078 | 0.608277 | 0.577361 | 0.547225 | 0.613729 | 0.576144 |
| GSE31210.Surv_AllMethods_Freq_3 | trial_4 | 0.600285 | 0.599744 | 0.568151 | 0.537441 | 0.607754 | 0.567364 |
| GSE31210.Surv_AllMethods_Freq_4 | trial_4 | 0.624608 | 0.624033 | 0.582863 | 0.558673 | 0.615517 | 0.581818 |
| GSE31210.Surv_AllMethods_Freq_5 | trial_4 | 0.630524 | 0.629713 | 0.581273 | 0.559551 | 0.629213 | 0.602434 |
| GSE31210.Surv_AllMethods_Freq_6 | trial_4 | 0.6837 | 0.674908 | 0.632692 | 0.638645 | 0.675092 | 0.650916 |
| GSE31210.Surv_AllMethods_Freq_2 | trial_5 | 0.61222 | 0.608277 | 0.577361 | 0.547225 | 0.613729 | 0.576144 |
| GSE31210.Surv_AllMethods_Freq_3 | trial_5 | 0.602545 | 0.599744 | 0.568151 | 0.537441 | 0.607754 | 0.567364 |
| GSE31210.Surv_AllMethods_Freq_4 | trial_5 | 0.627273 | 0.624033 | 0.582863 | 0.558673 | 0.615517 | 0.581818 |
| GSE31210.Surv_AllMethods_Freq_5 | trial_5 | 0.629463 | 0.629713 | 0.581273 | 0.559551 | 0.629213 | 0.602434 |
| GSE31210.Surv_AllMethods_Freq_6 | trial_5 | 0.6837 | 0.674908 | 0.632692 | 0.638645 | 0.675092 | 0.650916 |
| GSE31210.Surv_AllMethods_Freq_2 | trial_6 | 0.609348 | 0.608277 | 0.577361 | 0.547225 | 0.613729 | 0.576144 |
| GSE31210.Surv_AllMethods_Freq_3 | trial_6 | 0.600236 | 0.599744 | 0.568151 | 0.537441 | 0.607754 | 0.567364 |
| GSE31210.Surv_AllMethods_Freq_4 | trial_6 | 0.625026 | 0.624033 | 0.582863 | 0.558673 | 0.615517 | 0.581818 |
| GSE31210.Surv_AllMethods_Freq_5 | trial_6 | 0.6299 | 0.629713 | 0.581273 | 0.559551 | 0.629213 | 0.602434 |
| GSE31210.Surv_AllMethods_Freq_6 | trial_6 | 0.682692 | 0.674908 | 0.632692 | 0.638645 | 0.675092 | 0.650916 |
| GSE31210.Surv_AllMethods_Freq_2 | trial_7 | 0.611441 | 0.608277 | 0.577361 | 0.547225 | 0.613729 | 0.576144 |
| GSE31210.Surv_AllMethods_Freq_3 | trial_7 | 0.601661 | 0.599744 | 0.568151 | 0.537441 | 0.607754 | 0.567364 |
| GSE31210.Surv_AllMethods_Freq_4 | trial_7 | 0.626855 | 0.624033 | 0.582863 | 0.558673 | 0.615517 | 0.581818 |
| GSE31210.Surv_AllMethods_Freq_5 | trial_7 | 0.632272 | 0.629713 | 0.581273 | 0.559551 | 0.629213 | 0.602434 |
| GSE31210.Surv_AllMethods_Freq_6 | trial_7 | 0.687546 | 0.674908 | 0.632692 | 0.638645 | 0.675092 | 0.650916 |
| GSE31210.Surv_AllMethods_Freq_2 | trial_8 | 0.612804 | 0.608277 | 0.577361 | 0.547225 | 0.613729 | 0.576144 |
| GSE31210.Surv_AllMethods_Freq_3 | trial_8 | 0.603086 | 0.599744 | 0.568151 | 0.537441 | 0.607754 | 0.567364 |
| GSE31210.Surv_AllMethods_Freq_4 | trial_8 | 0.628474 | 0.624033 | 0.582863 | 0.558673 | 0.615517 | 0.581818 |
| GSE31210.Surv_AllMethods_Freq_5 | trial_8 | 0.633895 | 0.629713 | 0.581273 | 0.559551 | 0.629213 | 0.602434 |
| GSE31210.Surv_AllMethods_Freq_6 | trial_8 | 0.690293 | 0.674908 | 0.632692 | 0.638645 | 0.675092 | 0.650916 |
| GSE31210.Surv_AllMethods_Freq_2 | trial_9 | 0.610906 | 0.608277 | 0.577361 | 0.547225 | 0.613729 | 0.576144 |
| GSE31210.Surv_AllMethods_Freq_3 | trial_9 | 0.601808 | 0.599744 | 0.568151 | 0.537441 | 0.607754 | 0.567364 |
| GSE31210.Surv_AllMethods_Freq_4 | trial_9 | 0.62675 | 0.624033 | 0.582863 | 0.558673 | 0.615517 | 0.581818 |
| GSE31210.Surv_AllMethods_Freq_5 | trial_9 | 0.63171 | 0.629713 | 0.581273 | 0.559551 | 0.629213 | 0.602434 |
| GSE31210.Surv_AllMethods_Freq_6 | trial_9 | 0.685348 | 0.674908 | 0.632692 | 0.638645 | 0.675092 | 0.650916 |
| GSE31210.Surv_AllMethods_Freq_2 | trial_10 | 0.609883 | 0.608277 | 0.577361 | 0.547225 | 0.613729 | 0.576144 |
| GSE31210.Surv_AllMethods_Freq_3 | trial_10 | 0.600138 | 0.599744 | 0.568151 | 0.537441 | 0.607754 | 0.567364 |
| GSE31210.Surv_AllMethods_Freq_4 | trial_10 | 0.625758 | 0.624033 | 0.582863 | 0.558673 | 0.615517 | 0.581818 |
| GSE31210.Surv_AllMethods_Freq_5 | trial_10 | 0.632522 | 0.629713 | 0.581273 | 0.559551 | 0.629213 | 0.602434 |
| GSE31210.Surv_AllMethods_Freq_6 | trial_10 | 0.68663 | 0.674908 | 0.632692 | 0.638645 | 0.675092 | 0.650916 |
| GSE31210.Surv_AllMethods_Freq_2 | trial_11 | 0.613096 | 0.608277 | 0.577361 | 0.547225 | 0.613729 | 0.576144 |
| GSE31210.Surv_AllMethods_Freq_3 | trial_11 | 0.603921 | 0.599744 | 0.568151 | 0.537441 | 0.607754 | 0.567364 |
| GSE31210.Surv_AllMethods_Freq_4 | trial_11 | 0.628004 | 0.624033 | 0.582863 | 0.558673 | 0.615517 | 0.581818 |
| GSE31210.Surv_AllMethods_Freq_5 | trial_11 | 0.635331 | 0.629713 | 0.581273 | 0.559551 | 0.629213 | 0.602434 |
| GSE31210.Surv_AllMethods_Freq_6 | trial_11 | 0.690018 | 0.674908 | 0.632692 | 0.638645 | 0.675092 | 0.650916 |
| GSE31210.Surv_AllMethods_Freq_2 | trial_12 | 0.610419 | 0.608277 | 0.577361 | 0.547225 | 0.613729 | 0.576144 |
| GSE31210.Surv_AllMethods_Freq_3 | trial_12 | 0.600875 | 0.599744 | 0.568151 | 0.537441 | 0.607754 | 0.567364 |
| GSE31210.Surv_AllMethods_Freq_4 | trial_12 | 0.625862 | 0.624033 | 0.582863 | 0.558673 | 0.615517 | 0.581818 |
| GSE31210.Surv_AllMethods_Freq_5 | trial_12 | 0.631835 | 0.629713 | 0.581273 | 0.559551 | 0.629213 | 0.602434 |
| GSE31210.Surv_AllMethods_Freq_6 | trial_12 | 0.686355 | 0.674908 | 0.632692 | 0.638645 | 0.675092 | 0.650916 |
| GSE31210.Surv_AllMethods_Freq_2 | trial_13 | 0.611149 | 0.608277 | 0.577361 | 0.547225 | 0.613729 | 0.576144 |
| GSE31210.Surv_AllMethods_Freq_3 | trial_13 | 0.602447 | 0.599744 | 0.568151 | 0.537441 | 0.607754 | 0.567364 |
| GSE31210.Surv_AllMethods_Freq_4 | trial_13 | 0.626385 | 0.624033 | 0.582863 | 0.558673 | 0.615517 | 0.581818 |
| GSE31210.Surv_AllMethods_Freq_5 | trial_13 | 0.6304 | 0.629713 | 0.581273 | 0.559551 | 0.629213 | 0.602434 |
| GSE31210.Surv_AllMethods_Freq_6 | trial_13 | 0.684249 | 0.674908 | 0.632692 | 0.638645 | 0.675092 | 0.650916 |
| GSE31210.Surv_AllMethods_Freq_2 | trial_14 | 0.610224 | 0.608277 | 0.577361 | 0.547225 | 0.613729 | 0.576144 |
| GSE31210.Surv_AllMethods_Freq_3 | trial_14 | 0.601366 | 0.599744 | 0.568151 | 0.537441 | 0.607754 | 0.567364 |
| GSE31210.Surv_AllMethods_Freq_4 | trial_14 | 0.62628 | 0.624033 | 0.582863 | 0.558673 | 0.615517 | 0.581818 |
| GSE31210.Surv_AllMethods_Freq_5 | trial_14 | 0.630774 | 0.629713 | 0.581273 | 0.559551 | 0.629213 | 0.602434 |
| GSE31210.Surv_AllMethods_Freq_6 | trial_14 | 0.685256 | 0.674908 | 0.632692 | 0.638645 | 0.675092 | 0.650916 |
| GSE31210.Surv_AllMethods_Freq_2 | trial_15 | 0.610273 | 0.608277 | 0.577361 | 0.547225 | 0.613729 | 0.576144 |
| GSE31210.Surv_AllMethods_Freq_3 | trial_15 | 0.601513 | 0.599744 | 0.568151 | 0.537441 | 0.607754 | 0.567364 |
| GSE31210.Surv_AllMethods_Freq_4 | trial_15 | 0.625601 | 0.624033 | 0.582863 | 0.558673 | 0.615517 | 0.581818 |
| GSE31210.Surv_AllMethods_Freq_5 | trial_15 | 0.633208 | 0.629713 | 0.581273 | 0.559551 | 0.629213 | 0.602434 |
| GSE31210.Surv_AllMethods_Freq_6 | trial_15 | 0.687179 | 0.674908 | 0.632692 | 0.638645 | 0.675092 | 0.650916 |
| GSE31210.Surv_AllMethods_Freq_2 | trial_16 | 0.608958 | 0.608277 | 0.577361 | 0.547225 | 0.613729 | 0.576144 |
| GSE31210.Surv_AllMethods_Freq_3 | trial_16 | 0.599646 | 0.599744 | 0.568151 | 0.537441 | 0.607754 | 0.567364 |
| GSE31210.Surv_AllMethods_Freq_4 | trial_16 | 0.625287 | 0.624033 | 0.582863 | 0.558673 | 0.615517 | 0.581818 |
| GSE31210.Surv_AllMethods_Freq_5 | trial_16 | 0.631586 | 0.629713 | 0.581273 | 0.559551 | 0.629213 | 0.602434 |
| GSE31210.Surv_AllMethods_Freq_6 | trial_16 | 0.686722 | 0.674908 | 0.632692 | 0.638645 | 0.675092 | 0.650916 |
| GSE31210.Surv_AllMethods_Freq_2 | trial_17 | 0.612756 | 0.608277 | 0.577361 | 0.547225 | 0.613729 | 0.576144 |
| GSE31210.Surv_AllMethods_Freq_3 | trial_17 | 0.603331 | 0.599744 | 0.568151 | 0.537441 | 0.607754 | 0.567364 |
| GSE31210.Surv_AllMethods_Freq_4 | trial_17 | 0.627325 | 0.624033 | 0.582863 | 0.558673 | 0.615517 | 0.581818 |
| GSE31210.Surv_AllMethods_Freq_5 | trial_17 | 0.633271 | 0.629713 | 0.581273 | 0.559551 | 0.629213 | 0.602434 |
| GSE31210.Surv_AllMethods_Freq_6 | trial_17 | 0.688462 | 0.674908 | 0.632692 | 0.638645 | 0.675092 | 0.650916 |
| GSE31210.Surv_AllMethods_Freq_2 | trial_18 | 0.610759 | 0.608277 | 0.577361 | 0.547225 | 0.613729 | 0.576144 |
| GSE31210.Surv_AllMethods_Freq_3 | trial_18 | 0.60171 | 0.599744 | 0.568151 | 0.537441 | 0.607754 | 0.567364 |
| GSE31210.Surv_AllMethods_Freq_4 | trial_18 | 0.625287 | 0.624033 | 0.582863 | 0.558673 | 0.615517 | 0.581818 |
| GSE31210.Surv_AllMethods_Freq_5 | trial_18 | 0.632834 | 0.629713 | 0.581273 | 0.559551 | 0.629213 | 0.602434 |
| GSE31210.Surv_AllMethods_Freq_6 | trial_18 | 0.687729 | 0.674908 | 0.632692 | 0.638645 | 0.675092 | 0.650916 |
| GSE31210.Surv_AllMethods_Freq_2 | trial_19 | 0.609883 | 0.608277 | 0.577361 | 0.547225 | 0.613729 | 0.576144 |
| GSE31210.Surv_AllMethods_Freq_3 | trial_19 | 0.600924 | 0.599744 | 0.568151 | 0.537441 | 0.607754 | 0.567364 |
| GSE31210.Surv_AllMethods_Freq_4 | trial_19 | 0.625287 | 0.624033 | 0.582863 | 0.558673 | 0.615517 | 0.581818 |
| GSE31210.Surv_AllMethods_Freq_5 | trial_19 | 0.631461 | 0.629713 | 0.581273 | 0.559551 | 0.629213 | 0.602434 |
| GSE31210.Surv_AllMethods_Freq_6 | trial_19 | 0.68663 | 0.674908 | 0.632692 | 0.638645 | 0.675092 | 0.650916 |
| GSE31210.Surv_AllMethods_Freq_2 | trial_20 | 0.612463 | 0.608277 | 0.577361 | 0.547225 | 0.613729 | 0.576144 |
| GSE31210.Surv_AllMethods_Freq_3 | trial_20 | 0.603184 | 0.599744 | 0.568151 | 0.537441 | 0.607754 | 0.567364 |
| GSE31210.Surv_AllMethods_Freq_4 | trial_20 | 0.627116 | 0.624033 | 0.582863 | 0.558673 | 0.615517 | 0.581818 |
| GSE31210.Surv_AllMethods_Freq_5 | trial_20 | 0.632397 | 0.629713 | 0.581273 | 0.559551 | 0.629213 | 0.602434 |
| GSE31210.Surv_AllMethods_Freq_6 | trial_20 | 0.685531 | 0.674908 | 0.632692 | 0.638645 | 0.675092 | 0.650916 |
| GSE31210.Surv_AllMethods_Freq_2 | trial_21 | 0.60813 | 0.608277 | 0.577361 | 0.547225 | 0.613729 | 0.576144 |
| GSE31210.Surv_AllMethods_Freq_3 | trial_21 | 0.598811 | 0.599744 | 0.568151 | 0.537441 | 0.607754 | 0.567364 |
| GSE31210.Surv_AllMethods_Freq_4 | trial_21 | 0.623981 | 0.624033 | 0.582863 | 0.558673 | 0.615517 | 0.581818 |
| GSE31210.Surv_AllMethods_Freq_5 | trial_21 | 0.630462 | 0.629713 | 0.581273 | 0.559551 | 0.629213 | 0.602434 |
| GSE31210.Surv_AllMethods_Freq_6 | trial_21 | 0.684982 | 0.674908 | 0.632692 | 0.638645 | 0.675092 | 0.650916 |
| GSE31210.Surv_AllMethods_Freq_2 | trial_22 | 0.610419 | 0.608277 | 0.577361 | 0.547225 | 0.613729 | 0.576144 |
| GSE31210.Surv_AllMethods_Freq_3 | trial_22 | 0.601219 | 0.599744 | 0.568151 | 0.537441 | 0.607754 | 0.567364 |
| GSE31210.Surv_AllMethods_Freq_4 | trial_22 | 0.62675 | 0.624033 | 0.582863 | 0.558673 | 0.615517 | 0.581818 |
| GSE31210.Surv_AllMethods_Freq_5 | trial_22 | 0.63427 | 0.629713 | 0.581273 | 0.559551 | 0.629213 | 0.602434 |
| GSE31210.Surv_AllMethods_Freq_6 | trial_22 | 0.68837 | 0.674908 | 0.632692 | 0.638645 | 0.675092 | 0.650916 |
| GSE31210.Surv_AllMethods_Freq_2 | trial_23 | 0.609494 | 0.608277 | 0.577361 | 0.547225 | 0.613729 | 0.576144 |
| GSE31210.Surv_AllMethods_Freq_3 | trial_23 | 0.600727 | 0.599744 | 0.568151 | 0.537441 | 0.607754 | 0.567364 |
| GSE31210.Surv_AllMethods_Freq_4 | trial_23 | 0.626019 | 0.624033 | 0.582863 | 0.558673 | 0.615517 | 0.581818 |
| GSE31210.Surv_AllMethods_Freq_5 | trial_23 | 0.629026 | 0.629713 | 0.581273 | 0.559551 | 0.629213 | 0.602434 |
| GSE31210.Surv_AllMethods_Freq_6 | trial_23 | 0.683425 | 0.674908 | 0.632692 | 0.638645 | 0.675092 | 0.650916 |
| GSE31210.Surv_AllMethods_Freq_2 | trial_24 | 0.609396 | 0.608277 | 0.577361 | 0.547225 | 0.613729 | 0.576144 |
| GSE31210.Surv_AllMethods_Freq_3 | trial_24 | 0.600039 | 0.599744 | 0.568151 | 0.537441 | 0.607754 | 0.567364 |
| GSE31210.Surv_AllMethods_Freq_4 | trial_24 | 0.624765 | 0.624033 | 0.582863 | 0.558673 | 0.615517 | 0.581818 |
| GSE31210.Surv_AllMethods_Freq_5 | trial_24 | 0.629401 | 0.629713 | 0.581273 | 0.559551 | 0.629213 | 0.602434 |
| GSE31210.Surv_AllMethods_Freq_6 | trial_24 | 0.682967 | 0.674908 | 0.632692 | 0.638645 | 0.675092 | 0.650916 |
| GSE31210.Surv_AllMethods_Freq_2 | trial_25 | 0.610857 | 0.608277 | 0.577361 | 0.547225 | 0.613729 | 0.576144 |
| GSE31210.Surv_AllMethods_Freq_3 | trial_25 | 0.602152 | 0.599744 | 0.568151 | 0.537441 | 0.607754 | 0.567364 |
| GSE31210.Surv_AllMethods_Freq_4 | trial_25 | 0.627638 | 0.624033 | 0.582863 | 0.558673 | 0.615517 | 0.581818 |
| GSE31210.Surv_AllMethods_Freq_5 | trial_25 | 0.632147 | 0.629713 | 0.581273 | 0.559551 | 0.629213 | 0.602434 |
| GSE31210.Surv_AllMethods_Freq_6 | trial_25 | 0.688553 | 0.674908 | 0.632692 | 0.638645 | 0.675092 | 0.650916 |
| GSE31210.Surv_AllMethods_Freq_2 | trial_26 | 0.614021 | 0.608277 | 0.577361 | 0.547225 | 0.613729 | 0.576144 |
| GSE31210.Surv_AllMethods_Freq_3 | trial_26 | 0.604904 | 0.599744 | 0.568151 | 0.537441 | 0.607754 | 0.567364 |
| GSE31210.Surv_AllMethods_Freq_4 | trial_26 | 0.629833 | 0.624033 | 0.582863 | 0.558673 | 0.615517 | 0.581818 |
| GSE31210.Surv_AllMethods_Freq_5 | trial_26 | 0.63608 | 0.629713 | 0.581273 | 0.559551 | 0.629213 | 0.602434 |
| GSE31210.Surv_AllMethods_Freq_6 | trial_26 | 0.688462 | 0.674908 | 0.632692 | 0.638645 | 0.675092 | 0.650916 |
| GSE31210.Surv_AllMethods_Freq_2 | trial_27 | 0.608763 | 0.608277 | 0.577361 | 0.547225 | 0.613729 | 0.576144 |
| GSE31210.Surv_AllMethods_Freq_3 | trial_27 | 0.599499 | 0.599744 | 0.568151 | 0.537441 | 0.607754 | 0.567364 |
| GSE31210.Surv_AllMethods_Freq_4 | trial_27 | 0.623824 | 0.624033 | 0.582863 | 0.558673 | 0.615517 | 0.581818 |
| GSE31210.Surv_AllMethods_Freq_5 | trial_27 | 0.628652 | 0.629713 | 0.581273 | 0.559551 | 0.629213 | 0.602434 |
| GSE31210.Surv_AllMethods_Freq_6 | trial_27 | 0.683516 | 0.674908 | 0.632692 | 0.638645 | 0.675092 | 0.650916 |
| GSE31210.Surv_AllMethods_Freq_2 | trial_28 | 0.607546 | 0.608277 | 0.577361 | 0.547225 | 0.613729 | 0.576144 |
| GSE31210.Surv_AllMethods_Freq_3 | trial_28 | 0.598467 | 0.599744 | 0.568151 | 0.537441 | 0.607754 | 0.567364 |
| GSE31210.Surv_AllMethods_Freq_4 | trial_28 | 0.623302 | 0.624033 | 0.582863 | 0.558673 | 0.615517 | 0.581818 |
| GSE31210.Surv_AllMethods_Freq_5 | trial_28 | 0.631835 | 0.629713 | 0.581273 | 0.559551 | 0.629213 | 0.602434 |
| GSE31210.Surv_AllMethods_Freq_6 | trial_28 | 0.684707 | 0.674908 | 0.632692 | 0.638645 | 0.675092 | 0.650916 |
| GSE31210.Surv_AllMethods_Freq_2 | trial_29 | 0.612463 | 0.608277 | 0.577361 | 0.547225 | 0.613729 | 0.576144 |
| GSE31210.Surv_AllMethods_Freq_3 | trial_29 | 0.603184 | 0.599744 | 0.568151 | 0.537441 | 0.607754 | 0.567364 |
| GSE31210.Surv_AllMethods_Freq_4 | trial_29 | 0.627586 | 0.624033 | 0.582863 | 0.558673 | 0.615517 | 0.581818 |
| GSE31210.Surv_AllMethods_Freq_5 | trial_29 | 0.632459 | 0.629713 | 0.581273 | 0.559551 | 0.629213 | 0.602434 |
| GSE31210.Surv_AllMethods_Freq_6 | trial_29 | 0.684341 | 0.674908 | 0.632692 | 0.638645 | 0.675092 | 0.650916 |
| GSE31210.Surv_AllMethods_Freq_2 | trial_30 | 0.609153 | 0.608277 | 0.577361 | 0.547225 | 0.613729 | 0.576144 |
| GSE31210.Surv_AllMethods_Freq_3 | trial_30 | 0.599941 | 0.599744 | 0.568151 | 0.537441 | 0.607754 | 0.567364 |
| GSE31210.Surv_AllMethods_Freq_4 | trial_30 | 0.624086 | 0.624033 | 0.582863 | 0.558673 | 0.615517 | 0.581818 |
| GSE31210.Surv_AllMethods_Freq_5 | trial_30 | 0.629151 | 0.629713 | 0.581273 | 0.559551 | 0.629213 | 0.602434 |
| GSE31210.Surv_AllMethods_Freq_6 | trial_30 | 0.681227 | 0.674908 | 0.632692 | 0.638645 | 0.675092 | 0.650916 |
| GSE31210.Surv_AllMethods_Freq_2 | trial_31 | 0.610224 | 0.608277 | 0.577361 | 0.547225 | 0.613729 | 0.576144 |
| GSE31210.Surv_AllMethods_Freq_3 | trial_31 | 0.600678 | 0.599744 | 0.568151 | 0.537441 | 0.607754 | 0.567364 |
| GSE31210.Surv_AllMethods_Freq_4 | trial_31 | 0.623981 | 0.624033 | 0.582863 | 0.558673 | 0.615517 | 0.581818 |
| GSE31210.Surv_AllMethods_Freq_5 | trial_31 | 0.630212 | 0.629713 | 0.581273 | 0.559551 | 0.629213 | 0.602434 |
| GSE31210.Surv_AllMethods_Freq_6 | trial_31 | 0.682784 | 0.674908 | 0.632692 | 0.638645 | 0.675092 | 0.650916 |
| GSE31210.Surv_AllMethods_Freq_2 | trial_32 | 0.609396 | 0.608277 | 0.577361 | 0.547225 | 0.613729 | 0.576144 |
| GSE31210.Surv_AllMethods_Freq_3 | trial_32 | 0.600531 | 0.599744 | 0.568151 | 0.537441 | 0.607754 | 0.567364 |
| GSE31210.Surv_AllMethods_Freq_4 | trial_32 | 0.625914 | 0.624033 | 0.582863 | 0.558673 | 0.615517 | 0.581818 |
| GSE31210.Surv_AllMethods_Freq_5 | trial_32 | 0.631898 | 0.629713 | 0.581273 | 0.559551 | 0.629213 | 0.602434 |
| GSE31210.Surv_AllMethods_Freq_6 | trial_32 | 0.685806 | 0.674908 | 0.632692 | 0.638645 | 0.675092 | 0.650916 |
| GSE31210.Surv_AllMethods_Freq_2 | trial_33 | 0.610808 | 0.608277 | 0.577361 | 0.547225 | 0.613729 | 0.576144 |
| GSE31210.Surv_AllMethods_Freq_3 | trial_33 | 0.601366 | 0.599744 | 0.568151 | 0.537441 | 0.607754 | 0.567364 |
| GSE31210.Surv_AllMethods_Freq_4 | trial_33 | 0.625444 | 0.624033 | 0.582863 | 0.558673 | 0.615517 | 0.581818 |
| GSE31210.Surv_AllMethods_Freq_5 | trial_33 | 0.629588 | 0.629713 | 0.581273 | 0.559551 | 0.629213 | 0.602434 |
| GSE31210.Surv_AllMethods_Freq_6 | trial_33 | 0.682601 | 0.674908 | 0.632692 | 0.638645 | 0.675092 | 0.650916 |
| GSE31210.Surv_AllMethods_Freq_2 | trial_34 | 0.609396 | 0.608277 | 0.577361 | 0.547225 | 0.613729 | 0.576144 |
| GSE31210.Surv_AllMethods_Freq_3 | trial_34 | 0.600187 | 0.599744 | 0.568151 | 0.537441 | 0.607754 | 0.567364 |
| GSE31210.Surv_AllMethods_Freq_4 | trial_34 | 0.624974 | 0.624033 | 0.582863 | 0.558673 | 0.615517 | 0.581818 |
| GSE31210.Surv_AllMethods_Freq_5 | trial_34 | 0.631773 | 0.629713 | 0.581273 | 0.559551 | 0.629213 | 0.602434 |
| GSE31210.Surv_AllMethods_Freq_6 | trial_34 | 0.684982 | 0.674908 | 0.632692 | 0.638645 | 0.675092 | 0.650916 |
| GSE31210.Surv_AllMethods_Freq_2 | trial_35 | 0.611685 | 0.608277 | 0.577361 | 0.547225 | 0.613729 | 0.576144 |
| GSE31210.Surv_AllMethods_Freq_3 | trial_35 | 0.60284 | 0.599744 | 0.568151 | 0.537441 | 0.607754 | 0.567364 |
| GSE31210.Surv_AllMethods_Freq_4 | trial_35 | 0.628109 | 0.624033 | 0.582863 | 0.558673 | 0.615517 | 0.581818 |
| GSE31210.Surv_AllMethods_Freq_5 | trial_35 | 0.634956 | 0.629713 | 0.581273 | 0.559551 | 0.629213 | 0.602434 |
| GSE31210.Surv_AllMethods_Freq_6 | trial_35 | 0.688187 | 0.674908 | 0.632692 | 0.638645 | 0.675092 | 0.650916 |
| GSE31210.Surv_AllMethods_Freq_2 | trial_36 | 0.612366 | 0.608277 | 0.577361 | 0.547225 | 0.613729 | 0.576144 |
| GSE31210.Surv_AllMethods_Freq_3 | trial_36 | 0.602791 | 0.599744 | 0.568151 | 0.537441 | 0.607754 | 0.567364 |
| GSE31210.Surv_AllMethods_Freq_4 | trial_36 | 0.627064 | 0.624033 | 0.582863 | 0.558673 | 0.615517 | 0.581818 |
| GSE31210.Surv_AllMethods_Freq_5 | trial_36 | 0.633146 | 0.629713 | 0.581273 | 0.559551 | 0.629213 | 0.602434 |
| GSE31210.Surv_AllMethods_Freq_6 | trial_36 | 0.689103 | 0.674908 | 0.632692 | 0.638645 | 0.675092 | 0.650916 |
| GSE31210.Surv_AllMethods_Freq_2 | trial_37 | 0.60964 | 0.608277 | 0.577361 | 0.547225 | 0.613729 | 0.576144 |
| GSE31210.Surv_AllMethods_Freq_3 | trial_37 | 0.600432 | 0.599744 | 0.568151 | 0.537441 | 0.607754 | 0.567364 |
| GSE31210.Surv_AllMethods_Freq_4 | trial_37 | 0.625078 | 0.624033 | 0.582863 | 0.558673 | 0.615517 | 0.581818 |
| GSE31210.Surv_AllMethods_Freq_5 | trial_37 | 0.631024 | 0.629713 | 0.581273 | 0.559551 | 0.629213 | 0.602434 |
| GSE31210.Surv_AllMethods_Freq_6 | trial_37 | 0.686355 | 0.674908 | 0.632692 | 0.638645 | 0.675092 | 0.650916 |
| GSE31210.Surv_AllMethods_Freq_2 | trial_38 | 0.606865 | 0.608277 | 0.577361 | 0.547225 | 0.613729 | 0.576144 |
| GSE31210.Surv_AllMethods_Freq_3 | trial_38 | 0.598025 | 0.599744 | 0.568151 | 0.537441 | 0.607754 | 0.567364 |
| GSE31210.Surv_AllMethods_Freq_4 | trial_38 | 0.623197 | 0.624033 | 0.582863 | 0.558673 | 0.615517 | 0.581818 |
| GSE31210.Surv_AllMethods_Freq_5 | trial_38 | 0.626654 | 0.629713 | 0.581273 | 0.559551 | 0.629213 | 0.602434 |
| GSE31210.Surv_AllMethods_Freq_6 | trial_38 | 0.68141 | 0.674908 | 0.632692 | 0.638645 | 0.675092 | 0.650916 |
| GSE31210.Surv_AllMethods_Freq_2 | trial_39 | 0.606475 | 0.608277 | 0.577361 | 0.547225 | 0.613729 | 0.576144 |
| GSE31210.Surv_AllMethods_Freq_3 | trial_39 | 0.597288 | 0.599744 | 0.568151 | 0.537441 | 0.607754 | 0.567364 |
| GSE31210.Surv_AllMethods_Freq_4 | trial_39 | 0.62116 | 0.624033 | 0.582863 | 0.558673 | 0.615517 | 0.581818 |
| GSE31210.Surv_AllMethods_Freq_5 | trial_39 | 0.624906 | 0.629713 | 0.581273 | 0.559551 | 0.629213 | 0.602434 |
| GSE31210.Surv_AllMethods_Freq_6 | trial_39 | 0.681502 | 0.674908 | 0.632692 | 0.638645 | 0.675092 | 0.650916 |
| GSE31210.Surv_AllMethods_Freq_2 | trial_40 | 0.61037 | 0.608277 | 0.577361 | 0.547225 | 0.613729 | 0.576144 |
| GSE31210.Surv_AllMethods_Freq_3 | trial_40 | 0.601317 | 0.599744 | 0.568151 | 0.537441 | 0.607754 | 0.567364 |
| GSE31210.Surv_AllMethods_Freq_4 | trial_40 | 0.627011 | 0.624033 | 0.582863 | 0.558673 | 0.615517 | 0.581818 |
| GSE31210.Surv_AllMethods_Freq_5 | trial_40 | 0.633708 | 0.629713 | 0.581273 | 0.559551 | 0.629213 | 0.602434 |
| GSE31210.Surv_AllMethods_Freq_6 | trial_40 | 0.690659 | 0.674908 | 0.632692 | 0.638645 | 0.675092 | 0.650916 |
| GSE31210.Surv_AllMethods_Freq_2 | trial_41 | 0.612756 | 0.608277 | 0.577361 | 0.547225 | 0.613729 | 0.576144 |
| GSE31210.Surv_AllMethods_Freq_3 | trial_41 | 0.603577 | 0.599744 | 0.568151 | 0.537441 | 0.607754 | 0.567364 |
| GSE31210.Surv_AllMethods_Freq_4 | trial_41 | 0.62722 | 0.624033 | 0.582863 | 0.558673 | 0.615517 | 0.581818 |
| GSE31210.Surv_AllMethods_Freq_5 | trial_41 | 0.633208 | 0.629713 | 0.581273 | 0.559551 | 0.629213 | 0.602434 |
| GSE31210.Surv_AllMethods_Freq_6 | trial_41 | 0.686996 | 0.674908 | 0.632692 | 0.638645 | 0.675092 | 0.650916 |
| GSE31210.Surv_AllMethods_Freq_2 | trial_42 | 0.6074 | 0.608277 | 0.577361 | 0.547225 | 0.613729 | 0.576144 |
| GSE31210.Surv_AllMethods_Freq_3 | trial_42 | 0.598958 | 0.599744 | 0.568151 | 0.537441 | 0.607754 | 0.567364 |
| GSE31210.Surv_AllMethods_Freq_4 | trial_42 | 0.623354 | 0.624033 | 0.582863 | 0.558673 | 0.615517 | 0.581818 |
| GSE31210.Surv_AllMethods_Freq_5 | trial_42 | 0.629151 | 0.629713 | 0.581273 | 0.559551 | 0.629213 | 0.602434 |
| GSE31210.Surv_AllMethods_Freq_6 | trial_42 | 0.681136 | 0.674908 | 0.632692 | 0.638645 | 0.675092 | 0.650916 |
| GSE31210.Surv_AllMethods_Freq_2 | trial_43 | 0.607254 | 0.608277 | 0.577361 | 0.547225 | 0.613729 | 0.576144 |
| GSE31210.Surv_AllMethods_Freq_3 | trial_43 | 0.59827 | 0.599744 | 0.568151 | 0.537441 | 0.607754 | 0.567364 |
| GSE31210.Surv_AllMethods_Freq_4 | trial_43 | 0.621317 | 0.624033 | 0.582863 | 0.558673 | 0.615517 | 0.581818 |
| GSE31210.Surv_AllMethods_Freq_5 | trial_43 | 0.627341 | 0.629713 | 0.581273 | 0.559551 | 0.629213 | 0.602434 |
| GSE31210.Surv_AllMethods_Freq_6 | trial_43 | 0.681136 | 0.674908 | 0.632692 | 0.638645 | 0.675092 | 0.650916 |
| GSE31210.Surv_AllMethods_Freq_2 | trial_44 | 0.611733 | 0.608277 | 0.577361 | 0.547225 | 0.613729 | 0.576144 |
| GSE31210.Surv_AllMethods_Freq_3 | trial_44 | 0.602791 | 0.599744 | 0.568151 | 0.537441 | 0.607754 | 0.567364 |
| GSE31210.Surv_AllMethods_Freq_4 | trial_44 | 0.627429 | 0.624033 | 0.582863 | 0.558673 | 0.615517 | 0.581818 |
| GSE31210.Surv_AllMethods_Freq_5 | trial_44 | 0.630649 | 0.629713 | 0.581273 | 0.559551 | 0.629213 | 0.602434 |
| GSE31210.Surv_AllMethods_Freq_6 | trial_44 | 0.684066 | 0.674908 | 0.632692 | 0.638645 | 0.675092 | 0.650916 |
| GSE31210.Surv_AllMethods_Freq_2 | trial_45 | 0.609786 | 0.608277 | 0.577361 | 0.547225 | 0.613729 | 0.576144 |
| GSE31210.Surv_AllMethods_Freq_3 | trial_45 | 0.600727 | 0.599744 | 0.568151 | 0.537441 | 0.607754 | 0.567364 |
| GSE31210.Surv_AllMethods_Freq_4 | trial_45 | 0.625496 | 0.624033 | 0.582863 | 0.558673 | 0.615517 | 0.581818 |
| GSE31210.Surv_AllMethods_Freq_5 | trial_45 | 0.631523 | 0.629713 | 0.581273 | 0.559551 | 0.629213 | 0.602434 |
| GSE31210.Surv_AllMethods_Freq_6 | trial_45 | 0.685348 | 0.674908 | 0.632692 | 0.638645 | 0.675092 | 0.650916 |
| GSE31210.Surv_AllMethods_Freq_2 | trial_46 | 0.61295 | 0.608277 | 0.577361 | 0.547225 | 0.613729 | 0.576144 |
| GSE31210.Surv_AllMethods_Freq_3 | trial_46 | 0.604068 | 0.599744 | 0.568151 | 0.537441 | 0.607754 | 0.567364 |
| GSE31210.Surv_AllMethods_Freq_4 | trial_46 | 0.628683 | 0.624033 | 0.582863 | 0.558673 | 0.615517 | 0.581818 |
| GSE31210.Surv_AllMethods_Freq_5 | trial_46 | 0.633084 | 0.629713 | 0.581273 | 0.559551 | 0.629213 | 0.602434 |
| GSE31210.Surv_AllMethods_Freq_6 | trial_46 | 0.685623 | 0.674908 | 0.632692 | 0.638645 | 0.675092 | 0.650916 |
| GSE31210.Surv_AllMethods_Freq_2 | trial_47 | 0.609348 | 0.608277 | 0.577361 | 0.547225 | 0.613729 | 0.576144 |
| GSE31210.Surv_AllMethods_Freq_3 | trial_47 | 0.600727 | 0.599744 | 0.568151 | 0.537441 | 0.607754 | 0.567364 |
| GSE31210.Surv_AllMethods_Freq_4 | trial_47 | 0.624347 | 0.624033 | 0.582863 | 0.558673 | 0.615517 | 0.581818 |
| GSE31210.Surv_AllMethods_Freq_5 | trial_47 | 0.630524 | 0.629713 | 0.581273 | 0.559551 | 0.629213 | 0.602434 |
| GSE31210.Surv_AllMethods_Freq_6 | trial_47 | 0.685531 | 0.674908 | 0.632692 | 0.638645 | 0.675092 | 0.650916 |
| GSE31210.Surv_AllMethods_Freq_2 | trial_48 | 0.612123 | 0.608277 | 0.577361 | 0.547225 | 0.613729 | 0.576144 |
| GSE31210.Surv_AllMethods_Freq_3 | trial_48 | 0.60284 | 0.599744 | 0.568151 | 0.537441 | 0.607754 | 0.567364 |
| GSE31210.Surv_AllMethods_Freq_4 | trial_48 | 0.627586 | 0.624033 | 0.582863 | 0.558673 | 0.615517 | 0.581818 |
| GSE31210.Surv_AllMethods_Freq_5 | trial_48 | 0.634395 | 0.629713 | 0.581273 | 0.559551 | 0.629213 | 0.602434 |
| GSE31210.Surv_AllMethods_Freq_6 | trial_48 | 0.690018 | 0.674908 | 0.632692 | 0.638645 | 0.675092 | 0.650916 |
| GSE31210.Surv_AllMethods_Freq_2 | trial_49 | 0.611928 | 0.608277 | 0.577361 | 0.547225 | 0.613729 | 0.576144 |
| GSE31210.Surv_AllMethods_Freq_3 | trial_49 | 0.602398 | 0.599744 | 0.568151 | 0.537441 | 0.607754 | 0.567364 |
| GSE31210.Surv_AllMethods_Freq_4 | trial_49 | 0.627325 | 0.624033 | 0.582863 | 0.558673 | 0.615517 | 0.581818 |
| GSE31210.Surv_AllMethods_Freq_5 | trial_49 | 0.633333 | 0.629713 | 0.581273 | 0.559551 | 0.629213 | 0.602434 |
| GSE31210.Surv_AllMethods_Freq_6 | trial_49 | 0.686996 | 0.674908 | 0.632692 | 0.638645 | 0.675092 | 0.650916 |
| GSE31210.Surv_AllMethods_Freq_2 | trial_50 | 0.609883 | 0.608277 | 0.577361 | 0.547225 | 0.613729 | 0.576144 |
| GSE31210.Surv_AllMethods_Freq_3 | trial_50 | 0.600629 | 0.599744 | 0.568151 | 0.537441 | 0.607754 | 0.567364 |
| GSE31210.Surv_AllMethods_Freq_4 | trial_50 | 0.625287 | 0.624033 | 0.582863 | 0.558673 | 0.615517 | 0.581818 |
| GSE31210.Surv_AllMethods_Freq_5 | trial_50 | 0.632022 | 0.629713 | 0.581273 | 0.559551 | 0.629213 | 0.602434 |
| GSE31210.Surv_AllMethods_Freq_6 | trial_50 | 0.686905 | 0.674908 | 0.632692 | 0.638645 | 0.675092 | 0.650916 |
| GSE31210.Surv_AllMethods_Freq_2 | trial_51 | 0.609883 | 0.608277 | 0.577361 | 0.547225 | 0.613729 | 0.576144 |
| GSE31210.Surv_AllMethods_Freq_3 | trial_51 | 0.600776 | 0.599744 | 0.568151 | 0.537441 | 0.607754 | 0.567364 |
| GSE31210.Surv_AllMethods_Freq_4 | trial_51 | 0.62581 | 0.624033 | 0.582863 | 0.558673 | 0.615517 | 0.581818 |
| GSE31210.Surv_AllMethods_Freq_5 | trial_51 | 0.633333 | 0.629713 | 0.581273 | 0.559551 | 0.629213 | 0.602434 |
| GSE31210.Surv_AllMethods_Freq_6 | trial_51 | 0.687821 | 0.674908 | 0.632692 | 0.638645 | 0.675092 | 0.650916 |
| GSE31210.Surv_AllMethods_Freq_2 | trial_52 | 0.610419 | 0.608277 | 0.577361 | 0.547225 | 0.613729 | 0.576144 |
| GSE31210.Surv_AllMethods_Freq_3 | trial_52 | 0.601612 | 0.599744 | 0.568151 | 0.537441 | 0.607754 | 0.567364 |
| GSE31210.Surv_AllMethods_Freq_4 | trial_52 | 0.62675 | 0.624033 | 0.582863 | 0.558673 | 0.615517 | 0.581818 |
| GSE31210.Surv_AllMethods_Freq_5 | trial_52 | 0.631523 | 0.629713 | 0.581273 | 0.559551 | 0.629213 | 0.602434 |
| GSE31210.Surv_AllMethods_Freq_6 | trial_52 | 0.686264 | 0.674908 | 0.632692 | 0.638645 | 0.675092 | 0.650916 |
| GSE31210.Surv_AllMethods_Freq_2 | trial_53 | 0.612415 | 0.608277 | 0.577361 | 0.547225 | 0.613729 | 0.576144 |
| GSE31210.Surv_AllMethods_Freq_3 | trial_53 | 0.60284 | 0.599744 | 0.568151 | 0.537441 | 0.607754 | 0.567364 |
| GSE31210.Surv_AllMethods_Freq_4 | trial_53 | 0.627795 | 0.624033 | 0.582863 | 0.558673 | 0.615517 | 0.581818 |
| GSE31210.Surv_AllMethods_Freq_5 | trial_53 | 0.634769 | 0.629713 | 0.581273 | 0.559551 | 0.629213 | 0.602434 |
| GSE31210.Surv_AllMethods_Freq_6 | trial_53 | 0.68956 | 0.674908 | 0.632692 | 0.638645 | 0.675092 | 0.650916 |
| GSE31210.Surv_AllMethods_Freq_2 | trial_54 | 0.608812 | 0.608277 | 0.577361 | 0.547225 | 0.613729 | 0.576144 |
| GSE31210.Surv_AllMethods_Freq_3 | trial_54 | 0.599204 | 0.599744 | 0.568151 | 0.537441 | 0.607754 | 0.567364 |
| GSE31210.Surv_AllMethods_Freq_4 | trial_54 | 0.623668 | 0.624033 | 0.582863 | 0.558673 | 0.615517 | 0.581818 |
| GSE31210.Surv_AllMethods_Freq_5 | trial_54 | 0.6304 | 0.629713 | 0.581273 | 0.559551 | 0.629213 | 0.602434 |
| GSE31210.Surv_AllMethods_Freq_6 | trial_54 | 0.685348 | 0.674908 | 0.632692 | 0.638645 | 0.675092 | 0.650916 |
| GSE31210.Surv_AllMethods_Freq_2 | trial_55 | 0.606378 | 0.608277 | 0.577361 | 0.547225 | 0.613729 | 0.576144 |
| GSE31210.Surv_AllMethods_Freq_3 | trial_55 | 0.597189 | 0.599744 | 0.568151 | 0.537441 | 0.607754 | 0.567364 |
| GSE31210.Surv_AllMethods_Freq_4 | trial_55 | 0.621578 | 0.624033 | 0.582863 | 0.558673 | 0.615517 | 0.581818 |
| GSE31210.Surv_AllMethods_Freq_5 | trial_55 | 0.627715 | 0.629713 | 0.581273 | 0.559551 | 0.629213 | 0.602434 |
| GSE31210.Surv_AllMethods_Freq_6 | trial_55 | 0.682784 | 0.674908 | 0.632692 | 0.638645 | 0.675092 | 0.650916 |
| GSE31210.Surv_AllMethods_Freq_2 | trial_56 | 0.609737 | 0.608277 | 0.577361 | 0.547225 | 0.613729 | 0.576144 |
| GSE31210.Surv_AllMethods_Freq_3 | trial_56 | 0.600629 | 0.599744 | 0.568151 | 0.537441 | 0.607754 | 0.567364 |
| GSE31210.Surv_AllMethods_Freq_4 | trial_56 | 0.62466 | 0.624033 | 0.582863 | 0.558673 | 0.615517 | 0.581818 |
| GSE31210.Surv_AllMethods_Freq_5 | trial_56 | 0.631398 | 0.629713 | 0.581273 | 0.559551 | 0.629213 | 0.602434 |
| GSE31210.Surv_AllMethods_Freq_6 | trial_56 | 0.687729 | 0.674908 | 0.632692 | 0.638645 | 0.675092 | 0.650916 |
| GSE31210.Surv_AllMethods_Freq_2 | trial_57 | 0.609494 | 0.608277 | 0.577361 | 0.547225 | 0.613729 | 0.576144 |
| GSE31210.Surv_AllMethods_Freq_3 | trial_57 | 0.600383 | 0.599744 | 0.568151 | 0.537441 | 0.607754 | 0.567364 |
| GSE31210.Surv_AllMethods_Freq_4 | trial_57 | 0.625078 | 0.624033 | 0.582863 | 0.558673 | 0.615517 | 0.581818 |
| GSE31210.Surv_AllMethods_Freq_5 | trial_57 | 0.631336 | 0.629713 | 0.581273 | 0.559551 | 0.629213 | 0.602434 |
| GSE31210.Surv_AllMethods_Freq_6 | trial_57 | 0.686905 | 0.674908 | 0.632692 | 0.638645 | 0.675092 | 0.650916 |
| GSE31210.Surv_AllMethods_Freq_2 | trial_58 | 0.609834 | 0.608277 | 0.577361 | 0.547225 | 0.613729 | 0.576144 |
| GSE31210.Surv_AllMethods_Freq_3 | trial_58 | 0.600334 | 0.599744 | 0.568151 | 0.537441 | 0.607754 | 0.567364 |
| GSE31210.Surv_AllMethods_Freq_4 | trial_58 | 0.623877 | 0.624033 | 0.582863 | 0.558673 | 0.615517 | 0.581818 |
| GSE31210.Surv_AllMethods_Freq_5 | trial_58 | 0.627591 | 0.629713 | 0.581273 | 0.559551 | 0.629213 | 0.602434 |
| GSE31210.Surv_AllMethods_Freq_6 | trial_58 | 0.681685 | 0.674908 | 0.632692 | 0.638645 | 0.675092 | 0.650916 |
| GSE31210.Surv_AllMethods_Freq_2 | trial_59 | 0.613486 | 0.608277 | 0.577361 | 0.547225 | 0.613729 | 0.576144 |
| GSE31210.Surv_AllMethods_Freq_3 | trial_59 | 0.604314 | 0.599744 | 0.568151 | 0.537441 | 0.607754 | 0.567364 |
| GSE31210.Surv_AllMethods_Freq_4 | trial_59 | 0.62931 | 0.624033 | 0.582863 | 0.558673 | 0.615517 | 0.581818 |
| GSE31210.Surv_AllMethods_Freq_5 | trial_59 | 0.635518 | 0.629713 | 0.581273 | 0.559551 | 0.629213 | 0.602434 |
| GSE31210.Surv_AllMethods_Freq_6 | trial_59 | 0.691667 | 0.674908 | 0.632692 | 0.638645 | 0.675092 | 0.650916 |
| GSE31210.Surv_AllMethods_Freq_2 | trial_60 | 0.611149 | 0.608277 | 0.577361 | 0.547225 | 0.613729 | 0.576144 |
| GSE31210.Surv_AllMethods_Freq_3 | trial_60 | 0.601366 | 0.599744 | 0.568151 | 0.537441 | 0.607754 | 0.567364 |
| GSE31210.Surv_AllMethods_Freq_4 | trial_60 | 0.62628 | 0.624033 | 0.582863 | 0.558673 | 0.615517 | 0.581818 |
| GSE31210.Surv_AllMethods_Freq_5 | trial_60 | 0.63377 | 0.629713 | 0.581273 | 0.559551 | 0.629213 | 0.602434 |
| GSE31210.Surv_AllMethods_Freq_6 | trial_60 | 0.686355 | 0.674908 | 0.632692 | 0.638645 | 0.675092 | 0.650916 |
| GSE31210.Surv_AllMethods_Freq_2 | trial_61 | 0.613924 | 0.608277 | 0.577361 | 0.547225 | 0.613729 | 0.576144 |
| GSE31210.Surv_AllMethods_Freq_3 | trial_61 | 0.604511 | 0.599744 | 0.568151 | 0.537441 | 0.607754 | 0.567364 |
| GSE31210.Surv_AllMethods_Freq_4 | trial_61 | 0.629833 | 0.624033 | 0.582863 | 0.558673 | 0.615517 | 0.581818 |
| GSE31210.Surv_AllMethods_Freq_5 | trial_61 | 0.635206 | 0.629713 | 0.581273 | 0.559551 | 0.629213 | 0.602434 |
| GSE31210.Surv_AllMethods_Freq_6 | trial_61 | 0.692216 | 0.674908 | 0.632692 | 0.638645 | 0.675092 | 0.650916 |
| GSE31210.Surv_AllMethods_Freq_2 | trial_62 | 0.610565 | 0.608277 | 0.577361 | 0.547225 | 0.613729 | 0.576144 |
| GSE31210.Surv_AllMethods_Freq_3 | trial_62 | 0.601513 | 0.599744 | 0.568151 | 0.537441 | 0.607754 | 0.567364 |
| GSE31210.Surv_AllMethods_Freq_4 | trial_62 | 0.625392 | 0.624033 | 0.582863 | 0.558673 | 0.615517 | 0.581818 |
| GSE31210.Surv_AllMethods_Freq_5 | trial_62 | 0.632397 | 0.629713 | 0.581273 | 0.559551 | 0.629213 | 0.602434 |
| GSE31210.Surv_AllMethods_Freq_6 | trial_62 | 0.684707 | 0.674908 | 0.632692 | 0.638645 | 0.675092 | 0.650916 |
| GSE31210.Surv_AllMethods_Freq_2 | trial_63 | 0.611733 | 0.608277 | 0.577361 | 0.547225 | 0.613729 | 0.576144 |
| GSE31210.Surv_AllMethods_Freq_3 | trial_63 | 0.602447 | 0.599744 | 0.568151 | 0.537441 | 0.607754 | 0.567364 |
| GSE31210.Surv_AllMethods_Freq_4 | trial_63 | 0.627064 | 0.624033 | 0.582863 | 0.558673 | 0.615517 | 0.581818 |
| GSE31210.Surv_AllMethods_Freq_5 | trial_63 | 0.633208 | 0.629713 | 0.581273 | 0.559551 | 0.629213 | 0.602434 |
| GSE31210.Surv_AllMethods_Freq_6 | trial_63 | 0.686355 | 0.674908 | 0.632692 | 0.638645 | 0.675092 | 0.650916 |
| GSE31210.Surv_AllMethods_Freq_2 | trial_64 | 0.6111 | 0.608277 | 0.577361 | 0.547225 | 0.613729 | 0.576144 |
| GSE31210.Surv_AllMethods_Freq_3 | trial_64 | 0.602005 | 0.599744 | 0.568151 | 0.537441 | 0.607754 | 0.567364 |
| GSE31210.Surv_AllMethods_Freq_4 | trial_64 | 0.626228 | 0.624033 | 0.582863 | 0.558673 | 0.615517 | 0.581818 |
| GSE31210.Surv_AllMethods_Freq_5 | trial_64 | 0.63171 | 0.629713 | 0.581273 | 0.559551 | 0.629213 | 0.602434 |
| GSE31210.Surv_AllMethods_Freq_6 | trial_64 | 0.687454 | 0.674908 | 0.632692 | 0.638645 | 0.675092 | 0.650916 |
| GSE31210.Surv_AllMethods_Freq_2 | trial_65 | 0.612171 | 0.608277 | 0.577361 | 0.547225 | 0.613729 | 0.576144 |
| GSE31210.Surv_AllMethods_Freq_3 | trial_65 | 0.602742 | 0.599744 | 0.568151 | 0.537441 | 0.607754 | 0.567364 |
| GSE31210.Surv_AllMethods_Freq_4 | trial_65 | 0.626907 | 0.624033 | 0.582863 | 0.558673 | 0.615517 | 0.581818 |
| GSE31210.Surv_AllMethods_Freq_5 | trial_65 | 0.632335 | 0.629713 | 0.581273 | 0.559551 | 0.629213 | 0.602434 |
| GSE31210.Surv_AllMethods_Freq_6 | trial_65 | 0.685806 | 0.674908 | 0.632692 | 0.638645 | 0.675092 | 0.650916 |
| GSE31210.Surv_AllMethods_Freq_2 | trial_66 | 0.609104 | 0.608277 | 0.577361 | 0.547225 | 0.613729 | 0.576144 |
| GSE31210.Surv_AllMethods_Freq_3 | trial_66 | 0.599597 | 0.599744 | 0.568151 | 0.537441 | 0.607754 | 0.567364 |
| GSE31210.Surv_AllMethods_Freq_4 | trial_66 | 0.624765 | 0.624033 | 0.582863 | 0.558673 | 0.615517 | 0.581818 |
| GSE31210.Surv_AllMethods_Freq_5 | trial_66 | 0.630649 | 0.629713 | 0.581273 | 0.559551 | 0.629213 | 0.602434 |
| GSE31210.Surv_AllMethods_Freq_6 | trial_66 | 0.686447 | 0.674908 | 0.632692 | 0.638645 | 0.675092 | 0.650916 |
| GSE31210.Surv_AllMethods_Freq_2 | trial_67 | 0.611344 | 0.608277 | 0.577361 | 0.547225 | 0.613729 | 0.576144 |
| GSE31210.Surv_AllMethods_Freq_3 | trial_67 | 0.602447 | 0.599744 | 0.568151 | 0.537441 | 0.607754 | 0.567364 |
| GSE31210.Surv_AllMethods_Freq_4 | trial_67 | 0.627168 | 0.624033 | 0.582863 | 0.558673 | 0.615517 | 0.581818 |
| GSE31210.Surv_AllMethods_Freq_5 | trial_67 | 0.634582 | 0.629713 | 0.581273 | 0.559551 | 0.629213 | 0.602434 |
| GSE31210.Surv_AllMethods_Freq_6 | trial_67 | 0.690568 | 0.674908 | 0.632692 | 0.638645 | 0.675092 | 0.650916 |
| GSE31210.Surv_AllMethods_Freq_2 | trial_68 | 0.612171 | 0.608277 | 0.577361 | 0.547225 | 0.613729 | 0.576144 |
| GSE31210.Surv_AllMethods_Freq_3 | trial_68 | 0.602791 | 0.599744 | 0.568151 | 0.537441 | 0.607754 | 0.567364 |
| GSE31210.Surv_AllMethods_Freq_4 | trial_68 | 0.627273 | 0.624033 | 0.582863 | 0.558673 | 0.615517 | 0.581818 |
| GSE31210.Surv_AllMethods_Freq_5 | trial_68 | 0.633084 | 0.629713 | 0.581273 | 0.559551 | 0.629213 | 0.602434 |
| GSE31210.Surv_AllMethods_Freq_6 | trial_68 | 0.689286 | 0.674908 | 0.632692 | 0.638645 | 0.675092 | 0.650916 |
| GSE31210.Surv_AllMethods_Freq_2 | trial_69 | 0.611246 | 0.608277 | 0.577361 | 0.547225 | 0.613729 | 0.576144 |
| GSE31210.Surv_AllMethods_Freq_3 | trial_69 | 0.60171 | 0.599744 | 0.568151 | 0.537441 | 0.607754 | 0.567364 |
| GSE31210.Surv_AllMethods_Freq_4 | trial_69 | 0.626176 | 0.624033 | 0.582863 | 0.558673 | 0.615517 | 0.581818 |
| GSE31210.Surv_AllMethods_Freq_5 | trial_69 | 0.631898 | 0.629713 | 0.581273 | 0.559551 | 0.629213 | 0.602434 |
| GSE31210.Surv_AllMethods_Freq_6 | trial_69 | 0.686447 | 0.674908 | 0.632692 | 0.638645 | 0.675092 | 0.650916 |
| GSE31210.Surv_AllMethods_Freq_2 | trial_70 | 0.610516 | 0.608277 | 0.577361 | 0.547225 | 0.613729 | 0.576144 |
| GSE31210.Surv_AllMethods_Freq_3 | trial_70 | 0.601219 | 0.599744 | 0.568151 | 0.537441 | 0.607754 | 0.567364 |
| GSE31210.Surv_AllMethods_Freq_4 | trial_70 | 0.626071 | 0.624033 | 0.582863 | 0.558673 | 0.615517 | 0.581818 |
| GSE31210.Surv_AllMethods_Freq_5 | trial_70 | 0.634332 | 0.629713 | 0.581273 | 0.559551 | 0.629213 | 0.602434 |
| GSE31210.Surv_AllMethods_Freq_6 | trial_70 | 0.688095 | 0.674908 | 0.632692 | 0.638645 | 0.675092 | 0.650916 |
| GSE31210.Surv_AllMethods_Freq_2 | trial_71 | 0.609786 | 0.608277 | 0.577361 | 0.547225 | 0.613729 | 0.576144 |
| GSE31210.Surv_AllMethods_Freq_3 | trial_71 | 0.600482 | 0.599744 | 0.568151 | 0.537441 | 0.607754 | 0.567364 |
| GSE31210.Surv_AllMethods_Freq_4 | trial_71 | 0.62466 | 0.624033 | 0.582863 | 0.558673 | 0.615517 | 0.581818 |
| GSE31210.Surv_AllMethods_Freq_5 | trial_71 | 0.628901 | 0.629713 | 0.581273 | 0.559551 | 0.629213 | 0.602434 |
| GSE31210.Surv_AllMethods_Freq_6 | trial_71 | 0.68315 | 0.674908 | 0.632692 | 0.638645 | 0.675092 | 0.650916 |
| GSE31210.Surv_AllMethods_Freq_2 | trial_72 | 0.608179 | 0.608277 | 0.577361 | 0.547225 | 0.613729 | 0.576144 |
| GSE31210.Surv_AllMethods_Freq_3 | trial_72 | 0.599106 | 0.599744 | 0.568151 | 0.537441 | 0.607754 | 0.567364 |
| GSE31210.Surv_AllMethods_Freq_4 | trial_72 | 0.623354 | 0.624033 | 0.582863 | 0.558673 | 0.615517 | 0.581818 |
| GSE31210.Surv_AllMethods_Freq_5 | trial_72 | 0.629713 | 0.629713 | 0.581273 | 0.559551 | 0.629213 | 0.602434 |
| GSE31210.Surv_AllMethods_Freq_6 | trial_72 | 0.680861 | 0.674908 | 0.632692 | 0.638645 | 0.675092 | 0.650916 |
| GSE31210.Surv_AllMethods_Freq_2 | trial_73 | 0.608423 | 0.608277 | 0.577361 | 0.547225 | 0.613729 | 0.576144 |
| GSE31210.Surv_AllMethods_Freq_3 | trial_73 | 0.599548 | 0.599744 | 0.568151 | 0.537441 | 0.607754 | 0.567364 |
| GSE31210.Surv_AllMethods_Freq_4 | trial_73 | 0.623145 | 0.624033 | 0.582863 | 0.558673 | 0.615517 | 0.581818 |
| GSE31210.Surv_AllMethods_Freq_5 | trial_73 | 0.627965 | 0.629713 | 0.581273 | 0.559551 | 0.629213 | 0.602434 |
| GSE31210.Surv_AllMethods_Freq_6 | trial_73 | 0.682601 | 0.674908 | 0.632692 | 0.638645 | 0.675092 | 0.650916 |
| GSE31210.Surv_AllMethods_Freq_2 | trial_74 | 0.610467 | 0.608277 | 0.577361 | 0.547225 | 0.613729 | 0.576144 |
| GSE31210.Surv_AllMethods_Freq_3 | trial_74 | 0.601317 | 0.599744 | 0.568151 | 0.537441 | 0.607754 | 0.567364 |
| GSE31210.Surv_AllMethods_Freq_4 | trial_74 | 0.62675 | 0.624033 | 0.582863 | 0.558673 | 0.615517 | 0.581818 |
| GSE31210.Surv_AllMethods_Freq_5 | trial_74 | 0.633458 | 0.629713 | 0.581273 | 0.559551 | 0.629213 | 0.602434 |
| GSE31210.Surv_AllMethods_Freq_6 | trial_74 | 0.687271 | 0.674908 | 0.632692 | 0.638645 | 0.675092 | 0.650916 |
| GSE31210.Surv_AllMethods_Freq_2 | trial_75 | 0.612463 | 0.608277 | 0.577361 | 0.547225 | 0.613729 | 0.576144 |
| GSE31210.Surv_AllMethods_Freq_3 | trial_75 | 0.603479 | 0.599744 | 0.568151 | 0.537441 | 0.607754 | 0.567364 |
| GSE31210.Surv_AllMethods_Freq_4 | trial_75 | 0.62884 | 0.624033 | 0.582863 | 0.558673 | 0.615517 | 0.581818 |
| GSE31210.Surv_AllMethods_Freq_5 | trial_75 | 0.634457 | 0.629713 | 0.581273 | 0.559551 | 0.629213 | 0.602434 |
| GSE31210.Surv_AllMethods_Freq_6 | trial_75 | 0.69011 | 0.674908 | 0.632692 | 0.638645 | 0.675092 | 0.650916 |
| GSE31210.Surv_AllMethods_Freq_2 | trial_76 | 0.611052 | 0.608277 | 0.577361 | 0.547225 | 0.613729 | 0.576144 |
| GSE31210.Surv_AllMethods_Freq_3 | trial_76 | 0.602447 | 0.599744 | 0.568151 | 0.537441 | 0.607754 | 0.567364 |
| GSE31210.Surv_AllMethods_Freq_4 | trial_76 | 0.627325 | 0.624033 | 0.582863 | 0.558673 | 0.615517 | 0.581818 |
| GSE31210.Surv_AllMethods_Freq_5 | trial_76 | 0.632772 | 0.629713 | 0.581273 | 0.559551 | 0.629213 | 0.602434 |
| GSE31210.Surv_AllMethods_Freq_6 | trial_76 | 0.685256 | 0.674908 | 0.632692 | 0.638645 | 0.675092 | 0.650916 |
| GSE31210.Surv_AllMethods_Freq_2 | trial_77 | 0.609202 | 0.608277 | 0.577361 | 0.547225 | 0.613729 | 0.576144 |
| GSE31210.Surv_AllMethods_Freq_3 | trial_77 | 0.600236 | 0.599744 | 0.568151 | 0.537441 | 0.607754 | 0.567364 |
| GSE31210.Surv_AllMethods_Freq_4 | trial_77 | 0.624451 | 0.624033 | 0.582863 | 0.558673 | 0.615517 | 0.581818 |
| GSE31210.Surv_AllMethods_Freq_5 | trial_77 | 0.630649 | 0.629713 | 0.581273 | 0.559551 | 0.629213 | 0.602434 |
| GSE31210.Surv_AllMethods_Freq_6 | trial_77 | 0.685073 | 0.674908 | 0.632692 | 0.638645 | 0.675092 | 0.650916 |
| GSE31210.Surv_AllMethods_Freq_2 | trial_78 | 0.609542 | 0.608277 | 0.577361 | 0.547225 | 0.613729 | 0.576144 |
| GSE31210.Surv_AllMethods_Freq_3 | trial_78 | 0.600187 | 0.599744 | 0.568151 | 0.537441 | 0.607754 | 0.567364 |
| GSE31210.Surv_AllMethods_Freq_4 | trial_78 | 0.625131 | 0.624033 | 0.582863 | 0.558673 | 0.615517 | 0.581818 |
| GSE31210.Surv_AllMethods_Freq_5 | trial_78 | 0.631898 | 0.629713 | 0.581273 | 0.559551 | 0.629213 | 0.602434 |
| GSE31210.Surv_AllMethods_Freq_6 | trial_78 | 0.686264 | 0.674908 | 0.632692 | 0.638645 | 0.675092 | 0.650916 |
| GSE31210.Surv_AllMethods_Freq_2 | trial_79 | 0.611636 | 0.608277 | 0.577361 | 0.547225 | 0.613729 | 0.576144 |
| GSE31210.Surv_AllMethods_Freq_3 | trial_79 | 0.602496 | 0.599744 | 0.568151 | 0.537441 | 0.607754 | 0.567364 |
| GSE31210.Surv_AllMethods_Freq_4 | trial_79 | 0.626541 | 0.624033 | 0.582863 | 0.558673 | 0.615517 | 0.581818 |
| GSE31210.Surv_AllMethods_Freq_5 | trial_79 | 0.63402 | 0.629713 | 0.581273 | 0.559551 | 0.629213 | 0.602434 |
| GSE31210.Surv_AllMethods_Freq_6 | trial_79 | 0.688187 | 0.674908 | 0.632692 | 0.638645 | 0.675092 | 0.650916 |
| GSE31210.Surv_AllMethods_Freq_2 | trial_80 | 0.607692 | 0.608277 | 0.577361 | 0.547225 | 0.613729 | 0.576144 |
| GSE31210.Surv_AllMethods_Freq_3 | trial_80 | 0.598025 | 0.599744 | 0.568151 | 0.537441 | 0.607754 | 0.567364 |
| GSE31210.Surv_AllMethods_Freq_4 | trial_80 | 0.622048 | 0.624033 | 0.582863 | 0.558673 | 0.615517 | 0.581818 |
| GSE31210.Surv_AllMethods_Freq_5 | trial_80 | 0.628402 | 0.629713 | 0.581273 | 0.559551 | 0.629213 | 0.602434 |
| GSE31210.Surv_AllMethods_Freq_6 | trial_80 | 0.684158 | 0.674908 | 0.632692 | 0.638645 | 0.675092 | 0.650916 |
| GSE31210.Surv_AllMethods_Freq_2 | trial_81 | 0.609737 | 0.608277 | 0.577361 | 0.547225 | 0.613729 | 0.576144 |
| GSE31210.Surv_AllMethods_Freq_3 | trial_81 | 0.600187 | 0.599744 | 0.568151 | 0.537441 | 0.607754 | 0.567364 |
| GSE31210.Surv_AllMethods_Freq_4 | trial_81 | 0.624974 | 0.624033 | 0.582863 | 0.558673 | 0.615517 | 0.581818 |
| GSE31210.Surv_AllMethods_Freq_5 | trial_81 | 0.632959 | 0.629713 | 0.581273 | 0.559551 | 0.629213 | 0.602434 |
| GSE31210.Surv_AllMethods_Freq_6 | trial_81 | 0.684158 | 0.674908 | 0.632692 | 0.638645 | 0.675092 | 0.650916 |
| GSE31210.Surv_AllMethods_Freq_2 | trial_82 | 0.610321 | 0.608277 | 0.577361 | 0.547225 | 0.613729 | 0.576144 |
| GSE31210.Surv_AllMethods_Freq_3 | trial_82 | 0.601661 | 0.599744 | 0.568151 | 0.537441 | 0.607754 | 0.567364 |
| GSE31210.Surv_AllMethods_Freq_4 | trial_82 | 0.62628 | 0.624033 | 0.582863 | 0.558673 | 0.615517 | 0.581818 |
| GSE31210.Surv_AllMethods_Freq_5 | trial_82 | 0.633958 | 0.629713 | 0.581273 | 0.559551 | 0.629213 | 0.602434 |
| GSE31210.Surv_AllMethods_Freq_6 | trial_82 | 0.68837 | 0.674908 | 0.632692 | 0.638645 | 0.675092 | 0.650916 |
| GSE31210.Surv_AllMethods_Freq_2 | trial_83 | 0.614752 | 0.608277 | 0.577361 | 0.547225 | 0.613729 | 0.576144 |
| GSE31210.Surv_AllMethods_Freq_3 | trial_83 | 0.605346 | 0.599744 | 0.568151 | 0.537441 | 0.607754 | 0.567364 |
| GSE31210.Surv_AllMethods_Freq_4 | trial_83 | 0.629676 | 0.624033 | 0.582863 | 0.558673 | 0.615517 | 0.581818 |
| GSE31210.Surv_AllMethods_Freq_5 | trial_83 | 0.635019 | 0.629713 | 0.581273 | 0.559551 | 0.629213 | 0.602434 |
| GSE31210.Surv_AllMethods_Freq_6 | trial_83 | 0.690476 | 0.674908 | 0.632692 | 0.638645 | 0.675092 | 0.650916 |
| GSE31210.Surv_AllMethods_Freq_2 | trial_84 | 0.608325 | 0.608277 | 0.577361 | 0.547225 | 0.613729 | 0.576144 |
| GSE31210.Surv_AllMethods_Freq_3 | trial_84 | 0.599843 | 0.599744 | 0.568151 | 0.537441 | 0.607754 | 0.567364 |
| GSE31210.Surv_AllMethods_Freq_4 | trial_84 | 0.623877 | 0.624033 | 0.582863 | 0.558673 | 0.615517 | 0.581818 |
| GSE31210.Surv_AllMethods_Freq_5 | trial_84 | 0.628215 | 0.629713 | 0.581273 | 0.559551 | 0.629213 | 0.602434 |
| GSE31210.Surv_AllMethods_Freq_6 | trial_84 | 0.684158 | 0.674908 | 0.632692 | 0.638645 | 0.675092 | 0.650916 |
| GSE31210.Surv_AllMethods_Freq_2 | trial_85 | 0.610662 | 0.608277 | 0.577361 | 0.547225 | 0.613729 | 0.576144 |
| GSE31210.Surv_AllMethods_Freq_3 | trial_85 | 0.601464 | 0.599744 | 0.568151 | 0.537441 | 0.607754 | 0.567364 |
| GSE31210.Surv_AllMethods_Freq_4 | trial_85 | 0.626803 | 0.624033 | 0.582863 | 0.558673 | 0.615517 | 0.581818 |
| GSE31210.Surv_AllMethods_Freq_5 | trial_85 | 0.63221 | 0.629713 | 0.581273 | 0.559551 | 0.629213 | 0.602434 |
| GSE31210.Surv_AllMethods_Freq_6 | trial_85 | 0.686447 | 0.674908 | 0.632692 | 0.638645 | 0.675092 | 0.650916 |
| GSE31210.Surv_AllMethods_Freq_2 | trial_86 | 0.607936 | 0.608277 | 0.577361 | 0.547225 | 0.613729 | 0.576144 |
| GSE31210.Surv_AllMethods_Freq_3 | trial_86 | 0.598516 | 0.599744 | 0.568151 | 0.537441 | 0.607754 | 0.567364 |
| GSE31210.Surv_AllMethods_Freq_4 | trial_86 | 0.623459 | 0.624033 | 0.582863 | 0.558673 | 0.615517 | 0.581818 |
| GSE31210.Surv_AllMethods_Freq_5 | trial_86 | 0.629963 | 0.629713 | 0.581273 | 0.559551 | 0.629213 | 0.602434 |
| GSE31210.Surv_AllMethods_Freq_6 | trial_86 | 0.680037 | 0.674908 | 0.632692 | 0.638645 | 0.675092 | 0.650916 |
| GSE31210.Surv_AllMethods_Freq_2 | trial_87 | 0.610613 | 0.608277 | 0.577361 | 0.547225 | 0.613729 | 0.576144 |
| GSE31210.Surv_AllMethods_Freq_3 | trial_87 | 0.601268 | 0.599744 | 0.568151 | 0.537441 | 0.607754 | 0.567364 |
| GSE31210.Surv_AllMethods_Freq_4 | trial_87 | 0.624451 | 0.624033 | 0.582863 | 0.558673 | 0.615517 | 0.581818 |
| GSE31210.Surv_AllMethods_Freq_5 | trial_87 | 0.629026 | 0.629713 | 0.581273 | 0.559551 | 0.629213 | 0.602434 |
| GSE31210.Surv_AllMethods_Freq_6 | trial_87 | 0.68141 | 0.674908 | 0.632692 | 0.638645 | 0.675092 | 0.650916 |
| GSE31210.Surv_AllMethods_Freq_2 | trial_88 | 0.612902 | 0.608277 | 0.577361 | 0.547225 | 0.613729 | 0.576144 |
| GSE31210.Surv_AllMethods_Freq_3 | trial_88 | 0.603872 | 0.599744 | 0.568151 | 0.537441 | 0.607754 | 0.567364 |
| GSE31210.Surv_AllMethods_Freq_4 | trial_88 | 0.628056 | 0.624033 | 0.582863 | 0.558673 | 0.615517 | 0.581818 |
| GSE31210.Surv_AllMethods_Freq_5 | trial_88 | 0.635955 | 0.629713 | 0.581273 | 0.559551 | 0.629213 | 0.602434 |
| GSE31210.Surv_AllMethods_Freq_6 | trial_88 | 0.689927 | 0.674908 | 0.632692 | 0.638645 | 0.675092 | 0.650916 |
| GSE31210.Surv_AllMethods_Freq_2 | trial_89 | 0.60964 | 0.608277 | 0.577361 | 0.547225 | 0.613729 | 0.576144 |
| GSE31210.Surv_AllMethods_Freq_3 | trial_89 | 0.599941 | 0.599744 | 0.568151 | 0.537441 | 0.607754 | 0.567364 |
| GSE31210.Surv_AllMethods_Freq_4 | trial_89 | 0.624033 | 0.624033 | 0.582863 | 0.558673 | 0.615517 | 0.581818 |
| GSE31210.Surv_AllMethods_Freq_5 | trial_89 | 0.62965 | 0.629713 | 0.581273 | 0.559551 | 0.629213 | 0.602434 |
| GSE31210.Surv_AllMethods_Freq_6 | trial_89 | 0.682143 | 0.674908 | 0.632692 | 0.638645 | 0.675092 | 0.650916 |
| GSE31210.Surv_AllMethods_Freq_2 | trial_90 | 0.612902 | 0.608277 | 0.577361 | 0.547225 | 0.613729 | 0.576144 |
| GSE31210.Surv_AllMethods_Freq_3 | trial_90 | 0.603479 | 0.599744 | 0.568151 | 0.537441 | 0.607754 | 0.567364 |
| GSE31210.Surv_AllMethods_Freq_4 | trial_90 | 0.627116 | 0.624033 | 0.582863 | 0.558673 | 0.615517 | 0.581818 |
| GSE31210.Surv_AllMethods_Freq_5 | trial_90 | 0.633958 | 0.629713 | 0.581273 | 0.559551 | 0.629213 | 0.602434 |
| GSE31210.Surv_AllMethods_Freq_6 | trial_90 | 0.692857 | 0.674908 | 0.632692 | 0.638645 | 0.675092 | 0.650916 |
| GSE31210.Surv_AllMethods_Freq_2 | trial_91 | 0.609348 | 0.608277 | 0.577361 | 0.547225 | 0.613729 | 0.576144 |
| GSE31210.Surv_AllMethods_Freq_3 | trial_91 | 0.600875 | 0.599744 | 0.568151 | 0.537441 | 0.607754 | 0.567364 |
| GSE31210.Surv_AllMethods_Freq_4 | trial_91 | 0.625758 | 0.624033 | 0.582863 | 0.558673 | 0.615517 | 0.581818 |
| GSE31210.Surv_AllMethods_Freq_5 | trial_91 | 0.632896 | 0.629713 | 0.581273 | 0.559551 | 0.629213 | 0.602434 |
| GSE31210.Surv_AllMethods_Freq_6 | trial_91 | 0.685348 | 0.674908 | 0.632692 | 0.638645 | 0.675092 | 0.650916 |
| GSE31210.Surv_AllMethods_Freq_2 | trial_92 | 0.612658 | 0.608277 | 0.577361 | 0.547225 | 0.613729 | 0.576144 |
| GSE31210.Surv_AllMethods_Freq_3 | trial_92 | 0.603479 | 0.599744 | 0.568151 | 0.537441 | 0.607754 | 0.567364 |
| GSE31210.Surv_AllMethods_Freq_4 | trial_92 | 0.628213 | 0.624033 | 0.582863 | 0.558673 | 0.615517 | 0.581818 |
| GSE31210.Surv_AllMethods_Freq_5 | trial_92 | 0.633458 | 0.629713 | 0.581273 | 0.559551 | 0.629213 | 0.602434 |
| GSE31210.Surv_AllMethods_Freq_6 | trial_92 | 0.687546 | 0.674908 | 0.632692 | 0.638645 | 0.675092 | 0.650916 |
| GSE31210.Surv_AllMethods_Freq_2 | trial_93 | 0.609981 | 0.608277 | 0.577361 | 0.547225 | 0.613729 | 0.576144 |
| GSE31210.Surv_AllMethods_Freq_3 | trial_93 | 0.60058 | 0.599744 | 0.568151 | 0.537441 | 0.607754 | 0.567364 |
| GSE31210.Surv_AllMethods_Freq_4 | trial_93 | 0.624399 | 0.624033 | 0.582863 | 0.558673 | 0.615517 | 0.581818 |
| GSE31210.Surv_AllMethods_Freq_5 | trial_93 | 0.631086 | 0.629713 | 0.581273 | 0.559551 | 0.629213 | 0.602434 |
| GSE31210.Surv_AllMethods_Freq_6 | trial_93 | 0.68489 | 0.674908 | 0.632692 | 0.638645 | 0.675092 | 0.650916 |
| GSE31210.Surv_AllMethods_Freq_2 | trial_94 | 0.609396 | 0.608277 | 0.577361 | 0.547225 | 0.613729 | 0.576144 |
| GSE31210.Surv_AllMethods_Freq_3 | trial_94 | 0.599892 | 0.599744 | 0.568151 | 0.537441 | 0.607754 | 0.567364 |
| GSE31210.Surv_AllMethods_Freq_4 | trial_94 | 0.624974 | 0.624033 | 0.582863 | 0.558673 | 0.615517 | 0.581818 |
| GSE31210.Surv_AllMethods_Freq_5 | trial_94 | 0.631461 | 0.629713 | 0.581273 | 0.559551 | 0.629213 | 0.602434 |
| GSE31210.Surv_AllMethods_Freq_6 | trial_94 | 0.684982 | 0.674908 | 0.632692 | 0.638645 | 0.675092 | 0.650916 |
| GSE31210.Surv_AllMethods_Freq_2 | trial_95 | 0.613827 | 0.608277 | 0.577361 | 0.547225 | 0.613729 | 0.576144 |
| GSE31210.Surv_AllMethods_Freq_3 | trial_95 | 0.604363 | 0.599744 | 0.568151 | 0.537441 | 0.607754 | 0.567364 |
| GSE31210.Surv_AllMethods_Freq_4 | trial_95 | 0.629101 | 0.624033 | 0.582863 | 0.558673 | 0.615517 | 0.581818 |
| GSE31210.Surv_AllMethods_Freq_5 | trial_95 | 0.636454 | 0.629713 | 0.581273 | 0.559551 | 0.629213 | 0.602434 |
| GSE31210.Surv_AllMethods_Freq_6 | trial_95 | 0.690476 | 0.674908 | 0.632692 | 0.638645 | 0.675092 | 0.650916 |
| GSE31210.Surv_AllMethods_Freq_2 | trial_96 | 0.609737 | 0.608277 | 0.577361 | 0.547225 | 0.613729 | 0.576144 |
| GSE31210.Surv_AllMethods_Freq_3 | trial_96 | 0.600285 | 0.599744 | 0.568151 | 0.537441 | 0.607754 | 0.567364 |
| GSE31210.Surv_AllMethods_Freq_4 | trial_96 | 0.62466 | 0.624033 | 0.582863 | 0.558673 | 0.615517 | 0.581818 |
| GSE31210.Surv_AllMethods_Freq_5 | trial_96 | 0.629963 | 0.629713 | 0.581273 | 0.559551 | 0.629213 | 0.602434 |
| GSE31210.Surv_AllMethods_Freq_6 | trial_96 | 0.686264 | 0.674908 | 0.632692 | 0.638645 | 0.675092 | 0.650916 |
| GSE31210.Surv_AllMethods_Freq_2 | trial_97 | 0.608666 | 0.608277 | 0.577361 | 0.547225 | 0.613729 | 0.576144 |
| GSE31210.Surv_AllMethods_Freq_3 | trial_97 | 0.599401 | 0.599744 | 0.568151 | 0.537441 | 0.607754 | 0.567364 |
| GSE31210.Surv_AllMethods_Freq_4 | trial_97 | 0.62372 | 0.624033 | 0.582863 | 0.558673 | 0.615517 | 0.581818 |
| GSE31210.Surv_AllMethods_Freq_5 | trial_97 | 0.631149 | 0.629713 | 0.581273 | 0.559551 | 0.629213 | 0.602434 |
| GSE31210.Surv_AllMethods_Freq_6 | trial_97 | 0.686264 | 0.674908 | 0.632692 | 0.638645 | 0.675092 | 0.650916 |
| GSE31210.Surv_AllMethods_Freq_2 | trial_98 | 0.611149 | 0.608277 | 0.577361 | 0.547225 | 0.613729 | 0.576144 |
| GSE31210.Surv_AllMethods_Freq_3 | trial_98 | 0.602201 | 0.599744 | 0.568151 | 0.537441 | 0.607754 | 0.567364 |
| GSE31210.Surv_AllMethods_Freq_4 | trial_98 | 0.626646 | 0.624033 | 0.582863 | 0.558673 | 0.615517 | 0.581818 |
| GSE31210.Surv_AllMethods_Freq_5 | trial_98 | 0.630774 | 0.629713 | 0.581273 | 0.559551 | 0.629213 | 0.602434 |
| GSE31210.Surv_AllMethods_Freq_6 | trial_98 | 0.686447 | 0.674908 | 0.632692 | 0.638645 | 0.675092 | 0.650916 |
| GSE31210.Surv_AllMethods_Freq_2 | trial_99 | 0.610127 | 0.608277 | 0.577361 | 0.547225 | 0.613729 | 0.576144 |
| GSE31210.Surv_AllMethods_Freq_3 | trial_99 | 0.600383 | 0.599744 | 0.568151 | 0.537441 | 0.607754 | 0.567364 |
| GSE31210.Surv_AllMethods_Freq_4 | trial_99 | 0.62419 | 0.624033 | 0.582863 | 0.558673 | 0.615517 | 0.581818 |
| GSE31210.Surv_AllMethods_Freq_5 | trial_99 | 0.631024 | 0.629713 | 0.581273 | 0.559551 | 0.629213 | 0.602434 |
| GSE31210.Surv_AllMethods_Freq_6 | trial_99 | 0.683791 | 0.674908 | 0.632692 | 0.638645 | 0.675092 | 0.650916 |
| GSE31210.Surv_AllMethods_Freq_2 | trial_100 | 0.608471 | 0.608277 | 0.577361 | 0.547225 | 0.613729 | 0.576144 |
| GSE31210.Surv_AllMethods_Freq_3 | trial_100 | 0.599204 | 0.599744 | 0.568151 | 0.537441 | 0.607754 | 0.567364 |
| GSE31210.Surv_AllMethods_Freq_4 | trial_100 | 0.623563 | 0.624033 | 0.582863 | 0.558673 | 0.615517 | 0.581818 |
| GSE31210.Surv_AllMethods_Freq_5 | trial_100 | 0.629588 | 0.629713 | 0.581273 | 0.559551 | 0.629213 | 0.602434 |
| GSE31210.Surv_AllMethods_Freq_6 | trial_100 | 0.686264 | 0.674908 | 0.632692 | 0.638645 | 0.675092 | 0.650916 |
| GSE31210.SurvRelapseFree_AllMethods_Freq_2 | trial_1 | 0.567756 | 0.545359 | 0.525666 | 0.494754 | 0.531195 | 0.500807 |
| GSE31210.SurvRelapseFree_AllMethods_Freq_3 | trial_1 | 0.578062 | 0.55239 | 0.529293 | 0.509939 | 0.537422 | 0.502575 |
| GSE31210.SurvRelapseFree_AllMethods_Freq_4 | trial_1 | 0.575201 | 0.539694 | 0.528744 | 0.511755 | 0.538607 | 0.510749 |
| GSE31210.SurvRelapseFree_AllMethods_Freq_5 | trial_1 | 0.585567 | 0.560773 | 0.551809 | 0.53491 | 0.585567 | 0.54449 |
| GSE31210.SurvRelapseFree_AllMethods_Freq_6 | trial_1 | 0.616586 | 0.589375 | 0.573443 | 0.578947 | 0.594835 | 0.578634 |
| GSE31210.SurvRelapseFree_AllMethods_Freq_2 | trial_2 | 0.565981 | 0.545359 | 0.525666 | 0.494754 | 0.531195 | 0.500807 |
| GSE31210.SurvRelapseFree_AllMethods_Freq_3 | trial_2 | 0.576734 | 0.55239 | 0.529293 | 0.509939 | 0.537422 | 0.502575 |
| GSE31210.SurvRelapseFree_AllMethods_Freq_4 | trial_2 | 0.572786 | 0.539694 | 0.528744 | 0.511755 | 0.538607 | 0.510749 |
| GSE31210.SurvRelapseFree_AllMethods_Freq_5 | trial_2 | 0.583758 | 0.560773 | 0.551809 | 0.53491 | 0.585567 | 0.54449 |
| GSE31210.SurvRelapseFree_AllMethods_Freq_6 | trial_2 | 0.614438 | 0.589375 | 0.573443 | 0.578947 | 0.594835 | 0.578634 |
| GSE31210.SurvRelapseFree_AllMethods_Freq_2 | trial_3 | 0.570541 | 0.545359 | 0.525666 | 0.494754 | 0.531195 | 0.500807 |
| GSE31210.SurvRelapseFree_AllMethods_Freq_3 | trial_3 | 0.580517 | 0.55239 | 0.529293 | 0.509939 | 0.537422 | 0.502575 |
| GSE31210.SurvRelapseFree_AllMethods_Freq_4 | trial_3 | 0.576973 | 0.539694 | 0.528744 | 0.511755 | 0.538607 | 0.510749 |
| GSE31210.SurvRelapseFree_AllMethods_Freq_5 | trial_3 | 0.587171 | 0.560773 | 0.551809 | 0.53491 | 0.585567 | 0.54449 |
| GSE31210.SurvRelapseFree_AllMethods_Freq_6 | trial_3 | 0.617481 | 0.589375 | 0.573443 | 0.578947 | 0.594835 | 0.578634 |
| GSE31210.SurvRelapseFree_AllMethods_Freq_2 | trial_4 | 0.569169 | 0.545359 | 0.525666 | 0.494754 | 0.531195 | 0.500807 |
| GSE31210.SurvRelapseFree_AllMethods_Freq_3 | trial_4 | 0.579068 | 0.55239 | 0.529293 | 0.509939 | 0.537422 | 0.502575 |
| GSE31210.SurvRelapseFree_AllMethods_Freq_4 | trial_4 | 0.575161 | 0.539694 | 0.528744 | 0.511755 | 0.538607 | 0.510749 |
| GSE31210.SurvRelapseFree_AllMethods_Freq_5 | trial_4 | 0.585074 | 0.560773 | 0.551809 | 0.53491 | 0.585567 | 0.54449 |
| GSE31210.SurvRelapseFree_AllMethods_Freq_6 | trial_4 | 0.616676 | 0.589375 | 0.573443 | 0.578947 | 0.594835 | 0.578634 |
| GSE31210.SurvRelapseFree_AllMethods_Freq_2 | trial_5 | 0.567514 | 0.545359 | 0.525666 | 0.494754 | 0.531195 | 0.500807 |
| GSE31210.SurvRelapseFree_AllMethods_Freq_3 | trial_5 | 0.57762 | 0.55239 | 0.529293 | 0.509939 | 0.537422 | 0.502575 |
| GSE31210.SurvRelapseFree_AllMethods_Freq_4 | trial_5 | 0.574114 | 0.539694 | 0.528744 | 0.511755 | 0.538607 | 0.510749 |
| GSE31210.SurvRelapseFree_AllMethods_Freq_5 | trial_5 | 0.58491 | 0.560773 | 0.551809 | 0.53491 | 0.585567 | 0.54449 |
| GSE31210.SurvRelapseFree_AllMethods_Freq_6 | trial_5 | 0.616407 | 0.589375 | 0.573443 | 0.578947 | 0.594835 | 0.578634 |
| GSE31210.SurvRelapseFree_AllMethods_Freq_2 | trial_6 | 0.568079 | 0.545359 | 0.525666 | 0.494754 | 0.531195 | 0.500807 |
| GSE31210.SurvRelapseFree_AllMethods_Freq_3 | trial_6 | 0.578505 | 0.55239 | 0.529293 | 0.509939 | 0.537422 | 0.502575 |
| GSE31210.SurvRelapseFree_AllMethods_Freq_4 | trial_6 | 0.575805 | 0.539694 | 0.528744 | 0.511755 | 0.538607 | 0.510749 |
| GSE31210.SurvRelapseFree_AllMethods_Freq_5 | trial_6 | 0.586513 | 0.560773 | 0.551809 | 0.53491 | 0.585567 | 0.54449 |
| GSE31210.SurvRelapseFree_AllMethods_Freq_6 | trial_6 | 0.617123 | 0.589375 | 0.573443 | 0.578947 | 0.594835 | 0.578634 |
| GSE31210.SurvRelapseFree_AllMethods_Freq_2 | trial_7 | 0.567837 | 0.545359 | 0.525666 | 0.494754 | 0.531195 | 0.500807 |
| GSE31210.SurvRelapseFree_AllMethods_Freq_3 | trial_7 | 0.577982 | 0.55239 | 0.529293 | 0.509939 | 0.537422 | 0.502575 |
| GSE31210.SurvRelapseFree_AllMethods_Freq_4 | trial_7 | 0.573792 | 0.539694 | 0.528744 | 0.511755 | 0.538607 | 0.510749 |
| GSE31210.SurvRelapseFree_AllMethods_Freq_5 | trial_7 | 0.583347 | 0.560773 | 0.551809 | 0.53491 | 0.585567 | 0.54449 |
| GSE31210.SurvRelapseFree_AllMethods_Freq_6 | trial_7 | 0.615154 | 0.589375 | 0.573443 | 0.578947 | 0.594835 | 0.578634 |
| GSE31210.SurvRelapseFree_AllMethods_Freq_2 | trial_8 | 0.566425 | 0.545359 | 0.525666 | 0.494754 | 0.531195 | 0.500807 |
| GSE31210.SurvRelapseFree_AllMethods_Freq_3 | trial_8 | 0.576614 | 0.55239 | 0.529293 | 0.509939 | 0.537422 | 0.502575 |
| GSE31210.SurvRelapseFree_AllMethods_Freq_4 | trial_8 | 0.572343 | 0.539694 | 0.528744 | 0.511755 | 0.538607 | 0.510749 |
| GSE31210.SurvRelapseFree_AllMethods_Freq_5 | trial_8 | 0.584005 | 0.560773 | 0.551809 | 0.53491 | 0.585567 | 0.54449 |
| GSE31210.SurvRelapseFree_AllMethods_Freq_6 | trial_8 | 0.615064 | 0.589375 | 0.573443 | 0.578947 | 0.594835 | 0.578634 |
| GSE31210.SurvRelapseFree_AllMethods_Freq_2 | trial_9 | 0.569169 | 0.545359 | 0.525666 | 0.494754 | 0.531195 | 0.500807 |
| GSE31210.SurvRelapseFree_AllMethods_Freq_3 | trial_9 | 0.579913 | 0.55239 | 0.529293 | 0.509939 | 0.537422 | 0.502575 |
| GSE31210.SurvRelapseFree_AllMethods_Freq_4 | trial_9 | 0.575926 | 0.539694 | 0.528744 | 0.511755 | 0.538607 | 0.510749 |
| GSE31210.SurvRelapseFree_AllMethods_Freq_5 | trial_9 | 0.585362 | 0.560773 | 0.551809 | 0.53491 | 0.585567 | 0.54449 |
| GSE31210.SurvRelapseFree_AllMethods_Freq_6 | trial_9 | 0.616318 | 0.589375 | 0.573443 | 0.578947 | 0.594835 | 0.578634 |
| GSE31210.SurvRelapseFree_AllMethods_Freq_2 | trial_10 | 0.568483 | 0.545359 | 0.525666 | 0.494754 | 0.531195 | 0.500807 |
| GSE31210.SurvRelapseFree_AllMethods_Freq_3 | trial_10 | 0.578143 | 0.55239 | 0.529293 | 0.509939 | 0.537422 | 0.502575 |
| GSE31210.SurvRelapseFree_AllMethods_Freq_4 | trial_10 | 0.574316 | 0.539694 | 0.528744 | 0.511755 | 0.538607 | 0.510749 |
| GSE31210.SurvRelapseFree_AllMethods_Freq_5 | trial_10 | 0.584005 | 0.560773 | 0.551809 | 0.53491 | 0.585567 | 0.54449 |
| GSE31210.SurvRelapseFree_AllMethods_Freq_6 | trial_10 | 0.614572 | 0.589375 | 0.573443 | 0.578947 | 0.594835 | 0.578634 |
| GSE31210.SurvRelapseFree_AllMethods_Freq_2 | trial_11 | 0.56937 | 0.545359 | 0.525666 | 0.494754 | 0.531195 | 0.500807 |
| GSE31210.SurvRelapseFree_AllMethods_Freq_3 | trial_11 | 0.579953 | 0.55239 | 0.529293 | 0.509939 | 0.537422 | 0.502575 |
| GSE31210.SurvRelapseFree_AllMethods_Freq_4 | trial_11 | 0.577134 | 0.539694 | 0.528744 | 0.511755 | 0.538607 | 0.510749 |
| GSE31210.SurvRelapseFree_AllMethods_Freq_5 | trial_11 | 0.587788 | 0.560773 | 0.551809 | 0.53491 | 0.585567 | 0.54449 |
| GSE31210.SurvRelapseFree_AllMethods_Freq_6 | trial_11 | 0.619048 | 0.589375 | 0.573443 | 0.578947 | 0.594835 | 0.578634 |
| GSE31210.SurvRelapseFree_AllMethods_Freq_2 | trial_12 | 0.568523 | 0.545359 | 0.525666 | 0.494754 | 0.531195 | 0.500807 |
| GSE31210.SurvRelapseFree_AllMethods_Freq_3 | trial_12 | 0.579028 | 0.55239 | 0.529293 | 0.509939 | 0.537422 | 0.502575 |
| GSE31210.SurvRelapseFree_AllMethods_Freq_4 | trial_12 | 0.574799 | 0.539694 | 0.528744 | 0.511755 | 0.538607 | 0.510749 |
| GSE31210.SurvRelapseFree_AllMethods_Freq_5 | trial_12 | 0.585321 | 0.560773 | 0.551809 | 0.53491 | 0.585567 | 0.54449 |
| GSE31210.SurvRelapseFree_AllMethods_Freq_6 | trial_12 | 0.614841 | 0.589375 | 0.573443 | 0.578947 | 0.594835 | 0.578634 |
| GSE31210.SurvRelapseFree_AllMethods_Freq_2 | trial_13 | 0.565456 | 0.545359 | 0.525666 | 0.494754 | 0.531195 | 0.500807 |
| GSE31210.SurvRelapseFree_AllMethods_Freq_3 | trial_13 | 0.575769 | 0.55239 | 0.529293 | 0.509939 | 0.537422 | 0.502575 |
| GSE31210.SurvRelapseFree_AllMethods_Freq_4 | trial_13 | 0.572303 | 0.539694 | 0.528744 | 0.511755 | 0.538607 | 0.510749 |
| GSE31210.SurvRelapseFree_AllMethods_Freq_5 | trial_13 | 0.582607 | 0.560773 | 0.551809 | 0.53491 | 0.585567 | 0.54449 |
| GSE31210.SurvRelapseFree_AllMethods_Freq_6 | trial_13 | 0.614662 | 0.589375 | 0.573443 | 0.578947 | 0.594835 | 0.578634 |
| GSE31210.SurvRelapseFree_AllMethods_Freq_2 | trial_14 | 0.568039 | 0.545359 | 0.525666 | 0.494754 | 0.531195 | 0.500807 |
| GSE31210.SurvRelapseFree_AllMethods_Freq_3 | trial_14 | 0.578545 | 0.55239 | 0.529293 | 0.509939 | 0.537422 | 0.502575 |
| GSE31210.SurvRelapseFree_AllMethods_Freq_4 | trial_14 | 0.57496 | 0.539694 | 0.528744 | 0.511755 | 0.538607 | 0.510749 |
| GSE31210.SurvRelapseFree_AllMethods_Freq_5 | trial_14 | 0.584992 | 0.560773 | 0.551809 | 0.53491 | 0.585567 | 0.54449 |
| GSE31210.SurvRelapseFree_AllMethods_Freq_6 | trial_14 | 0.614796 | 0.589375 | 0.573443 | 0.578947 | 0.594835 | 0.578634 |
| GSE31210.SurvRelapseFree_AllMethods_Freq_2 | trial_15 | 0.566586 | 0.545359 | 0.525666 | 0.494754 | 0.531195 | 0.500807 |
| GSE31210.SurvRelapseFree_AllMethods_Freq_3 | trial_15 | 0.576412 | 0.55239 | 0.529293 | 0.509939 | 0.537422 | 0.502575 |
| GSE31210.SurvRelapseFree_AllMethods_Freq_4 | trial_15 | 0.573349 | 0.539694 | 0.528744 | 0.511755 | 0.538607 | 0.510749 |
| GSE31210.SurvRelapseFree_AllMethods_Freq_5 | trial_15 | 0.583059 | 0.560773 | 0.551809 | 0.53491 | 0.585567 | 0.54449 |
| GSE31210.SurvRelapseFree_AllMethods_Freq_6 | trial_15 | 0.613767 | 0.589375 | 0.573443 | 0.578947 | 0.594835 | 0.578634 |
| GSE31210.SurvRelapseFree_AllMethods_Freq_2 | trial_16 | 0.56816 | 0.545359 | 0.525666 | 0.494754 | 0.531195 | 0.500807 |
| GSE31210.SurvRelapseFree_AllMethods_Freq_3 | trial_16 | 0.578545 | 0.55239 | 0.529293 | 0.509939 | 0.537422 | 0.502575 |
| GSE31210.SurvRelapseFree_AllMethods_Freq_4 | trial_16 | 0.574638 | 0.539694 | 0.528744 | 0.511755 | 0.538607 | 0.510749 |
| GSE31210.SurvRelapseFree_AllMethods_Freq_5 | trial_16 | 0.584992 | 0.560773 | 0.551809 | 0.53491 | 0.585567 | 0.54449 |
| GSE31210.SurvRelapseFree_AllMethods_Freq_6 | trial_16 | 0.616541 | 0.589375 | 0.573443 | 0.578947 | 0.594835 | 0.578634 |
| GSE31210.SurvRelapseFree_AllMethods_Freq_2 | trial_17 | 0.56929 | 0.545359 | 0.525666 | 0.494754 | 0.531195 | 0.500807 |
| GSE31210.SurvRelapseFree_AllMethods_Freq_3 | trial_17 | 0.579229 | 0.55239 | 0.529293 | 0.509939 | 0.537422 | 0.502575 |
| GSE31210.SurvRelapseFree_AllMethods_Freq_4 | trial_17 | 0.57649 | 0.539694 | 0.528744 | 0.511755 | 0.538607 | 0.510749 |
| GSE31210.SurvRelapseFree_AllMethods_Freq_5 | trial_17 | 0.586637 | 0.560773 | 0.551809 | 0.53491 | 0.585567 | 0.54449 |
| GSE31210.SurvRelapseFree_AllMethods_Freq_6 | trial_17 | 0.61672 | 0.589375 | 0.573443 | 0.578947 | 0.594835 | 0.578634 |
| GSE31210.SurvRelapseFree_AllMethods_Freq_2 | trial_18 | 0.567111 | 0.545359 | 0.525666 | 0.494754 | 0.531195 | 0.500807 |
| GSE31210.SurvRelapseFree_AllMethods_Freq_3 | trial_18 | 0.577298 | 0.55239 | 0.529293 | 0.509939 | 0.537422 | 0.502575 |
| GSE31210.SurvRelapseFree_AllMethods_Freq_4 | trial_18 | 0.573108 | 0.539694 | 0.528744 | 0.511755 | 0.538607 | 0.510749 |
| GSE31210.SurvRelapseFree_AllMethods_Freq_5 | trial_18 | 0.585115 | 0.560773 | 0.551809 | 0.53491 | 0.585567 | 0.54449 |
| GSE31210.SurvRelapseFree_AllMethods_Freq_6 | trial_18 | 0.616228 | 0.589375 | 0.573443 | 0.578947 | 0.594835 | 0.578634 |
| GSE31210.SurvRelapseFree_AllMethods_Freq_2 | trial_19 | 0.565981 | 0.545359 | 0.525666 | 0.494754 | 0.531195 | 0.500807 |
| GSE31210.SurvRelapseFree_AllMethods_Freq_3 | trial_19 | 0.576654 | 0.55239 | 0.529293 | 0.509939 | 0.537422 | 0.502575 |
| GSE31210.SurvRelapseFree_AllMethods_Freq_4 | trial_19 | 0.573873 | 0.539694 | 0.528744 | 0.511755 | 0.538607 | 0.510749 |
| GSE31210.SurvRelapseFree_AllMethods_Freq_5 | trial_19 | 0.583429 | 0.560773 | 0.551809 | 0.53491 | 0.585567 | 0.54449 |
| GSE31210.SurvRelapseFree_AllMethods_Freq_6 | trial_19 | 0.614125 | 0.589375 | 0.573443 | 0.578947 | 0.594835 | 0.578634 |
| GSE31210.SurvRelapseFree_AllMethods_Freq_2 | trial_20 | 0.567474 | 0.545359 | 0.525666 | 0.494754 | 0.531195 | 0.500807 |
| GSE31210.SurvRelapseFree_AllMethods_Freq_3 | trial_20 | 0.577941 | 0.55239 | 0.529293 | 0.509939 | 0.537422 | 0.502575 |
| GSE31210.SurvRelapseFree_AllMethods_Freq_4 | trial_20 | 0.574074 | 0.539694 | 0.528744 | 0.511755 | 0.538607 | 0.510749 |
| GSE31210.SurvRelapseFree_AllMethods_Freq_5 | trial_20 | 0.584046 | 0.560773 | 0.551809 | 0.53491 | 0.585567 | 0.54449 |
| GSE31210.SurvRelapseFree_AllMethods_Freq_6 | trial_20 | 0.614841 | 0.589375 | 0.573443 | 0.578947 | 0.594835 | 0.578634 |
| GSE31210.SurvRelapseFree_AllMethods_Freq_2 | trial_21 | 0.565052 | 0.545359 | 0.525666 | 0.494754 | 0.531195 | 0.500807 |
| GSE31210.SurvRelapseFree_AllMethods_Freq_3 | trial_21 | 0.575205 | 0.55239 | 0.529293 | 0.509939 | 0.537422 | 0.502575 |
| GSE31210.SurvRelapseFree_AllMethods_Freq_4 | trial_21 | 0.572262 | 0.539694 | 0.528744 | 0.511755 | 0.538607 | 0.510749 |
| GSE31210.SurvRelapseFree_AllMethods_Freq_5 | trial_21 | 0.582155 | 0.560773 | 0.551809 | 0.53491 | 0.585567 | 0.54449 |
| GSE31210.SurvRelapseFree_AllMethods_Freq_6 | trial_21 | 0.61399 | 0.589375 | 0.573443 | 0.578947 | 0.594835 | 0.578634 |
| GSE31210.SurvRelapseFree_AllMethods_Freq_2 | trial_22 | 0.566586 | 0.545359 | 0.525666 | 0.494754 | 0.531195 | 0.500807 |
| GSE31210.SurvRelapseFree_AllMethods_Freq_3 | trial_22 | 0.576694 | 0.55239 | 0.529293 | 0.509939 | 0.537422 | 0.502575 |
| GSE31210.SurvRelapseFree_AllMethods_Freq_4 | trial_22 | 0.574074 | 0.539694 | 0.528744 | 0.511755 | 0.538607 | 0.510749 |
| GSE31210.SurvRelapseFree_AllMethods_Freq_5 | trial_22 | 0.583512 | 0.560773 | 0.551809 | 0.53491 | 0.585567 | 0.54449 |
| GSE31210.SurvRelapseFree_AllMethods_Freq_6 | trial_22 | 0.612513 | 0.589375 | 0.573443 | 0.578947 | 0.594835 | 0.578634 |
| GSE31210.SurvRelapseFree_AllMethods_Freq_2 | trial_23 | 0.566303 | 0.545359 | 0.525666 | 0.494754 | 0.531195 | 0.500807 |
| GSE31210.SurvRelapseFree_AllMethods_Freq_3 | trial_23 | 0.576694 | 0.55239 | 0.529293 | 0.509939 | 0.537422 | 0.502575 |
| GSE31210.SurvRelapseFree_AllMethods_Freq_4 | trial_23 | 0.573953 | 0.539694 | 0.528744 | 0.511755 | 0.538607 | 0.510749 |
| GSE31210.SurvRelapseFree_AllMethods_Freq_5 | trial_23 | 0.584416 | 0.560773 | 0.551809 | 0.53491 | 0.585567 | 0.54449 |
| GSE31210.SurvRelapseFree_AllMethods_Freq_6 | trial_23 | 0.617078 | 0.589375 | 0.573443 | 0.578947 | 0.594835 | 0.578634 |
| GSE31210.SurvRelapseFree_AllMethods_Freq_2 | trial_24 | 0.5659 | 0.545359 | 0.525666 | 0.494754 | 0.531195 | 0.500807 |
| GSE31210.SurvRelapseFree_AllMethods_Freq_3 | trial_24 | 0.576332 | 0.55239 | 0.529293 | 0.509939 | 0.537422 | 0.502575 |
| GSE31210.SurvRelapseFree_AllMethods_Freq_4 | trial_24 | 0.572424 | 0.539694 | 0.528744 | 0.511755 | 0.538607 | 0.510749 |
| GSE31210.SurvRelapseFree_AllMethods_Freq_5 | trial_24 | 0.583018 | 0.560773 | 0.551809 | 0.53491 | 0.585567 | 0.54449 |
| GSE31210.SurvRelapseFree_AllMethods_Freq_6 | trial_24 | 0.614169 | 0.589375 | 0.573443 | 0.578947 | 0.594835 | 0.578634 |
| GSE31210.SurvRelapseFree_AllMethods_Freq_2 | trial_25 | 0.566465 | 0.545359 | 0.525666 | 0.494754 | 0.531195 | 0.500807 |
| GSE31210.SurvRelapseFree_AllMethods_Freq_3 | trial_25 | 0.577338 | 0.55239 | 0.529293 | 0.509939 | 0.537422 | 0.502575 |
| GSE31210.SurvRelapseFree_AllMethods_Freq_4 | trial_25 | 0.573752 | 0.539694 | 0.528744 | 0.511755 | 0.538607 | 0.510749 |
| GSE31210.SurvRelapseFree_AllMethods_Freq_5 | trial_25 | 0.584169 | 0.560773 | 0.551809 | 0.53491 | 0.585567 | 0.54449 |
| GSE31210.SurvRelapseFree_AllMethods_Freq_6 | trial_25 | 0.615781 | 0.589375 | 0.573443 | 0.578947 | 0.594835 | 0.578634 |
| GSE31210.SurvRelapseFree_AllMethods_Freq_2 | trial_26 | 0.569613 | 0.545359 | 0.525666 | 0.494754 | 0.531195 | 0.500807 |
| GSE31210.SurvRelapseFree_AllMethods_Freq_3 | trial_26 | 0.579994 | 0.55239 | 0.529293 | 0.509939 | 0.537422 | 0.502575 |
| GSE31210.SurvRelapseFree_AllMethods_Freq_4 | trial_26 | 0.575322 | 0.539694 | 0.528744 | 0.511755 | 0.538607 | 0.510749 |
| GSE31210.SurvRelapseFree_AllMethods_Freq_5 | trial_26 | 0.584622 | 0.560773 | 0.551809 | 0.53491 | 0.585567 | 0.54449 |
| GSE31210.SurvRelapseFree_AllMethods_Freq_6 | trial_26 | 0.615646 | 0.589375 | 0.573443 | 0.578947 | 0.594835 | 0.578634 |
| GSE31210.SurvRelapseFree_AllMethods_Freq_2 | trial_27 | 0.564528 | 0.545359 | 0.525666 | 0.494754 | 0.531195 | 0.500807 |
| GSE31210.SurvRelapseFree_AllMethods_Freq_3 | trial_27 | 0.574038 | 0.55239 | 0.529293 | 0.509939 | 0.537422 | 0.502575 |
| GSE31210.SurvRelapseFree_AllMethods_Freq_4 | trial_27 | 0.570733 | 0.539694 | 0.528744 | 0.511755 | 0.538607 | 0.510749 |
| GSE31210.SurvRelapseFree_AllMethods_Freq_5 | trial_27 | 0.580428 | 0.560773 | 0.551809 | 0.53491 | 0.585567 | 0.54449 |
| GSE31210.SurvRelapseFree_AllMethods_Freq_6 | trial_27 | 0.612782 | 0.589375 | 0.573443 | 0.578947 | 0.594835 | 0.578634 |
| GSE31210.SurvRelapseFree_AllMethods_Freq_2 | trial_28 | 0.563882 | 0.545359 | 0.525666 | 0.494754 | 0.531195 | 0.500807 |
| GSE31210.SurvRelapseFree_AllMethods_Freq_3 | trial_28 | 0.574119 | 0.55239 | 0.529293 | 0.509939 | 0.537422 | 0.502575 |
| GSE31210.SurvRelapseFree_AllMethods_Freq_4 | trial_28 | 0.571176 | 0.539694 | 0.528744 | 0.511755 | 0.538607 | 0.510749 |
| GSE31210.SurvRelapseFree_AllMethods_Freq_5 | trial_28 | 0.58125 | 0.560773 | 0.551809 | 0.53491 | 0.585567 | 0.54449 |
| GSE31210.SurvRelapseFree_AllMethods_Freq_6 | trial_28 | 0.612692 | 0.589375 | 0.573443 | 0.578947 | 0.594835 | 0.578634 |
| GSE31210.SurvRelapseFree_AllMethods_Freq_2 | trial_29 | 0.568765 | 0.545359 | 0.525666 | 0.494754 | 0.531195 | 0.500807 |
| GSE31210.SurvRelapseFree_AllMethods_Freq_3 | trial_29 | 0.578625 | 0.55239 | 0.529293 | 0.509939 | 0.537422 | 0.502575 |
| GSE31210.SurvRelapseFree_AllMethods_Freq_4 | trial_29 | 0.575966 | 0.539694 | 0.528744 | 0.511755 | 0.538607 | 0.510749 |
| GSE31210.SurvRelapseFree_AllMethods_Freq_5 | trial_29 | 0.585855 | 0.560773 | 0.551809 | 0.53491 | 0.585567 | 0.54449 |
| GSE31210.SurvRelapseFree_AllMethods_Freq_6 | trial_29 | 0.615378 | 0.589375 | 0.573443 | 0.578947 | 0.594835 | 0.578634 |
| GSE31210.SurvRelapseFree_AllMethods_Freq_2 | trial_30 | 0.568321 | 0.545359 | 0.525666 | 0.494754 | 0.531195 | 0.500807 |
| GSE31210.SurvRelapseFree_AllMethods_Freq_3 | trial_30 | 0.577418 | 0.55239 | 0.529293 | 0.509939 | 0.537422 | 0.502575 |
| GSE31210.SurvRelapseFree_AllMethods_Freq_4 | trial_30 | 0.573873 | 0.539694 | 0.528744 | 0.511755 | 0.538607 | 0.510749 |
| GSE31210.SurvRelapseFree_AllMethods_Freq_5 | trial_30 | 0.584211 | 0.560773 | 0.551809 | 0.53491 | 0.585567 | 0.54449 |
| GSE31210.SurvRelapseFree_AllMethods_Freq_6 | trial_30 | 0.614975 | 0.589375 | 0.573443 | 0.578947 | 0.594835 | 0.578634 |
| GSE31210.SurvRelapseFree_AllMethods_Freq_2 | trial_31 | 0.566384 | 0.545359 | 0.525666 | 0.494754 | 0.531195 | 0.500807 |
| GSE31210.SurvRelapseFree_AllMethods_Freq_3 | trial_31 | 0.577056 | 0.55239 | 0.529293 | 0.509939 | 0.537422 | 0.502575 |
| GSE31210.SurvRelapseFree_AllMethods_Freq_4 | trial_31 | 0.57339 | 0.539694 | 0.528744 | 0.511755 | 0.538607 | 0.510749 |
| GSE31210.SurvRelapseFree_AllMethods_Freq_5 | trial_31 | 0.583388 | 0.560773 | 0.551809 | 0.53491 | 0.585567 | 0.54449 |
| GSE31210.SurvRelapseFree_AllMethods_Freq_6 | trial_31 | 0.613319 | 0.589375 | 0.573443 | 0.578947 | 0.594835 | 0.578634 |
| GSE31210.SurvRelapseFree_AllMethods_Freq_2 | trial_32 | 0.56594 | 0.545359 | 0.525666 | 0.494754 | 0.531195 | 0.500807 |
| GSE31210.SurvRelapseFree_AllMethods_Freq_3 | trial_32 | 0.576453 | 0.55239 | 0.529293 | 0.509939 | 0.537422 | 0.502575 |
| GSE31210.SurvRelapseFree_AllMethods_Freq_4 | trial_32 | 0.57347 | 0.539694 | 0.528744 | 0.511755 | 0.538607 | 0.510749 |
| GSE31210.SurvRelapseFree_AllMethods_Freq_5 | trial_32 | 0.582607 | 0.560773 | 0.551809 | 0.53491 | 0.585567 | 0.54449 |
| GSE31210.SurvRelapseFree_AllMethods_Freq_6 | trial_32 | 0.613095 | 0.589375 | 0.573443 | 0.578947 | 0.594835 | 0.578634 |
| GSE31210.SurvRelapseFree_AllMethods_Freq_2 | trial_33 | 0.567474 | 0.545359 | 0.525666 | 0.494754 | 0.531195 | 0.500807 |
| GSE31210.SurvRelapseFree_AllMethods_Freq_3 | trial_33 | 0.578183 | 0.55239 | 0.529293 | 0.509939 | 0.537422 | 0.502575 |
| GSE31210.SurvRelapseFree_AllMethods_Freq_4 | trial_33 | 0.575443 | 0.539694 | 0.528744 | 0.511755 | 0.538607 | 0.510749 |
| GSE31210.SurvRelapseFree_AllMethods_Freq_5 | trial_33 | 0.587747 | 0.560773 | 0.551809 | 0.53491 | 0.585567 | 0.54449 |
| GSE31210.SurvRelapseFree_AllMethods_Freq_6 | trial_33 | 0.617526 | 0.589375 | 0.573443 | 0.578947 | 0.594835 | 0.578634 |
| GSE31210.SurvRelapseFree_AllMethods_Freq_2 | trial_34 | 0.568846 | 0.545359 | 0.525666 | 0.494754 | 0.531195 | 0.500807 |
| GSE31210.SurvRelapseFree_AllMethods_Freq_3 | trial_34 | 0.578827 | 0.55239 | 0.529293 | 0.509939 | 0.537422 | 0.502575 |
| GSE31210.SurvRelapseFree_AllMethods_Freq_4 | trial_34 | 0.574517 | 0.539694 | 0.528744 | 0.511755 | 0.538607 | 0.510749 |
| GSE31210.SurvRelapseFree_AllMethods_Freq_5 | trial_34 | 0.584046 | 0.560773 | 0.551809 | 0.53491 | 0.585567 | 0.54449 |
| GSE31210.SurvRelapseFree_AllMethods_Freq_6 | trial_34 | 0.613722 | 0.589375 | 0.573443 | 0.578947 | 0.594835 | 0.578634 |
| GSE31210.SurvRelapseFree_AllMethods_Freq_2 | trial_35 | 0.569814 | 0.545359 | 0.525666 | 0.494754 | 0.531195 | 0.500807 |
| GSE31210.SurvRelapseFree_AllMethods_Freq_3 | trial_35 | 0.579953 | 0.55239 | 0.529293 | 0.509939 | 0.537422 | 0.502575 |
| GSE31210.SurvRelapseFree_AllMethods_Freq_4 | trial_35 | 0.575886 | 0.539694 | 0.528744 | 0.511755 | 0.538607 | 0.510749 |
| GSE31210.SurvRelapseFree_AllMethods_Freq_5 | trial_35 | 0.585156 | 0.560773 | 0.551809 | 0.53491 | 0.585567 | 0.54449 |
| GSE31210.SurvRelapseFree_AllMethods_Freq_6 | trial_35 | 0.616452 | 0.589375 | 0.573443 | 0.578947 | 0.594835 | 0.578634 |
| GSE31210.SurvRelapseFree_AllMethods_Freq_2 | trial_36 | 0.566182 | 0.545359 | 0.525666 | 0.494754 | 0.531195 | 0.500807 |
| GSE31210.SurvRelapseFree_AllMethods_Freq_3 | trial_36 | 0.576131 | 0.55239 | 0.529293 | 0.509939 | 0.537422 | 0.502575 |
| GSE31210.SurvRelapseFree_AllMethods_Freq_4 | trial_36 | 0.57347 | 0.539694 | 0.528744 | 0.511755 | 0.538607 | 0.510749 |
| GSE31210.SurvRelapseFree_AllMethods_Freq_5 | trial_36 | 0.58602 | 0.560773 | 0.551809 | 0.53491 | 0.585567 | 0.54449 |
| GSE31210.SurvRelapseFree_AllMethods_Freq_6 | trial_36 | 0.619048 | 0.589375 | 0.573443 | 0.578947 | 0.594835 | 0.578634 |
| GSE31210.SurvRelapseFree_AllMethods_Freq_2 | trial_37 | 0.566707 | 0.545359 | 0.525666 | 0.494754 | 0.531195 | 0.500807 |
| GSE31210.SurvRelapseFree_AllMethods_Freq_3 | trial_37 | 0.577177 | 0.55239 | 0.529293 | 0.509939 | 0.537422 | 0.502575 |
| GSE31210.SurvRelapseFree_AllMethods_Freq_4 | trial_37 | 0.572826 | 0.539694 | 0.528744 | 0.511755 | 0.538607 | 0.510749 |
| GSE31210.SurvRelapseFree_AllMethods_Freq_5 | trial_37 | 0.581908 | 0.560773 | 0.551809 | 0.53491 | 0.585567 | 0.54449 |
| GSE31210.SurvRelapseFree_AllMethods_Freq_6 | trial_37 | 0.612737 | 0.589375 | 0.573443 | 0.578947 | 0.594835 | 0.578634 |
| GSE31210.SurvRelapseFree_AllMethods_Freq_2 | trial_38 | 0.566344 | 0.545359 | 0.525666 | 0.494754 | 0.531195 | 0.500807 |
| GSE31210.SurvRelapseFree_AllMethods_Freq_3 | trial_38 | 0.576694 | 0.55239 | 0.529293 | 0.509939 | 0.537422 | 0.502575 |
| GSE31210.SurvRelapseFree_AllMethods_Freq_4 | trial_38 | 0.573551 | 0.539694 | 0.528744 | 0.511755 | 0.538607 | 0.510749 |
| GSE31210.SurvRelapseFree_AllMethods_Freq_5 | trial_38 | 0.583964 | 0.560773 | 0.551809 | 0.53491 | 0.585567 | 0.54449 |
| GSE31210.SurvRelapseFree_AllMethods_Freq_6 | trial_38 | 0.613543 | 0.589375 | 0.573443 | 0.578947 | 0.594835 | 0.578634 |
| GSE31210.SurvRelapseFree_AllMethods_Freq_2 | trial_39 | 0.563842 | 0.545359 | 0.525666 | 0.494754 | 0.531195 | 0.500807 |
| GSE31210.SurvRelapseFree_AllMethods_Freq_3 | trial_39 | 0.5744 | 0.55239 | 0.529293 | 0.509939 | 0.537422 | 0.502575 |
| GSE31210.SurvRelapseFree_AllMethods_Freq_4 | trial_39 | 0.570974 | 0.539694 | 0.528744 | 0.511755 | 0.538607 | 0.510749 |
| GSE31210.SurvRelapseFree_AllMethods_Freq_5 | trial_39 | 0.58088 | 0.560773 | 0.551809 | 0.53491 | 0.585567 | 0.54449 |
| GSE31210.SurvRelapseFree_AllMethods_Freq_6 | trial_39 | 0.6122 | 0.589375 | 0.573443 | 0.578947 | 0.594835 | 0.578634 |
| GSE31210.SurvRelapseFree_AllMethods_Freq_2 | trial_40 | 0.565214 | 0.545359 | 0.525666 | 0.494754 | 0.531195 | 0.500807 |
| GSE31210.SurvRelapseFree_AllMethods_Freq_3 | trial_40 | 0.575688 | 0.55239 | 0.529293 | 0.509939 | 0.537422 | 0.502575 |
| GSE31210.SurvRelapseFree_AllMethods_Freq_4 | trial_40 | 0.571457 | 0.539694 | 0.528744 | 0.511755 | 0.538607 | 0.510749 |
| GSE31210.SurvRelapseFree_AllMethods_Freq_5 | trial_40 | 0.581785 | 0.560773 | 0.551809 | 0.53491 | 0.585567 | 0.54449 |
| GSE31210.SurvRelapseFree_AllMethods_Freq_6 | trial_40 | 0.613767 | 0.589375 | 0.573443 | 0.578947 | 0.594835 | 0.578634 |
| GSE31210.SurvRelapseFree_AllMethods_Freq_2 | trial_41 | 0.568563 | 0.545359 | 0.525666 | 0.494754 | 0.531195 | 0.500807 |
| GSE31210.SurvRelapseFree_AllMethods_Freq_3 | trial_41 | 0.578746 | 0.55239 | 0.529293 | 0.509939 | 0.537422 | 0.502575 |
| GSE31210.SurvRelapseFree_AllMethods_Freq_4 | trial_41 | 0.575523 | 0.539694 | 0.528744 | 0.511755 | 0.538607 | 0.510749 |
| GSE31210.SurvRelapseFree_AllMethods_Freq_5 | trial_41 | 0.587212 | 0.560773 | 0.551809 | 0.53491 | 0.585567 | 0.54449 |
| GSE31210.SurvRelapseFree_AllMethods_Freq_6 | trial_41 | 0.617929 | 0.589375 | 0.573443 | 0.578947 | 0.594835 | 0.578634 |
| GSE31210.SurvRelapseFree_AllMethods_Freq_2 | trial_42 | 0.567353 | 0.545359 | 0.525666 | 0.494754 | 0.531195 | 0.500807 |
| GSE31210.SurvRelapseFree_AllMethods_Freq_3 | trial_42 | 0.577338 | 0.55239 | 0.529293 | 0.509939 | 0.537422 | 0.502575 |
| GSE31210.SurvRelapseFree_AllMethods_Freq_4 | trial_42 | 0.572826 | 0.539694 | 0.528744 | 0.511755 | 0.538607 | 0.510749 |
| GSE31210.SurvRelapseFree_AllMethods_Freq_5 | trial_42 | 0.583018 | 0.560773 | 0.551809 | 0.53491 | 0.585567 | 0.54449 |
| GSE31210.SurvRelapseFree_AllMethods_Freq_6 | trial_42 | 0.612021 | 0.589375 | 0.573443 | 0.578947 | 0.594835 | 0.578634 |
| GSE31210.SurvRelapseFree_AllMethods_Freq_2 | trial_43 | 0.565698 | 0.545359 | 0.525666 | 0.494754 | 0.531195 | 0.500807 |
| GSE31210.SurvRelapseFree_AllMethods_Freq_3 | trial_43 | 0.576171 | 0.55239 | 0.529293 | 0.509939 | 0.537422 | 0.502575 |
| GSE31210.SurvRelapseFree_AllMethods_Freq_4 | trial_43 | 0.573108 | 0.539694 | 0.528744 | 0.511755 | 0.538607 | 0.510749 |
| GSE31210.SurvRelapseFree_AllMethods_Freq_5 | trial_43 | 0.582977 | 0.560773 | 0.551809 | 0.53491 | 0.585567 | 0.54449 |
| GSE31210.SurvRelapseFree_AllMethods_Freq_6 | trial_43 | 0.611797 | 0.589375 | 0.573443 | 0.578947 | 0.594835 | 0.578634 |
| GSE31210.SurvRelapseFree_AllMethods_Freq_2 | trial_44 | 0.570016 | 0.545359 | 0.525666 | 0.494754 | 0.531195 | 0.500807 |
| GSE31210.SurvRelapseFree_AllMethods_Freq_3 | trial_44 | 0.58112 | 0.55239 | 0.529293 | 0.509939 | 0.537422 | 0.502575 |
| GSE31210.SurvRelapseFree_AllMethods_Freq_4 | trial_44 | 0.576973 | 0.539694 | 0.528744 | 0.511755 | 0.538607 | 0.510749 |
| GSE31210.SurvRelapseFree_AllMethods_Freq_5 | trial_44 | 0.587336 | 0.560773 | 0.551809 | 0.53491 | 0.585567 | 0.54449 |
| GSE31210.SurvRelapseFree_AllMethods_Freq_6 | trial_44 | 0.617213 | 0.589375 | 0.573443 | 0.578947 | 0.594835 | 0.578634 |
| GSE31210.SurvRelapseFree_AllMethods_Freq_2 | trial_45 | 0.566747 | 0.545359 | 0.525666 | 0.494754 | 0.531195 | 0.500807 |
| GSE31210.SurvRelapseFree_AllMethods_Freq_3 | trial_45 | 0.577056 | 0.55239 | 0.529293 | 0.509939 | 0.537422 | 0.502575 |
| GSE31210.SurvRelapseFree_AllMethods_Freq_4 | trial_45 | 0.573188 | 0.539694 | 0.528744 | 0.511755 | 0.538607 | 0.510749 |
| GSE31210.SurvRelapseFree_AllMethods_Freq_5 | trial_45 | 0.582689 | 0.560773 | 0.551809 | 0.53491 | 0.585567 | 0.54449 |
| GSE31210.SurvRelapseFree_AllMethods_Freq_6 | trial_45 | 0.612469 | 0.589375 | 0.573443 | 0.578947 | 0.594835 | 0.578634 |
| GSE31210.SurvRelapseFree_AllMethods_Freq_2 | trial_46 | 0.568563 | 0.545359 | 0.525666 | 0.494754 | 0.531195 | 0.500807 |
| GSE31210.SurvRelapseFree_AllMethods_Freq_3 | trial_46 | 0.578827 | 0.55239 | 0.529293 | 0.509939 | 0.537422 | 0.502575 |
| GSE31210.SurvRelapseFree_AllMethods_Freq_4 | trial_46 | 0.575081 | 0.539694 | 0.528744 | 0.511755 | 0.538607 | 0.510749 |
| GSE31210.SurvRelapseFree_AllMethods_Freq_5 | trial_46 | 0.584581 | 0.560773 | 0.551809 | 0.53491 | 0.585567 | 0.54449 |
| GSE31210.SurvRelapseFree_AllMethods_Freq_6 | trial_46 | 0.613856 | 0.589375 | 0.573443 | 0.578947 | 0.594835 | 0.578634 |
| GSE31210.SurvRelapseFree_AllMethods_Freq_2 | trial_47 | 0.564528 | 0.545359 | 0.525666 | 0.494754 | 0.531195 | 0.500807 |
| GSE31210.SurvRelapseFree_AllMethods_Freq_3 | trial_47 | 0.575326 | 0.55239 | 0.529293 | 0.509939 | 0.537422 | 0.502575 |
| GSE31210.SurvRelapseFree_AllMethods_Freq_4 | trial_47 | 0.572182 | 0.539694 | 0.528744 | 0.511755 | 0.538607 | 0.510749 |
| GSE31210.SurvRelapseFree_AllMethods_Freq_5 | trial_47 | 0.584252 | 0.560773 | 0.551809 | 0.53491 | 0.585567 | 0.54449 |
| GSE31210.SurvRelapseFree_AllMethods_Freq_6 | trial_47 | 0.615378 | 0.589375 | 0.573443 | 0.578947 | 0.594835 | 0.578634 |
| GSE31210.SurvRelapseFree_AllMethods_Freq_2 | trial_48 | 0.567998 | 0.545359 | 0.525666 | 0.494754 | 0.531195 | 0.500807 |
| GSE31210.SurvRelapseFree_AllMethods_Freq_3 | trial_48 | 0.578102 | 0.55239 | 0.529293 | 0.509939 | 0.537422 | 0.502575 |
| GSE31210.SurvRelapseFree_AllMethods_Freq_4 | trial_48 | 0.574235 | 0.539694 | 0.528744 | 0.511755 | 0.538607 | 0.510749 |
| GSE31210.SurvRelapseFree_AllMethods_Freq_5 | trial_48 | 0.585238 | 0.560773 | 0.551809 | 0.53491 | 0.585567 | 0.54449 |
| GSE31210.SurvRelapseFree_AllMethods_Freq_6 | trial_48 | 0.616273 | 0.589375 | 0.573443 | 0.578947 | 0.594835 | 0.578634 |
| GSE31210.SurvRelapseFree_AllMethods_Freq_2 | trial_49 | 0.570581 | 0.545359 | 0.525666 | 0.494754 | 0.531195 | 0.500807 |
| GSE31210.SurvRelapseFree_AllMethods_Freq_3 | trial_49 | 0.580678 | 0.55239 | 0.529293 | 0.509939 | 0.537422 | 0.502575 |
| GSE31210.SurvRelapseFree_AllMethods_Freq_4 | trial_49 | 0.577496 | 0.539694 | 0.528744 | 0.511755 | 0.538607 | 0.510749 |
| GSE31210.SurvRelapseFree_AllMethods_Freq_5 | trial_49 | 0.587788 | 0.560773 | 0.551809 | 0.53491 | 0.585567 | 0.54449 |
| GSE31210.SurvRelapseFree_AllMethods_Freq_6 | trial_49 | 0.616049 | 0.589375 | 0.573443 | 0.578947 | 0.594835 | 0.578634 |
| GSE31210.SurvRelapseFree_AllMethods_Freq_2 | trial_50 | 0.568241 | 0.545359 | 0.525666 | 0.494754 | 0.531195 | 0.500807 |
| GSE31210.SurvRelapseFree_AllMethods_Freq_3 | trial_50 | 0.577539 | 0.55239 | 0.529293 | 0.509939 | 0.537422 | 0.502575 |
| GSE31210.SurvRelapseFree_AllMethods_Freq_4 | trial_50 | 0.574356 | 0.539694 | 0.528744 | 0.511755 | 0.538607 | 0.510749 |
| GSE31210.SurvRelapseFree_AllMethods_Freq_5 | trial_50 | 0.584868 | 0.560773 | 0.551809 | 0.53491 | 0.585567 | 0.54449 |
| GSE31210.SurvRelapseFree_AllMethods_Freq_6 | trial_50 | 0.613901 | 0.589375 | 0.573443 | 0.578947 | 0.594835 | 0.578634 |
| GSE31210.SurvRelapseFree_AllMethods_Freq_2 | trial_51 | 0.565496 | 0.545359 | 0.525666 | 0.494754 | 0.531195 | 0.500807 |
| GSE31210.SurvRelapseFree_AllMethods_Freq_3 | trial_51 | 0.57601 | 0.55239 | 0.529293 | 0.509939 | 0.537422 | 0.502575 |
| GSE31210.SurvRelapseFree_AllMethods_Freq_4 | trial_51 | 0.572061 | 0.539694 | 0.528744 | 0.511755 | 0.538607 | 0.510749 |
| GSE31210.SurvRelapseFree_AllMethods_Freq_5 | trial_51 | 0.582278 | 0.560773 | 0.551809 | 0.53491 | 0.585567 | 0.54449 |
| GSE31210.SurvRelapseFree_AllMethods_Freq_6 | trial_51 | 0.614438 | 0.589375 | 0.573443 | 0.578947 | 0.594835 | 0.578634 |
| GSE31210.SurvRelapseFree_AllMethods_Freq_2 | trial_52 | 0.566263 | 0.545359 | 0.525666 | 0.494754 | 0.531195 | 0.500807 |
| GSE31210.SurvRelapseFree_AllMethods_Freq_3 | trial_52 | 0.576171 | 0.55239 | 0.529293 | 0.509939 | 0.537422 | 0.502575 |
| GSE31210.SurvRelapseFree_AllMethods_Freq_4 | trial_52 | 0.572705 | 0.539694 | 0.528744 | 0.511755 | 0.538607 | 0.510749 |
| GSE31210.SurvRelapseFree_AllMethods_Freq_5 | trial_52 | 0.583347 | 0.560773 | 0.551809 | 0.53491 | 0.585567 | 0.54449 |
| GSE31210.SurvRelapseFree_AllMethods_Freq_6 | trial_52 | 0.615378 | 0.589375 | 0.573443 | 0.578947 | 0.594835 | 0.578634 |
| GSE31210.SurvRelapseFree_AllMethods_Freq_2 | trial_53 | 0.56816 | 0.545359 | 0.525666 | 0.494754 | 0.531195 | 0.500807 |
| GSE31210.SurvRelapseFree_AllMethods_Freq_3 | trial_53 | 0.578706 | 0.55239 | 0.529293 | 0.509939 | 0.537422 | 0.502575 |
| GSE31210.SurvRelapseFree_AllMethods_Freq_4 | trial_53 | 0.574638 | 0.539694 | 0.528744 | 0.511755 | 0.538607 | 0.510749 |
| GSE31210.SurvRelapseFree_AllMethods_Freq_5 | trial_53 | 0.584992 | 0.560773 | 0.551809 | 0.53491 | 0.585567 | 0.54449 |
| GSE31210.SurvRelapseFree_AllMethods_Freq_6 | trial_53 | 0.616631 | 0.589375 | 0.573443 | 0.578947 | 0.594835 | 0.578634 |
| GSE31210.SurvRelapseFree_AllMethods_Freq_2 | trial_54 | 0.568967 | 0.545359 | 0.525666 | 0.494754 | 0.531195 | 0.500807 |
| GSE31210.SurvRelapseFree_AllMethods_Freq_3 | trial_54 | 0.57931 | 0.55239 | 0.529293 | 0.509939 | 0.537422 | 0.502575 |
| GSE31210.SurvRelapseFree_AllMethods_Freq_4 | trial_54 | 0.575845 | 0.539694 | 0.528744 | 0.511755 | 0.538607 | 0.510749 |
| GSE31210.SurvRelapseFree_AllMethods_Freq_5 | trial_54 | 0.585732 | 0.560773 | 0.551809 | 0.53491 | 0.585567 | 0.54449 |
| GSE31210.SurvRelapseFree_AllMethods_Freq_6 | trial_54 | 0.616676 | 0.589375 | 0.573443 | 0.578947 | 0.594835 | 0.578634 |
| GSE31210.SurvRelapseFree_AllMethods_Freq_2 | trial_55 | 0.566747 | 0.545359 | 0.525666 | 0.494754 | 0.531195 | 0.500807 |
| GSE31210.SurvRelapseFree_AllMethods_Freq_3 | trial_55 | 0.577177 | 0.55239 | 0.529293 | 0.509939 | 0.537422 | 0.502575 |
| GSE31210.SurvRelapseFree_AllMethods_Freq_4 | trial_55 | 0.572907 | 0.539694 | 0.528744 | 0.511755 | 0.538607 | 0.510749 |
| GSE31210.SurvRelapseFree_AllMethods_Freq_5 | trial_55 | 0.584128 | 0.560773 | 0.551809 | 0.53491 | 0.585567 | 0.54449 |
| GSE31210.SurvRelapseFree_AllMethods_Freq_6 | trial_55 | 0.614617 | 0.589375 | 0.573443 | 0.578947 | 0.594835 | 0.578634 |
| GSE31210.SurvRelapseFree_AllMethods_Freq_2 | trial_56 | 0.567918 | 0.545359 | 0.525666 | 0.494754 | 0.531195 | 0.500807 |
| GSE31210.SurvRelapseFree_AllMethods_Freq_3 | trial_56 | 0.578706 | 0.55239 | 0.529293 | 0.509939 | 0.537422 | 0.502575 |
| GSE31210.SurvRelapseFree_AllMethods_Freq_4 | trial_56 | 0.575242 | 0.539694 | 0.528744 | 0.511755 | 0.538607 | 0.510749 |
| GSE31210.SurvRelapseFree_AllMethods_Freq_5 | trial_56 | 0.584128 | 0.560773 | 0.551809 | 0.53491 | 0.585567 | 0.54449 |
| GSE31210.SurvRelapseFree_AllMethods_Freq_6 | trial_56 | 0.613677 | 0.589375 | 0.573443 | 0.578947 | 0.594835 | 0.578634 |
| GSE31210.SurvRelapseFree_AllMethods_Freq_2 | trial_57 | 0.568684 | 0.545359 | 0.525666 | 0.494754 | 0.531195 | 0.500807 |
| GSE31210.SurvRelapseFree_AllMethods_Freq_3 | trial_57 | 0.578344 | 0.55239 | 0.529293 | 0.509939 | 0.537422 | 0.502575 |
| GSE31210.SurvRelapseFree_AllMethods_Freq_4 | trial_57 | 0.573873 | 0.539694 | 0.528744 | 0.511755 | 0.538607 | 0.510749 |
| GSE31210.SurvRelapseFree_AllMethods_Freq_5 | trial_57 | 0.584622 | 0.560773 | 0.551809 | 0.53491 | 0.585567 | 0.54449 |
| GSE31210.SurvRelapseFree_AllMethods_Freq_6 | trial_57 | 0.615243 | 0.589375 | 0.573443 | 0.578947 | 0.594835 | 0.578634 |
| GSE31210.SurvRelapseFree_AllMethods_Freq_2 | trial_58 | 0.566182 | 0.545359 | 0.525666 | 0.494754 | 0.531195 | 0.500807 |
| GSE31210.SurvRelapseFree_AllMethods_Freq_3 | trial_58 | 0.576412 | 0.55239 | 0.529293 | 0.509939 | 0.537422 | 0.502575 |
| GSE31210.SurvRelapseFree_AllMethods_Freq_4 | trial_58 | 0.573309 | 0.539694 | 0.528744 | 0.511755 | 0.538607 | 0.510749 |
| GSE31210.SurvRelapseFree_AllMethods_Freq_5 | trial_58 | 0.584498 | 0.560773 | 0.551809 | 0.53491 | 0.585567 | 0.54449 |
| GSE31210.SurvRelapseFree_AllMethods_Freq_6 | trial_58 | 0.614035 | 0.589375 | 0.573443 | 0.578947 | 0.594835 | 0.578634 |
| GSE31210.SurvRelapseFree_AllMethods_Freq_2 | trial_59 | 0.571429 | 0.545359 | 0.525666 | 0.494754 | 0.531195 | 0.500807 |
| GSE31210.SurvRelapseFree_AllMethods_Freq_3 | trial_59 | 0.581442 | 0.55239 | 0.529293 | 0.509939 | 0.537422 | 0.502575 |
| GSE31210.SurvRelapseFree_AllMethods_Freq_4 | trial_59 | 0.57806 | 0.539694 | 0.528744 | 0.511755 | 0.538607 | 0.510749 |
| GSE31210.SurvRelapseFree_AllMethods_Freq_5 | trial_59 | 0.586883 | 0.560773 | 0.551809 | 0.53491 | 0.585567 | 0.54449 |
| GSE31210.SurvRelapseFree_AllMethods_Freq_6 | trial_59 | 0.617213 | 0.589375 | 0.573443 | 0.578947 | 0.594835 | 0.578634 |
| GSE31210.SurvRelapseFree_AllMethods_Freq_2 | trial_60 | 0.567918 | 0.545359 | 0.525666 | 0.494754 | 0.531195 | 0.500807 |
| GSE31210.SurvRelapseFree_AllMethods_Freq_3 | trial_60 | 0.577821 | 0.55239 | 0.529293 | 0.509939 | 0.537422 | 0.502575 |
| GSE31210.SurvRelapseFree_AllMethods_Freq_4 | trial_60 | 0.57496 | 0.539694 | 0.528744 | 0.511755 | 0.538607 | 0.510749 |
| GSE31210.SurvRelapseFree_AllMethods_Freq_5 | trial_60 | 0.585074 | 0.560773 | 0.551809 | 0.53491 | 0.585567 | 0.54449 |
| GSE31210.SurvRelapseFree_AllMethods_Freq_6 | trial_60 | 0.616094 | 0.589375 | 0.573443 | 0.578947 | 0.594835 | 0.578634 |
| GSE31210.SurvRelapseFree_AllMethods_Freq_2 | trial_61 | 0.568402 | 0.545359 | 0.525666 | 0.494754 | 0.531195 | 0.500807 |
| GSE31210.SurvRelapseFree_AllMethods_Freq_3 | trial_61 | 0.578947 | 0.55239 | 0.529293 | 0.509939 | 0.537422 | 0.502575 |
| GSE31210.SurvRelapseFree_AllMethods_Freq_4 | trial_61 | 0.575725 | 0.539694 | 0.528744 | 0.511755 | 0.538607 | 0.510749 |
| GSE31210.SurvRelapseFree_AllMethods_Freq_5 | trial_61 | 0.587541 | 0.560773 | 0.551809 | 0.53491 | 0.585567 | 0.54449 |
| GSE31210.SurvRelapseFree_AllMethods_Freq_6 | trial_61 | 0.620435 | 0.589375 | 0.573443 | 0.578947 | 0.594835 | 0.578634 |
| GSE31210.SurvRelapseFree_AllMethods_Freq_2 | trial_62 | 0.565052 | 0.545359 | 0.525666 | 0.494754 | 0.531195 | 0.500807 |
| GSE31210.SurvRelapseFree_AllMethods_Freq_3 | trial_62 | 0.5744 | 0.55239 | 0.529293 | 0.509939 | 0.537422 | 0.502575 |
| GSE31210.SurvRelapseFree_AllMethods_Freq_4 | trial_62 | 0.570531 | 0.539694 | 0.528744 | 0.511755 | 0.538607 | 0.510749 |
| GSE31210.SurvRelapseFree_AllMethods_Freq_5 | trial_62 | 0.580469 | 0.560773 | 0.551809 | 0.53491 | 0.585567 | 0.54449 |
| GSE31210.SurvRelapseFree_AllMethods_Freq_6 | trial_62 | 0.6122 | 0.589375 | 0.573443 | 0.578947 | 0.594835 | 0.578634 |
| GSE31210.SurvRelapseFree_AllMethods_Freq_2 | trial_63 | 0.567514 | 0.545359 | 0.525666 | 0.494754 | 0.531195 | 0.500807 |
| GSE31210.SurvRelapseFree_AllMethods_Freq_3 | trial_63 | 0.577901 | 0.55239 | 0.529293 | 0.509939 | 0.537422 | 0.502575 |
| GSE31210.SurvRelapseFree_AllMethods_Freq_4 | trial_63 | 0.574195 | 0.539694 | 0.528744 | 0.511755 | 0.538607 | 0.510749 |
| GSE31210.SurvRelapseFree_AllMethods_Freq_5 | trial_63 | 0.584128 | 0.560773 | 0.551809 | 0.53491 | 0.585567 | 0.54449 |
| GSE31210.SurvRelapseFree_AllMethods_Freq_6 | trial_63 | 0.61587 | 0.589375 | 0.573443 | 0.578947 | 0.594835 | 0.578634 |
| GSE31210.SurvRelapseFree_AllMethods_Freq_2 | trial_64 | 0.569855 | 0.545359 | 0.525666 | 0.494754 | 0.531195 | 0.500807 |
| GSE31210.SurvRelapseFree_AllMethods_Freq_3 | trial_64 | 0.579631 | 0.55239 | 0.529293 | 0.509939 | 0.537422 | 0.502575 |
| GSE31210.SurvRelapseFree_AllMethods_Freq_4 | trial_64 | 0.577254 | 0.539694 | 0.528744 | 0.511755 | 0.538607 | 0.510749 |
| GSE31210.SurvRelapseFree_AllMethods_Freq_5 | trial_64 | 0.587623 | 0.560773 | 0.551809 | 0.53491 | 0.585567 | 0.54449 |
| GSE31210.SurvRelapseFree_AllMethods_Freq_6 | trial_64 | 0.618242 | 0.589375 | 0.573443 | 0.578947 | 0.594835 | 0.578634 |
| GSE31210.SurvRelapseFree_AllMethods_Freq_2 | trial_65 | 0.566949 | 0.545359 | 0.525666 | 0.494754 | 0.531195 | 0.500807 |
| GSE31210.SurvRelapseFree_AllMethods_Freq_3 | trial_65 | 0.577539 | 0.55239 | 0.529293 | 0.509939 | 0.537422 | 0.502575 |
| GSE31210.SurvRelapseFree_AllMethods_Freq_4 | trial_65 | 0.574919 | 0.539694 | 0.528744 | 0.511755 | 0.538607 | 0.510749 |
| GSE31210.SurvRelapseFree_AllMethods_Freq_5 | trial_65 | 0.585403 | 0.560773 | 0.551809 | 0.53491 | 0.585567 | 0.54449 |
| GSE31210.SurvRelapseFree_AllMethods_Freq_6 | trial_65 | 0.617078 | 0.589375 | 0.573443 | 0.578947 | 0.594835 | 0.578634 |
| GSE31210.SurvRelapseFree_AllMethods_Freq_2 | trial_66 | 0.564487 | 0.545359 | 0.525666 | 0.494754 | 0.531195 | 0.500807 |
| GSE31210.SurvRelapseFree_AllMethods_Freq_3 | trial_66 | 0.574843 | 0.55239 | 0.529293 | 0.509939 | 0.537422 | 0.502575 |
| GSE31210.SurvRelapseFree_AllMethods_Freq_4 | trial_66 | 0.571779 | 0.539694 | 0.528744 | 0.511755 | 0.538607 | 0.510749 |
| GSE31210.SurvRelapseFree_AllMethods_Freq_5 | trial_66 | 0.583347 | 0.560773 | 0.551809 | 0.53491 | 0.585567 | 0.54449 |
| GSE31210.SurvRelapseFree_AllMethods_Freq_6 | trial_66 | 0.614169 | 0.589375 | 0.573443 | 0.578947 | 0.594835 | 0.578634 |
| GSE31210.SurvRelapseFree_AllMethods_Freq_2 | trial_67 | 0.565295 | 0.545359 | 0.525666 | 0.494754 | 0.531195 | 0.500807 |
| GSE31210.SurvRelapseFree_AllMethods_Freq_3 | trial_67 | 0.575366 | 0.55239 | 0.529293 | 0.509939 | 0.537422 | 0.502575 |
| GSE31210.SurvRelapseFree_AllMethods_Freq_4 | trial_67 | 0.572142 | 0.539694 | 0.528744 | 0.511755 | 0.538607 | 0.510749 |
| GSE31210.SurvRelapseFree_AllMethods_Freq_5 | trial_67 | 0.583018 | 0.560773 | 0.551809 | 0.53491 | 0.585567 | 0.54449 |
| GSE31210.SurvRelapseFree_AllMethods_Freq_6 | trial_67 | 0.61408 | 0.589375 | 0.573443 | 0.578947 | 0.594835 | 0.578634 |
| GSE31210.SurvRelapseFree_AllMethods_Freq_2 | trial_68 | 0.568604 | 0.545359 | 0.525666 | 0.494754 | 0.531195 | 0.500807 |
| GSE31210.SurvRelapseFree_AllMethods_Freq_3 | trial_68 | 0.578907 | 0.55239 | 0.529293 | 0.509939 | 0.537422 | 0.502575 |
| GSE31210.SurvRelapseFree_AllMethods_Freq_4 | trial_68 | 0.575201 | 0.539694 | 0.528744 | 0.511755 | 0.538607 | 0.510749 |
| GSE31210.SurvRelapseFree_AllMethods_Freq_5 | trial_68 | 0.586308 | 0.560773 | 0.551809 | 0.53491 | 0.585567 | 0.54449 |
| GSE31210.SurvRelapseFree_AllMethods_Freq_6 | trial_68 | 0.617526 | 0.589375 | 0.573443 | 0.578947 | 0.594835 | 0.578634 |
| GSE31210.SurvRelapseFree_AllMethods_Freq_2 | trial_69 | 0.567353 | 0.545359 | 0.525666 | 0.494754 | 0.531195 | 0.500807 |
| GSE31210.SurvRelapseFree_AllMethods_Freq_3 | trial_69 | 0.57778 | 0.55239 | 0.529293 | 0.509939 | 0.537422 | 0.502575 |
| GSE31210.SurvRelapseFree_AllMethods_Freq_4 | trial_69 | 0.573913 | 0.539694 | 0.528744 | 0.511755 | 0.538607 | 0.510749 |
| GSE31210.SurvRelapseFree_AllMethods_Freq_5 | trial_69 | 0.583594 | 0.560773 | 0.551809 | 0.53491 | 0.585567 | 0.54449 |
| GSE31210.SurvRelapseFree_AllMethods_Freq_6 | trial_69 | 0.616183 | 0.589375 | 0.573443 | 0.578947 | 0.594835 | 0.578634 |
| GSE31210.SurvRelapseFree_AllMethods_Freq_2 | trial_70 | 0.568402 | 0.545359 | 0.525666 | 0.494754 | 0.531195 | 0.500807 |
| GSE31210.SurvRelapseFree_AllMethods_Freq_3 | trial_70 | 0.578545 | 0.55239 | 0.529293 | 0.509939 | 0.537422 | 0.502575 |
| GSE31210.SurvRelapseFree_AllMethods_Freq_4 | trial_70 | 0.574839 | 0.539694 | 0.528744 | 0.511755 | 0.538607 | 0.510749 |
| GSE31210.SurvRelapseFree_AllMethods_Freq_5 | trial_70 | 0.583635 | 0.560773 | 0.551809 | 0.53491 | 0.585567 | 0.54449 |
| GSE31210.SurvRelapseFree_AllMethods_Freq_6 | trial_70 | 0.615288 | 0.589375 | 0.573443 | 0.578947 | 0.594835 | 0.578634 |
| GSE31210.SurvRelapseFree_AllMethods_Freq_2 | trial_71 | 0.56933 | 0.545359 | 0.525666 | 0.494754 | 0.531195 | 0.500807 |
| GSE31210.SurvRelapseFree_AllMethods_Freq_3 | trial_71 | 0.579873 | 0.55239 | 0.529293 | 0.509939 | 0.537422 | 0.502575 |
| GSE31210.SurvRelapseFree_AllMethods_Freq_4 | trial_71 | 0.576167 | 0.539694 | 0.528744 | 0.511755 | 0.538607 | 0.510749 |
| GSE31210.SurvRelapseFree_AllMethods_Freq_5 | trial_71 | 0.586266 | 0.560773 | 0.551809 | 0.53491 | 0.585567 | 0.54449 |
| GSE31210.SurvRelapseFree_AllMethods_Freq_6 | trial_71 | 0.616004 | 0.589375 | 0.573443 | 0.578947 | 0.594835 | 0.578634 |
| GSE31210.SurvRelapseFree_AllMethods_Freq_2 | trial_72 | 0.566102 | 0.545359 | 0.525666 | 0.494754 | 0.531195 | 0.500807 |
| GSE31210.SurvRelapseFree_AllMethods_Freq_3 | trial_72 | 0.576935 | 0.55239 | 0.529293 | 0.509939 | 0.537422 | 0.502575 |
| GSE31210.SurvRelapseFree_AllMethods_Freq_4 | trial_72 | 0.57343 | 0.539694 | 0.528744 | 0.511755 | 0.538607 | 0.510749 |
| GSE31210.SurvRelapseFree_AllMethods_Freq_5 | trial_72 | 0.582771 | 0.560773 | 0.551809 | 0.53491 | 0.585567 | 0.54449 |
| GSE31210.SurvRelapseFree_AllMethods_Freq_6 | trial_72 | 0.611484 | 0.589375 | 0.573443 | 0.578947 | 0.594835 | 0.578634 |
| GSE31210.SurvRelapseFree_AllMethods_Freq_2 | trial_73 | 0.565214 | 0.545359 | 0.525666 | 0.494754 | 0.531195 | 0.500807 |
| GSE31210.SurvRelapseFree_AllMethods_Freq_3 | trial_73 | 0.575527 | 0.55239 | 0.529293 | 0.509939 | 0.537422 | 0.502575 |
| GSE31210.SurvRelapseFree_AllMethods_Freq_4 | trial_73 | 0.572021 | 0.539694 | 0.528744 | 0.511755 | 0.538607 | 0.510749 |
| GSE31210.SurvRelapseFree_AllMethods_Freq_5 | trial_73 | 0.583429 | 0.560773 | 0.551809 | 0.53491 | 0.585567 | 0.54449 |
| GSE31210.SurvRelapseFree_AllMethods_Freq_6 | trial_73 | 0.61323 | 0.589375 | 0.573443 | 0.578947 | 0.594835 | 0.578634 |
| GSE31210.SurvRelapseFree_AllMethods_Freq_2 | trial_74 | 0.568119 | 0.545359 | 0.525666 | 0.494754 | 0.531195 | 0.500807 |
| GSE31210.SurvRelapseFree_AllMethods_Freq_3 | trial_74 | 0.578022 | 0.55239 | 0.529293 | 0.509939 | 0.537422 | 0.502575 |
| GSE31210.SurvRelapseFree_AllMethods_Freq_4 | trial_74 | 0.574074 | 0.539694 | 0.528744 | 0.511755 | 0.538607 | 0.510749 |
| GSE31210.SurvRelapseFree_AllMethods_Freq_5 | trial_74 | 0.584745 | 0.560773 | 0.551809 | 0.53491 | 0.585567 | 0.54449 |
| GSE31210.SurvRelapseFree_AllMethods_Freq_6 | trial_74 | 0.613767 | 0.589375 | 0.573443 | 0.578947 | 0.594835 | 0.578634 |
| GSE31210.SurvRelapseFree_AllMethods_Freq_2 | trial_75 | 0.571953 | 0.545359 | 0.525666 | 0.494754 | 0.531195 | 0.500807 |
| GSE31210.SurvRelapseFree_AllMethods_Freq_3 | trial_75 | 0.582529 | 0.55239 | 0.529293 | 0.509939 | 0.537422 | 0.502575 |
| GSE31210.SurvRelapseFree_AllMethods_Freq_4 | trial_75 | 0.577979 | 0.539694 | 0.528744 | 0.511755 | 0.538607 | 0.510749 |
| GSE31210.SurvRelapseFree_AllMethods_Freq_5 | trial_75 | 0.588322 | 0.560773 | 0.551809 | 0.53491 | 0.585567 | 0.54449 |
| GSE31210.SurvRelapseFree_AllMethods_Freq_6 | trial_75 | 0.61954 | 0.589375 | 0.573443 | 0.578947 | 0.594835 | 0.578634 |
| GSE31210.SurvRelapseFree_AllMethods_Freq_2 | trial_76 | 0.567918 | 0.545359 | 0.525666 | 0.494754 | 0.531195 | 0.500807 |
| GSE31210.SurvRelapseFree_AllMethods_Freq_3 | trial_76 | 0.578143 | 0.55239 | 0.529293 | 0.509939 | 0.537422 | 0.502575 |
| GSE31210.SurvRelapseFree_AllMethods_Freq_4 | trial_76 | 0.574316 | 0.539694 | 0.528744 | 0.511755 | 0.538607 | 0.510749 |
| GSE31210.SurvRelapseFree_AllMethods_Freq_5 | trial_76 | 0.584252 | 0.560773 | 0.551809 | 0.53491 | 0.585567 | 0.54449 |
| GSE31210.SurvRelapseFree_AllMethods_Freq_6 | trial_76 | 0.614259 | 0.589375 | 0.573443 | 0.578947 | 0.594835 | 0.578634 |
| GSE31210.SurvRelapseFree_AllMethods_Freq_2 | trial_77 | 0.566747 | 0.545359 | 0.525666 | 0.494754 | 0.531195 | 0.500807 |
| GSE31210.SurvRelapseFree_AllMethods_Freq_3 | trial_77 | 0.577016 | 0.55239 | 0.529293 | 0.509939 | 0.537422 | 0.502575 |
| GSE31210.SurvRelapseFree_AllMethods_Freq_4 | trial_77 | 0.57339 | 0.539694 | 0.528744 | 0.511755 | 0.538607 | 0.510749 |
| GSE31210.SurvRelapseFree_AllMethods_Freq_5 | trial_77 | 0.583717 | 0.560773 | 0.551809 | 0.53491 | 0.585567 | 0.54449 |
| GSE31210.SurvRelapseFree_AllMethods_Freq_6 | trial_77 | 0.615243 | 0.589375 | 0.573443 | 0.578947 | 0.594835 | 0.578634 |
| GSE31210.SurvRelapseFree_AllMethods_Freq_2 | trial_78 | 0.569048 | 0.545359 | 0.525666 | 0.494754 | 0.531195 | 0.500807 |
| GSE31210.SurvRelapseFree_AllMethods_Freq_3 | trial_78 | 0.578907 | 0.55239 | 0.529293 | 0.509939 | 0.537422 | 0.502575 |
| GSE31210.SurvRelapseFree_AllMethods_Freq_4 | trial_78 | 0.575564 | 0.539694 | 0.528744 | 0.511755 | 0.538607 | 0.510749 |
| GSE31210.SurvRelapseFree_AllMethods_Freq_5 | trial_78 | 0.584868 | 0.560773 | 0.551809 | 0.53491 | 0.585567 | 0.54449 |
| GSE31210.SurvRelapseFree_AllMethods_Freq_6 | trial_78 | 0.614483 | 0.589375 | 0.573443 | 0.578947 | 0.594835 | 0.578634 |
| GSE31210.SurvRelapseFree_AllMethods_Freq_2 | trial_79 | 0.568119 | 0.545359 | 0.525666 | 0.494754 | 0.531195 | 0.500807 |
| GSE31210.SurvRelapseFree_AllMethods_Freq_3 | trial_79 | 0.578384 | 0.55239 | 0.529293 | 0.509939 | 0.537422 | 0.502575 |
| GSE31210.SurvRelapseFree_AllMethods_Freq_4 | trial_79 | 0.573792 | 0.539694 | 0.528744 | 0.511755 | 0.538607 | 0.510749 |
| GSE31210.SurvRelapseFree_AllMethods_Freq_5 | trial_79 | 0.585444 | 0.560773 | 0.551809 | 0.53491 | 0.585567 | 0.54449 |
| GSE31210.SurvRelapseFree_AllMethods_Freq_6 | trial_79 | 0.615467 | 0.589375 | 0.573443 | 0.578947 | 0.594835 | 0.578634 |
| GSE31210.SurvRelapseFree_AllMethods_Freq_2 | trial_80 | 0.560613 | 0.545359 | 0.525666 | 0.494754 | 0.531195 | 0.500807 |
| GSE31210.SurvRelapseFree_AllMethods_Freq_3 | trial_80 | 0.571825 | 0.55239 | 0.529293 | 0.509939 | 0.537422 | 0.502575 |
| GSE31210.SurvRelapseFree_AllMethods_Freq_4 | trial_80 | 0.568438 | 0.539694 | 0.528744 | 0.511755 | 0.538607 | 0.510749 |
| GSE31210.SurvRelapseFree_AllMethods_Freq_5 | trial_80 | 0.580345 | 0.560773 | 0.551809 | 0.53491 | 0.585567 | 0.54449 |
| GSE31210.SurvRelapseFree_AllMethods_Freq_6 | trial_80 | 0.612424 | 0.589375 | 0.573443 | 0.578947 | 0.594835 | 0.578634 |
| GSE31210.SurvRelapseFree_AllMethods_Freq_2 | trial_81 | 0.568927 | 0.545359 | 0.525666 | 0.494754 | 0.531195 | 0.500807 |
| GSE31210.SurvRelapseFree_AllMethods_Freq_3 | trial_81 | 0.578505 | 0.55239 | 0.529293 | 0.509939 | 0.537422 | 0.502575 |
| GSE31210.SurvRelapseFree_AllMethods_Freq_4 | trial_81 | 0.57504 | 0.539694 | 0.528744 | 0.511755 | 0.538607 | 0.510749 |
| GSE31210.SurvRelapseFree_AllMethods_Freq_5 | trial_81 | 0.584457 | 0.560773 | 0.551809 | 0.53491 | 0.585567 | 0.54449 |
| GSE31210.SurvRelapseFree_AllMethods_Freq_6 | trial_81 | 0.615691 | 0.589375 | 0.573443 | 0.578947 | 0.594835 | 0.578634 |
| GSE31210.SurvRelapseFree_AllMethods_Freq_2 | trial_82 | 0.569814 | 0.545359 | 0.525666 | 0.494754 | 0.531195 | 0.500807 |
| GSE31210.SurvRelapseFree_AllMethods_Freq_3 | trial_82 | 0.57931 | 0.55239 | 0.529293 | 0.509939 | 0.537422 | 0.502575 |
| GSE31210.SurvRelapseFree_AllMethods_Freq_4 | trial_82 | 0.575805 | 0.539694 | 0.528744 | 0.511755 | 0.538607 | 0.510749 |
| GSE31210.SurvRelapseFree_AllMethods_Freq_5 | trial_82 | 0.586102 | 0.560773 | 0.551809 | 0.53491 | 0.585567 | 0.54449 |
| GSE31210.SurvRelapseFree_AllMethods_Freq_6 | trial_82 | 0.616944 | 0.589375 | 0.573443 | 0.578947 | 0.594835 | 0.578634 |
| GSE31210.SurvRelapseFree_AllMethods_Freq_2 | trial_83 | 0.570379 | 0.545359 | 0.525666 | 0.494754 | 0.531195 | 0.500807 |
| GSE31210.SurvRelapseFree_AllMethods_Freq_3 | trial_83 | 0.580637 | 0.55239 | 0.529293 | 0.509939 | 0.537422 | 0.502575 |
| GSE31210.SurvRelapseFree_AllMethods_Freq_4 | trial_83 | 0.576409 | 0.539694 | 0.528744 | 0.511755 | 0.538607 | 0.510749 |
| GSE31210.SurvRelapseFree_AllMethods_Freq_5 | trial_83 | 0.585814 | 0.560773 | 0.551809 | 0.53491 | 0.585567 | 0.54449 |
| GSE31210.SurvRelapseFree_AllMethods_Freq_6 | trial_83 | 0.616541 | 0.589375 | 0.573443 | 0.578947 | 0.594835 | 0.578634 |
| GSE31210.SurvRelapseFree_AllMethods_Freq_2 | trial_84 | 0.566747 | 0.545359 | 0.525666 | 0.494754 | 0.531195 | 0.500807 |
| GSE31210.SurvRelapseFree_AllMethods_Freq_3 | trial_84 | 0.576654 | 0.55239 | 0.529293 | 0.509939 | 0.537422 | 0.502575 |
| GSE31210.SurvRelapseFree_AllMethods_Freq_4 | trial_84 | 0.571739 | 0.539694 | 0.528744 | 0.511755 | 0.538607 | 0.510749 |
| GSE31210.SurvRelapseFree_AllMethods_Freq_5 | trial_84 | 0.582155 | 0.560773 | 0.551809 | 0.53491 | 0.585567 | 0.54449 |
| GSE31210.SurvRelapseFree_AllMethods_Freq_6 | trial_84 | 0.613632 | 0.589375 | 0.573443 | 0.578947 | 0.594835 | 0.578634 |
| GSE31210.SurvRelapseFree_AllMethods_Freq_2 | trial_85 | 0.5659 | 0.545359 | 0.525666 | 0.494754 | 0.531195 | 0.500807 |
| GSE31210.SurvRelapseFree_AllMethods_Freq_3 | trial_85 | 0.576412 | 0.55239 | 0.529293 | 0.509939 | 0.537422 | 0.502575 |
| GSE31210.SurvRelapseFree_AllMethods_Freq_4 | trial_85 | 0.573269 | 0.539694 | 0.528744 | 0.511755 | 0.538607 | 0.510749 |
| GSE31210.SurvRelapseFree_AllMethods_Freq_5 | trial_85 | 0.583183 | 0.560773 | 0.551809 | 0.53491 | 0.585567 | 0.54449 |
| GSE31210.SurvRelapseFree_AllMethods_Freq_6 | trial_85 | 0.615915 | 0.589375 | 0.573443 | 0.578947 | 0.594835 | 0.578634 |
| GSE31210.SurvRelapseFree_AllMethods_Freq_2 | trial_86 | 0.568765 | 0.545359 | 0.525666 | 0.494754 | 0.531195 | 0.500807 |
| GSE31210.SurvRelapseFree_AllMethods_Freq_3 | trial_86 | 0.578625 | 0.55239 | 0.529293 | 0.509939 | 0.537422 | 0.502575 |
| GSE31210.SurvRelapseFree_AllMethods_Freq_4 | trial_86 | 0.574758 | 0.539694 | 0.528744 | 0.511755 | 0.538607 | 0.510749 |
| GSE31210.SurvRelapseFree_AllMethods_Freq_5 | trial_86 | 0.584539 | 0.560773 | 0.551809 | 0.53491 | 0.585567 | 0.54449 |
| GSE31210.SurvRelapseFree_AllMethods_Freq_6 | trial_86 | 0.613274 | 0.589375 | 0.573443 | 0.578947 | 0.594835 | 0.578634 |
| GSE31210.SurvRelapseFree_AllMethods_Freq_2 | trial_87 | 0.567151 | 0.545359 | 0.525666 | 0.494754 | 0.531195 | 0.500807 |
| GSE31210.SurvRelapseFree_AllMethods_Freq_3 | trial_87 | 0.57762 | 0.55239 | 0.529293 | 0.509939 | 0.537422 | 0.502575 |
| GSE31210.SurvRelapseFree_AllMethods_Freq_4 | trial_87 | 0.575483 | 0.539694 | 0.528744 | 0.511755 | 0.538607 | 0.510749 |
| GSE31210.SurvRelapseFree_AllMethods_Freq_5 | trial_87 | 0.585444 | 0.560773 | 0.551809 | 0.53491 | 0.585567 | 0.54449 |
| GSE31210.SurvRelapseFree_AllMethods_Freq_6 | trial_87 | 0.614841 | 0.589375 | 0.573443 | 0.578947 | 0.594835 | 0.578634 |
| GSE31210.SurvRelapseFree_AllMethods_Freq_2 | trial_88 | 0.569935 | 0.545359 | 0.525666 | 0.494754 | 0.531195 | 0.500807 |
| GSE31210.SurvRelapseFree_AllMethods_Freq_3 | trial_88 | 0.580114 | 0.55239 | 0.529293 | 0.509939 | 0.537422 | 0.502575 |
| GSE31210.SurvRelapseFree_AllMethods_Freq_4 | trial_88 | 0.575604 | 0.539694 | 0.528744 | 0.511755 | 0.538607 | 0.510749 |
| GSE31210.SurvRelapseFree_AllMethods_Freq_5 | trial_88 | 0.586554 | 0.560773 | 0.551809 | 0.53491 | 0.585567 | 0.54449 |
| GSE31210.SurvRelapseFree_AllMethods_Freq_6 | trial_88 | 0.618511 | 0.589375 | 0.573443 | 0.578947 | 0.594835 | 0.578634 |
| GSE31210.SurvRelapseFree_AllMethods_Freq_2 | trial_89 | 0.568563 | 0.545359 | 0.525666 | 0.494754 | 0.531195 | 0.500807 |
| GSE31210.SurvRelapseFree_AllMethods_Freq_3 | trial_89 | 0.578746 | 0.55239 | 0.529293 | 0.509939 | 0.537422 | 0.502575 |
| GSE31210.SurvRelapseFree_AllMethods_Freq_4 | trial_89 | 0.575242 | 0.539694 | 0.528744 | 0.511755 | 0.538607 | 0.510749 |
| GSE31210.SurvRelapseFree_AllMethods_Freq_5 | trial_89 | 0.585691 | 0.560773 | 0.551809 | 0.53491 | 0.585567 | 0.54449 |
| GSE31210.SurvRelapseFree_AllMethods_Freq_6 | trial_89 | 0.615825 | 0.589375 | 0.573443 | 0.578947 | 0.594835 | 0.578634 |
| GSE31210.SurvRelapseFree_AllMethods_Freq_2 | trial_90 | 0.567958 | 0.545359 | 0.525666 | 0.494754 | 0.531195 | 0.500807 |
| GSE31210.SurvRelapseFree_AllMethods_Freq_3 | trial_90 | 0.578062 | 0.55239 | 0.529293 | 0.509939 | 0.537422 | 0.502575 |
| GSE31210.SurvRelapseFree_AllMethods_Freq_4 | trial_90 | 0.575201 | 0.539694 | 0.528744 | 0.511755 | 0.538607 | 0.510749 |
| GSE31210.SurvRelapseFree_AllMethods_Freq_5 | trial_90 | 0.585362 | 0.560773 | 0.551809 | 0.53491 | 0.585567 | 0.54449 |
| GSE31210.SurvRelapseFree_AllMethods_Freq_6 | trial_90 | 0.616676 | 0.589375 | 0.573443 | 0.578947 | 0.594835 | 0.578634 |
| GSE31210.SurvRelapseFree_AllMethods_Freq_2 | trial_91 | 0.567797 | 0.545359 | 0.525666 | 0.494754 | 0.531195 | 0.500807 |
| GSE31210.SurvRelapseFree_AllMethods_Freq_3 | trial_91 | 0.577982 | 0.55239 | 0.529293 | 0.509939 | 0.537422 | 0.502575 |
| GSE31210.SurvRelapseFree_AllMethods_Freq_4 | trial_91 | 0.575161 | 0.539694 | 0.528744 | 0.511755 | 0.538607 | 0.510749 |
| GSE31210.SurvRelapseFree_AllMethods_Freq_5 | trial_91 | 0.585238 | 0.560773 | 0.551809 | 0.53491 | 0.585567 | 0.54449 |
| GSE31210.SurvRelapseFree_AllMethods_Freq_6 | trial_91 | 0.616228 | 0.589375 | 0.573443 | 0.578947 | 0.594835 | 0.578634 |
| GSE31210.SurvRelapseFree_AllMethods_Freq_2 | trial_92 | 0.567837 | 0.545359 | 0.525666 | 0.494754 | 0.531195 | 0.500807 |
| GSE31210.SurvRelapseFree_AllMethods_Freq_3 | trial_92 | 0.57778 | 0.55239 | 0.529293 | 0.509939 | 0.537422 | 0.502575 |
| GSE31210.SurvRelapseFree_AllMethods_Freq_4 | trial_92 | 0.573309 | 0.539694 | 0.528744 | 0.511755 | 0.538607 | 0.510749 |
| GSE31210.SurvRelapseFree_AllMethods_Freq_5 | trial_92 | 0.585567 | 0.560773 | 0.551809 | 0.53491 | 0.585567 | 0.54449 |
| GSE31210.SurvRelapseFree_AllMethods_Freq_6 | trial_92 | 0.61672 | 0.589375 | 0.573443 | 0.578947 | 0.594835 | 0.578634 |
| GSE31210.SurvRelapseFree_AllMethods_Freq_2 | trial_93 | 0.57042 | 0.545359 | 0.525666 | 0.494754 | 0.531195 | 0.500807 |
| GSE31210.SurvRelapseFree_AllMethods_Freq_3 | trial_93 | 0.579953 | 0.55239 | 0.529293 | 0.509939 | 0.537422 | 0.502575 |
| GSE31210.SurvRelapseFree_AllMethods_Freq_4 | trial_93 | 0.575644 | 0.539694 | 0.528744 | 0.511755 | 0.538607 | 0.510749 |
| GSE31210.SurvRelapseFree_AllMethods_Freq_5 | trial_93 | 0.585938 | 0.560773 | 0.551809 | 0.53491 | 0.585567 | 0.54449 |
| GSE31210.SurvRelapseFree_AllMethods_Freq_6 | trial_93 | 0.616183 | 0.589375 | 0.573443 | 0.578947 | 0.594835 | 0.578634 |
| GSE31210.SurvRelapseFree_AllMethods_Freq_2 | trial_94 | 0.568321 | 0.545359 | 0.525666 | 0.494754 | 0.531195 | 0.500807 |
| GSE31210.SurvRelapseFree_AllMethods_Freq_3 | trial_94 | 0.578505 | 0.55239 | 0.529293 | 0.509939 | 0.537422 | 0.502575 |
| GSE31210.SurvRelapseFree_AllMethods_Freq_4 | trial_94 | 0.574436 | 0.539694 | 0.528744 | 0.511755 | 0.538607 | 0.510749 |
| GSE31210.SurvRelapseFree_AllMethods_Freq_5 | trial_94 | 0.583594 | 0.560773 | 0.551809 | 0.53491 | 0.585567 | 0.54449 |
| GSE31210.SurvRelapseFree_AllMethods_Freq_6 | trial_94 | 0.613588 | 0.589375 | 0.573443 | 0.578947 | 0.594835 | 0.578634 |
| GSE31210.SurvRelapseFree_AllMethods_Freq_2 | trial_95 | 0.568846 | 0.545359 | 0.525666 | 0.494754 | 0.531195 | 0.500807 |
| GSE31210.SurvRelapseFree_AllMethods_Freq_3 | trial_95 | 0.579068 | 0.55239 | 0.529293 | 0.509939 | 0.537422 | 0.502575 |
| GSE31210.SurvRelapseFree_AllMethods_Freq_4 | trial_95 | 0.575886 | 0.539694 | 0.528744 | 0.511755 | 0.538607 | 0.510749 |
| GSE31210.SurvRelapseFree_AllMethods_Freq_5 | trial_95 | 0.586719 | 0.560773 | 0.551809 | 0.53491 | 0.585567 | 0.54449 |
| GSE31210.SurvRelapseFree_AllMethods_Freq_6 | trial_95 | 0.617839 | 0.589375 | 0.573443 | 0.578947 | 0.594835 | 0.578634 |
| GSE31210.SurvRelapseFree_AllMethods_Freq_2 | trial_96 | 0.567918 | 0.545359 | 0.525666 | 0.494754 | 0.531195 | 0.500807 |
| GSE31210.SurvRelapseFree_AllMethods_Freq_3 | trial_96 | 0.578102 | 0.55239 | 0.529293 | 0.509939 | 0.537422 | 0.502575 |
| GSE31210.SurvRelapseFree_AllMethods_Freq_4 | trial_96 | 0.574396 | 0.539694 | 0.528744 | 0.511755 | 0.538607 | 0.510749 |
| GSE31210.SurvRelapseFree_AllMethods_Freq_5 | trial_96 | 0.585526 | 0.560773 | 0.551809 | 0.53491 | 0.585567 | 0.54449 |
| GSE31210.SurvRelapseFree_AllMethods_Freq_6 | trial_96 | 0.614975 | 0.589375 | 0.573443 | 0.578947 | 0.594835 | 0.578634 |
| GSE31210.SurvRelapseFree_AllMethods_Freq_2 | trial_97 | 0.566707 | 0.545359 | 0.525666 | 0.494754 | 0.531195 | 0.500807 |
| GSE31210.SurvRelapseFree_AllMethods_Freq_3 | trial_97 | 0.577016 | 0.55239 | 0.529293 | 0.509939 | 0.537422 | 0.502575 |
| GSE31210.SurvRelapseFree_AllMethods_Freq_4 | trial_97 | 0.572907 | 0.539694 | 0.528744 | 0.511755 | 0.538607 | 0.510749 |
| GSE31210.SurvRelapseFree_AllMethods_Freq_5 | trial_97 | 0.583594 | 0.560773 | 0.551809 | 0.53491 | 0.585567 | 0.54449 |
| GSE31210.SurvRelapseFree_AllMethods_Freq_6 | trial_97 | 0.614214 | 0.589375 | 0.573443 | 0.578947 | 0.594835 | 0.578634 |
| GSE31210.SurvRelapseFree_AllMethods_Freq_2 | trial_98 | 0.567756 | 0.545359 | 0.525666 | 0.494754 | 0.531195 | 0.500807 |
| GSE31210.SurvRelapseFree_AllMethods_Freq_3 | trial_98 | 0.577378 | 0.55239 | 0.529293 | 0.509939 | 0.537422 | 0.502575 |
| GSE31210.SurvRelapseFree_AllMethods_Freq_4 | trial_98 | 0.573551 | 0.539694 | 0.528744 | 0.511755 | 0.538607 | 0.510749 |
| GSE31210.SurvRelapseFree_AllMethods_Freq_5 | trial_98 | 0.584581 | 0.560773 | 0.551809 | 0.53491 | 0.585567 | 0.54449 |
| GSE31210.SurvRelapseFree_AllMethods_Freq_6 | trial_98 | 0.615154 | 0.589375 | 0.573443 | 0.578947 | 0.594835 | 0.578634 |
| GSE31210.SurvRelapseFree_AllMethods_Freq_2 | trial_99 | 0.565295 | 0.545359 | 0.525666 | 0.494754 | 0.531195 | 0.500807 |
| GSE31210.SurvRelapseFree_AllMethods_Freq_3 | trial_99 | 0.575527 | 0.55239 | 0.529293 | 0.509939 | 0.537422 | 0.502575 |
| GSE31210.SurvRelapseFree_AllMethods_Freq_4 | trial_99 | 0.572987 | 0.539694 | 0.528744 | 0.511755 | 0.538607 | 0.510749 |
| GSE31210.SurvRelapseFree_AllMethods_Freq_5 | trial_99 | 0.582936 | 0.560773 | 0.551809 | 0.53491 | 0.585567 | 0.54449 |
| GSE31210.SurvRelapseFree_AllMethods_Freq_6 | trial_99 | 0.613364 | 0.589375 | 0.573443 | 0.578947 | 0.594835 | 0.578634 |
| GSE31210.SurvRelapseFree_AllMethods_Freq_2 | trial_100 | 0.566909 | 0.545359 | 0.525666 | 0.494754 | 0.531195 | 0.500807 |
| GSE31210.SurvRelapseFree_AllMethods_Freq_3 | trial_100 | 0.577499 | 0.55239 | 0.529293 | 0.509939 | 0.537422 | 0.502575 |
| GSE31210.SurvRelapseFree_AllMethods_Freq_4 | trial_100 | 0.574275 | 0.539694 | 0.528744 | 0.511755 | 0.538607 | 0.510749 |
| GSE31210.SurvRelapseFree_AllMethods_Freq_5 | trial_100 | 0.583265 | 0.560773 | 0.551809 | 0.53491 | 0.585567 | 0.54449 |
| GSE31210.SurvRelapseFree_AllMethods_Freq_6 | trial_100 | 0.613006 | 0.589375 | 0.573443 | 0.578947 | 0.594835 | 0.578634 |
| HARVARD-LC.Surv_AllMethods_Freq_2 | trial_1 | 0.789593 | 0.707014 | 0.779412 | 0.763575 | 0.781674 | 0.723982 |
| HARVARD-LC.Surv_AllMethods_Freq_2 | trial_2 | 0.799774 | 0.707014 | 0.779412 | 0.763575 | 0.781674 | 0.723982 |
| HARVARD-LC.Surv_AllMethods_Freq_2 | trial_3 | 0.774887 | 0.707014 | 0.779412 | 0.763575 | 0.781674 | 0.723982 |
| HARVARD-LC.Surv_AllMethods_Freq_2 | trial_4 | 0.797511 | 0.707014 | 0.779412 | 0.763575 | 0.781674 | 0.723982 |
| HARVARD-LC.Surv_AllMethods_Freq_2 | trial_5 | 0.779412 | 0.707014 | 0.779412 | 0.763575 | 0.781674 | 0.723982 |
| HARVARD-LC.Surv_AllMethods_Freq_2 | trial_6 | 0.772624 | 0.707014 | 0.779412 | 0.763575 | 0.781674 | 0.723982 |
| HARVARD-LC.Surv_AllMethods_Freq_2 | trial_7 | 0.773756 | 0.707014 | 0.779412 | 0.763575 | 0.781674 | 0.723982 |
| HARVARD-LC.Surv_AllMethods_Freq_2 | trial_8 | 0.770362 | 0.707014 | 0.779412 | 0.763575 | 0.781674 | 0.723982 |
| HARVARD-LC.Surv_AllMethods_Freq_2 | trial_9 | 0.788462 | 0.707014 | 0.779412 | 0.763575 | 0.781674 | 0.723982 |
| HARVARD-LC.Surv_AllMethods_Freq_2 | trial_10 | 0.789593 | 0.707014 | 0.779412 | 0.763575 | 0.781674 | 0.723982 |
| HARVARD-LC.Surv_AllMethods_Freq_2 | trial_11 | 0.789593 | 0.707014 | 0.779412 | 0.763575 | 0.781674 | 0.723982 |
| HARVARD-LC.Surv_AllMethods_Freq_2 | trial_12 | 0.781674 | 0.707014 | 0.779412 | 0.763575 | 0.781674 | 0.723982 |
| HARVARD-LC.Surv_AllMethods_Freq_2 | trial_13 | 0.785068 | 0.707014 | 0.779412 | 0.763575 | 0.781674 | 0.723982 |
| HARVARD-LC.Surv_AllMethods_Freq_2 | trial_14 | 0.780543 | 0.707014 | 0.779412 | 0.763575 | 0.781674 | 0.723982 |
| HARVARD-LC.Surv_AllMethods_Freq_2 | trial_15 | 0.776018 | 0.707014 | 0.779412 | 0.763575 | 0.781674 | 0.723982 |
| HARVARD-LC.Surv_AllMethods_Freq_2 | trial_16 | 0.7681 | 0.707014 | 0.779412 | 0.763575 | 0.781674 | 0.723982 |
| HARVARD-LC.Surv_AllMethods_Freq_2 | trial_17 | 0.762443 | 0.707014 | 0.779412 | 0.763575 | 0.781674 | 0.723982 |
| HARVARD-LC.Surv_AllMethods_Freq_2 | trial_18 | 0.786199 | 0.707014 | 0.779412 | 0.763575 | 0.781674 | 0.723982 |
| HARVARD-LC.Surv_AllMethods_Freq_2 | trial_19 | 0.808824 | 0.707014 | 0.779412 | 0.763575 | 0.781674 | 0.723982 |
| HARVARD-LC.Surv_AllMethods_Freq_2 | trial_20 | 0.779412 | 0.707014 | 0.779412 | 0.763575 | 0.781674 | 0.723982 |
| HARVARD-LC.Surv_AllMethods_Freq_2 | trial_21 | 0.789593 | 0.707014 | 0.779412 | 0.763575 | 0.781674 | 0.723982 |
| HARVARD-LC.Surv_AllMethods_Freq_2 | trial_22 | 0.78733 | 0.707014 | 0.779412 | 0.763575 | 0.781674 | 0.723982 |
| HARVARD-LC.Surv_AllMethods_Freq_2 | trial_23 | 0.774887 | 0.707014 | 0.779412 | 0.763575 | 0.781674 | 0.723982 |
| HARVARD-LC.Surv_AllMethods_Freq_2 | trial_24 | 0.789593 | 0.707014 | 0.779412 | 0.763575 | 0.781674 | 0.723982 |
| HARVARD-LC.Surv_AllMethods_Freq_2 | trial_25 | 0.788462 | 0.707014 | 0.779412 | 0.763575 | 0.781674 | 0.723982 |
| HARVARD-LC.Surv_AllMethods_Freq_2 | trial_26 | 0.786199 | 0.707014 | 0.779412 | 0.763575 | 0.781674 | 0.723982 |
| HARVARD-LC.Surv_AllMethods_Freq_2 | trial_27 | 0.792986 | 0.707014 | 0.779412 | 0.763575 | 0.781674 | 0.723982 |
| HARVARD-LC.Surv_AllMethods_Freq_2 | trial_28 | 0.772624 | 0.707014 | 0.779412 | 0.763575 | 0.781674 | 0.723982 |
| HARVARD-LC.Surv_AllMethods_Freq_2 | trial_29 | 0.788462 | 0.707014 | 0.779412 | 0.763575 | 0.781674 | 0.723982 |
| HARVARD-LC.Surv_AllMethods_Freq_2 | trial_30 | 0.774887 | 0.707014 | 0.779412 | 0.763575 | 0.781674 | 0.723982 |
| HARVARD-LC.Surv_AllMethods_Freq_2 | trial_31 | 0.785068 | 0.707014 | 0.779412 | 0.763575 | 0.781674 | 0.723982 |
| HARVARD-LC.Surv_AllMethods_Freq_2 | trial_32 | 0.795249 | 0.707014 | 0.779412 | 0.763575 | 0.781674 | 0.723982 |
| HARVARD-LC.Surv_AllMethods_Freq_2 | trial_33 | 0.806561 | 0.707014 | 0.779412 | 0.763575 | 0.781674 | 0.723982 |
| HARVARD-LC.Surv_AllMethods_Freq_2 | trial_34 | 0.789593 | 0.707014 | 0.779412 | 0.763575 | 0.781674 | 0.723982 |
| HARVARD-LC.Surv_AllMethods_Freq_2 | trial_35 | 0.779412 | 0.707014 | 0.779412 | 0.763575 | 0.781674 | 0.723982 |
| HARVARD-LC.Surv_AllMethods_Freq_2 | trial_36 | 0.799774 | 0.707014 | 0.779412 | 0.763575 | 0.781674 | 0.723982 |
| HARVARD-LC.Surv_AllMethods_Freq_2 | trial_37 | 0.791855 | 0.707014 | 0.779412 | 0.763575 | 0.781674 | 0.723982 |
| HARVARD-LC.Surv_AllMethods_Freq_2 | trial_38 | 0.773756 | 0.707014 | 0.779412 | 0.763575 | 0.781674 | 0.723982 |
| HARVARD-LC.Surv_AllMethods_Freq_2 | trial_39 | 0.78733 | 0.707014 | 0.779412 | 0.763575 | 0.781674 | 0.723982 |
| HARVARD-LC.Surv_AllMethods_Freq_2 | trial_40 | 0.771493 | 0.707014 | 0.779412 | 0.763575 | 0.781674 | 0.723982 |
| HARVARD-LC.Surv_AllMethods_Freq_2 | trial_41 | 0.785068 | 0.707014 | 0.779412 | 0.763575 | 0.781674 | 0.723982 |
| HARVARD-LC.Surv_AllMethods_Freq_2 | trial_42 | 0.766968 | 0.707014 | 0.779412 | 0.763575 | 0.781674 | 0.723982 |
| HARVARD-LC.Surv_AllMethods_Freq_2 | trial_43 | 0.799774 | 0.707014 | 0.779412 | 0.763575 | 0.781674 | 0.723982 |
| HARVARD-LC.Surv_AllMethods_Freq_2 | trial_44 | 0.779412 | 0.707014 | 0.779412 | 0.763575 | 0.781674 | 0.723982 |
| HARVARD-LC.Surv_AllMethods_Freq_2 | trial_45 | 0.776018 | 0.707014 | 0.779412 | 0.763575 | 0.781674 | 0.723982 |
| HARVARD-LC.Surv_AllMethods_Freq_2 | trial_46 | 0.786199 | 0.707014 | 0.779412 | 0.763575 | 0.781674 | 0.723982 |
| HARVARD-LC.Surv_AllMethods_Freq_2 | trial_47 | 0.753394 | 0.707014 | 0.779412 | 0.763575 | 0.781674 | 0.723982 |
| HARVARD-LC.Surv_AllMethods_Freq_2 | trial_48 | 0.792986 | 0.707014 | 0.779412 | 0.763575 | 0.781674 | 0.723982 |
| HARVARD-LC.Surv_AllMethods_Freq_2 | trial_49 | 0.781674 | 0.707014 | 0.779412 | 0.763575 | 0.781674 | 0.723982 |
| HARVARD-LC.Surv_AllMethods_Freq_2 | trial_50 | 0.766968 | 0.707014 | 0.779412 | 0.763575 | 0.781674 | 0.723982 |
| HARVARD-LC.Surv_AllMethods_Freq_2 | trial_51 | 0.788462 | 0.707014 | 0.779412 | 0.763575 | 0.781674 | 0.723982 |
| HARVARD-LC.Surv_AllMethods_Freq_2 | trial_52 | 0.790724 | 0.707014 | 0.779412 | 0.763575 | 0.781674 | 0.723982 |
| HARVARD-LC.Surv_AllMethods_Freq_2 | trial_53 | 0.78733 | 0.707014 | 0.779412 | 0.763575 | 0.781674 | 0.723982 |
| HARVARD-LC.Surv_AllMethods_Freq_2 | trial_54 | 0.785068 | 0.707014 | 0.779412 | 0.763575 | 0.781674 | 0.723982 |
| HARVARD-LC.Surv_AllMethods_Freq_2 | trial_55 | 0.795249 | 0.707014 | 0.779412 | 0.763575 | 0.781674 | 0.723982 |
| HARVARD-LC.Surv_AllMethods_Freq_2 | trial_56 | 0.772624 | 0.707014 | 0.779412 | 0.763575 | 0.781674 | 0.723982 |
| HARVARD-LC.Surv_AllMethods_Freq_2 | trial_57 | 0.782805 | 0.707014 | 0.779412 | 0.763575 | 0.781674 | 0.723982 |
| HARVARD-LC.Surv_AllMethods_Freq_2 | trial_58 | 0.798643 | 0.707014 | 0.779412 | 0.763575 | 0.781674 | 0.723982 |
| HARVARD-LC.Surv_AllMethods_Freq_2 | trial_59 | 0.774887 | 0.707014 | 0.779412 | 0.763575 | 0.781674 | 0.723982 |
| HARVARD-LC.Surv_AllMethods_Freq_2 | trial_60 | 0.778281 | 0.707014 | 0.779412 | 0.763575 | 0.781674 | 0.723982 |
| HARVARD-LC.Surv_AllMethods_Freq_2 | trial_61 | 0.79638 | 0.707014 | 0.779412 | 0.763575 | 0.781674 | 0.723982 |
| HARVARD-LC.Surv_AllMethods_Freq_2 | trial_62 | 0.778281 | 0.707014 | 0.779412 | 0.763575 | 0.781674 | 0.723982 |
| HARVARD-LC.Surv_AllMethods_Freq_2 | trial_63 | 0.769231 | 0.707014 | 0.779412 | 0.763575 | 0.781674 | 0.723982 |
| HARVARD-LC.Surv_AllMethods_Freq_2 | trial_64 | 0.788462 | 0.707014 | 0.779412 | 0.763575 | 0.781674 | 0.723982 |
| HARVARD-LC.Surv_AllMethods_Freq_2 | trial_65 | 0.795249 | 0.707014 | 0.779412 | 0.763575 | 0.781674 | 0.723982 |
| HARVARD-LC.Surv_AllMethods_Freq_2 | trial_66 | 0.766968 | 0.707014 | 0.779412 | 0.763575 | 0.781674 | 0.723982 |
| HARVARD-LC.Surv_AllMethods_Freq_2 | trial_67 | 0.785068 | 0.707014 | 0.779412 | 0.763575 | 0.781674 | 0.723982 |
| HARVARD-LC.Surv_AllMethods_Freq_2 | trial_68 | 0.802036 | 0.707014 | 0.779412 | 0.763575 | 0.781674 | 0.723982 |
| HARVARD-LC.Surv_AllMethods_Freq_2 | trial_69 | 0.789593 | 0.707014 | 0.779412 | 0.763575 | 0.781674 | 0.723982 |
| HARVARD-LC.Surv_AllMethods_Freq_2 | trial_70 | 0.777149 | 0.707014 | 0.779412 | 0.763575 | 0.781674 | 0.723982 |
| HARVARD-LC.Surv_AllMethods_Freq_2 | trial_71 | 0.78733 | 0.707014 | 0.779412 | 0.763575 | 0.781674 | 0.723982 |
| HARVARD-LC.Surv_AllMethods_Freq_2 | trial_72 | 0.788462 | 0.707014 | 0.779412 | 0.763575 | 0.781674 | 0.723982 |
| HARVARD-LC.Surv_AllMethods_Freq_2 | trial_73 | 0.795249 | 0.707014 | 0.779412 | 0.763575 | 0.781674 | 0.723982 |
| HARVARD-LC.Surv_AllMethods_Freq_2 | trial_74 | 0.766968 | 0.707014 | 0.779412 | 0.763575 | 0.781674 | 0.723982 |
| HARVARD-LC.Surv_AllMethods_Freq_2 | trial_75 | 0.786199 | 0.707014 | 0.779412 | 0.763575 | 0.781674 | 0.723982 |
| HARVARD-LC.Surv_AllMethods_Freq_2 | trial_76 | 0.788462 | 0.707014 | 0.779412 | 0.763575 | 0.781674 | 0.723982 |
| HARVARD-LC.Surv_AllMethods_Freq_2 | trial_77 | 0.764706 | 0.707014 | 0.779412 | 0.763575 | 0.781674 | 0.723982 |
| HARVARD-LC.Surv_AllMethods_Freq_2 | trial_78 | 0.776018 | 0.707014 | 0.779412 | 0.763575 | 0.781674 | 0.723982 |
| HARVARD-LC.Surv_AllMethods_Freq_2 | trial_79 | 0.774887 | 0.707014 | 0.779412 | 0.763575 | 0.781674 | 0.723982 |
| HARVARD-LC.Surv_AllMethods_Freq_2 | trial_80 | 0.777149 | 0.707014 | 0.779412 | 0.763575 | 0.781674 | 0.723982 |
| HARVARD-LC.Surv_AllMethods_Freq_2 | trial_81 | 0.781674 | 0.707014 | 0.779412 | 0.763575 | 0.781674 | 0.723982 |
| HARVARD-LC.Surv_AllMethods_Freq_2 | trial_82 | 0.806561 | 0.707014 | 0.779412 | 0.763575 | 0.781674 | 0.723982 |
| HARVARD-LC.Surv_AllMethods_Freq_2 | trial_83 | 0.765837 | 0.707014 | 0.779412 | 0.763575 | 0.781674 | 0.723982 |
| HARVARD-LC.Surv_AllMethods_Freq_2 | trial_84 | 0.762443 | 0.707014 | 0.779412 | 0.763575 | 0.781674 | 0.723982 |
| HARVARD-LC.Surv_AllMethods_Freq_2 | trial_85 | 0.786199 | 0.707014 | 0.779412 | 0.763575 | 0.781674 | 0.723982 |
| HARVARD-LC.Surv_AllMethods_Freq_2 | trial_86 | 0.780543 | 0.707014 | 0.779412 | 0.763575 | 0.781674 | 0.723982 |
| HARVARD-LC.Surv_AllMethods_Freq_2 | trial_87 | 0.78733 | 0.707014 | 0.779412 | 0.763575 | 0.781674 | 0.723982 |
| HARVARD-LC.Surv_AllMethods_Freq_2 | trial_88 | 0.780543 | 0.707014 | 0.779412 | 0.763575 | 0.781674 | 0.723982 |
| HARVARD-LC.Surv_AllMethods_Freq_2 | trial_89 | 0.794118 | 0.707014 | 0.779412 | 0.763575 | 0.781674 | 0.723982 |
| HARVARD-LC.Surv_AllMethods_Freq_2 | trial_90 | 0.762443 | 0.707014 | 0.779412 | 0.763575 | 0.781674 | 0.723982 |
| HARVARD-LC.Surv_AllMethods_Freq_2 | trial_91 | 0.789593 | 0.707014 | 0.779412 | 0.763575 | 0.781674 | 0.723982 |
| HARVARD-LC.Surv_AllMethods_Freq_2 | trial_92 | 0.766968 | 0.707014 | 0.779412 | 0.763575 | 0.781674 | 0.723982 |
| HARVARD-LC.Surv_AllMethods_Freq_2 | trial_93 | 0.771493 | 0.707014 | 0.779412 | 0.763575 | 0.781674 | 0.723982 |
| HARVARD-LC.Surv_AllMethods_Freq_2 | trial_94 | 0.770362 | 0.707014 | 0.779412 | 0.763575 | 0.781674 | 0.723982 |
| HARVARD-LC.Surv_AllMethods_Freq_2 | trial_95 | 0.78733 | 0.707014 | 0.779412 | 0.763575 | 0.781674 | 0.723982 |
| HARVARD-LC.Surv_AllMethods_Freq_2 | trial_96 | 0.772624 | 0.707014 | 0.779412 | 0.763575 | 0.781674 | 0.723982 |
| HARVARD-LC.Surv_AllMethods_Freq_2 | trial_97 | 0.778281 | 0.707014 | 0.779412 | 0.763575 | 0.781674 | 0.723982 |
| HARVARD-LC.Surv_AllMethods_Freq_2 | trial_98 | 0.792986 | 0.707014 | 0.779412 | 0.763575 | 0.781674 | 0.723982 |
| HARVARD-LC.Surv_AllMethods_Freq_2 | trial_99 | 0.791855 | 0.707014 | 0.779412 | 0.763575 | 0.781674 | 0.723982 |
| HARVARD-LC.Surv_AllMethods_Freq_2 | trial_100 | 0.774887 | 0.707014 | 0.779412 | 0.763575 | 0.781674 | 0.723982 |

**Table C.** AUCs of all methods for each trial of tests, from which **Table B** is created. Rows with all NA in all methods indicated there is no AUC for corresponding method and trial datasets, where no genes in the Truth gene lists were derived from Training Set data, which were deleted from the table to save space (total 3900 rows or cases like that). The general ROC analysis procedure was described in Material and methods section. Column “PrognoScan_TruthGeneLists”: Truth Gene Lists from each indicated PrognoScan LUAD datasets (GSE13213, GSE31210 etc) with shared gene lists from the methods in comparison with the indicated frequency (Freq_2 for shared by 2 of method lists; Freq_2 for shared by 3 of method lists, and so on so forth). Column “TCGA_Trials”: trials derived from TCGA LUAD data (total 100 trials from different trials of permutations in our method). Other columns showed the corresponding AUCs for each method in each of the combined truth gene list of PrognoScan datasets at different shared frequency (Freq_2, Freq_3 etc) and each of the TCGA_Trials in each row.


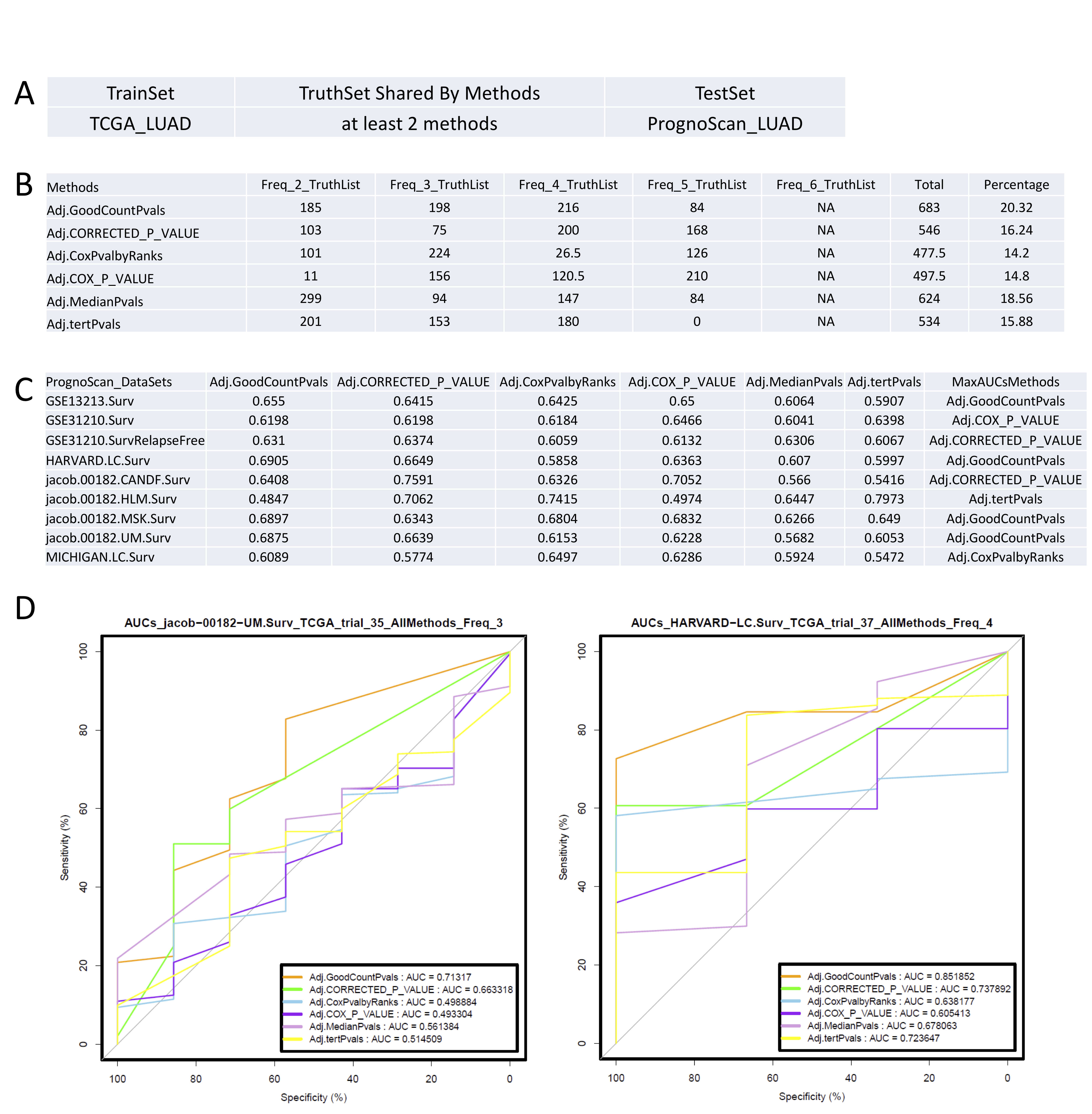


**Figure P.** Performance comparison by ROC analysis comparing all listed methods for the same set of truth gene lists that were shared by multiple truth lists derived from individual method on TCGA LUAD data as Training Set and PrognoScan LUAD data as Testing Set. All Ras pathway genes were used for analysis. (A). the setting table show what datasets have been used for the Training Sets and Testing Sets, as well as whether the Truth Set of genes derived from training are shared list from at least 2 listed methods. Downloaded TCGA LUAD (lung adenocarcinoma) datasets were used as Training Sets data. Downloaded PrognoScan LUAD data was used as Testing Set data. Due to GradientScanSurv method has a step of permutations, multiple trials (total 100) of GradientScanSurv analysis were used in combination with other methods results to form trials of Training Set data. The general ROC analysis procedure was described in Material and method section. Specifically, the shared positive gene lists in at least two of the selected methods from the Training Set were used as Truth lists for each method in the Testing Set data. (B). Performance comparison table listed numbers of best times/trials that a method has the largest AUC of ROC curves comparing other methods for the same trial of truth gene lists (shared by at least 2 listed methods) and same Testing dataset. All truth lists from training and result lists derived from Testing Set all are at adjusted p-value<=0.05 for each method. Freq_2_TruthList consists of the truth gene list shared by identified genes from at least 2 of these selected methods; Freq_3_TruthList consists of the truth gene list shared by identified genes from at least 3 of these selected methods; so on and so forth. Column Total summarized the total counts for all scenarios (Freq_2_TruthList , Freq_3_TruthList etc) of each method listed in column Methods. Percentage listed the proportions of those counts. The GradientScanSurv method (Adj.GoodCountPvals) has the largest percentage at 21.13% of total trials with largest AUCs comparing other methods including PrognoScan method (Adj.CORRECTED_P_VALUE), Univariate Cox Regression (Adj.COX_P_VALUE) on gene expression, Univariate Cox Regression on ranks of gene expression (Adj.CoxPvalbyRanks), Median cutpoint based logrank test (Adj.MedianPvals), tertile cutpoint based (top quantile vs bottom quantile) logrank test (Adj.tertPvals). (C). Average AUCs of ROC analysis for each method in each subset of total trials of data defined by 100 trials of TCGA data combined with one of the indicated PrognoScan datasets in column “PrognoScan_Datasets” (also see Materials and methods section for details). Last column”MaxAUCsMethods” showed the methods with the maximal AUCs in each row. (D). examples of ROC plots showing AUCs of GradientScanSurv method are largest in these trials.

| **Table D. Summary of the methods for each trial of tests with the top AUC comparing with other methods, from which Fig 5B is created** | | | |
| --- | --- | --- | --- |
| TCGA_TrainSet | PrognoScan_TestSet | TopAUCMethod | TopAUCMethodAUCs |
| TCGA_trial_1 | GSE13213.Surv | Adj.GoodCountPvals | 0.846201 |
| TCGA_trial_1 | GSE31210.Surv | Adj.GoodCountPvals | 0.851744 |
| TCGA_trial_1 | GSE31210.SurvRelapseFree | Adj.GoodCountPvals | 0.829651 |
| TCGA_trial_1 | HARVARD-LC.Surv | Adj.GoodCountPvals | 0.807692 |
| TCGA_trial_1 | jacob-00182-CANDF.Surv | Adj.GoodCountPvals | 0.778995 |
| TCGA_trial_1 | jacob-00182-HLM.Surv | Adj.tertPvals | 0.912437 |
| TCGA_trial_1 | jacob-00182-MSK.Surv | Adj.GoodCountPvals | 0.816026 |
| TCGA_trial_1 | jacob-00182-UM.Surv | Adj.CORRECTED_P_VALUE | 0.539894 |
| TCGA_trial_1 | MICHIGAN-LC.Surv | Adj.CoxPvalbyRanks | 0.660714 |
| TCGA_trial_2 | GSE13213.Surv | Adj.GoodCountPvals | 0.776847 |
| TCGA_trial_2 | GSE31210.Surv | Adj.GoodCountPvals | 0.755607 |
| TCGA_trial_2 | GSE31210.SurvRelapseFree | Adj.GoodCountPvals | 0.754673 |
| TCGA_trial_2 | HARVARD-LC.Surv | Adj.COX_P_VALUE | 0.616818 |
| TCGA_trial_2 | jacob-00182-CANDF.Surv | Adj.GoodCountPvals | 0.754404 |
| TCGA_trial_2 | jacob-00182-HLM.Surv | Adj.tertPvals | 0.912437 |
| TCGA_trial_2 | jacob-00182-MSK.Surv | Adj.GoodCountPvals | 0.953093 |
| TCGA_trial_2 | jacob-00182-UM.Surv | Adj.CORRECTED_P_VALUE | 0.539894 |
| TCGA_trial_2 | MICHIGAN-LC.Surv | Adj.CoxPvalbyRanks | 0.660714 |
| TCGA_trial_3 | GSE13213.Surv | Adj.GoodCountPvals | 0.747727 |
| TCGA_trial_3 | GSE31210.Surv | Adj.GoodCountPvals | 0.730144 |
| TCGA_trial_3 | GSE31210.SurvRelapseFree | Adj.GoodCountPvals | 0.71866 |
| TCGA_trial_3 | HARVARD-LC.Surv | Adj.COX_P_VALUE | 0.616818 |
| TCGA_trial_3 | jacob-00182-CANDF.Surv | Adj.GoodCountPvals | 0.757496 |
| TCGA_trial_3 | jacob-00182-HLM.Surv | Adj.tertPvals | 0.912437 |
| TCGA_trial_3 | jacob-00182-MSK.Surv | Adj.GoodCountPvals | 0.798538 |
| TCGA_trial_3 | jacob-00182-UM.Surv | Adj.CORRECTED_P_VALUE | 0.539894 |
| TCGA_trial_3 | MICHIGAN-LC.Surv | Adj.CoxPvalbyRanks | 0.660714 |
| TCGA_trial_4 | GSE13213.Surv | Adj.GoodCountPvals | 0.688721 |
| TCGA_trial_4 | GSE31210.Surv | Adj.CORRECTED_P_VALUE | 0.654187 |
| TCGA_trial_4 | GSE31210.SurvRelapseFree | Adj.GoodCountPvals | 0.601323 |
| TCGA_trial_4 | HARVARD-LC.Surv | Adj.COX_P_VALUE | 0.616818 |
| TCGA_trial_4 | jacob-00182-CANDF.Surv | Adj.GoodCountPvals | 0.704605 |
| TCGA_trial_4 | jacob-00182-HLM.Surv | Adj.tertPvals | 0.912437 |
| TCGA_trial_4 | jacob-00182-MSK.Surv | Adj.GoodCountPvals | 0.668521 |
| TCGA_trial_4 | jacob-00182-UM.Surv | Adj.GoodCountPvals | 0.63089 |
| TCGA_trial_4 | MICHIGAN-LC.Surv | Adj.CoxPvalbyRanks | 0.660714 |
| TCGA_trial_5 | GSE13213.Surv | Adj.GoodCountPvals | 0.87335 |
| TCGA_trial_5 | GSE31210.Surv | Adj.GoodCountPvals | 0.722222 |
| TCGA_trial_5 | GSE31210.SurvRelapseFree | Adj.GoodCountPvals | 0.730438 |
| TCGA_trial_5 | HARVARD-LC.Surv | Adj.COX_P_VALUE | 0.616818 |
| TCGA_trial_5 | jacob-00182-CANDF.Surv | Adj.GoodCountPvals | 0.782986 |
| TCGA_trial_5 | jacob-00182-HLM.Surv | Adj.tertPvals | 0.912437 |
| TCGA_trial_5 | jacob-00182-MSK.Surv | Adj.GoodCountPvals | 0.784974 |
| TCGA_trial_5 | jacob-00182-UM.Surv | Adj.GoodCountPvals | 0.624784 |
| TCGA_trial_5 | MICHIGAN-LC.Surv | Adj.CoxPvalbyRanks | 0.660714 |
| TCGA_trial_6 | GSE13213.Surv | Adj.GoodCountPvals | 0.740924 |
| TCGA_trial_6 | GSE31210.Surv | Adj.CORRECTED_P_VALUE | 0.654187 |
| TCGA_trial_6 | GSE31210.SurvRelapseFree | Adj.GoodCountPvals | 0.630673 |
| TCGA_trial_6 | HARVARD-LC.Surv | Adj.COX_P_VALUE | 0.616818 |
| TCGA_trial_6 | jacob-00182-CANDF.Surv | Adj.GoodCountPvals | 0.720207 |
| TCGA_trial_6 | jacob-00182-HLM.Surv | Adj.tertPvals | 0.912437 |
| TCGA_trial_6 | jacob-00182-MSK.Surv | Adj.GoodCountPvals | 0.772165 |
| TCGA_trial_6 | jacob-00182-UM.Surv | Adj.GoodCountPvals | 0.68299 |
| TCGA_trial_6 | MICHIGAN-LC.Surv | Adj.CoxPvalbyRanks | 0.660714 |
| TCGA_trial_7 | GSE13213.Surv | Adj.GoodCountPvals | 0.738384 |
| TCGA_trial_7 | GSE31210.Surv | Adj.CORRECTED_P_VALUE | 0.654187 |
| TCGA_trial_7 | GSE31210.SurvRelapseFree | Adj.GoodCountPvals | 0.629904 |
| TCGA_trial_7 | HARVARD-LC.Surv | Adj.COX_P_VALUE | 0.616818 |
| TCGA_trial_7 | jacob-00182-CANDF.Surv | Adj.GoodCountPvals | 0.702822 |
| TCGA_trial_7 | jacob-00182-HLM.Surv | Adj.tertPvals | 0.912437 |
| TCGA_trial_7 | jacob-00182-MSK.Surv | Adj.GoodCountPvals | 0.769883 |
| TCGA_trial_7 | jacob-00182-UM.Surv | Adj.CORRECTED_P_VALUE | 0.539894 |
| TCGA_trial_7 | MICHIGAN-LC.Surv | Adj.CoxPvalbyRanks | 0.660714 |
| TCGA_trial_8 | GSE13213.Surv | Adj.GoodCountPvals | 0.666667 |
| TCGA_trial_8 | GSE31210.Surv | Adj.GoodCountPvals | 0.657277 |
| TCGA_trial_8 | GSE31210.SurvRelapseFree | Adj.GoodCountPvals | 0.635759 |
| TCGA_trial_8 | HARVARD-LC.Surv | Adj.COX_P_VALUE | 0.616818 |
| TCGA_trial_8 | jacob-00182-CANDF.Surv | Adj.GoodCountPvals | 0.694878 |
| TCGA_trial_8 | jacob-00182-HLM.Surv | Adj.tertPvals | 0.912437 |
| TCGA_trial_8 | jacob-00182-MSK.Surv | Adj.GoodCountPvals | 0.802677 |
| TCGA_trial_8 | jacob-00182-UM.Surv | Adj.GoodCountPvals | 0.652418 |
| TCGA_trial_8 | MICHIGAN-LC.Surv | Adj.CoxPvalbyRanks | 0.660714 |
| TCGA_trial_9 | GSE13213.Surv | Adj.GoodCountPvals | 0.652273 |
| TCGA_trial_9 | GSE31210.Surv | Adj.GoodCountPvals | 0.683493 |
| TCGA_trial_9 | GSE31210.SurvRelapseFree | Adj.GoodCountPvals | 0.594976 |
| TCGA_trial_9 | HARVARD-LC.Surv | Adj.COX_P_VALUE | 0.616818 |
| TCGA_trial_9 | jacob-00182-CANDF.Surv | Adj.GoodCountPvals | 0.725798 |
| TCGA_trial_9 | jacob-00182-HLM.Surv | Adj.tertPvals | 0.912437 |
| TCGA_trial_9 | jacob-00182-MSK.Surv | Adj.GoodCountPvals | 0.644444 |
| TCGA_trial_9 | jacob-00182-UM.Surv | Adj.CORRECTED_P_VALUE | 0.539894 |
| TCGA_trial_9 | MICHIGAN-LC.Surv | Adj.CoxPvalbyRanks | 0.660714 |
| TCGA_trial_10 | GSE13213.Surv | Adj.GoodCountPvals | 0.755723 |
| TCGA_trial_10 | GSE31210.Surv | Adj.CORRECTED_P_VALUE | 0.654187 |
| TCGA_trial_10 | GSE31210.SurvRelapseFree | Adj.GoodCountPvals | 0.627778 |
| TCGA_trial_10 | HARVARD-LC.Surv | Adj.GoodCountPvals | 0.628655 |
| TCGA_trial_10 | jacob-00182-CANDF.Surv | Adj.tertPvals | 0.673469 |
| TCGA_trial_10 | jacob-00182-HLM.Surv | Adj.tertPvals | 0.912437 |
| TCGA_trial_10 | jacob-00182-MSK.Surv | Adj.GoodCountPvals | 0.621053 |
| TCGA_trial_10 | jacob-00182-UM.Surv | Adj.CORRECTED_P_VALUE | 0.539894 |
| TCGA_trial_10 | MICHIGAN-LC.Surv | Adj.CoxPvalbyRanks | 0.660714 |
| TCGA_trial_11 | GSE13213.Surv | Adj.GoodCountPvals | 0.722222 |
| TCGA_trial_11 | GSE31210.Surv | Adj.CORRECTED_P_VALUE | 0.654187 |
| TCGA_trial_11 | GSE31210.SurvRelapseFree | Adj.GoodCountPvals | 0.601323 |
| TCGA_trial_11 | HARVARD-LC.Surv | Adj.COX_P_VALUE | 0.616818 |
| TCGA_trial_11 | jacob-00182-CANDF.Surv | Adj.GoodCountPvals | 0.702822 |
| TCGA_trial_11 | jacob-00182-HLM.Surv | Adj.tertPvals | 0.912437 |
| TCGA_trial_11 | jacob-00182-MSK.Surv | Adj.GoodCountPvals | 0.769883 |
| TCGA_trial_11 | jacob-00182-UM.Surv | Adj.CORRECTED_P_VALUE | 0.539894 |
| TCGA_trial_11 | MICHIGAN-LC.Surv | Adj.CoxPvalbyRanks | 0.660714 |
| TCGA_trial_12 | GSE13213.Surv | Adj.GoodCountPvals | 0.857635 |
| TCGA_trial_12 | GSE31210.Surv | Adj.GoodCountPvals | 0.671495 |
| TCGA_trial_12 | GSE31210.SurvRelapseFree | Adj.GoodCountPvals | 0.700467 |
| TCGA_trial_12 | HARVARD-LC.Surv | Adj.COX_P_VALUE | 0.616818 |
| TCGA_trial_12 | jacob-00182-CANDF.Surv | Adj.GoodCountPvals | 0.740415 |
| TCGA_trial_12 | jacob-00182-HLM.Surv | Adj.tertPvals | 0.912437 |
| TCGA_trial_12 | jacob-00182-MSK.Surv | Adj.GoodCountPvals | 0.760309 |
| TCGA_trial_12 | jacob-00182-UM.Surv | Adj.GoodCountPvals | 0.741237 |
| TCGA_trial_12 | MICHIGAN-LC.Surv | Adj.CoxPvalbyRanks | 0.660714 |
| TCGA_trial_13 | GSE13213.Surv | Adj.GoodCountPvals | 0.726108 |
| TCGA_trial_13 | GSE31210.Surv | Adj.GoodCountPvals | 0.703738 |
| TCGA_trial_13 | GSE31210.SurvRelapseFree | Adj.GoodCountPvals | 0.67243 |
| TCGA_trial_13 | HARVARD-LC.Surv | Adj.COX_P_VALUE | 0.616818 |
| TCGA_trial_13 | jacob-00182-CANDF.Surv | Adj.GoodCountPvals | 0.697165 |
| TCGA_trial_13 | jacob-00182-HLM.Surv | Adj.tertPvals | 0.912437 |
| TCGA_trial_13 | jacob-00182-MSK.Surv | Adj.tertPvals | 0.614213 |
| TCGA_trial_13 | jacob-00182-UM.Surv | Adj.GoodCountPvals | 0.623718 |
| TCGA_trial_13 | MICHIGAN-LC.Surv | Adj.CoxPvalbyRanks | 0.660714 |
| TCGA_trial_14 | GSE13213.Surv | Adj.GoodCountPvals | 0.835749 |
| TCGA_trial_14 | GSE31210.Surv | Adj.GoodCountPvals | 0.711009 |
| TCGA_trial_14 | GSE31210.SurvRelapseFree | Adj.GoodCountPvals | 0.954128 |
| TCGA_trial_14 | HARVARD-LC.Surv | Adj.GoodCountPvals | 0.92437 |
| TCGA_trial_14 | jacob-00182-CANDF.Surv | Adj.tertPvals | 0.673469 |
| TCGA_trial_14 | jacob-00182-HLM.Surv | Adj.tertPvals | 0.912437 |
| TCGA_trial_14 | jacob-00182-MSK.Surv | Adj.GoodCountPvals | 0.90404 |
| TCGA_trial_14 | jacob-00182-UM.Surv | Adj.GoodCountPvals | 0.909091 |
| TCGA_trial_14 | MICHIGAN-LC.Surv | Adj.GoodCountPvals | 0.729412 |
| TCGA_trial_15 | GSE13213.Surv | Adj.GoodCountPvals | 0.620197 |
| TCGA_trial_15 | GSE31210.Surv | Adj.CORRECTED_P_VALUE | 0.654187 |
| TCGA_trial_15 | GSE31210.SurvRelapseFree | Adj.COX_P_VALUE | 0.590274 |
| TCGA_trial_15 | HARVARD-LC.Surv | Adj.COX_P_VALUE | 0.616818 |
| TCGA_trial_15 | jacob-00182-CANDF.Surv | Adj.tertPvals | 0.673469 |
| TCGA_trial_15 | jacob-00182-HLM.Surv | Adj.tertPvals | 0.912437 |
| TCGA_trial_15 | jacob-00182-MSK.Surv | Adj.GoodCountPvals | 0.772165 |
| TCGA_trial_15 | jacob-00182-UM.Surv | Adj.GoodCountPvals | 0.68299 |
| TCGA_trial_15 | MICHIGAN-LC.Surv | Adj.CoxPvalbyRanks | 0.660714 |
| TCGA_trial_16 | GSE13213.Surv | Adj.GoodCountPvals | 0.761576 |
| TCGA_trial_16 | GSE31210.Surv | Adj.GoodCountPvals | 0.69486 |
| TCGA_trial_16 | GSE31210.SurvRelapseFree | Adj.GoodCountPvals | 0.754673 |
| TCGA_trial_16 | HARVARD-LC.Surv | Adj.COX_P_VALUE | 0.616818 |
| TCGA_trial_16 | jacob-00182-CANDF.Surv | Adj.GoodCountPvals | 0.730026 |
| TCGA_trial_16 | jacob-00182-HLM.Surv | Adj.tertPvals | 0.912437 |
| TCGA_trial_16 | jacob-00182-MSK.Surv | Adj.GoodCountPvals | 0.941026 |
| TCGA_trial_16 | jacob-00182-UM.Surv | Adj.GoodCountPvals | 0.623718 |
| TCGA_trial_16 | MICHIGAN-LC.Surv | Adj.CoxPvalbyRanks | 0.660714 |
| TCGA_trial_17 | GSE13213.Surv | Adj.GoodCountPvals | 0.677188 |
| TCGA_trial_17 | GSE31210.Surv | Adj.CORRECTED_P_VALUE | 0.654187 |
| TCGA_trial_17 | GSE31210.SurvRelapseFree | Adj.COX_P_VALUE | 0.590274 |
| TCGA_trial_17 | HARVARD-LC.Surv | Adj.COX_P_VALUE | 0.616818 |
| TCGA_trial_17 | jacob-00182-CANDF.Surv | Adj.tertPvals | 0.673469 |
| TCGA_trial_17 | jacob-00182-HLM.Surv | Adj.tertPvals | 0.912437 |
| TCGA_trial_17 | jacob-00182-MSK.Surv | Adj.GoodCountPvals | 0.624673 |
| TCGA_trial_17 | jacob-00182-UM.Surv | Adj.GoodCountPvals | 0.625 |
| TCGA_trial_17 | MICHIGAN-LC.Surv | Adj.CoxPvalbyRanks | 0.660714 |
| TCGA_trial_18 | GSE13213.Surv | Adj.GoodCountPvals | 0.714778 |
| TCGA_trial_18 | GSE31210.Surv | Adj.CORRECTED_P_VALUE | 0.654187 |
| TCGA_trial_18 | GSE31210.SurvRelapseFree | Adj.COX_P_VALUE | 0.590274 |
| TCGA_trial_18 | HARVARD-LC.Surv | Adj.COX_P_VALUE | 0.616818 |
| TCGA_trial_18 | jacob-00182-CANDF.Surv | Adj.GoodCountPvals | 0.720207 |
| TCGA_trial_18 | jacob-00182-HLM.Surv | Adj.tertPvals | 0.912437 |
| TCGA_trial_18 | jacob-00182-MSK.Surv | Adj.GoodCountPvals | 0.772165 |
| TCGA_trial_18 | jacob-00182-UM.Surv | Adj.GoodCountPvals | 0.68299 |
| TCGA_trial_18 | MICHIGAN-LC.Surv | Adj.CoxPvalbyRanks | 0.660714 |
| TCGA_trial_19 | GSE13213.Surv | Adj.GoodCountPvals | 0.623312 |
| TCGA_trial_19 | GSE31210.Surv | Adj.CORRECTED_P_VALUE | 0.654187 |
| TCGA_trial_19 | GSE31210.SurvRelapseFree | Adj.COX_P_VALUE | 0.590274 |
| TCGA_trial_19 | HARVARD-LC.Surv | Adj.GoodCountPvals | 0.703664 |
| TCGA_trial_19 | jacob-00182-CANDF.Surv | Adj.tertPvals | 0.673469 |
| TCGA_trial_19 | jacob-00182-HLM.Surv | Adj.tertPvals | 0.912437 |
| TCGA_trial_19 | jacob-00182-MSK.Surv | Adj.GoodCountPvals | 0.633929 |
| TCGA_trial_19 | jacob-00182-UM.Surv | Adj.GoodCountPvals | 0.624628 |
| TCGA_trial_19 | MICHIGAN-LC.Surv | Adj.CoxPvalbyRanks | 0.660714 |
| TCGA_trial_20 | GSE13213.Surv | Adj.GoodCountPvals | 0.709158 |
| TCGA_trial_20 | GSE31210.Surv | Adj.GoodCountPvals | 0.655712 |
| TCGA_trial_20 | GSE31210.SurvRelapseFree | Adj.GoodCountPvals | 0.602113 |
| TCGA_trial_20 | HARVARD-LC.Surv | Adj.COX_P_VALUE | 0.616818 |
| TCGA_trial_20 | jacob-00182-CANDF.Surv | Adj.GoodCountPvals | 0.730035 |
| TCGA_trial_20 | jacob-00182-HLM.Surv | Adj.tertPvals | 0.912437 |
| TCGA_trial_20 | jacob-00182-MSK.Surv | Adj.GoodCountPvals | 0.716753 |
| TCGA_trial_20 | jacob-00182-UM.Surv | Adj.GoodCountPvals | 0.603627 |
| TCGA_trial_20 | MICHIGAN-LC.Surv | Adj.CoxPvalbyRanks | 0.660714 |
| TCGA_trial_21 | GSE13213.Surv | Adj.GoodCountPvals | 0.714778 |
| TCGA_trial_21 | GSE31210.Surv | Adj.CORRECTED_P_VALUE | 0.654187 |
| TCGA_trial_21 | GSE31210.SurvRelapseFree | Adj.COX_P_VALUE | 0.590274 |
| TCGA_trial_21 | HARVARD-LC.Surv | Adj.COX_P_VALUE | 0.616818 |
| TCGA_trial_21 | jacob-00182-CANDF.Surv | Adj.GoodCountPvals | 0.720207 |
| TCGA_trial_21 | jacob-00182-HLM.Surv | Adj.tertPvals | 0.912437 |
| TCGA_trial_21 | jacob-00182-MSK.Surv | Adj.GoodCountPvals | 0.772165 |
| TCGA_trial_21 | jacob-00182-UM.Surv | Adj.GoodCountPvals | 0.68299 |
| TCGA_trial_21 | MICHIGAN-LC.Surv | Adj.CoxPvalbyRanks | 0.660714 |
| TCGA_trial_22 | GSE13213.Surv | Adj.GoodCountPvals | 0.697376 |
| TCGA_trial_22 | GSE31210.Surv | Adj.GoodCountPvals | 0.658201 |
| TCGA_trial_22 | GSE31210.SurvRelapseFree | Adj.GoodCountPvals | 0.601323 |
| TCGA_trial_22 | HARVARD-LC.Surv | Adj.COX_P_VALUE | 0.616818 |
| TCGA_trial_22 | jacob-00182-CANDF.Surv | Adj.GoodCountPvals | 0.738095 |
| TCGA_trial_22 | jacob-00182-HLM.Surv | Adj.tertPvals | 0.912437 |
| TCGA_trial_22 | jacob-00182-MSK.Surv | Adj.GoodCountPvals | 0.695906 |
| TCGA_trial_22 | jacob-00182-UM.Surv | Adj.CORRECTED_P_VALUE | 0.539894 |
| TCGA_trial_22 | MICHIGAN-LC.Surv | Adj.CoxPvalbyRanks | 0.660714 |
| TCGA_trial_23 | GSE13213.Surv | Adj.GoodCountPvals | 0.658138 |
| TCGA_trial_23 | GSE31210.Surv | Adj.CORRECTED_P_VALUE | 0.654187 |
| TCGA_trial_23 | GSE31210.SurvRelapseFree | Adj.COX_P_VALUE | 0.590274 |
| TCGA_trial_23 | HARVARD-LC.Surv | Adj.GoodCountPvals | 0.671336 |
| TCGA_trial_23 | jacob-00182-CANDF.Surv | Adj.tertPvals | 0.673469 |
| TCGA_trial_23 | jacob-00182-HLM.Surv | Adj.tertPvals | 0.912437 |
| TCGA_trial_23 | jacob-00182-MSK.Surv | Adj.GoodCountPvals | 0.658895 |
| TCGA_trial_23 | jacob-00182-UM.Surv | Adj.GoodCountPvals | 0.72323 |
| TCGA_trial_23 | MICHIGAN-LC.Surv | Adj.CoxPvalbyRanks | 0.660714 |
| TCGA_trial_24 | GSE13213.Surv | Adj.GoodCountPvals | 0.699609 |
| TCGA_trial_24 | GSE31210.Surv | Adj.CORRECTED_P_VALUE | 0.654187 |
| TCGA_trial_24 | GSE31210.SurvRelapseFree | Adj.COX_P_VALUE | 0.590274 |
| TCGA_trial_24 | HARVARD-LC.Surv | Adj.COX_P_VALUE | 0.616818 |
| TCGA_trial_24 | jacob-00182-CANDF.Surv | Adj.tertPvals | 0.673469 |
| TCGA_trial_24 | jacob-00182-HLM.Surv | Adj.tertPvals | 0.912437 |
| TCGA_trial_24 | jacob-00182-MSK.Surv | Adj.GoodCountPvals | 0.624673 |
| TCGA_trial_24 | jacob-00182-UM.Surv | Adj.GoodCountPvals | 0.625 |
| TCGA_trial_24 | MICHIGAN-LC.Surv | Adj.CoxPvalbyRanks | 0.660714 |
| TCGA_trial_25 | GSE13213.Surv | Adj.GoodCountPvals | 0.723737 |
| TCGA_trial_25 | GSE31210.Surv | Adj.GoodCountPvals | 0.696172 |
| TCGA_trial_25 | GSE31210.SurvRelapseFree | Adj.GoodCountPvals | 0.61244 |
| TCGA_trial_25 | HARVARD-LC.Surv | Adj.COX_P_VALUE | 0.616818 |
| TCGA_trial_25 | jacob-00182-CANDF.Surv | Adj.GoodCountPvals | 0.725798 |
| TCGA_trial_25 | jacob-00182-HLM.Surv | Adj.tertPvals | 0.912437 |
| TCGA_trial_25 | jacob-00182-MSK.Surv | Adj.GoodCountPvals | 0.73254 |
| TCGA_trial_25 | jacob-00182-UM.Surv | Adj.CORRECTED_P_VALUE | 0.539894 |
| TCGA_trial_25 | MICHIGAN-LC.Surv | Adj.CoxPvalbyRanks | 0.660714 |
| TCGA_trial_26 | GSE13213.Surv | Adj.GoodCountPvals | 0.802345 |
| TCGA_trial_26 | GSE31210.Surv | Adj.GoodCountPvals | 0.662698 |
| TCGA_trial_26 | GSE31210.SurvRelapseFree | Adj.GoodCountPvals | 0.696032 |
| TCGA_trial_26 | HARVARD-LC.Surv | Adj.COX_P_VALUE | 0.616818 |
| TCGA_trial_26 | jacob-00182-CANDF.Surv | Adj.GoodCountPvals | 0.740789 |
| TCGA_trial_26 | jacob-00182-HLM.Surv | Adj.tertPvals | 0.912437 |
| TCGA_trial_26 | jacob-00182-MSK.Surv | Adj.GoodCountPvals | 0.854385 |
| TCGA_trial_26 | jacob-00182-UM.Surv | Adj.CORRECTED_P_VALUE | 0.539894 |
| TCGA_trial_26 | MICHIGAN-LC.Surv | Adj.CoxPvalbyRanks | 0.660714 |
| TCGA_trial_27 | GSE13213.Surv | Adj.GoodCountPvals | 0.726942 |
| TCGA_trial_27 | GSE31210.Surv | Adj.CORRECTED_P_VALUE | 0.654187 |
| TCGA_trial_27 | GSE31210.SurvRelapseFree | Adj.COX_P_VALUE | 0.590274 |
| TCGA_trial_27 | HARVARD-LC.Surv | Adj.GoodCountPvals | 0.840336 |
| TCGA_trial_27 | jacob-00182-CANDF.Surv | Adj.tertPvals | 0.673469 |
| TCGA_trial_27 | jacob-00182-HLM.Surv | Adj.tertPvals | 0.912437 |
| TCGA_trial_27 | jacob-00182-MSK.Surv | Adj.tertPvals | 0.614213 |
| TCGA_trial_27 | jacob-00182-UM.Surv | Adj.GoodCountPvals | 0.911168 |
| TCGA_trial_27 | MICHIGAN-LC.Surv | Adj.CoxPvalbyRanks | 0.660714 |
| TCGA_trial_28 | GSE13213.Surv | Adj.GoodCountPvals | 0.74835 |
| TCGA_trial_28 | GSE31210.Surv | Adj.GoodCountPvals | 0.741784 |
| TCGA_trial_28 | GSE31210.SurvRelapseFree | Adj.GoodCountPvals | 0.747261 |
| TCGA_trial_28 | HARVARD-LC.Surv | Adj.COX_P_VALUE | 0.616818 |
| TCGA_trial_28 | jacob-00182-CANDF.Surv | Adj.GoodCountPvals | 0.739378 |
| TCGA_trial_28 | jacob-00182-HLM.Surv | Adj.tertPvals | 0.912437 |
| TCGA_trial_28 | jacob-00182-MSK.Surv | Adj.GoodCountPvals | 0.841237 |
| TCGA_trial_28 | jacob-00182-UM.Surv | Adj.CORRECTED_P_VALUE | 0.539894 |
| TCGA_trial_28 | MICHIGAN-LC.Surv | Adj.GoodCountPvals | 0.714286 |
| TCGA_trial_29 | GSE13213.Surv | Adj.GoodCountPvals | 0.620197 |
| TCGA_trial_29 | GSE31210.Surv | Adj.CORRECTED_P_VALUE | 0.654187 |
| TCGA_trial_29 | GSE31210.SurvRelapseFree | Adj.COX_P_VALUE | 0.590274 |
| TCGA_trial_29 | HARVARD-LC.Surv | Adj.COX_P_VALUE | 0.616818 |
| TCGA_trial_29 | jacob-00182-CANDF.Surv | Adj.tertPvals | 0.673469 |
| TCGA_trial_29 | jacob-00182-HLM.Surv | Adj.tertPvals | 0.912437 |
| TCGA_trial_29 | jacob-00182-MSK.Surv | Adj.GoodCountPvals | 0.772165 |
| TCGA_trial_29 | jacob-00182-UM.Surv | Adj.GoodCountPvals | 0.68299 |
| TCGA_trial_29 | MICHIGAN-LC.Surv | Adj.CoxPvalbyRanks | 0.660714 |
| TCGA_trial_30 | GSE13213.Surv | Adj.GoodCountPvals | 0.713568 |
| TCGA_trial_30 | GSE31210.Surv | Adj.CORRECTED_P_VALUE | 0.654187 |
| TCGA_trial_30 | GSE31210.SurvRelapseFree | Adj.GoodCountPvals | 0.601323 |
| TCGA_trial_30 | HARVARD-LC.Surv | Adj.COX_P_VALUE | 0.616818 |
| TCGA_trial_30 | jacob-00182-CANDF.Surv | Adj.GoodCountPvals | 0.682895 |
| TCGA_trial_30 | jacob-00182-HLM.Surv | Adj.tertPvals | 0.912437 |
| TCGA_trial_30 | jacob-00182-MSK.Surv | Adj.GoodCountPvals | 0.739529 |
| TCGA_trial_30 | jacob-00182-UM.Surv | Adj.GoodCountPvals | 0.617474 |
| TCGA_trial_30 | MICHIGAN-LC.Surv | Adj.CoxPvalbyRanks | 0.660714 |
| TCGA_trial_31 | GSE13213.Surv | Adj.GoodCountPvals | 0.71636 |
| TCGA_trial_31 | GSE31210.Surv | Adj.CORRECTED_P_VALUE | 0.654187 |
| TCGA_trial_31 | GSE31210.SurvRelapseFree | Adj.GoodCountPvals | 0.620635 |
| TCGA_trial_31 | HARVARD-LC.Surv | Adj.COX_P_VALUE | 0.616818 |
| TCGA_trial_31 | jacob-00182-CANDF.Surv | Adj.tertPvals | 0.673469 |
| TCGA_trial_31 | jacob-00182-HLM.Surv | Adj.tertPvals | 0.912437 |
| TCGA_trial_31 | jacob-00182-MSK.Surv | Adj.GoodCountPvals | 0.684503 |
| TCGA_trial_31 | jacob-00182-UM.Surv | Adj.GoodCountPvals | 0.64269 |
| TCGA_trial_31 | MICHIGAN-LC.Surv | Adj.CoxPvalbyRanks | 0.660714 |
| TCGA_trial_32 | GSE13213.Surv | Adj.GoodCountPvals | 0.61656 |
| TCGA_trial_32 | GSE31210.Surv | Adj.CORRECTED_P_VALUE | 0.654187 |
| TCGA_trial_32 | GSE31210.SurvRelapseFree | Adj.COX_P_VALUE | 0.590274 |
| TCGA_trial_32 | HARVARD-LC.Surv | Adj.COX_P_VALUE | 0.616818 |
| TCGA_trial_32 | jacob-00182-CANDF.Surv | Adj.tertPvals | 0.673469 |
| TCGA_trial_32 | jacob-00182-HLM.Surv | Adj.tertPvals | 0.912437 |
| TCGA_trial_32 | jacob-00182-MSK.Surv | Adj.GoodCountPvals | 0.633929 |
| TCGA_trial_32 | jacob-00182-UM.Surv | Adj.GoodCountPvals | 0.59747 |
| TCGA_trial_32 | MICHIGAN-LC.Surv | Adj.CoxPvalbyRanks | 0.660714 |
| TCGA_trial_33 | GSE13213.Surv | Adj.GoodCountPvals | 0.757653 |
| TCGA_trial_33 | GSE31210.Surv | Adj.CORRECTED_P_VALUE | 0.654187 |
| TCGA_trial_33 | GSE31210.SurvRelapseFree | Adj.GoodCountPvals | 0.636473 |
| TCGA_trial_33 | HARVARD-LC.Surv | Adj.COX_P_VALUE | 0.616818 |
| TCGA_trial_33 | jacob-00182-CANDF.Surv | Adj.tertPvals | 0.673469 |
| TCGA_trial_33 | jacob-00182-HLM.Surv | Adj.tertPvals | 0.912437 |
| TCGA_trial_33 | jacob-00182-MSK.Surv | Adj.GoodCountPvals | 0.651354 |
| TCGA_trial_33 | jacob-00182-UM.Surv | Adj.CORRECTED_P_VALUE | 0.539894 |
| TCGA_trial_33 | MICHIGAN-LC.Surv | Adj.CoxPvalbyRanks | 0.660714 |
| TCGA_trial_34 | GSE13213.Surv | Adj.GoodCountPvals | 0.802345 |
| TCGA_trial_34 | GSE31210.Surv | Adj.GoodCountPvals | 0.677778 |
| TCGA_trial_34 | GSE31210.SurvRelapseFree | Adj.GoodCountPvals | 0.696032 |
| TCGA_trial_34 | HARVARD-LC.Surv | Adj.COX_P_VALUE | 0.616818 |
| TCGA_trial_34 | jacob-00182-CANDF.Surv | Adj.GoodCountPvals | 0.758553 |
| TCGA_trial_34 | jacob-00182-HLM.Surv | Adj.tertPvals | 0.912437 |
| TCGA_trial_34 | jacob-00182-MSK.Surv | Adj.GoodCountPvals | 0.842605 |
| TCGA_trial_34 | jacob-00182-UM.Surv | Adj.CORRECTED_P_VALUE | 0.539894 |
| TCGA_trial_34 | MICHIGAN-LC.Surv | Adj.CoxPvalbyRanks | 0.660714 |
| TCGA_trial_35 | GSE13213.Surv | Adj.GoodCountPvals | 0.622525 |
| TCGA_trial_35 | GSE31210.Surv | Adj.CORRECTED_P_VALUE | 0.654187 |
| TCGA_trial_35 | GSE31210.SurvRelapseFree | Adj.COX_P_VALUE | 0.590274 |
| TCGA_trial_35 | HARVARD-LC.Surv | Adj.GoodCountPvals | 0.671336 |
| TCGA_trial_35 | jacob-00182-CANDF.Surv | Adj.tertPvals | 0.673469 |
| TCGA_trial_35 | jacob-00182-HLM.Surv | Adj.tertPvals | 0.912437 |
| TCGA_trial_35 | jacob-00182-MSK.Surv | Adj.GoodCountPvals | 0.658895 |
| TCGA_trial_35 | jacob-00182-UM.Surv | Adj.GoodCountPvals | 0.72323 |
| TCGA_trial_35 | MICHIGAN-LC.Surv | Adj.CoxPvalbyRanks | 0.660714 |
| TCGA_trial_36 | GSE13213.Surv | Adj.GoodCountPvals | 0.797561 |
| TCGA_trial_36 | GSE31210.Surv | Adj.CORRECTED_P_VALUE | 0.654187 |
| TCGA_trial_36 | GSE31210.SurvRelapseFree | Adj.COX_P_VALUE | 0.590274 |
| TCGA_trial_36 | HARVARD-LC.Surv | Adj.GoodCountPvals | 0.840336 |
| TCGA_trial_36 | jacob-00182-CANDF.Surv | Adj.tertPvals | 0.673469 |
| TCGA_trial_36 | jacob-00182-HLM.Surv | Adj.tertPvals | 0.912437 |
| TCGA_trial_36 | jacob-00182-MSK.Surv | Adj.GoodCountPvals | 0.635204 |
| TCGA_trial_36 | jacob-00182-UM.Surv | Adj.GoodCountPvals | 0.913265 |
| TCGA_trial_36 | MICHIGAN-LC.Surv | Adj.CoxPvalbyRanks | 0.660714 |
| TCGA_trial_37 | GSE13213.Surv | Adj.GoodCountPvals | 0.697376 |
| TCGA_trial_37 | GSE31210.Surv | Adj.CORRECTED_P_VALUE | 0.654187 |
| TCGA_trial_37 | GSE31210.SurvRelapseFree | Adj.GoodCountPvals | 0.601323 |
| TCGA_trial_37 | HARVARD-LC.Surv | Adj.COX_P_VALUE | 0.616818 |
| TCGA_trial_37 | jacob-00182-CANDF.Surv | Adj.GoodCountPvals | 0.722222 |
| TCGA_trial_37 | jacob-00182-HLM.Surv | Adj.tertPvals | 0.912437 |
| TCGA_trial_37 | jacob-00182-MSK.Surv | Adj.GoodCountPvals | 0.706433 |
| TCGA_trial_37 | jacob-00182-UM.Surv | Adj.CORRECTED_P_VALUE | 0.539894 |
| TCGA_trial_37 | MICHIGAN-LC.Surv | Adj.CoxPvalbyRanks | 0.660714 |
| TCGA_trial_38 | GSE13213.Surv | Adj.GoodCountPvals | 0.662402 |
| TCGA_trial_38 | GSE31210.Surv | Adj.CORRECTED_P_VALUE | 0.654187 |
| TCGA_trial_38 | GSE31210.SurvRelapseFree | Adj.COX_P_VALUE | 0.590274 |
| TCGA_trial_38 | HARVARD-LC.Surv | Adj.COX_P_VALUE | 0.616818 |
| TCGA_trial_38 | jacob-00182-CANDF.Surv | Adj.tertPvals | 0.673469 |
| TCGA_trial_38 | jacob-00182-HLM.Surv | Adj.tertPvals | 0.912437 |
| TCGA_trial_38 | jacob-00182-MSK.Surv | Adj.GoodCountPvals | 0.707961 |
| TCGA_trial_38 | jacob-00182-UM.Surv | Adj.GoodCountPvals | 0.634301 |
| TCGA_trial_38 | MICHIGAN-LC.Surv | Adj.CoxPvalbyRanks | 0.660714 |
| TCGA_trial_39 | GSE13213.Surv | Adj.GoodCountPvals | 0.728788 |
| TCGA_trial_39 | GSE31210.Surv | Adj.CORRECTED_P_VALUE | 0.654187 |
| TCGA_trial_39 | GSE31210.SurvRelapseFree | Adj.COX_P_VALUE | 0.590274 |
| TCGA_trial_39 | HARVARD-LC.Surv | Adj.COX_P_VALUE | 0.616818 |
| TCGA_trial_39 | jacob-00182-CANDF.Surv | Adj.tertPvals | 0.673469 |
| TCGA_trial_39 | jacob-00182-HLM.Surv | Adj.tertPvals | 0.912437 |
| TCGA_trial_39 | jacob-00182-MSK.Surv | Adj.GoodCountPvals | 0.701852 |
| TCGA_trial_39 | jacob-00182-UM.Surv | Adj.CORRECTED_P_VALUE | 0.539894 |
| TCGA_trial_39 | MICHIGAN-LC.Surv | Adj.CoxPvalbyRanks | 0.660714 |
| TCGA_trial_40 | GSE13213.Surv | Adj.GoodCountPvals | 0.741347 |
| TCGA_trial_40 | GSE31210.Surv | Adj.GoodCountPvals | 0.699082 |
| TCGA_trial_40 | GSE31210.SurvRelapseFree | Adj.GoodCountPvals | 0.637675 |
| TCGA_trial_40 | HARVARD-LC.Surv | Adj.COX_P_VALUE | 0.616818 |
| TCGA_trial_40 | jacob-00182-CANDF.Surv | Adj.GoodCountPvals | 0.73894 |
| TCGA_trial_40 | jacob-00182-HLM.Surv | Adj.tertPvals | 0.912437 |
| TCGA_trial_40 | jacob-00182-MSK.Surv | Adj.GoodCountPvals | 0.754352 |
| TCGA_trial_40 | jacob-00182-UM.Surv | Adj.CORRECTED_P_VALUE | 0.539894 |
| TCGA_trial_40 | MICHIGAN-LC.Surv | Adj.CoxPvalbyRanks | 0.660714 |
| TCGA_trial_41 | GSE13213.Surv | Adj.GoodCountPvals | 0.678113 |
| TCGA_trial_41 | GSE31210.Surv | Adj.CORRECTED_P_VALUE | 0.654187 |
| TCGA_trial_41 | GSE31210.SurvRelapseFree | Adj.COX_P_VALUE | 0.590274 |
| TCGA_trial_41 | HARVARD-LC.Surv | Adj.COX_P_VALUE | 0.616818 |
| TCGA_trial_41 | jacob-00182-CANDF.Surv | Adj.tertPvals | 0.673469 |
| TCGA_trial_41 | jacob-00182-HLM.Surv | Adj.tertPvals | 0.912437 |
| TCGA_trial_41 | jacob-00182-MSK.Surv | Adj.tertPvals | 0.614213 |
| TCGA_trial_41 | jacob-00182-UM.Surv | Adj.CORRECTED_P_VALUE | 0.539894 |
| TCGA_trial_41 | MICHIGAN-LC.Surv | Adj.CoxPvalbyRanks | 0.660714 |
| TCGA_trial_42 | GSE13213.Surv | Adj.GoodCountPvals | 0.658138 |
| TCGA_trial_42 | GSE31210.Surv | Adj.CORRECTED_P_VALUE | 0.654187 |
| TCGA_trial_42 | GSE31210.SurvRelapseFree | Adj.COX_P_VALUE | 0.590274 |
| TCGA_trial_42 | HARVARD-LC.Surv | Adj.GoodCountPvals | 0.671336 |
| TCGA_trial_42 | jacob-00182-CANDF.Surv | Adj.tertPvals | 0.673469 |
| TCGA_trial_42 | jacob-00182-HLM.Surv | Adj.tertPvals | 0.912437 |
| TCGA_trial_42 | jacob-00182-MSK.Surv | Adj.GoodCountPvals | 0.658895 |
| TCGA_trial_42 | jacob-00182-UM.Surv | Adj.GoodCountPvals | 0.72323 |
| TCGA_trial_42 | MICHIGAN-LC.Surv | Adj.CoxPvalbyRanks | 0.660714 |
| TCGA_trial_43 | GSE13213.Surv | Adj.GoodCountPvals | 0.714778 |
| TCGA_trial_43 | GSE31210.Surv | Adj.CORRECTED_P_VALUE | 0.654187 |
| TCGA_trial_43 | GSE31210.SurvRelapseFree | Adj.COX_P_VALUE | 0.590274 |
| TCGA_trial_43 | HARVARD-LC.Surv | Adj.COX_P_VALUE | 0.616818 |
| TCGA_trial_43 | jacob-00182-CANDF.Surv | Adj.GoodCountPvals | 0.720207 |
| TCGA_trial_43 | jacob-00182-HLM.Surv | Adj.tertPvals | 0.912437 |
| TCGA_trial_43 | jacob-00182-MSK.Surv | Adj.GoodCountPvals | 0.772165 |
| TCGA_trial_43 | jacob-00182-UM.Surv | Adj.GoodCountPvals | 0.68299 |
| TCGA_trial_43 | MICHIGAN-LC.Surv | Adj.CoxPvalbyRanks | 0.660714 |
| TCGA_trial_44 | GSE13213.Surv | Adj.GoodCountPvals | 0.6283 |
| TCGA_trial_44 | GSE31210.Surv | Adj.CORRECTED_P_VALUE | 0.654187 |
| TCGA_trial_44 | GSE31210.SurvRelapseFree | Adj.COX_P_VALUE | 0.590274 |
| TCGA_trial_44 | HARVARD-LC.Surv | Adj.GoodCountPvals | 0.671336 |
| TCGA_trial_44 | jacob-00182-CANDF.Surv | Adj.GoodCountPvals | 0.688601 |
| TCGA_trial_44 | jacob-00182-HLM.Surv | Adj.tertPvals | 0.912437 |
| TCGA_trial_44 | jacob-00182-MSK.Surv | Adj.tertPvals | 0.614213 |
| TCGA_trial_44 | jacob-00182-UM.Surv | Adj.GoodCountPvals | 0.68299 |
| TCGA_trial_44 | MICHIGAN-LC.Surv | Adj.GoodCountPvals | 0.699405 |
| TCGA_trial_45 | GSE13213.Surv | Adj.GoodCountPvals | 0.725427 |
| TCGA_trial_45 | GSE31210.Surv | Adj.CORRECTED_P_VALUE | 0.654187 |
| TCGA_trial_45 | GSE31210.SurvRelapseFree | Adj.COX_P_VALUE | 0.590274 |
| TCGA_trial_45 | HARVARD-LC.Surv | Adj.COX_P_VALUE | 0.616818 |
| TCGA_trial_45 | jacob-00182-CANDF.Surv | Adj.tertPvals | 0.673469 |
| TCGA_trial_45 | jacob-00182-HLM.Surv | Adj.tertPvals | 0.912437 |
| TCGA_trial_45 | jacob-00182-MSK.Surv | Adj.GoodCountPvals | 0.678191 |
| TCGA_trial_45 | jacob-00182-UM.Surv | Adj.CORRECTED_P_VALUE | 0.539894 |
| TCGA_trial_45 | MICHIGAN-LC.Surv | Adj.CoxPvalbyRanks | 0.660714 |
| TCGA_trial_46 | GSE13213.Surv | Adj.GoodCountPvals | 0.64878 |
| TCGA_trial_46 | GSE31210.Surv | Adj.GoodCountPvals | 0.699846 |
| TCGA_trial_46 | GSE31210.SurvRelapseFree | Adj.GoodCountPvals | 0.673611 |
| TCGA_trial_46 | HARVARD-LC.Surv | Adj.GoodCountPvals | 0.773109 |
| TCGA_trial_46 | jacob-00182-CANDF.Surv | Adj.tertPvals | 0.673469 |
| TCGA_trial_46 | jacob-00182-HLM.Surv | Adj.tertPvals | 0.912437 |
| TCGA_trial_46 | jacob-00182-MSK.Surv | Adj.GoodCountPvals | 0.937925 |
| TCGA_trial_46 | jacob-00182-UM.Surv | Adj.GoodCountPvals | 0.677721 |
| TCGA_trial_46 | MICHIGAN-LC.Surv | Adj.CoxPvalbyRanks | 0.660714 |
| TCGA_trial_47 | GSE13213.Surv | Adj.GoodCountPvals | 0.688721 |
| TCGA_trial_47 | GSE31210.Surv | Adj.CORRECTED_P_VALUE | 0.654187 |
| TCGA_trial_47 | GSE31210.SurvRelapseFree | Adj.COX_P_VALUE | 0.590274 |
| TCGA_trial_47 | HARVARD-LC.Surv | Adj.COX_P_VALUE | 0.616818 |
| TCGA_trial_47 | jacob-00182-CANDF.Surv | Adj.tertPvals | 0.673469 |
| TCGA_trial_47 | jacob-00182-HLM.Surv | Adj.tertPvals | 0.912437 |
| TCGA_trial_47 | jacob-00182-MSK.Surv | Adj.GoodCountPvals | 0.674411 |
| TCGA_trial_47 | jacob-00182-UM.Surv | Adj.GoodCountPvals | 0.580497 |
| TCGA_trial_47 | MICHIGAN-LC.Surv | Adj.CoxPvalbyRanks | 0.660714 |
| TCGA_trial_48 | GSE13213.Surv | Adj.GoodCountPvals | 0.778336 |
| TCGA_trial_48 | GSE31210.Surv | Adj.GoodCountPvals | 0.72381 |
| TCGA_trial_48 | GSE31210.SurvRelapseFree | Adj.GoodCountPvals | 0.649471 |
| TCGA_trial_48 | HARVARD-LC.Surv | Adj.COX_P_VALUE | 0.616818 |
| TCGA_trial_48 | jacob-00182-CANDF.Surv | Adj.GoodCountPvals | 0.721634 |
| TCGA_trial_48 | jacob-00182-HLM.Surv | Adj.tertPvals | 0.912437 |
| TCGA_trial_48 | jacob-00182-MSK.Surv | Adj.GoodCountPvals | 0.694444 |
| TCGA_trial_48 | jacob-00182-UM.Surv | Adj.CORRECTED_P_VALUE | 0.539894 |
| TCGA_trial_48 | MICHIGAN-LC.Surv | Adj.CoxPvalbyRanks | 0.660714 |
| TCGA_trial_49 | GSE13213.Surv | Adj.GoodCountPvals | 0.701284 |
| TCGA_trial_49 | GSE31210.Surv | Adj.CORRECTED_P_VALUE | 0.654187 |
| TCGA_trial_49 | GSE31210.SurvRelapseFree | Adj.GoodCountPvals | 0.601323 |
| TCGA_trial_49 | HARVARD-LC.Surv | Adj.COX_P_VALUE | 0.616818 |
| TCGA_trial_49 | jacob-00182-CANDF.Surv | Adj.GoodCountPvals | 0.742434 |
| TCGA_trial_49 | jacob-00182-HLM.Surv | Adj.tertPvals | 0.912437 |
| TCGA_trial_49 | jacob-00182-MSK.Surv | Adj.GoodCountPvals | 0.674411 |
| TCGA_trial_49 | jacob-00182-UM.Surv | Adj.CORRECTED_P_VALUE | 0.539894 |
| TCGA_trial_49 | MICHIGAN-LC.Surv | Adj.CoxPvalbyRanks | 0.660714 |
| TCGA_trial_50 | GSE13213.Surv | Adj.GoodCountPvals | 0.715657 |
| TCGA_trial_50 | GSE31210.Surv | Adj.CORRECTED_P_VALUE | 0.654187 |
| TCGA_trial_50 | GSE31210.SurvRelapseFree | Adj.COX_P_VALUE | 0.590274 |
| TCGA_trial_50 | HARVARD-LC.Surv | Adj.COX_P_VALUE | 0.616818 |
| TCGA_trial_50 | jacob-00182-CANDF.Surv | Adj.tertPvals | 0.673469 |
| TCGA_trial_50 | jacob-00182-HLM.Surv | Adj.tertPvals | 0.912437 |
| TCGA_trial_50 | jacob-00182-MSK.Surv | Adj.tertPvals | 0.614213 |
| TCGA_trial_50 | jacob-00182-UM.Surv | Adj.GoodCountPvals | 0.62807 |
| TCGA_trial_50 | MICHIGAN-LC.Surv | Adj.CoxPvalbyRanks | 0.660714 |
| TCGA_trial_51 | GSE13213.Surv | Adj.GoodCountPvals | 0.688721 |
| TCGA_trial_51 | GSE31210.Surv | Adj.CORRECTED_P_VALUE | 0.654187 |
| TCGA_trial_51 | GSE31210.SurvRelapseFree | Adj.GoodCountPvals | 0.601323 |
| TCGA_trial_51 | HARVARD-LC.Surv | Adj.COX_P_VALUE | 0.616818 |
| TCGA_trial_51 | jacob-00182-CANDF.Surv | Adj.GoodCountPvals | 0.704605 |
| TCGA_trial_51 | jacob-00182-HLM.Surv | Adj.tertPvals | 0.912437 |
| TCGA_trial_51 | jacob-00182-MSK.Surv | Adj.GoodCountPvals | 0.668521 |
| TCGA_trial_51 | jacob-00182-UM.Surv | Adj.GoodCountPvals | 0.63089 |
| TCGA_trial_51 | MICHIGAN-LC.Surv | Adj.CoxPvalbyRanks | 0.660714 |
| TCGA_trial_52 | GSE13213.Surv | Adj.GoodCountPvals | 0.658138 |
| TCGA_trial_52 | GSE31210.Surv | Adj.CORRECTED_P_VALUE | 0.654187 |
| TCGA_trial_52 | GSE31210.SurvRelapseFree | Adj.COX_P_VALUE | 0.590274 |
| TCGA_trial_52 | HARVARD-LC.Surv | Adj.GoodCountPvals | 0.671336 |
| TCGA_trial_52 | jacob-00182-CANDF.Surv | Adj.tertPvals | 0.673469 |
| TCGA_trial_52 | jacob-00182-HLM.Surv | Adj.tertPvals | 0.912437 |
| TCGA_trial_52 | jacob-00182-MSK.Surv | Adj.GoodCountPvals | 0.658895 |
| TCGA_trial_52 | jacob-00182-UM.Surv | Adj.GoodCountPvals | 0.72323 |
| TCGA_trial_52 | MICHIGAN-LC.Surv | Adj.CoxPvalbyRanks | 0.660714 |
| TCGA_trial_53 | GSE13213.Surv | Adj.GoodCountPvals | 0.763054 |
| TCGA_trial_53 | GSE31210.Surv | Adj.GoodCountPvals | 0.724766 |
| TCGA_trial_53 | GSE31210.SurvRelapseFree | Adj.GoodCountPvals | 0.706542 |
| TCGA_trial_53 | HARVARD-LC.Surv | Adj.COX_P_VALUE | 0.616818 |
| TCGA_trial_53 | jacob-00182-CANDF.Surv | Adj.GoodCountPvals | 0.68342 |
| TCGA_trial_53 | jacob-00182-HLM.Surv | Adj.tertPvals | 0.912437 |
| TCGA_trial_53 | jacob-00182-MSK.Surv | Adj.GoodCountPvals | 0.760309 |
| TCGA_trial_53 | jacob-00182-UM.Surv | Adj.GoodCountPvals | 0.741237 |
| TCGA_trial_53 | MICHIGAN-LC.Surv | Adj.CoxPvalbyRanks | 0.660714 |
| TCGA_trial_54 | GSE13213.Surv | Adj.GoodCountPvals | 0.698485 |
| TCGA_trial_54 | GSE31210.Surv | Adj.CORRECTED_P_VALUE | 0.654187 |
| TCGA_trial_54 | GSE31210.SurvRelapseFree | Adj.COX_P_VALUE | 0.590274 |
| TCGA_trial_54 | HARVARD-LC.Surv | Adj.COX_P_VALUE | 0.616818 |
| TCGA_trial_54 | jacob-00182-CANDF.Surv | Adj.tertPvals | 0.673469 |
| TCGA_trial_54 | jacob-00182-HLM.Surv | Adj.tertPvals | 0.912437 |
| TCGA_trial_54 | jacob-00182-MSK.Surv | Adj.tertPvals | 0.614213 |
| TCGA_trial_54 | jacob-00182-UM.Surv | Adj.CORRECTED_P_VALUE | 0.539894 |
| TCGA_trial_54 | MICHIGAN-LC.Surv | Adj.CoxPvalbyRanks | 0.660714 |
| TCGA_trial_55 | GSE13213.Surv | Adj.GoodCountPvals | 0.706313 |
| TCGA_trial_55 | GSE31210.Surv | Adj.CORRECTED_P_VALUE | 0.654187 |
| TCGA_trial_55 | GSE31210.SurvRelapseFree | Adj.COX_P_VALUE | 0.590274 |
| TCGA_trial_55 | HARVARD-LC.Surv | Adj.COX_P_VALUE | 0.616818 |
| TCGA_trial_55 | jacob-00182-CANDF.Surv | Adj.tertPvals | 0.673469 |
| TCGA_trial_55 | jacob-00182-HLM.Surv | Adj.tertPvals | 0.912437 |
| TCGA_trial_55 | jacob-00182-MSK.Surv | Adj.GoodCountPvals | 0.644444 |
| TCGA_trial_55 | jacob-00182-UM.Surv | Adj.CORRECTED_P_VALUE | 0.539894 |
| TCGA_trial_55 | MICHIGAN-LC.Surv | Adj.CoxPvalbyRanks | 0.660714 |
| TCGA_trial_56 | GSE13213.Surv | Adj.GoodCountPvals | 0.630775 |
| TCGA_trial_56 | GSE31210.Surv | Adj.CORRECTED_P_VALUE | 0.654187 |
| TCGA_trial_56 | GSE31210.SurvRelapseFree | Adj.COX_P_VALUE | 0.590274 |
| TCGA_trial_56 | HARVARD-LC.Surv | Adj.COX_P_VALUE | 0.616818 |
| TCGA_trial_56 | jacob-00182-CANDF.Surv | Adj.GoodCountPvals | 0.682498 |
| TCGA_trial_56 | jacob-00182-HLM.Surv | Adj.tertPvals | 0.912437 |
| TCGA_trial_56 | jacob-00182-MSK.Surv | Adj.GoodCountPvals | 0.627232 |
| TCGA_trial_56 | jacob-00182-UM.Surv | Adj.GoodCountPvals | 0.649554 |
| TCGA_trial_56 | MICHIGAN-LC.Surv | Adj.CoxPvalbyRanks | 0.660714 |
| TCGA_trial_57 | GSE13213.Surv | Adj.GoodCountPvals | 0.668799 |
| TCGA_trial_57 | GSE31210.Surv | Adj.GoodCountPvals | 0.729447 |
| TCGA_trial_57 | GSE31210.SurvRelapseFree | Adj.GoodCountPvals | 0.621631 |
| TCGA_trial_57 | HARVARD-LC.Surv | Adj.COX_P_VALUE | 0.616818 |
| TCGA_trial_57 | jacob-00182-CANDF.Surv | Adj.tertPvals | 0.673469 |
| TCGA_trial_57 | jacob-00182-HLM.Surv | Adj.tertPvals | 0.912437 |
| TCGA_trial_57 | jacob-00182-MSK.Surv | Adj.GoodCountPvals | 0.757812 |
| TCGA_trial_57 | jacob-00182-UM.Surv | Adj.CORRECTED_P_VALUE | 0.539894 |
| TCGA_trial_57 | MICHIGAN-LC.Surv | Adj.CoxPvalbyRanks | 0.660714 |
| TCGA_trial_58 | GSE13213.Surv | Adj.GoodCountPvals | 0.764392 |
| TCGA_trial_58 | GSE31210.Surv | Adj.CORRECTED_P_VALUE | 0.654187 |
| TCGA_trial_58 | GSE31210.SurvRelapseFree | Adj.GoodCountPvals | 0.666442 |
| TCGA_trial_58 | HARVARD-LC.Surv | Adj.COX_P_VALUE | 0.616818 |
| TCGA_trial_58 | jacob-00182-CANDF.Surv | Adj.GoodCountPvals | 0.742622 |
| TCGA_trial_58 | jacob-00182-HLM.Surv | Adj.tertPvals | 0.912437 |
| TCGA_trial_58 | jacob-00182-MSK.Surv | Adj.GoodCountPvals | 0.802677 |
| TCGA_trial_58 | jacob-00182-UM.Surv | Adj.GoodCountPvals | 0.652418 |
| TCGA_trial_58 | MICHIGAN-LC.Surv | Adj.CoxPvalbyRanks | 0.660714 |
| TCGA_trial_59 | GSE13213.Surv | Adj.GoodCountPvals | 0.628019 |
| TCGA_trial_59 | GSE31210.Surv | Adj.GoodCountPvals | 0.972477 |
| TCGA_trial_59 | GSE31210.SurvRelapseFree | Adj.GoodCountPvals | 0.983945 |
| TCGA_trial_59 | HARVARD-LC.Surv | Adj.GoodCountPvals | 0.840336 |
| TCGA_trial_59 | jacob-00182-CANDF.Surv | Adj.tertPvals | 0.673469 |
| TCGA_trial_59 | jacob-00182-HLM.Surv | Adj.tertPvals | 0.912437 |
| TCGA_trial_59 | jacob-00182-MSK.Surv | Adj.GoodCountPvals | 0.90404 |
| TCGA_trial_59 | jacob-00182-UM.Surv | Adj.GoodCountPvals | 0.909091 |
| TCGA_trial_59 | MICHIGAN-LC.Surv | Adj.GoodCountPvals | 0.664706 |
| TCGA_trial_60 | GSE13213.Surv | Adj.GoodCountPvals | 0.884553 |
| TCGA_trial_60 | GSE31210.Surv | Adj.GoodCountPvals | 0.831019 |
| TCGA_trial_60 | GSE31210.SurvRelapseFree | Adj.GoodCountPvals | 0.87037 |
| TCGA_trial_60 | HARVARD-LC.Surv | Adj.GoodCountPvals | 0.794118 |
| TCGA_trial_60 | jacob-00182-CANDF.Surv | Adj.GoodCountPvals | 0.688889 |
| TCGA_trial_60 | jacob-00182-HLM.Surv | Adj.tertPvals | 0.912437 |
| TCGA_trial_60 | jacob-00182-MSK.Surv | Adj.GoodCountPvals | 0.918367 |
| TCGA_trial_60 | jacob-00182-UM.Surv | Adj.GoodCountPvals | 0.77381 |
| TCGA_trial_60 | MICHIGAN-LC.Surv | Adj.CoxPvalbyRanks | 0.660714 |
| TCGA_trial_61 | GSE13213.Surv | Adj.GoodCountPvals | 0.666667 |
| TCGA_trial_61 | GSE31210.Surv | Adj.GoodCountPvals | 0.657277 |
| TCGA_trial_61 | GSE31210.SurvRelapseFree | Adj.GoodCountPvals | 0.635759 |
| TCGA_trial_61 | HARVARD-LC.Surv | Adj.COX_P_VALUE | 0.616818 |
| TCGA_trial_61 | jacob-00182-CANDF.Surv | Adj.GoodCountPvals | 0.694878 |
| TCGA_trial_61 | jacob-00182-HLM.Surv | Adj.tertPvals | 0.912437 |
| TCGA_trial_61 | jacob-00182-MSK.Surv | Adj.GoodCountPvals | 0.802677 |
| TCGA_trial_61 | jacob-00182-UM.Surv | Adj.GoodCountPvals | 0.652418 |
| TCGA_trial_61 | MICHIGAN-LC.Surv | Adj.CoxPvalbyRanks | 0.660714 |
| TCGA_trial_62 | GSE13213.Surv | Adj.CORRECTED_P_VALUE | 0.614371 |
| TCGA_trial_62 | GSE31210.Surv | Adj.CORRECTED_P_VALUE | 0.654187 |
| TCGA_trial_62 | GSE31210.SurvRelapseFree | Adj.GoodCountPvals | 0.610329 |
| TCGA_trial_62 | HARVARD-LC.Surv | Adj.COX_P_VALUE | 0.616818 |
| TCGA_trial_62 | jacob-00182-CANDF.Surv | Adj.tertPvals | 0.673469 |
| TCGA_trial_62 | jacob-00182-HLM.Surv | Adj.tertPvals | 0.912437 |
| TCGA_trial_62 | jacob-00182-MSK.Surv | Adj.tertPvals | 0.614213 |
| TCGA_trial_62 | jacob-00182-UM.Surv | Adj.GoodCountPvals | 0.595855 |
| TCGA_trial_62 | MICHIGAN-LC.Surv | Adj.CoxPvalbyRanks | 0.660714 |
| TCGA_trial_63 | GSE13213.Surv | Adj.CORRECTED_P_VALUE | 0.614371 |
| TCGA_trial_63 | GSE31210.Surv | Adj.GoodCountPvals | 0.671963 |
| TCGA_trial_63 | GSE31210.SurvRelapseFree | Adj.COX_P_VALUE | 0.590274 |
| TCGA_trial_63 | HARVARD-LC.Surv | Adj.GoodCountPvals | 0.712251 |
| TCGA_trial_63 | jacob-00182-CANDF.Surv | Adj.tertPvals | 0.673469 |
| TCGA_trial_63 | jacob-00182-HLM.Surv | Adj.tertPvals | 0.912437 |
| TCGA_trial_63 | jacob-00182-MSK.Surv | Adj.tertPvals | 0.614213 |
| TCGA_trial_63 | jacob-00182-UM.Surv | Adj.GoodCountPvals | 0.631959 |
| TCGA_trial_63 | MICHIGAN-LC.Surv | Adj.CoxPvalbyRanks | 0.660714 |
| TCGA_trial_64 | GSE13213.Surv | Adj.GoodCountPvals | 0.622525 |
| TCGA_trial_64 | GSE31210.Surv | Adj.CORRECTED_P_VALUE | 0.654187 |
| TCGA_trial_64 | GSE31210.SurvRelapseFree | Adj.COX_P_VALUE | 0.590274 |
| TCGA_trial_64 | HARVARD-LC.Surv | Adj.GoodCountPvals | 0.671336 |
| TCGA_trial_64 | jacob-00182-CANDF.Surv | Adj.tertPvals | 0.673469 |
| TCGA_trial_64 | jacob-00182-HLM.Surv | Adj.tertPvals | 0.912437 |
| TCGA_trial_64 | jacob-00182-MSK.Surv | Adj.GoodCountPvals | 0.658895 |
| TCGA_trial_64 | jacob-00182-UM.Surv | Adj.GoodCountPvals | 0.72323 |
| TCGA_trial_64 | MICHIGAN-LC.Surv | Adj.CoxPvalbyRanks | 0.660714 |
| TCGA_trial_65 | GSE13213.Surv | Adj.CORRECTED_P_VALUE | 0.614371 |
| TCGA_trial_65 | GSE31210.Surv | Adj.CORRECTED_P_VALUE | 0.654187 |
| TCGA_trial_65 | GSE31210.SurvRelapseFree | Adj.COX_P_VALUE | 0.590274 |
| TCGA_trial_65 | HARVARD-LC.Surv | Adj.GoodCountPvals | 0.690733 |
| TCGA_trial_65 | jacob-00182-CANDF.Surv | Adj.tertPvals | 0.673469 |
| TCGA_trial_65 | jacob-00182-HLM.Surv | Adj.tertPvals | 0.912437 |
| TCGA_trial_65 | jacob-00182-MSK.Surv | Adj.GoodCountPvals | 0.658895 |
| TCGA_trial_65 | jacob-00182-UM.Surv | Adj.GoodCountPvals | 0.729275 |
| TCGA_trial_65 | MICHIGAN-LC.Surv | Adj.CoxPvalbyRanks | 0.660714 |
| TCGA_trial_66 | GSE13213.Surv | Adj.GoodCountPvals | 0.749747 |
| TCGA_trial_66 | GSE31210.Surv | Adj.CORRECTED_P_VALUE | 0.654187 |
| TCGA_trial_66 | GSE31210.SurvRelapseFree | Adj.GoodCountPvals | 0.629904 |
| TCGA_trial_66 | HARVARD-LC.Surv | Adj.COX_P_VALUE | 0.616818 |
| TCGA_trial_66 | jacob-00182-CANDF.Surv | Adj.GoodCountPvals | 0.752499 |
| TCGA_trial_66 | jacob-00182-HLM.Surv | Adj.tertPvals | 0.912437 |
| TCGA_trial_66 | jacob-00182-MSK.Surv | Adj.GoodCountPvals | 0.76462 |
| TCGA_trial_66 | jacob-00182-UM.Surv | Adj.CORRECTED_P_VALUE | 0.539894 |
| TCGA_trial_66 | MICHIGAN-LC.Surv | Adj.CoxPvalbyRanks | 0.660714 |
| TCGA_trial_67 | GSE13213.Surv | Adj.GoodCountPvals | 0.620197 |
| TCGA_trial_67 | GSE31210.Surv | Adj.CORRECTED_P_VALUE | 0.654187 |
| TCGA_trial_67 | GSE31210.SurvRelapseFree | Adj.COX_P_VALUE | 0.590274 |
| TCGA_trial_67 | HARVARD-LC.Surv | Adj.COX_P_VALUE | 0.616818 |
| TCGA_trial_67 | jacob-00182-CANDF.Surv | Adj.tertPvals | 0.673469 |
| TCGA_trial_67 | jacob-00182-HLM.Surv | Adj.tertPvals | 0.912437 |
| TCGA_trial_67 | jacob-00182-MSK.Surv | Adj.GoodCountPvals | 0.772165 |
| TCGA_trial_67 | jacob-00182-UM.Surv | Adj.GoodCountPvals | 0.68299 |
| TCGA_trial_67 | MICHIGAN-LC.Surv | Adj.CoxPvalbyRanks | 0.660714 |
| TCGA_trial_68 | GSE13213.Surv | Adj.GoodCountPvals | 0.71636 |
| TCGA_trial_68 | GSE31210.Surv | Adj.CORRECTED_P_VALUE | 0.654187 |
| TCGA_trial_68 | GSE31210.SurvRelapseFree | Adj.GoodCountPvals | 0.620635 |
| TCGA_trial_68 | HARVARD-LC.Surv | Adj.COX_P_VALUE | 0.616818 |
| TCGA_trial_68 | jacob-00182-CANDF.Surv | Adj.tertPvals | 0.673469 |
| TCGA_trial_68 | jacob-00182-HLM.Surv | Adj.tertPvals | 0.912437 |
| TCGA_trial_68 | jacob-00182-MSK.Surv | Adj.GoodCountPvals | 0.684503 |
| TCGA_trial_68 | jacob-00182-UM.Surv | Adj.GoodCountPvals | 0.64269 |
| TCGA_trial_68 | MICHIGAN-LC.Surv | Adj.CoxPvalbyRanks | 0.660714 |
| TCGA_trial_69 | GSE13213.Surv | Adj.GoodCountPvals | 0.633251 |
| TCGA_trial_69 | GSE31210.Surv | Adj.CORRECTED_P_VALUE | 0.654187 |
| TCGA_trial_69 | GSE31210.SurvRelapseFree | Adj.COX_P_VALUE | 0.590274 |
| TCGA_trial_69 | HARVARD-LC.Surv | Adj.COX_P_VALUE | 0.616818 |
| TCGA_trial_69 | jacob-00182-CANDF.Surv | Adj.GoodCountPvals | 0.716146 |
| TCGA_trial_69 | jacob-00182-HLM.Surv | Adj.tertPvals | 0.912437 |
| TCGA_trial_69 | jacob-00182-MSK.Surv | Adj.GoodCountPvals | 0.658895 |
| TCGA_trial_69 | jacob-00182-UM.Surv | Adj.GoodCountPvals | 0.652418 |
| TCGA_trial_69 | MICHIGAN-LC.Surv | Adj.GoodCountPvals | 0.690763 |
| TCGA_trial_70 | GSE13213.Surv | Adj.GoodCountPvals | 0.622525 |
| TCGA_trial_70 | GSE31210.Surv | Adj.CORRECTED_P_VALUE | 0.654187 |
| TCGA_trial_70 | GSE31210.SurvRelapseFree | Adj.COX_P_VALUE | 0.590274 |
| TCGA_trial_70 | HARVARD-LC.Surv | Adj.GoodCountPvals | 0.671336 |
| TCGA_trial_70 | jacob-00182-CANDF.Surv | Adj.tertPvals | 0.673469 |
| TCGA_trial_70 | jacob-00182-HLM.Surv | Adj.tertPvals | 0.912437 |
| TCGA_trial_70 | jacob-00182-MSK.Surv | Adj.GoodCountPvals | 0.658895 |
| TCGA_trial_70 | jacob-00182-UM.Surv | Adj.GoodCountPvals | 0.72323 |
| TCGA_trial_70 | MICHIGAN-LC.Surv | Adj.CoxPvalbyRanks | 0.660714 |
| TCGA_trial_71 | GSE13213.Surv | Adj.GoodCountPvals | 0.959951 |
| TCGA_trial_71 | GSE31210.Surv | Adj.GoodCountPvals | 0.860599 |
| TCGA_trial_71 | GSE31210.SurvRelapseFree | Adj.GoodCountPvals | 0.868664 |
| TCGA_trial_71 | HARVARD-LC.Surv | Adj.GoodCountPvals | 0.869748 |
| TCGA_trial_71 | jacob-00182-CANDF.Surv | Adj.GoodCountPvals | 0.809949 |
| TCGA_trial_71 | jacob-00182-HLM.Surv | Adj.tertPvals | 0.912437 |
| TCGA_trial_71 | jacob-00182-MSK.Surv | Adj.GoodCountPvals | 0.904822 |
| TCGA_trial_71 | jacob-00182-UM.Surv | Adj.GoodCountPvals | 0.685279 |
| TCGA_trial_71 | MICHIGAN-LC.Surv | Adj.CoxPvalbyRanks | 0.660714 |
| TCGA_trial_72 | GSE13213.Surv | Adj.GoodCountPvals | 0.728283 |
| TCGA_trial_72 | GSE31210.Surv | Adj.CORRECTED_P_VALUE | 0.654187 |
| TCGA_trial_72 | GSE31210.SurvRelapseFree | Adj.GoodCountPvals | 0.629904 |
| TCGA_trial_72 | HARVARD-LC.Surv | Adj.COX_P_VALUE | 0.616818 |
| TCGA_trial_72 | jacob-00182-CANDF.Surv | Adj.tertPvals | 0.673469 |
| TCGA_trial_72 | jacob-00182-HLM.Surv | Adj.tertPvals | 0.912437 |
| TCGA_trial_72 | jacob-00182-MSK.Surv | Adj.GoodCountPvals | 0.684503 |
| TCGA_trial_72 | jacob-00182-UM.Surv | Adj.GoodCountPvals | 0.621345 |
| TCGA_trial_72 | MICHIGAN-LC.Surv | Adj.CoxPvalbyRanks | 0.660714 |
| TCGA_trial_73 | GSE13213.Surv | Adj.GoodCountPvals | 0.695 |
| TCGA_trial_73 | GSE31210.Surv | Adj.CORRECTED_P_VALUE | 0.654187 |
| TCGA_trial_73 | GSE31210.SurvRelapseFree | Adj.COX_P_VALUE | 0.590274 |
| TCGA_trial_73 | HARVARD-LC.Surv | Adj.GoodCountPvals | 0.671336 |
| TCGA_trial_73 | jacob-00182-CANDF.Surv | Adj.GoodCountPvals | 0.693343 |
| TCGA_trial_73 | jacob-00182-HLM.Surv | Adj.tertPvals | 0.912437 |
| TCGA_trial_73 | jacob-00182-MSK.Surv | Adj.GoodCountPvals | 0.707961 |
| TCGA_trial_73 | jacob-00182-UM.Surv | Adj.GoodCountPvals | 0.661458 |
| TCGA_trial_73 | MICHIGAN-LC.Surv | Adj.CoxPvalbyRanks | 0.660714 |
| TCGA_trial_74 | GSE13213.Surv | Adj.GoodCountPvals | 0.753713 |
| TCGA_trial_74 | GSE31210.Surv | Adj.CORRECTED_P_VALUE | 0.654187 |
| TCGA_trial_74 | GSE31210.SurvRelapseFree | Adj.GoodCountPvals | 0.630673 |
| TCGA_trial_74 | HARVARD-LC.Surv | Adj.COX_P_VALUE | 0.616818 |
| TCGA_trial_74 | jacob-00182-CANDF.Surv | Adj.GoodCountPvals | 0.742622 |
| TCGA_trial_74 | jacob-00182-HLM.Surv | Adj.tertPvals | 0.912437 |
| TCGA_trial_74 | jacob-00182-MSK.Surv | Adj.GoodCountPvals | 0.810449 |
| TCGA_trial_74 | jacob-00182-UM.Surv | Adj.GoodCountPvals | 0.617444 |
| TCGA_trial_74 | MICHIGAN-LC.Surv | Adj.CoxPvalbyRanks | 0.660714 |
| TCGA_trial_75 | GSE13213.Surv | Adj.GoodCountPvals | 0.983092 |
| TCGA_trial_75 | GSE31210.Surv | Adj.GoodCountPvals | 0.830275 |
| TCGA_trial_75 | GSE31210.SurvRelapseFree | Adj.GoodCountPvals | 0.866972 |
| TCGA_trial_75 | HARVARD-LC.Surv | Adj.GoodCountPvals | 0.869748 |
| TCGA_trial_75 | jacob-00182-CANDF.Surv | Adj.GoodCountPvals | 0.944162 |
| TCGA_trial_75 | jacob-00182-HLM.Surv | Adj.tertPvals | 0.912437 |
| TCGA_trial_75 | jacob-00182-MSK.Surv | Adj.GoodCountPvals | 0.936869 |
| TCGA_trial_75 | jacob-00182-UM.Surv | Adj.GoodCountPvals | 0.540404 |
| TCGA_trial_75 | MICHIGAN-LC.Surv | Adj.CoxPvalbyRanks | 0.660714 |
| TCGA_trial_76 | GSE13213.Surv | Adj.GoodCountPvals | 0.6825 |
| TCGA_trial_76 | GSE31210.Surv | Adj.CORRECTED_P_VALUE | 0.654187 |
| TCGA_trial_76 | GSE31210.SurvRelapseFree | Adj.COX_P_VALUE | 0.590274 |
| TCGA_trial_76 | HARVARD-LC.Surv | Adj.GoodCountPvals | 0.671336 |
| TCGA_trial_76 | jacob-00182-CANDF.Surv | Adj.tertPvals | 0.673469 |
| TCGA_trial_76 | jacob-00182-HLM.Surv | Adj.tertPvals | 0.912437 |
| TCGA_trial_76 | jacob-00182-MSK.Surv | Adj.tertPvals | 0.614213 |
| TCGA_trial_76 | jacob-00182-UM.Surv | Adj.GoodCountPvals | 0.71317 |
| TCGA_trial_76 | MICHIGAN-LC.Surv | Adj.CoxPvalbyRanks | 0.660714 |
| TCGA_trial_77 | GSE13213.Surv | Adj.GoodCountPvals | 0.697376 |
| TCGA_trial_77 | GSE31210.Surv | Adj.GoodCountPvals | 0.663757 |
| TCGA_trial_77 | GSE31210.SurvRelapseFree | Adj.COX_P_VALUE | 0.590274 |
| TCGA_trial_77 | HARVARD-LC.Surv | Adj.COX_P_VALUE | 0.616818 |
| TCGA_trial_77 | jacob-00182-CANDF.Surv | Adj.GoodCountPvals | 0.694297 |
| TCGA_trial_77 | jacob-00182-HLM.Surv | Adj.tertPvals | 0.912437 |
| TCGA_trial_77 | jacob-00182-MSK.Surv | Adj.GoodCountPvals | 0.711696 |
| TCGA_trial_77 | jacob-00182-UM.Surv | Adj.CORRECTED_P_VALUE | 0.539894 |
| TCGA_trial_77 | MICHIGAN-LC.Surv | Adj.CoxPvalbyRanks | 0.660714 |
| TCGA_trial_78 | GSE13213.Surv | Adj.GoodCountPvals | 0.785625 |
| TCGA_trial_78 | GSE31210.Surv | Adj.GoodCountPvals | 0.664396 |
| TCGA_trial_78 | GSE31210.SurvRelapseFree | Adj.GoodCountPvals | 0.69372 |
| TCGA_trial_78 | HARVARD-LC.Surv | Adj.COX_P_VALUE | 0.616818 |
| TCGA_trial_78 | jacob-00182-CANDF.Surv | Adj.GoodCountPvals | 0.674671 |
| TCGA_trial_78 | jacob-00182-HLM.Surv | Adj.tertPvals | 0.912437 |
| TCGA_trial_78 | jacob-00182-MSK.Surv | Adj.GoodCountPvals | 0.758835 |
| TCGA_trial_78 | jacob-00182-UM.Surv | Adj.GoodCountPvals | 0.606675 |
| TCGA_trial_78 | MICHIGAN-LC.Surv | Adj.CoxPvalbyRanks | 0.660714 |
| TCGA_trial_79 | GSE13213.Surv | Adj.GoodCountPvals | 0.917073 |
| TCGA_trial_79 | GSE31210.Surv | Adj.GoodCountPvals | 0.871914 |
| TCGA_trial_79 | GSE31210.SurvRelapseFree | Adj.GoodCountPvals | 0.87037 |
| TCGA_trial_79 | HARVARD-LC.Surv | Adj.GoodCountPvals | 0.869748 |
| TCGA_trial_79 | jacob-00182-CANDF.Surv | Adj.GoodCountPvals | 0.723932 |
| TCGA_trial_79 | jacob-00182-HLM.Surv | Adj.tertPvals | 0.912437 |
| TCGA_trial_79 | jacob-00182-MSK.Surv | Adj.GoodCountPvals | 0.918367 |
| TCGA_trial_79 | jacob-00182-UM.Surv | Adj.GoodCountPvals | 0.761905 |
| TCGA_trial_79 | MICHIGAN-LC.Surv | Adj.CoxPvalbyRanks | 0.660714 |
| TCGA_trial_80 | GSE13213.Surv | Adj.GoodCountPvals | 0.713568 |
| TCGA_trial_80 | GSE31210.Surv | Adj.CORRECTED_P_VALUE | 0.654187 |
| TCGA_trial_80 | GSE31210.SurvRelapseFree | Adj.GoodCountPvals | 0.601323 |
| TCGA_trial_80 | HARVARD-LC.Surv | Adj.COX_P_VALUE | 0.616818 |
| TCGA_trial_80 | jacob-00182-CANDF.Surv | Adj.GoodCountPvals | 0.682895 |
| TCGA_trial_80 | jacob-00182-HLM.Surv | Adj.tertPvals | 0.912437 |
| TCGA_trial_80 | jacob-00182-MSK.Surv | Adj.GoodCountPvals | 0.739529 |
| TCGA_trial_80 | jacob-00182-UM.Surv | Adj.GoodCountPvals | 0.617474 |
| TCGA_trial_80 | MICHIGAN-LC.Surv | Adj.CoxPvalbyRanks | 0.660714 |
| TCGA_trial_81 | GSE13213.Surv | Adj.GoodCountPvals | 0.728283 |
| TCGA_trial_81 | GSE31210.Surv | Adj.CORRECTED_P_VALUE | 0.654187 |
| TCGA_trial_81 | GSE31210.SurvRelapseFree | Adj.GoodCountPvals | 0.629904 |
| TCGA_trial_81 | HARVARD-LC.Surv | Adj.COX_P_VALUE | 0.616818 |
| TCGA_trial_81 | jacob-00182-CANDF.Surv | Adj.tertPvals | 0.673469 |
| TCGA_trial_81 | jacob-00182-HLM.Surv | Adj.tertPvals | 0.912437 |
| TCGA_trial_81 | jacob-00182-MSK.Surv | Adj.GoodCountPvals | 0.684503 |
| TCGA_trial_81 | jacob-00182-UM.Surv | Adj.GoodCountPvals | 0.621345 |
| TCGA_trial_81 | MICHIGAN-LC.Surv | Adj.CoxPvalbyRanks | 0.660714 |
| TCGA_trial_82 | GSE13213.Surv | Adj.GoodCountPvals | 0.732765 |
| TCGA_trial_82 | GSE31210.Surv | Adj.CORRECTED_P_VALUE | 0.654187 |
| TCGA_trial_82 | GSE31210.SurvRelapseFree | Adj.GoodCountPvals | 0.641846 |
| TCGA_trial_82 | HARVARD-LC.Surv | Adj.COX_P_VALUE | 0.616818 |
| TCGA_trial_82 | jacob-00182-CANDF.Surv | Adj.GoodCountPvals | 0.730035 |
| TCGA_trial_82 | jacob-00182-HLM.Surv | Adj.tertPvals | 0.912437 |
| TCGA_trial_82 | jacob-00182-MSK.Surv | Adj.GoodCountPvals | 0.716753 |
| TCGA_trial_82 | jacob-00182-UM.Surv | Adj.GoodCountPvals | 0.603627 |
| TCGA_trial_82 | MICHIGAN-LC.Surv | Adj.CoxPvalbyRanks | 0.660714 |
| TCGA_trial_83 | GSE13213.Surv | Adj.GoodCountPvals | 0.665 |
| TCGA_trial_83 | GSE31210.Surv | Adj.CORRECTED_P_VALUE | 0.654187 |
| TCGA_trial_83 | GSE31210.SurvRelapseFree | Adj.COX_P_VALUE | 0.590274 |
| TCGA_trial_83 | HARVARD-LC.Surv | Adj.COX_P_VALUE | 0.616818 |
| TCGA_trial_83 | jacob-00182-CANDF.Surv | Adj.GoodCountPvals | 0.704605 |
| TCGA_trial_83 | jacob-00182-HLM.Surv | Adj.tertPvals | 0.912437 |
| TCGA_trial_83 | jacob-00182-MSK.Surv | Adj.GoodCountPvals | 0.668521 |
| TCGA_trial_83 | jacob-00182-UM.Surv | Adj.GoodCountPvals | 0.63089 |
| TCGA_trial_83 | MICHIGAN-LC.Surv | Adj.CoxPvalbyRanks | 0.660714 |
| TCGA_trial_84 | GSE13213.Surv | Adj.GoodCountPvals | 0.740924 |
| TCGA_trial_84 | GSE31210.Surv | Adj.CORRECTED_P_VALUE | 0.654187 |
| TCGA_trial_84 | GSE31210.SurvRelapseFree | Adj.GoodCountPvals | 0.630673 |
| TCGA_trial_84 | HARVARD-LC.Surv | Adj.COX_P_VALUE | 0.616818 |
| TCGA_trial_84 | jacob-00182-CANDF.Surv | Adj.GoodCountPvals | 0.720207 |
| TCGA_trial_84 | jacob-00182-HLM.Surv | Adj.tertPvals | 0.912437 |
| TCGA_trial_84 | jacob-00182-MSK.Surv | Adj.GoodCountPvals | 0.772165 |
| TCGA_trial_84 | jacob-00182-UM.Surv | Adj.GoodCountPvals | 0.68299 |
| TCGA_trial_84 | MICHIGAN-LC.Surv | Adj.CoxPvalbyRanks | 0.660714 |
| TCGA_trial_85 | GSE13213.Surv | Adj.GoodCountPvals | 0.85122 |
| TCGA_trial_85 | GSE31210.Surv | Adj.CORRECTED_P_VALUE | 0.654187 |
| TCGA_trial_85 | GSE31210.SurvRelapseFree | Adj.COX_P_VALUE | 0.590274 |
| TCGA_trial_85 | HARVARD-LC.Surv | Adj.COX_P_VALUE | 0.616818 |
| TCGA_trial_85 | jacob-00182-CANDF.Surv | Adj.GoodCountPvals | 0.765812 |
| TCGA_trial_85 | jacob-00182-HLM.Surv | Adj.tertPvals | 0.912437 |
| TCGA_trial_85 | jacob-00182-MSK.Surv | Adj.GoodCountPvals | 0.635204 |
| TCGA_trial_85 | jacob-00182-UM.Surv | Adj.GoodCountPvals | 0.761905 |
| TCGA_trial_85 | MICHIGAN-LC.Surv | Adj.GoodCountPvals | 0.664706 |
| TCGA_trial_86 | GSE13213.Surv | Adj.GoodCountPvals | 0.662402 |
| TCGA_trial_86 | GSE31210.Surv | Adj.CORRECTED_P_VALUE | 0.654187 |
| TCGA_trial_86 | GSE31210.SurvRelapseFree | Adj.COX_P_VALUE | 0.590274 |
| TCGA_trial_86 | HARVARD-LC.Surv | Adj.COX_P_VALUE | 0.616818 |
| TCGA_trial_86 | jacob-00182-CANDF.Surv | Adj.GoodCountPvals | 0.693343 |
| TCGA_trial_86 | jacob-00182-HLM.Surv | Adj.tertPvals | 0.912437 |
| TCGA_trial_86 | jacob-00182-MSK.Surv | Adj.GoodCountPvals | 0.701265 |
| TCGA_trial_86 | jacob-00182-UM.Surv | Adj.GoodCountPvals | 0.691592 |
| TCGA_trial_86 | MICHIGAN-LC.Surv | Adj.CoxPvalbyRanks | 0.660714 |
| TCGA_trial_87 | GSE13213.Surv | Adj.CORRECTED_P_VALUE | 0.614371 |
| TCGA_trial_87 | GSE31210.Surv | Adj.CORRECTED_P_VALUE | 0.654187 |
| TCGA_trial_87 | GSE31210.SurvRelapseFree | Adj.COX_P_VALUE | 0.590274 |
| TCGA_trial_87 | HARVARD-LC.Surv | Adj.GoodCountPvals | 0.851852 |
| TCGA_trial_87 | jacob-00182-CANDF.Surv | Adj.tertPvals | 0.673469 |
| TCGA_trial_87 | jacob-00182-HLM.Surv | Adj.tertPvals | 0.912437 |
| TCGA_trial_87 | jacob-00182-MSK.Surv | Adj.tertPvals | 0.614213 |
| TCGA_trial_87 | jacob-00182-UM.Surv | Adj.GoodCountPvals | 0.774742 |
| TCGA_trial_87 | MICHIGAN-LC.Surv | Adj.CoxPvalbyRanks | 0.660714 |
| TCGA_trial_88 | GSE13213.Surv | Adj.GoodCountPvals | 0.689062 |
| TCGA_trial_88 | GSE31210.Surv | Adj.CORRECTED_P_VALUE | 0.654187 |
| TCGA_trial_88 | GSE31210.SurvRelapseFree | Adj.COX_P_VALUE | 0.590274 |
| TCGA_trial_88 | HARVARD-LC.Surv | Adj.COX_P_VALUE | 0.616818 |
| TCGA_trial_88 | jacob-00182-CANDF.Surv | Adj.tertPvals | 0.673469 |
| TCGA_trial_88 | jacob-00182-HLM.Surv | Adj.tertPvals | 0.912437 |
| TCGA_trial_88 | jacob-00182-MSK.Surv | Adj.GoodCountPvals | 0.707961 |
| TCGA_trial_88 | jacob-00182-UM.Surv | Adj.GoodCountPvals | 0.634301 |
| TCGA_trial_88 | MICHIGAN-LC.Surv | Adj.CoxPvalbyRanks | 0.660714 |
| TCGA_trial_89 | GSE13213.Surv | Adj.GoodCountPvals | 0.662402 |
| TCGA_trial_89 | GSE31210.Surv | Adj.CORRECTED_P_VALUE | 0.654187 |
| TCGA_trial_89 | GSE31210.SurvRelapseFree | Adj.COX_P_VALUE | 0.590274 |
| TCGA_trial_89 | HARVARD-LC.Surv | Adj.COX_P_VALUE | 0.616818 |
| TCGA_trial_89 | jacob-00182-CANDF.Surv | Adj.GoodCountPvals | 0.693343 |
| TCGA_trial_89 | jacob-00182-HLM.Surv | Adj.tertPvals | 0.912437 |
| TCGA_trial_89 | jacob-00182-MSK.Surv | Adj.GoodCountPvals | 0.701265 |
| TCGA_trial_89 | jacob-00182-UM.Surv | Adj.GoodCountPvals | 0.691592 |
| TCGA_trial_89 | MICHIGAN-LC.Surv | Adj.CoxPvalbyRanks | 0.660714 |
| TCGA_trial_90 | GSE13213.Surv | Adj.GoodCountPvals | 0.718939 |
| TCGA_trial_90 | GSE31210.Surv | Adj.GoodCountPvals | 0.683493 |
| TCGA_trial_90 | GSE31210.SurvRelapseFree | Adj.GoodCountPvals | 0.61244 |
| TCGA_trial_90 | HARVARD-LC.Surv | Adj.COX_P_VALUE | 0.616818 |
| TCGA_trial_90 | jacob-00182-CANDF.Surv | Adj.GoodCountPvals | 0.725798 |
| TCGA_trial_90 | jacob-00182-HLM.Surv | Adj.tertPvals | 0.912437 |
| TCGA_trial_90 | jacob-00182-MSK.Surv | Adj.GoodCountPvals | 0.727778 |
| TCGA_trial_90 | jacob-00182-UM.Surv | Adj.CORRECTED_P_VALUE | 0.539894 |
| TCGA_trial_90 | MICHIGAN-LC.Surv | Adj.CoxPvalbyRanks | 0.660714 |
| TCGA_trial_91 | GSE13213.Surv | Adj.GoodCountPvals | 0.705808 |
| TCGA_trial_91 | GSE31210.Surv | Adj.CORRECTED_P_VALUE | 0.654187 |
| TCGA_trial_91 | GSE31210.SurvRelapseFree | Adj.GoodCountPvals | 0.61244 |
| TCGA_trial_91 | HARVARD-LC.Surv | Adj.COX_P_VALUE | 0.616818 |
| TCGA_trial_91 | jacob-00182-CANDF.Surv | Adj.tertPvals | 0.673469 |
| TCGA_trial_91 | jacob-00182-HLM.Surv | Adj.tertPvals | 0.912437 |
| TCGA_trial_91 | jacob-00182-MSK.Surv | Adj.GoodCountPvals | 0.626316 |
| TCGA_trial_91 | jacob-00182-UM.Surv | Adj.GoodCountPvals | 0.588304 |
| TCGA_trial_91 | MICHIGAN-LC.Surv | Adj.CoxPvalbyRanks | 0.660714 |
| TCGA_trial_92 | GSE13213.Surv | Adj.GoodCountPvals | 0.772194 |
| TCGA_trial_92 | GSE31210.Surv | Adj.GoodCountPvals | 0.677778 |
| TCGA_trial_92 | GSE31210.SurvRelapseFree | Adj.GoodCountPvals | 0.67672 |
| TCGA_trial_92 | HARVARD-LC.Surv | Adj.GoodCountPvals | 0.633772 |
| TCGA_trial_92 | jacob-00182-CANDF.Surv | Adj.GoodCountPvals | 0.73125 |
| TCGA_trial_92 | jacob-00182-HLM.Surv | Adj.tertPvals | 0.912437 |
| TCGA_trial_92 | jacob-00182-MSK.Surv | Adj.GoodCountPvals | 0.783377 |
| TCGA_trial_92 | jacob-00182-UM.Surv | Adj.CORRECTED_P_VALUE | 0.539894 |
| TCGA_trial_92 | MICHIGAN-LC.Surv | Adj.CoxPvalbyRanks | 0.660714 |
| TCGA_trial_93 | GSE13213.Surv | Adj.GoodCountPvals | 0.718872 |
| TCGA_trial_93 | GSE31210.Surv | Adj.CORRECTED_P_VALUE | 0.654187 |
| TCGA_trial_93 | GSE31210.SurvRelapseFree | Adj.GoodCountPvals | 0.620635 |
| TCGA_trial_93 | HARVARD-LC.Surv | Adj.COX_P_VALUE | 0.616818 |
| TCGA_trial_93 | jacob-00182-CANDF.Surv | Adj.GoodCountPvals | 0.714145 |
| TCGA_trial_93 | jacob-00182-HLM.Surv | Adj.tertPvals | 0.912437 |
[truncated: 245,444 more chars]
